# Deciphering the Role of *Emx1* in Neurogenesis: A Neuroproteomics Approach

**DOI:** 10.3389/fnmol.2016.00098

**Published:** 2016-10-17

**Authors:** Firas H. Kobeissy, Katharina Hansen, Melanie Neumann, Shuping Fu, Kulin Jin, Jialing Liu

**Affiliations:** ^1^Department of Psychiatry, Center for Neuroproteomics and Biomarkers Research, University of FloridaGainesville, FL, USA; ^2^Department of Neurological Surgery, University of California, San FranciscoSan Francisco, CA, USA; ^3^San Francisco VA Medical CenterSan Francisco, CA, USA; ^4^Key Laboratory of Acupuncture and Medicine Research of Minister of Education, Nanjing University of Chinese MedicineNanjing, China; ^5^Pharmacology & Neuroscience, University of North Texas Health Science CenterFort Worth, TX, USA

**Keywords:** cofilin, VEGF, neural stem cell, 2D-PAGE/MS-MS, Boyden chamber assay

## Abstract

*Emx1* has long been implicated in embryonic brain development. Previously we found that mice null of *Emx1* gene had smaller dentate gyri and reduced neurogenesis, although the molecular mechanisms underlying this defect was not well understood. To decipher the role of *Emx1* gene in neural regeneration and the timing of its involvement, we determine the frequency of neural stem cells (NSCs) in embryonic and adult forebrains of *Emx1* wild type (WT) and knock out (KO) mice in the neurosphere assay. *Emx1* gene deletion reduced the frequency and self-renewal capacity of NSCs of the embryonic brain but did not affect neuronal or glial differentiation. *Emx1* KO NSCs also exhibited a reduced migratory capacity in response to serum or vascular endothelial growth factor (VEGF) in the Boyden chamber migration assay compared to their WT counterparts. A thorough comparison between NSC lysates from *Emx1* WT and KO mice utilizing 2D-PAGE coupled with tandem mass spectrometry revealed 38 proteins differentially expressed between genotypes, including the F-actin depolymerization factor Cofilin. A global systems biology and cluster analysis identified several potential mechanisms and cellular pathways implicated in altered neurogenesis, all involving Cofilin1. Protein interaction network maps with functional enrichment analysis further indicated that the differentially expressed proteins participated in neural-specific functions including brain development, axonal guidance, synaptic transmission, neurogenesis, and hippocampal morphology, with VEGF as the upstream regulator intertwined with Cofilin1 and Emx1. Functional validation analysis indicated that apart from the overall reduced level of phosphorylated Cofilin1 (p-Cofilin1) in the *Emx1* KO NSCs compared to WT NSCs as demonstrated in the western blot analysis, VEGF was able to induce more Cofilin1 phosphorylation and FLK expression only in the latter. Our results suggest that a defect in Cofilin1 phosphorylation induced by VEGF or other growth factors might contribute to the reduced neurogenesis in the *Emx1* null mice during brain development.

## Introduction

Mammalian homeobox gene transcription factors including the *Emx, Hox, Pax*, and *Dlx*, have been implicated in the development of the forebrain (Boncinelli et al., 1995; Panganiban and Rubenstein, 2002; Gavalas et al., 2003; Muzio and Mallamaci, 2003; de Melo et al., 2005). Among which, the *Emx* family plays a crucial role in neurogenesis including neuronal migration, differentiation, and synaptic connectivity (Simeone et al., 1993). As the homolog of the *Drosophila melanogaster* empty spiracles ems gene, the *Emx* genes are well conserved in mammals and insects, sharing ~82% homology in amino acid identity with the ems homeodomains (Cecchi and Boncinelli, 2000).

Two of the *Emx1* isoforms, *Emx1* and *Emx2*, share a number of functional and structural characteristics with overlapping patterns of spatio-temporal expression (Simeone et al., 1992; Gulisano et al., 1996; Chan et al., 2001). Whereas, *Emx2* is expressed in the dorsal and ventral telencephalon and hypothalamus, *Emx1* is restricted to the dorsal telencephalon (Simeone et al., 1992). Single *Emx1* mutants showed a lack of corpus callosum with minimal structural or histological brain anomalies in contrast to the profound and severe developmental defects observed in *Emx2* mutants, such as disorganized olfactory bulb and reduced size of the cerebral cortex and hippocampus (Pellegrini et al., 1996; Qiu et al., 1996; Bishop et al., 2003). With respect to neurogenesis, we have demonstrated that *Emx1*, similar to *Emx2*, contributed to the genesis of the dentate gyrus (DG). Apart from the impairment in skill reaching learning, as well as blunted anxiety and depression (Cao and Li, 2002), the adult *Emx1* mutants exhibited smaller DG, coinciding with a decreased number of immature neurons and proliferating progenitor cells in the DG (Hong et al., 2007). However, the mechanism by which *Emx1* contributes to neurogenesis and the critical developmental stage involving *Emx1* is not well understood.

Neurogenesis encompasses several processes including the proliferation and differentiation of neural stem cells (NSCs), as well as the migration and survival of newborn neurons (Frisén, 2016; Liu and Song, 2016). Established evidence suggests that neuroblasts migrate in close association with blood vessels following the division of NSCs (Thored et al., 2007; Zhang et al., 2014). A number of vascular factors including VEGF contribute to the formation and maintenance of the neurovascular niche of neurogenesis (Li Q. et al., 2006; Madri, 2009; Ward and Cunningham, 2015). The current study sought to decipher the role of the *Emx1* gene in reduced neural regeneration and determine the putative mechanisms implicated by employing neuroproteomics-systems biology platforms and biochemical approaches. Our results indicate that *Emx1* gene deletion not only resulted in reduced embryonic neurosphere formation with diminished self-renewal capacity, but also negatively impacted the chemotaxic response of NSCs to VEGF or serum. Proteomics analysis suggests that the deficiency in Cofilin1 phosphorylation may underlie the defective signaling of NSCs in response to VEGF or other growth factors, potentially leading to abnormal forebrain development.

## Materials and methods

### Animals, housing, and ethical considerations

This study was conducted in accordance with the animal care guidelines issued by the National Institutes of Health and by the San Francisco Veterans Affairs Medical Center Animal Care and Use Committee. Adult mice heterozygous for *Emx1* gene derived from cryopreserved embryos at Jackson Laboratory were bred to generate heterozygous and homozygous offspring that were maintained in house in the institutional standard cages (4 mice per cage) on a 12-h light/12-h dark cycle, with *ad libitum* access to water and food before and during experimental procedures. All *in-vitro* and *in-vivo* procedures were conducted by examiners blinded to experimental conditions (for the detailed methodology of Western blot analysis and antibodies used, please refer to Data Sheet 1 in the Supplementary Material).

### Neural stem cell culture and neurosphere assay

Forebrains were dissected from *Emx1* WT or KO embryos at embryonic day 14 (E14) harvested from time-pregnant mice. Neurosphere assay for the adult mice (2.5 months of age) were conducted from the dissected subventricular zone (SVZ) or the hippocampus. After enzymatic digestion with Papain (10 U/ml) and DNase (0.5 mg/ml) (Sigma Aldrich, St. Louis, MO, USA) solution at 37°C for 20 min, brain tissue was mechanically triturated with a fire-polished Pasteur pipette into single cell suspension and resuspended in DMEM-F12 (1:1) medium in the presence of hEGF (20 ng/ml) or hEGF (20 ng/ml) + FGF (10 ng/ml) (Invitrogen, Eugene, Oregon, USA) and heparin (2 μg/ml) (StemCell Technologies, Inc., Vancouver, BC, Canada). Neural colony forming cell (NCFC) assay kit (StemCell Technologies, Inc., Vancouver, BC, Canada) was used for neurosphere assay. Cells were suspended in complete NeuroCult NCFC medium, and mixed with collagen solution, 7500 cells were dispensed into 35 mm culture dishes and cultured for 21 days. hEGF and FGF-2 (20 ng/ml each) was added into the semi-solid culture every 7 days. The diameters of spheres were measured using a 2 mm-grid dish on a Zeiss invert microscope. Remaining cells were suspended in proliferation medium composed of DMEM/F-12 (1:1) containing B27 supplement and 20 ng/ml hEGF, and were cultured in a T-75 tissue culture flask to form primary floating neurospheres. Floating neurospheres were passaged every 7 days.

### Neural stem cells self-renewal capacity

Primary neurospheres were mechanically dissociated into single cells suspension and ~400 viable cells were plated into each well of a 6-well cell culture plate containing proliferation medium. The number of secondary neurospheres generated in each well was counted 10 days after plating and averaged (*n* = 3–4 replicates/neurosphere samples/genotype).

### Neural stem cell differentiation assay and immunocytochemistry

Neurospheres were harvested after 2nd passage by centrifugation at 500 rpm for 5 min and resuspended in growth factor-free DMEM/F-12 (1:1) medium. Cells in neurospheres were mechanically dissociated by trituration and filtered through 0.45 μm cell strainer. Viable single cells (10^5^ cells/well) were plated on poly-D-lysine/laminin coated 6-well culture plate in DMEM-F12 (1:1) plus NeuroCult NSFC differentiation supplements for 10 days. The differentiated cells were fixed with 4% paraformaldehyde in 0.1 M PBS for 15 min, and then washed with PBS twice. Differentiated cells were immunostained with following reagents: mouse anti-GFAP; mouse anti-Tuj1; rat anti-nestin; Cy3 goat anti-mouse; biotinylated sheep anti-rat; Cy3 streptavidine. Cells were count stained with DNA dye Hoechst. Stained cells were photographed with a Zeiss invert fluorescence microscope. Differentiation ratio was calculated as immuno-positive cells divided by total cells stained with Hoechst.

### Boyden chamber chemotaxis assay

The migration of the NSCs in response to fetal calf serum (FCS) or recombinant human VEGF_165_ (R&D Systems, Minneapolis, USA) was assessed using a modified Boyden chamber assay as previously described (Lamszus et al., 1998; Schmidt et al., 2009). Briefly, quadruplicates of media containing 10% FCS or VEGF were added to the lower wells of a 96-well modified Boyden chamber (Neuro Probe, Gaithersburg, MD, USA), and wells were covered with an 8-μ m pore size Nucleopore filter that had previously been coated with 10 μg/ml laminin (Invitrogen, Eugene, Oregon, USA). NSCs from dissociated neurospheres passages 3–4 were then suspended at 2.5 × 10^4^ cells in 50 μl of either serum-free DMEM/F12 medium containing 0.1% bovine serum albumin (BSA) or in growth medium containing EGF alone or EGF+FGF and seeded into the upper wells. After incubation for 15 h at 37°C, non-migrated cells were scraped off the upper side of the filter and filters were stained with Diff-Quik (VWR, Brisbane, CA, USA). Nuclei of migrated cells were counted in an average of 180 high power fields (field size 20 × 20 to 100 × 100 μm) using a 40x objective by using the Stereo Investigator software (Stereo Investigator, MicroBrightField, VA). Values were assessed in quadruplicate from at least three independent experiments and expressed as mean ± standard deviation in percentage of the control migration (= 100%). The control migration rate for each assay was assessed in response to either serum-free medium containing 0.1% BSA or growth medium containing EGF or EGF+FGF.

### Two-dimensional polyacrylamide gel electrophoresis (2D-PAGE) sample preparation

Forty milliliters of NSCs (passage 2), seeded with a density of 100,000 cells/ml, were harvested after 5 days of growing in defined media by centrifugation at 400 × g. Cell pellets were lysed in 1 ml of lysis-buffer containing 9 M urea (Sigma), 4% (w/v) CHAPS (Calbiochem, San Diego, CA), 0.5% (v/v) pharmalytes pH 3–10 (Amersham Bioscience), 1 mM EDTA (American Bioanalytical, Natick, MA), Complete protease inhibitor tablets were from Roche (Indianapolis, IN). Homogenates were triturated using a syringe with 20G-needle and incubated for 1 h at RT. After samples were centrifuged at 4000 rpm for 10 min, supernatants were transferred into an empty 1.5 ml tube, while pellets were discarded. To determine protein concentrations the 2-D-Quant-protein concentration kit (Amersham Bioscience) was used following the instructor's guideline. The first dimension was performed using an Ettan IPGphor II isoelectric focusing unit (Amersham Bioscience). Five-hundred micrograms of protein sample were mixed with 0.2% (v/v) ampholytes (Amersham Bioscience) and 170 μl of 2 × rehydration-buffer containing 9 M urea, 2% (w/v) CHAPS, 0.002% (w/v) bromphenol blue, 18 mm of dithiothreitol (DTT (ThermoFisher), and 0.5% IPG-buffer 3–10 (Amersham Bioscience). To reach the final volume of 340 μl, lysis-buffer (see above) was added. Each sample was pipetted into a slot of the dehydration tray in which an 18 cm-Immobilene™ Dry Strip pH 3–10 (Amersham Bioscience) was placed upside down. In order to inhibit drying-out of the solution overnight each slot was covered with mineral oil (Amersham Bioscience). The strips passively rehydrated at constant 25°C for 18 h. Afterwards the strips were transferred to the focusing tray where the proteins were separated according to their isoelectric points using the following protocol: 1 h at 150 V, 3 h at 300 V, 6 h at 1000 V using the linear ramp, 3 h at 6000 V using the linear ramp, and 3 h at 6000 V. To inhibit diffusion of the proteins after focusing the machine held 50 V until manually stopped. After the focusing process strips were equilibrated two times for 10 min in a buffer containing 50 mM of Tris-HCl (pH 8.8), 6 M Urea, 30% (v/v) glycerol, 2% (w/v) SDS, 0.002% (w/v) bromphenol blue and either 65 mM DTT. Equilibrated strips were put on top of a 12% polyacrylamide gel, made by using standard laboratory procedures, and covered with 0.5% (w/v) melted agarose also containing 0.002% (w/v) bromphenol blue. Ten microliters of a molecular weight standard (BioRad, Hercules, CA) was dropped on a piece of blotting paper and was put next to the strip into the agarose solution before polymerization. Gels were run over night at 10 μA per gel at 4°C using an Ettan DALTsix electrophoreses unit (Amersham Bioscience, Piscataway, NJ; for the detailed methodology of Gel staining with silver or Coomassie Blue, please refer to Data Sheet 1 in the Supplementary Material).

### High performance liquid chromatography (HPLC) tandem mass spectrometry (MS/MS)

Peptide extracts were separated on-line by nanoLC utilizing a 2D LC NanoLC System (Eksigent/AB Sciex). Liquid chromatographic separations were performed using a PepMap (Dionex/LC Packings) trap column and a reversed phase nano-column (75 μm in diameter × 150 mm in height packed in-house with Jupiter Proteo C12 endcapped resin, 90 Å pore size, 4 μm particle size). An aliquot of peptide extract (3–4 μL) was loaded onto the trap column with loading solvent (0.1% formic acid) at a flow rate of 20 μL/min. The trap column was washed with the loading solvent for 3 min prior to switching it in line with the reversed phase nano-column. The nano-column and elution buffers were maintained at ambient temperature and mobile phase flow rate was 250 nL/min. The nano-column was equilibrated with 2% Solvent B in Solvent A for 20 min prior to sample injection (Solvent A: 2% acetonitrile/0.1% formic acid; Solvent B: 80% acetonitrile/0.1% formic acid). Peptide separation was accomplished using a binary gradient which consisted of a 5 min isocratic wash at 2% B followed by a linear gradient of 2–50% B over 45 min, and concluded with a column cleanup step of 95% B for 7 min. The nano-column was interfaced directly to a nanoelectrospray ion source (Protana) mounted on a QSTAR Elite quadrupole/quadrupole/time-of-flight (QqTOF) mass spectrometer (AB Sciex). Protein identification was accomplished by isolating sequentially eluting peptide populations with a single mass-to-charge ratio (m/z), within the mass spectrometer, fragmenting this population, and measuring the masses of the peptide fragment ions. Peak lists were generated with the Mascot Daemon (Matrix Sciences) and the Mascot v2.2 search engine (Matrix Science) was used to search the experimentally determined peptide fragment ion masses against a theoretical fragment ion mass database generated by *in silico* digestion and fragmentation of all proteins in the UniProtKB/Swiss-Prot Protein Knowledgebase (downloaded 03-09-2011; 525,997 protein sequences; 185,874,894 residues), taxonomy: rodentia (25,596 protein sequences). The following search parameters were utilized: precursor ion mass tolerance ± 150 ppm; fragment ion mass tolerance 0.15 Da; tryptic digestion; 2 missed cleavages; *p* < 0.05; ion score or expect cut-off 0.05; fixed modification: Cys-carbamidomethyl; variable modifications: deamidation (Asn and Gln), Met-sulfoxide, and Pyro-glu (N-term Gln; for the detailed methodology of In-gel digestion procedure, please refer to Data Sheet 1 in the Supplementary Material).

### Subnetwork enrichment pathway analyses and statistical testing

The Elsevier's Pathway Studio v. 10.0 (Ariadne Genomics/Elsevier) was used to deduce relationships among differentially expressed proteomics protein candidates using the Ariadne ResNet database (Bonnet et al., 2009; Yuryev et al., 2009). “Subnetwork Enrichment Analysis” (SNEA) algorithm was selected to extract statistically significant altered biological and functional pathways pertaining to each identified set of protein hits SNEA utilizes Fisher's statistical test used to determine if there are nonrandom associations between two categorical variables organized by specific relationship. SNEA starts by creating a central “seed” from all relevant entities in the database, and retrieving associated entities based on their relationship with the seed (that is, binding partners, expression targets, protein modification targets, regulation). The algorithm compares the sub-network distribution to the background distribution using one-sided Mann–Whitney *U*-Test, and calculates a *p*-value indicating the statistical significance of difference between two distributions. In our analysis, “GenBank ID symbols” of the 38 altered proteins from 2D-PAGE-MS/MS spectrometry data were imported to the software to form an experimental data set. For the reconstruction of networks of pathways, biological processes, and Molecular function were evaluated for each single protein hit and its associated targets (networks and pathways; Daraselia et al., 2012; Pyatnitskiy et al., 2014). Integrated Venn diagram analysis was performed using “the InteractiVenn”: a web-based tool for the analysis of comlex data sets. Green rectangles, violet rectangles, and blue hexagons reflect biological processes, disease processes, and functional classes, respectively. The different colors of proteins reflect their degree of expression.

### Gene ontology: molecular function, biological process, and cellular localization of EMX1 differential proteome

Differentially expressed proteins were GO-classified based on the PANTHER (Protein ANalysis THrough Evolutionary Relationships) system (http://www.pantherdb.org; Mi et al., 2016). PANTHER software classifies genes and proteins by their functions, using published scientific experimental evidence and evolutionary relationships abstracted by curators with the goal of predicting function even in the absence of direct experimental evidence (Mi et al., 2010). Proteins are classified into families and subfamilies of shared function, which are then categorized using a highly gene controlled vocabulary (Gene Ontology terms) by biological process, molecular function and cellular localization (for the detailed methodology of Gene Ontology analysis, please refer to Data Sheet 1 in the Supplementary Material, a detailed description of these data is presented in the Supplemental Figures 1–3).

### BrdU and proliferation assay

NSCs were plated onto poly-L-ornithine coated plates at 10^5^/well (24-well plate). Twenty-four hours post plating, recombinant mouse VEGF-A (100 ng/ml, R&D system) was added to culture medium and BrdU was added into culture 24 h later at a concentration of 10 μM prior to fixation with 4% PFA. SU1498 was added into culture at 5 or 10 μM prior to the addition of VEGF. The number of BrdU positive cells was quantified only in live cells.

### Statistical analyses

Data were expressed as mean ± s.e.m. Statistical tests were carried out with one-way or two-way analyses of variance (ANOVAs) followed by *post-hoc* paired comparisons using the Fisher's PLSD test when appropriate (Statview 5.0.1, SAS Institute Inc., Cary, NC). Values of *p* < 0.05 were considered as significant.

## Results

### *Emx1* gene deletion reduces embryonic neural stem cell population

**Figure 1:**
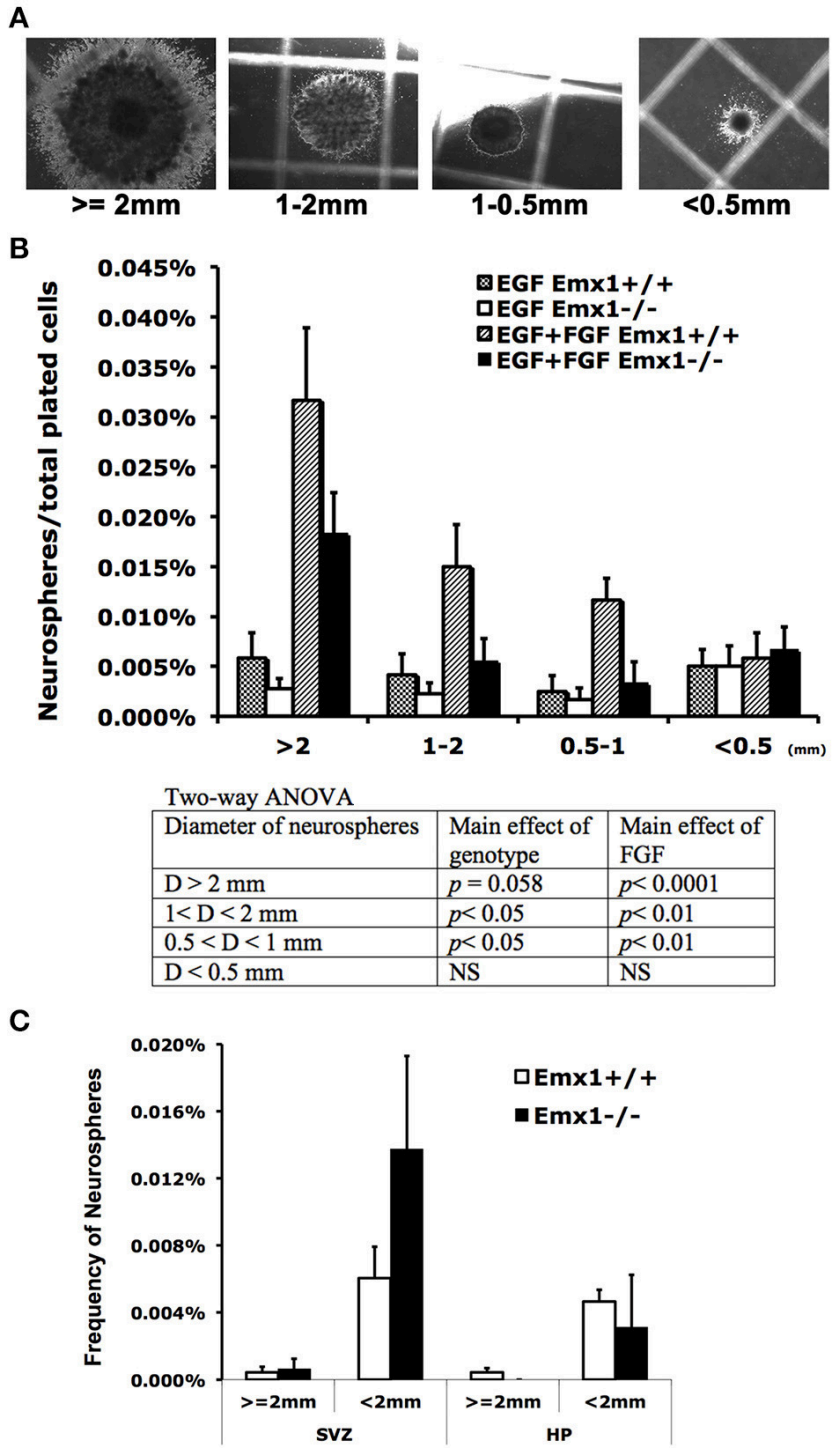
**The effect of homeobox gene *Emx1* deletion on the formation of neurospheres in the embryonic and adult forebrains**. Neurospheres grown on the semi-solid collagen plates were categorized into four groups according to size as indicated **(A)**. Neurospheres that have a diameter of more than 2 mm are regarded as true neural stem cells and others are progenitor cells (Louis and Reynolds, 2010). **(B)** Neural colony forming cell assay demonstrates that *Emx1*^−/−^ embryonic forebrains formed significantly less neurospheres with sizes between 0.5 and 1 mm in diameter. The presence of FGF-2 (20 ng/ml) in the growth medium enhanced the genotype disparity in neurosphere formation. The *p*-values of the main effects of genotype and FGF following 2-way ANOVA are summarized as shown. Sample sizes for WT EGF, KO EGF, WT EGF+FGF, KO EGF+FGF are 4, 6, 4, 6, respectively. **(C)** The frequency of neurospheres per plated cells from the dissected subventricular zone (SVZ) or hippocampus (HP) in the adult forebrains cultured in the presence of EGF+FGF. There was no significant difference in the frequency of neurospheres from adult brain tissue between two genotypes. Unlike the embryonic brains, the adult brains have very few true neural stem cells (≥ 2 mm in diameter). *N* = 4–6 brain samples/group for embryonic and 4–9 brain samples/group for adult neurosphere assay.

*Emx1* deletion is associated with the reduction of volume and immature neurons in the dentate gyrus (DG) of adult mice (Hong et al., 2007). To determine whether this abnormality originated from the early stage of brain development, neurosphere assay was performed with embryonic or adult brains from *Emx1* knockout (KO) and wild type (WT) mice. Although embryonic brains from both genotypes formed neurospheres, the number of neurospheres or neuroprogenitor cells (0.5 mm < diameter < 1 mm) in E14 *Emx1*^−/−^ forebrains was significantly less than those of *Emx1*^+/+^ brains. In addition, the size of neurospheres was smaller, indicating that *Emx1*^−/−^ embryonic NSCs had a limited proliferative potential (Figures 1A,B). The frequency of neurospheres formed during 21 days of culture also differed depending on the presence of growth factors. There was a significant effect of FGF on the frequency of neurospheres greater than 0.5 mm in diameter in both genotypes (Figure 1). In contrast to the embryonic brains, the adult brains harbor very few true NSCs in either genotype. There was also no significant difference in the overall frequency of NSCs within the progenitor regions of subventricular zone or the hippocampus between genotypes (Figure 1C).

### *Emx1* gene deletion impairs the self-renewal capacity of the embryonic neural stem cells

**Figure 2:**
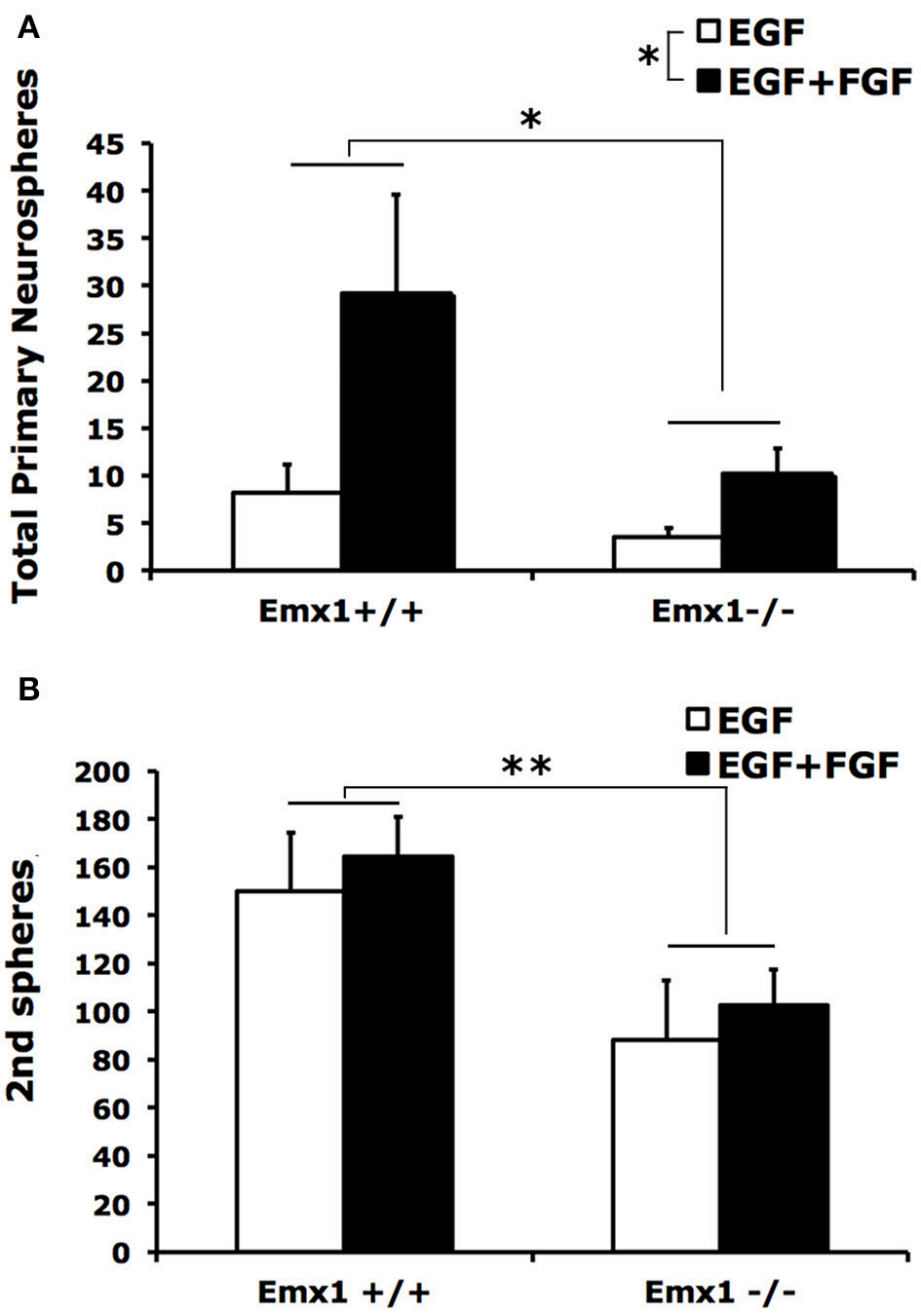
**The self-renewal capacity is diminished in embryonic *Emx1*^−/−^ NSCs**. **(A)** There is a significant effect of genotype (*p* < 0.05) and FGF (*p* < 0.05) on the total number of primary neurospheres of all sizes formed from dissociated E14 forebrains. **(B)**, Primary neurospheres from *Emx1* WT and *Emx1* KO mice were mechanically dissociated into single cells, counted and plated at clonal density. The number of secondary neurospheres generated from triplicate sets of 400 viable cells derived from the primary neurospheres from each forebrain sample was quantified after 10 days. Both primary neurospheres from forebrains of *Emx1* KO and *Emx1* WT embryos were able to self-renew. However, the ability of dissociated primary neuospheres from *Emx1* KO to form secondary neurospheres was reduced compared to those from *Emx1* WT (genotype effect: *p* < 0.01). There was no significant effect of FGF on the formation of secondary neurospheres. *N* = 6/group. ^*^p < 0.05; ^**^p < 0.01.

Neurospheres contain multipotent NSCs with heterogeneous capacity in clonal expansion. To determine whether the observed difference in the number or size of neurospheres is attributed to differences in self-renewal capacity between genotypes, we quantified the number of secondary neurospheres generated by single primary neurospheres following 10 days of culture in a self-renewal assay. We found that primary neurospheres from forebrains of both *Emx1* WT and KO embryos had the ability of self-renewal (Figure 2A), but differed in the extent of producing secondary neurospheres (Figure 2B). The numbers of primary and secondary neurospheres from *Emx1* KO brains were significantly reduced to ~30 and 50% of those from the *Emx1* WT ones, respectively (Figure 2).

### *Emx1* gene deletion does not affect neuronal or glial differentiation of NSCs *in vitro*

**Figure 3:**
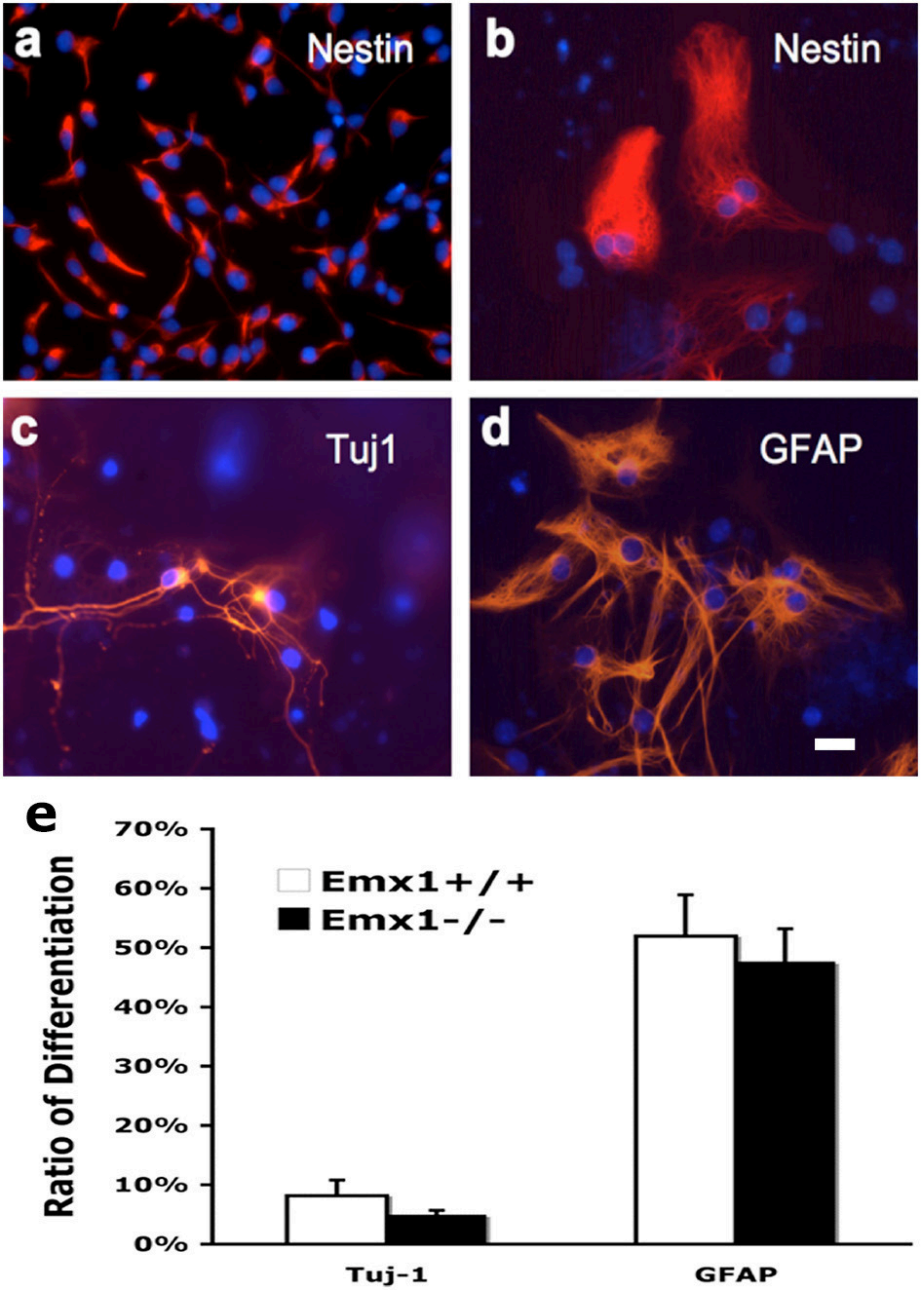
**Neuronal and glial differentiation from embryonic NSCs is not affected by *Emx1* gene deletion**. NSCs differentiate in response to growth medium containing differentiation supplement after 7–14 days. Representative photomicrographs showing undifferentiated neural stem cells grown as monolayer (Nestin; **A**, soon after seeding; **B**, 7 days after seeding), differentiated neurons (**C**, TuJ1) and glia (**D**, GFAP) in red and nuclei in blue (Hoechst). Bar graph indicated the quantitative results for differentiation. The extent of neuronal vs. glial differentiation was determined by counting 200 cells after staining with antibodies against TuJ1 and GFAP. No differences in the differentiation ratios of neurons and astrocytes between *Emx1*^+/+^ and *Emx1*^−/−^ group **(E)**. Scale bar, 25 μm. *N* = 4 NSC samples/group, and each NSC sample was plated in 3–4 wells.

To compare the extent of multipotency of NSCs derived from *Emx1* WT and KO embryonic forebrains, cells from neurospheres of both genotypes were exposed to differentiation medium for 10 days. The majority of dissociated NSCs cultured as monolayer cells displayed immunoreactivity for nestin, a marker of neuroprogenitor cells, prior to the induction of differentiation (Figures 3A–D). Multipotency was demonstrated by differentiation into TuJ1-positive neurons, and GFAP-positive astrocytes. However, no difference was found in the differentiation ratio of neurons or astrocytes between *Emx1* WT and *Emx1* KO NSCs (Figure 3E).

### *Emx1* gene deletion reduces the chemotactic response of NSCs toward serum or VEGF

**Figure 4:**
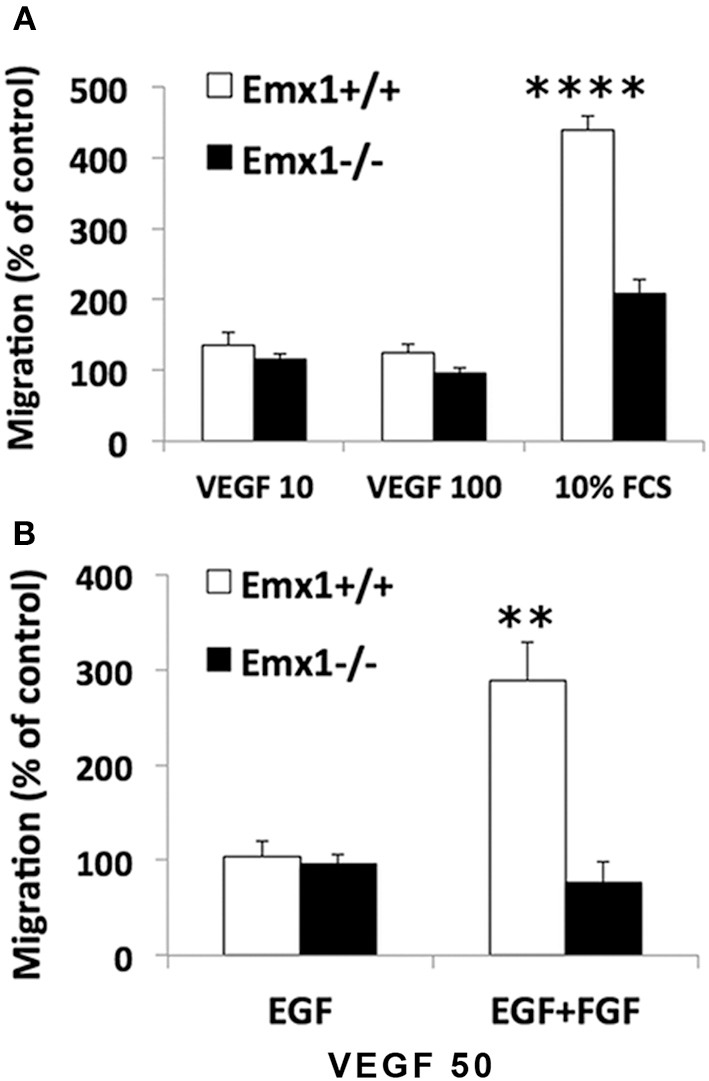
**Chemotactic response to serum or VEGF is reduced in *Emx1*^−/−^ NSCs. (A)** *Emx1* KO NSCs exhibited a reduced chemotactic response to serum compared to *Emx1* WT NSCs. The migratory response of E14 NSCs to fetal calf serum (FCS) and recombinant human VEGF165 were tested in a modified 96-well Boyden chamber assay when cells were cultured in medium containing EGF. Ten percent FCS induced a 4.4-fold (439 ± 19%) and 2.1-fold (209 ± 19%) increase in migratory response in *Emx1* WT and *Emx1* KO NSCs, respectively. To the contrary, VEGF, either at 10 or 100 ng/ml, stimulated very little migration of NSCs from either genotype. **(B)** FGF is necessary for the chemotactic response of *Emx1* WT NSCs to VEGF. NSCs were cultured in medium containing either EGF alone or EGF+FGF. Chemotaxic response toward 50 ng/ml VEGF was detected in NSCs from *Emx1* WT, but not from *Emx1* KO, cultured in the presence of EGF+FGF. Values shown are mean ± SEM and are expressed as percentage of the unstimulated basal migratory rate (control = 100%). ^**^p < 0.01; ^****^p < 0.001. *N* = 24–32/group.

Neuroblasts migrate in close association with blood vessels and long-distance migration of neuroblasts was found in peri-infarct tissue in human stroke, implicating a role for VEGF or other angiogenic factors in neurogenesis and neuroblast migration. To quantify the chemotactic response of NSCs, a modified Boyden Chamber assay was used in the presence of FCS or chemokine VEGF_165_ as chemoattractants. In response to 10% FCS, *Emx1* WT NSCs exhibited a 2.1-fold increase in chemotaxis activity relative to *Emx1* KO NSCs. When cultured in the presence of EGF, NSCs of either *Emx1* genotype failed to migrate in response to VEGF_165_ at 10 or 100 ng/ml (Figure 4A). In contrast, when cultured in the presence of EGF and FGF, VEGF_165_ at 50 ng/ml was able to induce a robust migration of *Emx1* WT NSCs compared to *Emx1* KO NSCs (Figure 4B). Out findings indicate that *Emx1* gene deletion negatively impacted the chemotactic response of NSCs toward serum or VEGF, and the migration *Emx1* WT NSCs induced by VEGF requires the presence of FGF in addition to EGF (Zhang et al., 2014).

### Proteomic profiling reveals differences in the protein expression pattern between *Emx1* WT and KO NSCs

**Figure 5:**
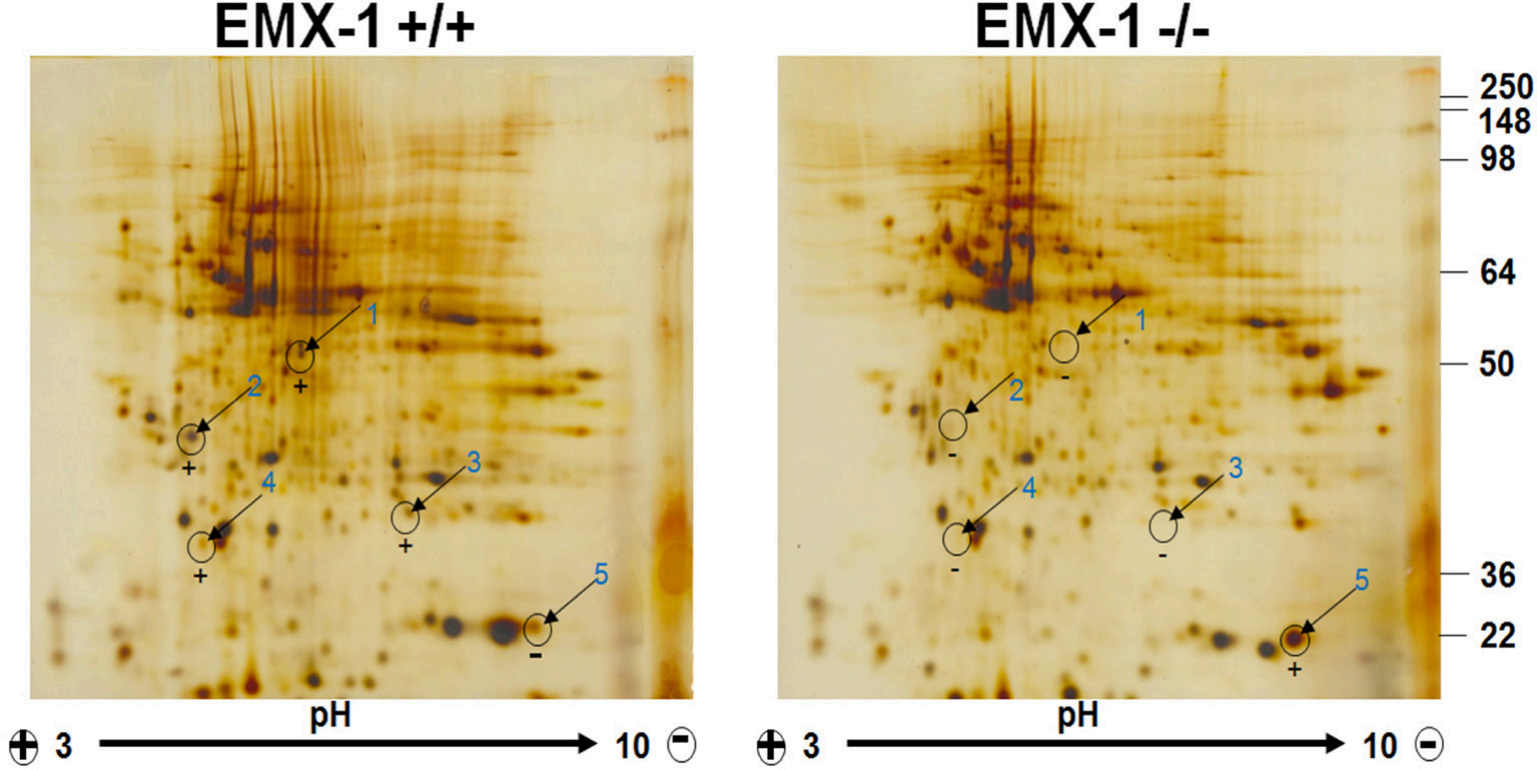
**Two-Dimensional Gel Electrophoresis proteomic profiling of *Emx1*^+/+^ and *Emx1*^−/−^ NSCs**. Differential comparative proteome profile from *Emx1*^+/+^ and *Emx*1^−/−^ NSCs lysates was compared and visualized by silver staining. Comparative Coomassie stain performed on gels was used for spot cutting. Five most apparent spots showing differential expression level between genotypes were labeled and excised for protein identification by tandem MS/MS analysis. Spot #5 was identified as Cofilin through Swiss-Prot Protein Knowledgebase.

Using tandem mass spectrometry, we analyzed a number of most distinctly different spots on the 2-D PAGE comparing the patterns of protein expression from the lysates of *Emx1* WT and KO embryonic NSCs (Figure 5). Our neuroproteomics analysis detected 38 differentially expressed proteins between *Emx1*^−/−^ and *Emx*1^+/+^ (see Table 1 for protein Ids and their respective biological functions). Detailed characteristics of each individual protein with its corresponding mass spectrometry data are included in Supplemental Tables 1, 2 and Data Sheet 1 in the Supplementary Material.

**Table 1:** **Differentially altered proteins and their functional ontology between *Emx1*^+/+^ and *Emx1*^−/−^ NSCs as identified from spots on the 2-D gel**.

**Spot No**.	**Protein name**	**Functional ontology**
1	Phosphoglycerate kinase	-A protein kinase involved in glycolysis.
		-Loss of function mutation (deficiency) causes chronic hemolysis with or without mental retardation (Beutler, 2007).
1	Alpha-enolase	-A protein involved in glycolysis pathway.
		-This protein acts as an autoantigen in Hashimoto encephalopathy and used as a marker for hypoxic brain injury (heterodimeric form) after cardiac arrest (Ochi et al., 2002; Rech et al., 2006).
1	Ornithine aminotransferase, mitochondrial	-An enzyme that catalyzes the formation of the non-essential amino acid proline from ornithine.
		-The inheritance of a mutated form causes chorioretinal atrophy that can progressively lead to blindness, but patients generally have normal intelligence.
1	Tubulin beta-2A chain	-Tubulin is the principle component of microtubules that involve in many processes. Mutation in this gene causes brain malformation that affects neuronal migration and axonal guidance (Cushion et al., 2014).
1	ADP-ribose pyrophosphatase, mitochondrial	-An enzyme with a catalytic activity responsible for the formation AMP and ribose 5'-phosphate from ADP-ribose (ADPR).
1	Calponin-3	-It regulates actin filament during neuronal remodeling (Rami et al., 2006). It also regulates dendritic spine plasticity in adult hippocampal neurons (Ferhat et al., 2003), and it was found to be upregulated in epileptic patients (Han et al., 2012).
1	Nucleoporin Nup37	-It is a component of the nuclear pore complex (NPC) that is required for its proper assembly and function. It is also essential for the attachments of microtubules to kinetochores.
1	Pyruvate kinase isozymes M1/M2	-A glycolytic enzyme that catalyzes the final step of glycolysis (rate limiting step) and generates ATP by transferring a phosphate group from PEP (phosphoenolpyruvate) to ADP (adenosine diphosphate).
2	Creatine kinase B-type	-A protein kinase responsible for the reversible reaction of transferring phosphate group between ATP and various phosphogens (e.g., creatine phosphate).
2	14-3-3 protein epsilon	-An adapter protein that has the ability to bind to a large number of proteins that are implicated in different signaling pathways.
2	14-3-3 protein zeta/delta	-An adapter protein that has the ability to bind to a large number of proteins. The binding occurs by the recognition of a phosphoserine or phosphothreonine. It plays an important role in cell survival and apoptosis.
2	14-3-3 protein theta	-An adapter protein that has the ability to bind to a large number of proteins that are implicated in different signaling pathways.
3	ATP synthase subunit alpha, mitochondrial	-A mitochondrial ATP synthase, a membrane bound protein that drives the formation of ATP by harnessing the energy from protons gradient.
3	ATP synthase subunit O, mitochondrial	-A mitochondrial ATP synthase, a membrane bound protein that drives the formation of ATP by harnessing the energy from protons gradient.
3	ADP-ribosylation factor-like protein 3	-A small GTP-binding protein that can exist in two states: GTP-binding (active) and GDP-binding (inactive). It is required for normal cytokinesis and cilia signaling.
3	Heterogeneous nuclear ribonucleoprotein A1-	It plays a role in pre-mRNA processing and is involved in the formation of hnRNP (heterogeneous nuclear ribonucleoproteins) and transport of poly(A) mRNA from the nucleus to the cytoplasm.
3	Peroxiredoxin-1	-An antioxidant protein that participates in the elimination of peroxides from the cell. Additionally, it might play a role in the signaling cascades of growth factors and tumor necrosis factor-alpha.
3	Tetratricopeptide repeat protein 25	-No known function.
4	Peroxiredoxin-2	-An antioxidant protein that participates in the elimination of peroxides from the cell. Additionally, it might play a role in the signaling cascades of growth factors and tumor necrosis factor-alpha.
4	Heterogeneous nuclear ribonucleoprotein K	-A pre-mRNA binding protein that has an affinity toward poly C (cytidine) sequence. It plays a role pre-mRNA metabolism and processing and participates in p53/TP53 response to DNA damage, acting at the level of both transcription activation and repression.
4	Tubulin beta-2C chain	-Tubulin is the major constituent of microtubules. It binds two moles of GTP, one at an exchangeable site on the beta chain and the other at a non-exchangeable site on the alpha chain.
4	Phosphatidylethanolamine-binding protein 1	-It binds to different molecules including ATP, opioids and phosphatidylethanolamine.
4	Proteasome subunit beta type-6	-The proteasome is a protein complex that acts as a proteinase and has the ability to cleave peptides with very broad specificity that include peptide bonds with Arg, Phe, Tyr, Leu, and Glu adjacent to the leaving group at neutral or slightly basic pH. It is ATP-dependent.
4	NudC domain-containing protein 2	-This protein may regulate the LIS1/dynein pathway by stabilizing LIS1 with Hsp90 chaperone.
4	Ras-related protein Rap-2a	-A small GTP-binding protein. Rap-2a has a role in different signaling cascades that regulate cytoskeleton, cell migration, cell adhesion and cell spreading. It is a part of a signaling complex that regulates neuronal dendrite extension and arborization during development.
4	Tubulin alpha-1A chain	-Tubulin is the major constituent of microtubules. It binds two moles of GTP, one at an exchangeable site on the beta chain and the other at a non-exchangeable site on the alpha chain.
4	F-actin-capping protein subunit alpha-1	-F-actin-capping proteins bind to the fast growing ends of actin filaments (barbed end) independently of calcium, thus blocking the exchange of subunits at these ends. In contrast to other capping proteins, these proteins do not sever actin filaments.
4	Ubiquitin-conjugating enzyme E2 variant 1	-It lacks ubiquitin ligase activity on its own. It also has a crucial role in the control of progress through the cell cycle, differentiation and in the error-free DNA repair pathway and contributes to the survival of cells after DNA damage.
5	Glyceraldehyde-3-phosphate dehydrogenase	-A glycolytic enzyme that catalyzes the first step of the pathway.
5	ADP-ribosylation factor 1	-A GTP-binding protein that has an ADP-ribosyltransferase activity. It activates cholera toxin and plays a role in intracellular trafficking.
5	Destrin	-An actin-depolymerizing protein. It binds to actin monomers and breaks off actin filaments (F-actin) in a pH-independent manner.
5	Peroxiredoxin-5, mitochondrial	-An antioxidant protein that reduces hydrogen peroxide and alkyl hydroperoxides in the cell and is involved in intracellular redox signaling.
5	N(G),N(G)-dimethylarginine dimethylaminohydrolase 1-	-It hydrolyzes N(G),N(G)-dimethyl-L-arginine (ADMA) and N(G)-monomethyl-L-arginine (MMA) with a role in nitric oxide generation through the inhibition of NOS.
5	Cofilin-1	-It regulates actin cytoskeleton dynamics through binding to F-actin in a PH dependent manner, playing a major role in the regulation of cell morphology and cytoskeletal organization. It is also important for the progression to mitosis and cytokinesis.
5	Cofilin-2	-It controls both actin polymerization and depolymerization in a reversible and pH-sensitive manner. It has the ability to bind G- and F-actin and is an essential component of the intranuclear as well as cytoplasmic actin rods.
5	Peptidyl-prolyl cis-trans isomerase A	-A PPIases catalyzes the cis-trans isomerization; it increases the folding rate of different proteins and regulates many biological Processes including cellular signaling, apoptosis and inflammation.
5	Peptidyl-prolyl cis-trans isomerase H	-A PPIases catalyzes the cis-trans isomerization of proline imidic peptide bonds in oligopeptides. It has been shown that PPIH participates in pre-mRNA splicing and it might act as a chaperone.
5	Nucleoside diphosphate kinase A	-An enzyme that catalyzes the transfer of phosphate from ATP to the NDP beta phosphate via a ping-pong mechanism. It plays a role in cell proliferation, differentiation, development, signal transduction and gene expression. It is necessary for neural development including neural patterning and cell fate determination.

We utilized Uniprot database to extrcat protein functions (http://www.uniprot.org/uniprot/).

### Global interaction map and protein cluster analysis of the Emx1 altered proteome

Based on the above neuroproteomics findings, we utilized a neurosystem biology platform for protein network and pathway analysis relevant to the altered proteins in the *Emx1*^+/+^ vs. *Emx1*^−/−^ NSCs. For this purpose, we utilized two bioinformatics analysis approaches: a global protein interaction and unsupervised pathway assessment on one side and a cluster targeted approach for selective implicated functional biological pathways on the other hand.

**Figure 6:**
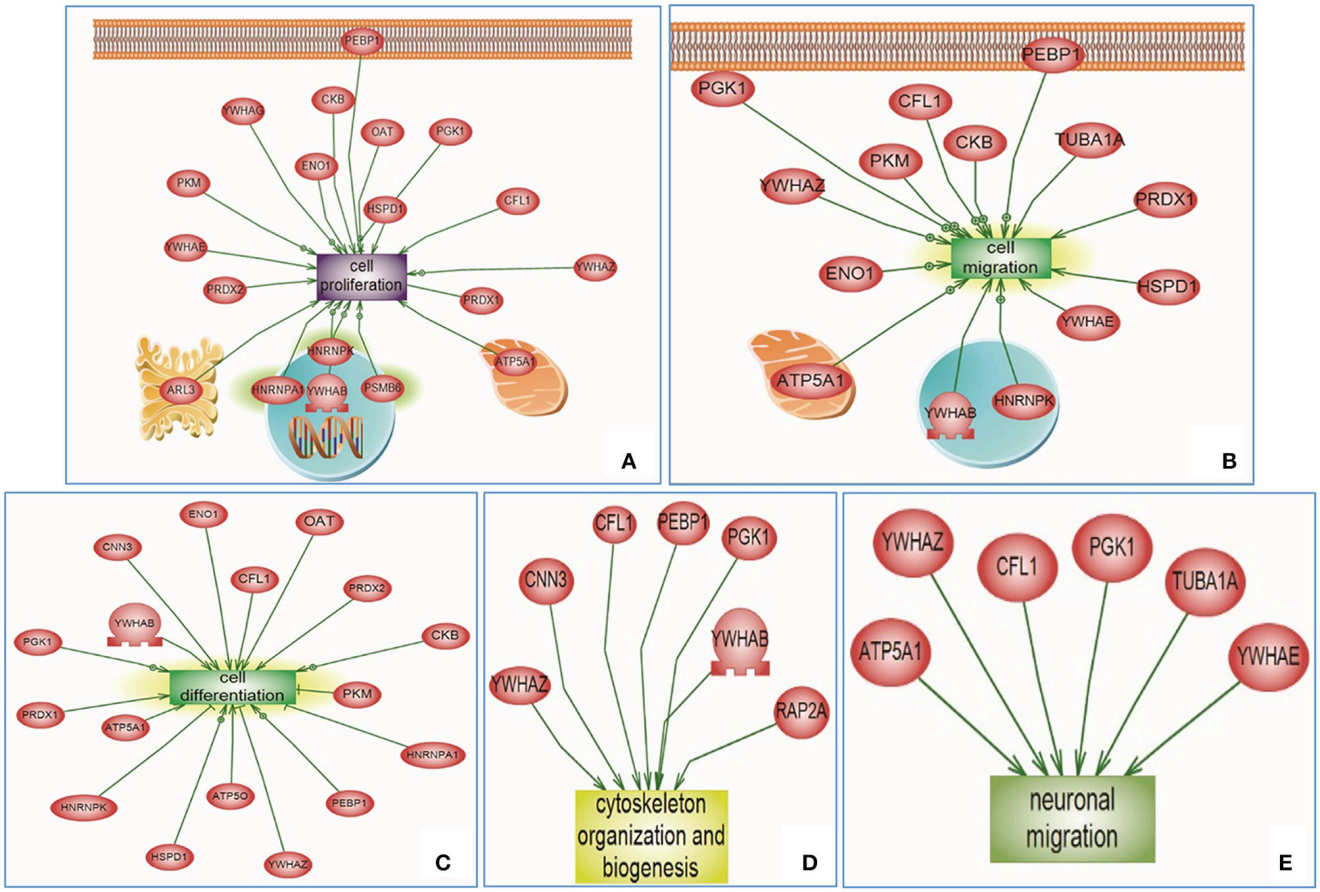
**Altered Emx1-related protein cluster analysis extracted from the Sub Network Enrichment Analysis (SNEA) Algorithm**. Subnetwork Enrichment Pathway Analyses and statistical testing for the entire altered Emx1 proteome was analyzed using the SNEA algorithm to identify unique biological clusters of differential proteins. Several proteins were clustered under certain functional classes, such as cell proliferation, cell migration, cell differentiation, cytoskeletal organization, and neuronal migration (Clusters **A–E**). *In silico* cluster analysis validation including the protein Entity and biological process involved along with the interaction type and directionality and the PubMed reference utilized to derive these interaction type are presented in Supplemental Tables 3A–C, 4A–C, 5A–C, 6A–C and Data Sheet 1 in the Supplementary Material. The shape of a given protein is indicative of its functional class as shown in the legend (rectangular shapes indicate the biological process) & (the elliptical shape indicates the proteins). Also included in the legend is the directionality relation of the protein with the corresponding biological process (arrow head).

For the targeted cluster analysis, selected Emx1-implicated pathways were assessed against Emx1-identified differential proteomic data. Using the SNEA algorithm, targeted enriched pathways revealed that several altered proteins were associated with *Emx1* differential proteome-implicated neural pathways including: cell proliferation, cell migration, cell differentiation, cytoskeletal actin organization, and neuronal migration as illustrated in Figures 6A–E. For detailed cluster analysis *in silico* validation including statistical analysis, protein entity, biological process along with the interaction type and the PubMed reference utilized see Supplemental Tables 3A–C, 4A–C, 5A–C, 6A–C and Data Sheet 1 in the Supplementary Material.

**Figure 7:**
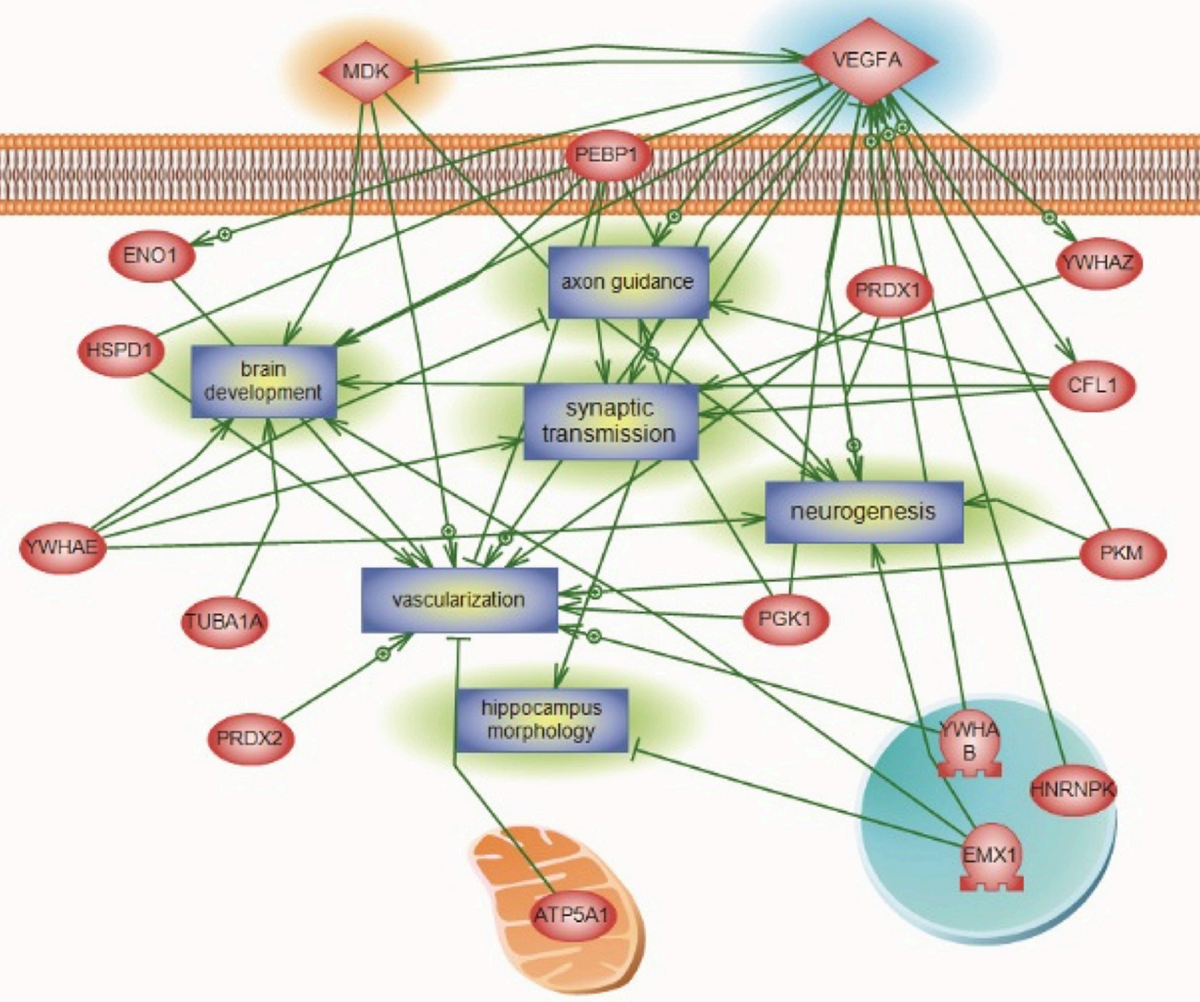
**Global Interaction Map and differential regulatory pathways of the Emx altered proteome**. Altered Emx1-identified proteins are highlighted in light pink. Seventeen out of the 38 proteins had direct regulatory relationships, including binding, post-translational modifications, and transcriptional regulation. Differential pathways were generated using the “direct interaction” algorithm to map interaction and relationships. Based on Biological Process analysis and molecular functions, these proteins are implicated in brain development, axonal guidance, synaptic transmission, neurogenesis along with the hippocampal morphology biological process (highlighted as rectangles). VEGF-A was identified as the upstream regulator of the represented processes, intertwined with Cofilin1 and Emx1. *In silico* validation including the protein Entity and biological process involved along with the interaction type and directionality and the PubMed reference utilized to derive these interaction type are presented in Supplemental Tables 7A–C and Data Sheet 1 in the Supplementary Material.

For the global interaction and unsupervised pathway assessment, differential pathways were generated using the “direct interaction” algorithm to map the relationship among the identified proteins. We found that among the 38 altered proteins, 17 had direct regulatory function, including binding, post-translational modifications and transcriptional regulation. Unsupervised pathway assessment, showed that these proteins are implicated in several biological pathways involving brain development, axonal guidance, synaptic transmission, neurogenesis along with the hippocampal morphology biological process (highlighted in blue rectangles). Of interest, these altered proteins showed a centrality relation with VEGF protein being an upstream regulator for several of the Emx1-identified proteome involving Cofilin1, enolase, phosphatidyl-ethanolamine-binding protein, and neurite growth-promoting factor 2 (Figure 7). Statistical significance of the interaction was performed *in silico* for the validation process. A detailed depiction of these data is presented in the (Supplemental Tables 7A–C and Data Sheet 1 in the Supplementary Material). These include the protein entity and biological process involved along with the interaction type and directionality and the PubMed reference utilized to derive these interaction type. Due to the well-recognized central role of Cofilin1 and its diverse interaction with several of the identified biological pathways, we performed a functional validation analysis to determine the expression of Cofilin1/p-Cofilin1in the NSCs of both genotypes in response to VEGF signaling.

### *Emx1* KO NSCs display reduced level of p-Cofilin1 expression

**Figure 8:**
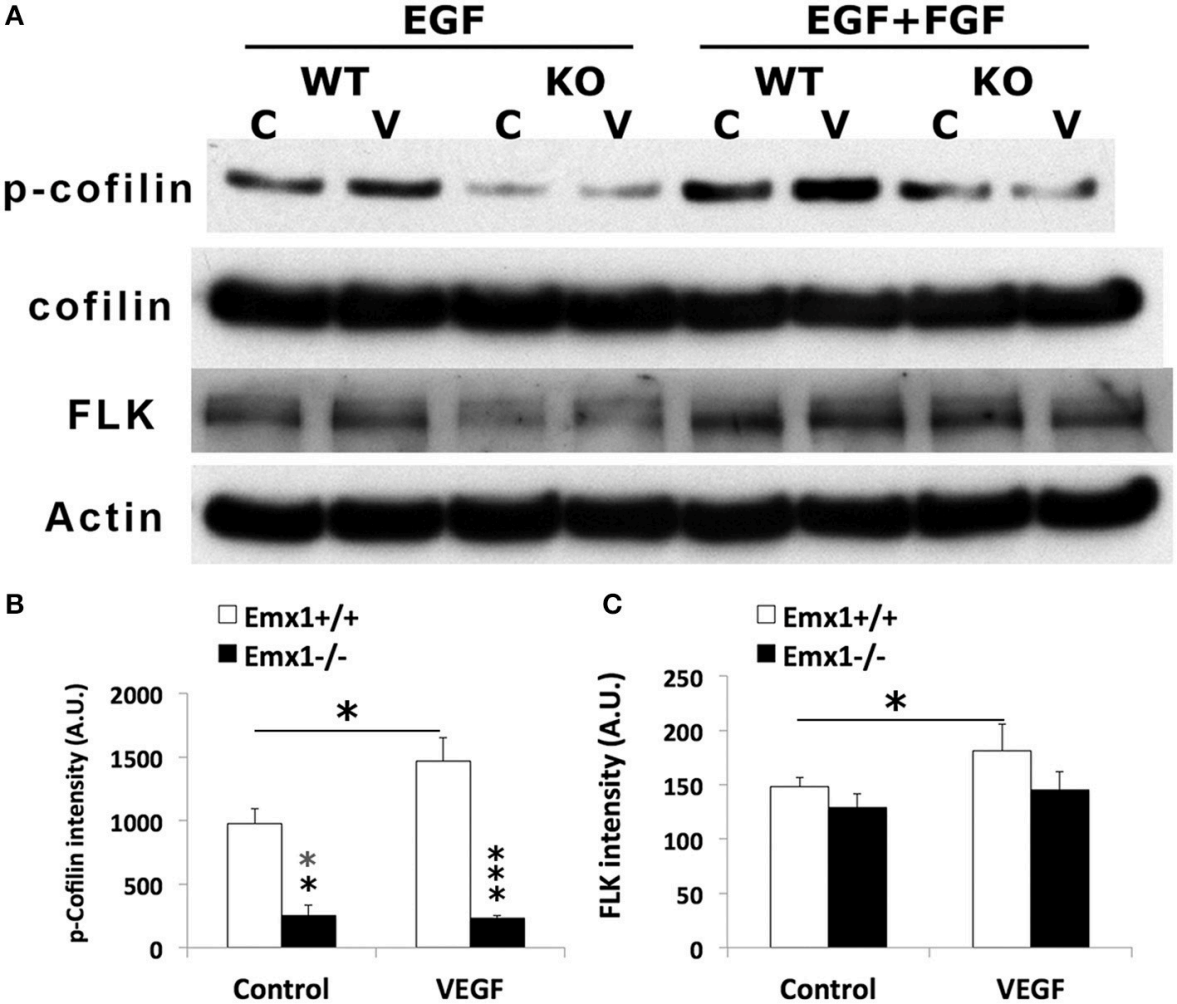
**VEGF induces Cofilin1 phosphorylation in *Emx1*^+/+^ NSCs. (A)** Western blot analysis of NSC lysates for p-Cofilin1, Cofilin1, VEGF receptor 2 FLK and actin from both *Emx1* genotypes treated with VEGF (V) (50 ng/ml) or medium (C) for 1 h in the presence EGF, or EGF+FGF as indicated. VEGF increased the level of p-Cofilin1in the *Emx1*^+/+^ NSCs cultured in EGF or EGF+FGF, compared to those treated with medium (^*^p < 0.05). In contrast, *Emx1*^−/−^ NSCs expressed significantly less amount of p-Cofilin1 whether in the absence (^**^p < 0.01) or presence of VEGF (^***^p < 0.005), compared to that of the *Emx1*^+/+^ NSCs **(B)**. VEGF increased FLK expression only in the *Emx1*^+/+^ NSCs (**C**, ^*^p < 0.05). *N* = 3/group.

To further determine whether Cofilin1 plays a role in VEGF signaling, lysates of NSCs from both genotypes stimulated with VEGF or media were analyzed by western blotting for the expression of p-Cofilin1, total Cofilin1, and VEGF RECEPTOR 2 FLK. We found that *Emx1* KO NSCs had significantly reduced p-Cofilin1 compared to those from *Emx1* WT, when cultured in medium containing EGF only or EGF+FGF (Figures 8A,B). VEGF further increased the level of p-Cofilin only in the WT NSCs (Figures 8A,B). VEGF also modestly induced the expression of FLK only in the WT NSCs (Figures 8A,C). Our results suggest that *Emx1* gene deletion reduces phosphorylated Cofilin1, likely contributes to the difference in neurogenesis and chemotactic response to VEGF.

Because VEGF receptors are expressed in NSCs (Maurer et al., 2003) and VEGF stimulates the expansion of NSCs *in vitro* (Schänzer et al., 2004), we determine the effect of VEGF on the proliferation and survival of NSCs. We found that VEGF-A (100 ng/ml) significantly increased the percentage of BrdU immunoreactive cells already after 4 h (*p* < 0.05) of treatment. The effect was even more pronounced after 17 h (*p* < 0.001; Supplemental Figure 1A and Data Sheet 1 in the Supplementary Material). In addition, VEGF-induced NSC expansion was dose dependently reduced by the specific VEGF RECEPTOR 2 antagonist SU1498 (Supplemental Figure 1B and Data Sheet 1 in the Supplementary Material), suggesting that VEGF increased the number of NSCs possibly by stimulating proliferation or enhancing their survival.

## Discussion

Existing literature suggests that the *Emx1* transcription factor is not among the most critical players in mammalian brain development since the *Emx1*-single mutant was not only viable but also without any severe CNS phenotype in contrast to the mutations caused by a number of other genes (Patarnello et al., 1997; Guo et al., 2000; Bishop et al., 2002, 2003; Cao and Li, 2002; Muzio and Mallamaci, 2003; Shinozaki et al., 2004; Tamamaki, 2005; von Frowein et al., 2006; Piper et al., 2007; Cocas et al., 2009; Sen et al., 2013). The role of *Emx1* during brain development was first implicated in axon guidance, regulating midline crossing of an axonal subpopulation of the corpus callosum derived from the anterior cingulate cortex (Lim et al., 2015). Apart from an acallosal phenotype, the adult *Emx1* null mice also exhibited smaller dentate gyri and reduced neurogenesis (Hong et al., 2007). However, it is unclear whether the reduced dentate gyrus is attributed to defective signaling in neurogenesis. By comparing the frequency of neurosphere formation between the WT and KO *Emx1* forebrains, we found that the embryonic but not adult *Emx1* KO brains had significantly fewer NSCs and neuroprogenitor cells, suggesting that *Emx1* was involved in neurogenesis at least during early brain development. The deletion of the *Emx1* gene also negatively impacted the self-renewal capacity of isolated NSCs, diminishing their chemotactic response to FCS or VEGF, but it did not affect neuronal or glial differentiation. Comparative proteomics revealed that the altered proteins from *Emx1* KO NSCs participated in many aspects of brain development, including axonal guidance and neurogenesis, through interaction with VEGF. Cofilin1 exists in balance with its phosphorylated form in the VEGF signaling pathway. *Emx1* KO NSCs appeared to have significantly reduced level of p-Cofilin1 compared to *Emx1* WT NSCs. Additionally, VEGF further increased p-Cofilin1 only in the latter. To the best of our knowledge, this is the first study demonstrating the role of *Emx1* in neurogenesis and the effect of *Emx1* gene deletion on Cofilin1 phosphorylation utilizing proteomics platform.

Not surprisingly, the neurosphere assay results indicate that the adult brains harbor significantly fewer neuroprogenitor cells compared to embryonic brains, regardless of *Emx1* genotype. It appears that only the embryonic brains contain a significant amount of true NSCs that can be propagated in many generations *in vitro*, while the adult brains have a number of neurospheres that are >2 mm in diameter. The presence of FGF increases the frequency of neurospheres >0.5 mm in diameter from embryonic brains of both genotypes. A previous report suggests that stem/precursor cells in the embryonic cortex can be very diverse in regards to their signaling property. Linear progression of the precursor cells to an EGF-responsive state can be promoted by FGF among other factors (Lillien, 2014), which is consistent with our observation. There also exists a rostral-caudal gradient of *Emx1*-lineage precursors, with more cells rostrally. We did not determine the rostral-caudal relationship in the abundance of precursor cells in the embryonic brains due to the small size; however, data from the adult neurosphere assay seem to agree with this finding, as the SVZ tended to produce more neurospheres than the hippocampus. Data from the self-renewal assay suggest that *Emx1* KO precursors likely do not respond as well to EGF or FGF signaling to form primary or secondary neurospheres. Apart from its key role in neurogenesis and NSC self-renewal, *Emx1* also contributes to other aspects of brain development such as cortical patterning (Stocker and O'Leary, 2016) and regional fate determination (Chou et al., 2009) via the complex interplay between *Emx1* and other genes.

A proteomic search using 2-D gel electrophoresis coupled with tandem mass spectrometry revealed the differential expression of at least 38 proteins between WT and KO NSCs. These protein hits were assessed via a targeted approach to study their relationship in already established pathways implicating *Emx1's* role in neurogenesis, and the details regarding their molecular function, biological process and cellular locationization are shown in Supplemental Figures 2–4. Among the proteins identified, OAT, Cofilin1, 14-3-3 beta protein, Creatine Kinase B-type Peroxiredoxin 2, Peroxiredoxin 1, and HNRNPA1 were implicated in the cell differentiation process (Figure 6, Supplemental Tables 3A–C and Data Sheet 1 in the Supplementary Material). This is in agreement with what has been identified in a similar proteomics study performed on neural differentiation of human embryonic stem cells utilizing 2D-DIGE at three different stages early neural differentiation, neural ectoderm and mature neurons. Ten members of the peroxiredoxin (PRDX) family were upregulated in the different stages of differentiation, thus highlighting a link between neural differentiation and the redox process (Hoffrogge et al., 2007; Fathi et al., 2014). Indeed, the work of Novitch and Butler highlighted the role of Prdx1 along with the GDE2 protein in mediating a thiol-redox reaction involved in the process neural regeneration process (Novitch and Butler, 2009). Other altered pathways identified included cellular migration and proliferation involving a number of proteins including Cofilin1, YWHAE, ATP5A1, TUBA1A, PRDX1, ADP-ribosylation factor-like 3, and ENO1 among others (Figure 6, Supplemental Tables 4A–C, 5A–C and Data Sheet 1 in the Supplementary Material). The enolase protein has been shown to promote cell migration in the context of cancer (Yu et al., 2010; Hsiao et al., 2013). Similarly, the brain specific creatine kinase protein involved in cellular energetics, has been correlated with loss of neural cell circuits function upon downregulation; however, its contributes a part to local cytoskeletal dynamics promoting cell migration via lamellipodia formation (Kuiper et al., 2009). The role of ADP-ribosylation factor-like 3 has been recently studied as an indirect mediator of cell proliferation and neurogenesis via binding to the signal transducer and activator of transcription 3 (Togi et al., 2016). Our data also corroborate with the finding of a pro-survival role of ADP- ribosylation factor-like-3 protein in neural progenitor cells (Zhou et al., 2013).

In addition, cytoskeleton organization and biogenesis of biologically related proteins were shown to be among the major protein families to be altered in the context of *Emx1* deletion. These include Cofilin1, calponin, destrin, and F-actin-capping protein subunit alpha-1 proteins (refer to Table 1, Figure 6, and Supplemental Tables 6A–C and Data Sheet 1 in the Supplementary Material). Also of interest, these groups of proteins involve actin-binding activity implicated in cell motility. Calponin protein expression has been suggested to be involved in neural cell contractility, proliferation, and migration (Represa et al., 1995; Ferhat et al., 1996; Ulmer et al., 2013). Similarly, destrin is also an actin-depolymerizing protein, which is closely related to Cofilin1 (Hatanaka et al., 1996) with a similar peptide sequence to the ADF (actin depolymerizing factor) protein (Hawkins et al., 1993), which is implicated in the regulation of neural crest cell migration (Vermillion et al., 2014). Along the same line, among the altered proteins Tubulin protein family including Tubulin α and Tubulin β (2a and 2c) also appears in high abundance (Supplemental Table 1 and Data Sheet 1 in the Supplementary Material). Tubulins play a key mechanical role in neuronal proliferation and migration during cortical development (Barkovich et al., 2012; Cushion et al., 2014). Both α- and β-Tubulin protein family bind and assemble microtubule polymers, forming the essential cytoskeletal structure during brain development with specific functions include facilitating neuronal communication by generating axonal fibers away from the cell body; as well as in assisting neuroblast proliferation and migration from the SVZ to developing neocortex (Desai and Mitchison, 1997; Dehmelt and Halpain, 2004, 2005; Guzik and Goldstein, 2004) The critical role of tubulin gene family is highlighted in the mutation studies identified in the neuronal specific α- and β-*tubulin* gene affecting the cortex, corpus callosum as well as basal ganglia (Kumar et al., 2010; Barkovich et al., 2012; Cushion et al., 2013, 2014; Amrom et al., 2014). In one proteomic study assessing temporal synaptic plasticity induced by GABA_A_ receptor blockade, Tubulin β-2A protein regulated dendritic spine morphology and synaptic plasticity in the hippocampus (Jaworski et al., 2009). Another elegant recent study indicated that there existed an interaction of the Collapsin Response Mediator Protein (CRMP) family, CRMP2 and CRMP-4 in particular, with tubulin and actin proteins *in vitro* that appeared to mediate growth cones development (Tan et al., 2015). This newly identified interaction is of high importance, considering the role of CRMP family in mediating cell migration, differentiation, neurite extension, and axonal regeneration in the developing nervous system, (Yoshimura et al., 2005; Ip et al., 2014). Our data suggest that Tubulin α and Tubulin β (2a and 2c) were upregulated in the *Emx1*^−/−^ NSCs, possibily attributable to be a feedback mechanism to counter balance the potential pathological phenotype of *Emx1* deletion, although further experiments are warranted to ascertain this hypothesis.

Of special interest to us were the down regulation of p-Cofilin1 protein in the *Emx1*^−/−^ NSCs and the diverse involvement of Cofilin1 in several of the targeted biological pathways as well as in the global interaction map (Figures 6, 7; respectively). Neuronal migration and neurite outgrowth are highly regulated processes that both depend on the assembly and disassembly of F-actin via the formation of appropriate connections within the neural network. *In vivo*, the length of the actin filament can be regulated by members of the Cofilin1 family of actin filament depolymerizing factors. Cofilin1, a downstream target of Rho kinase, is an actin-binding protein known to orchestrate actin filament turnover and regulate neuronal migration and neurite outgrowth (Carlier et al., 1997; Meberg, 2000; Meberg and Bamburg, 2000). Cofilin1 has also been shown to regulate cell cycle progression during cortical development (Bellenchi et al., 2007). The loss of Cofilin1 leads to the depletion of the ventricular zone neuronal progenitor pool and reduced cortical layer formation (Bellenchi et al., 2007). Aberrant expression of Cofilin1 might also underlie the defective development and cell migration in the telencephalon of *Emx2* mutants (Li H. et al., 2006).

VEGF also enhances axonal outgrowth by signaling through the Rho family of GTPases and their effector serine/threonine kinases, leading to downstream Cofilin1 phosphorylation (Jin et al., 2006; Zhang et al., 2011). However, we are uncertain whether the defect in *Emx1* KO NSC migration toward VEGF or other factors in the serum is related to the defective midline crossing of axons that resulted in the observed acallosal phenotype in the *Emx1* mutant. Interestingly, the disruption of interhemispheric connection in the naive *Emx1* KO mice did not significantly affect gross motor coordination nor learning and memory. However, it impaired fine motor function such as performance in the skill reaching task and reduced training induced neurogenesis (Hong et al., 2007). P-Cofilin1, on the other hand, regulates both cell proliferation and axon growth by promoting actin polymerization and cytoskeletal rearrangement. It is not surprising that training may lead to angiogenesis and the release of VEGF in addition to other growth factors that activate signals for neurogenesis in the WT, but less so in the KO mice due to an imbalance between Cofilin1 and p-Cofilin1 in the latter.

The VEGF receptor tyrosine kinase FLK-1, previously shown to be expressed in endothelial cells, is also expressed in neural progenitor cells of the mouse retina (Yang and Cepko, 1996) and embryonic forebrain NSCs (current study). Thus, VEGF signaling through FLK plays a dual role in neurogenesis and angiogenesis during development. In the adult brain, the coupling between angiogenic blood vessels and neuroblasts further supports a neurovascular niche in the post stroke microenvironment (Ohab and Carmichael, 2008), suggesting that ischemic brain injury induces signals recapitulating those during brain development. One of the most important and well studied growth factors that regulate post stroke neurogenesis and angiogenesis is VEGF. Both exogenous VEGF and stroke-induced endogenous VEGF promote neurogenesis (Sun et al., 2006), while the blockade of endogenous VEGF receptor downregulates neurogenesis (Bao et al., 1999). Despite the impaired VEGF signaling and reduced neurogenesis in the *Emx1* KO mice, ischemic stroke with distal occlusion of the middle cerebral artery did not induce a greater lesion size in the KO compared to the WT mice, neither did traumatic brain injury (TBI) (unpublished results). Interestingly, forelimb skill training induced neurogenesis in the uninjured WT mice, while it modestly induced neurogenesis in the KO mice after TBI (unpublished results). This suggests that brain injury and behavioral training elicit more growth promoting signals aside from VEGF that are conducive for neurogenesis in the *Emx1* KO mice.

In summary, we have identified Cofilin1 as one of the proteins differentially regulated in NSCs between embryonic *Emx1* WT and KO forebrains. The defect in Cofilin1 phosphorylation induced by VEGF or other growth factors may, in part, be responsible for the reduced neurogenesis observed during brain development and in adult CNS that underwent brain insult. Future studies are needed to identify pertinent neurogenesis signal pathways that still remain unaffected by *Emx1* gene deletion. In addition, several identified differentially expressed proteins would be subject for further analysis to assess their exact role in neurogenesis and their contribution to the pathologic phenotype of *Emx1* mutant.

## Author contributions

FK performed the proteomic pathway analysis and contributed to the interpretation and writing. KH conducted the migration assay and validated the differential expression of cofilin and p-cofilin. MN performed the 2D-PAGE and MS-MS analysis. SF contributed to cell counting. KJ contributed to western blot analysis. JL designed the experiments and wrote the manuscript.

## Funding

This work was supported by NIH grant R01 NS071050 (JL), VA merit award I01RX000655 (JL), and National Natural Science Foundation of China No.81403479 (SF).

### Conflict of interest statement

The authors declare that the research was conducted in the absence of any commercial or financial relationships that could be construed as a potential conflict of interest.
