# Supplementary material for: Deciphering the Role of Emx1 in Neurogenesis: A Neuroproteomics Approach
Source: Front Mol Neurosci. 2016 Oct 17;9:98. doi: 10.3389/fnmol.2016.00098 (PMC5065984; doi:10.3389/fnmol.2016.00098)
Supplement: Supplementary file 3 [file Table3.PDF]

Table 3a: Cell Differentiation Cluster

Entity Table:

Name	Type	Description	Connectivity	Probe Value	Local Connectivity	Indegree
ATP5O	Protein	ATP synthase, H+ transporting, mitochondrial F1 complex, O subunit	46		1	0
PGK1	Protein	phosphoglycerate kinase 1	155	1	0	1
PKM	Protein	pyruvate kinase, muscle	346	1	0	1
OAT	Protein	ornithine aminotransferase	98	1	0	1
ATP5A1	Protein	ATP synthase, H+ transporting, mitochondrial F1 complex, alpha subunit 1, cardiac muscle	139	1	0	1
YWHAB	Protein	tyrosine 3-monooxygenase/tryptophan 5-monooxygenase activation protein, beta polypeptide	251	1	0	1
HSPD1	Protein	heat shock 60kDa protein 1 (chaperonin)	988	1	0	1
CFL1	Protein	cofilin 1 (non-muscle)	920	1	0	1
HNRNPK	Protein	heterogeneous nuclear ribonucleoprotein K	412	1	0	1
HNRNPA1	Protein	heterogeneous nuclear ribonucleoprotein A1	353	1	0	1
PEBP1	Protein	phosphatidylethanolamine binding protein 1	384	1	0	1
PRDX1	Protein	peroxiredoxin 1	388	1	0	1
PRDX2	Protein	peroxiredoxin 2	347	1	0	1
YWHAZ	Protein	tyrosine 3-monooxygenase/tryptophan 5-monooxygenase activation protein, zeta polypeptide	557	1	0	1
PSMB6	Protein	proteasome subunit beta type 6-like	10	1	0	1
ARL3	Protein	ADP-ribosylation factor-like 3	44	1	0	1
YWHAE	Protein	tyrosine 3-monooxygenase/tryptophan 5-monooxygenase activation protein, epsilon polypeptide	328	1	0	1
YWHAG	Protein	tyrosine 3-monooxygenase/tryptophan 5-monooxygenase activation protein, gamma polypeptide	200	1	0	1
CKB	Protein	creatine kinase, brain	158	1	0	1
cell proliferation	Cell Process		8159	19	19	0

Table 3b: Cell Differentiation Cluster

Relationship Table:

Relation	Type	Sentence	TextRef	Connectivity	# of References	Organ
CFL1 ---> cell differentiation	Regulation	These were classified in to 3 main groups of proteins: cytosolic factors, chaperones and the actin-binding protein cofilin, all of which are involved in cell differentiation, survival or metabolism., Knockdown of cofilin with small-interfering RNA inhibited collagen gel contraction and reduced myofibroblast differentiation as assessed by the SMA incorporation into stress fibers., Thirteen proteins from several pathways (nucleoside diphosphate kinase A, stathmin, valosin-containing protein, annexin A1, dihydropyrimidinase-related protein-3, DJ-1 protein, glutathione S-transferase P, lamin A/C, fascin, cofilin, vimentin, vinculin, and moesin) were differentially expressed and most have been shown to play a role in differentiation, migration, invasion, proliferation, apoptosis, drug resistance, or oncogenesis. <more data available...>	info:pmid/19647006#abs:5, info:pmid/18263709#abs:11, info:pmid/19156760#abs:5, info:pmid/20060088#body:119, info:pmid/22587391#cont:204, info:pmid/18216281#body:339, info:pmid/24129212#body:21, info:pmid/19647225#body:89, info:pmid/21060807#cont:28, info:pmid/20826231#body:19	2	10	Epidermis {Organ urn:agi-ncimorgan:C0014520}, Brain {Organ urn:agi-ncimorgan:C1269537}, Liver {Organ urn:agi-ncimorgan:CL384198}
YWHAB ---> cell differentiation	Regulation	These results provide additional evidence that RNH-1/14-3-3 beta protein participates in cellular differentiation, proliferation and transformation through the signal transduction pathways of various growth factors., Thus, 14-3-3ß may	info:pmid/8749325#abs:6, info:pmid/21967815#body:153, info:pmid/17470426#body:241	2	3	

		contribute to control cell proliferation and differentiation by regulating Raf-1 and its downstream signal pathway factors., The Ca2+-dependent interactions of Scarf with annexins, calreticulin, and ERp72 may play crucial roles in Ca2+ homeostasis, molecular trafficking, and chaperone activity, and interactions with 14-3-3[?], 14-3-3ß, and nucleolin are potentially involved in growth inhibition and differentiation of keratinocytes.				
PEBP1 --+> cell differentiation	Regulation	Hippocampal cholinergic neurostimulating peptide (HCNP) is known to promote differentiation of septohippocampal cholinergic neurons., As a modulator of key signaling pathways, RKIP affects various cellular processes including cell differentiation, the cell cycle, apoptosis and cell migration., These results indicate that PEBP not only inhibits cell proliferation but also induces differentiation of human keratinocytes., These findings suggest that NGF and free-HCNP play a cooperative role during the biochemical differentiation of cholinergic neurons in medial septal nuclei. <more data available...>	info:pmid/22469272#abs:1, info:pmid/18781826#abs:4, info:pmid/15155742#abs:13, info:pmid/8070052#abs:9, info:pmid/15063784#abs:5, info:pmid/21732351#abs:1, info:pmid/15894619#body:65, info:pmid/17030190#body:176, info:pmid/24209905#cont:1007 , info:pmid/15313400#body:19 <more data available...>	2	20	medial septal nucleus {Organ urn:agi-ncimorgan:C0175233}, Heart {Organ urn:agi-ncimorgan:C1281570}, Endoderm {Organ urn:agi-ncimorgan:C0014144}, Hippocampus {Organ urn:agi-ncimorgan:C0019564}, tibialis anterior muscle {Organ urn:agi-organ:tibialis%20anterior%20muscle}, Intestines {Organ urn:agi-ncimorgan:C002

						1853}, Prostate {Organ urn:agi-ncimorgan:C1278980}
HNRNPK ---| cell differentiation	Regulation	As components of different mRNA-protein complexes, hnRNP K and hnRNP E1/E2 function in the control of mRNA translation and mRNA stability in haematopoietic cell differentiation., As components of different messenger ribonucleoprotein complexes, hnRNP K and heterogeneous nuclear ribonucleoproteins E1/E2 function as crucial modulators of mRNA stability and translation in hematopoietic cell differentiation., Regulation of neuroendocrine differentiation by AKT/hnRNPK/AR/ß-catenin signaling in prostate cancer cells. <more data available...>	info:pmid/15384226#abs:4, info:pmid/22708489#abs:4, info:pmid/22015967#title:1, info:pmid/18054780#body:90, info:pmid/21357748#cont:132, info:pmid/15671036#body:345, info:pmid/17428473#body:163, info:pmid/16004877#body:105, info:pmid/18441016#body:288	2	9	Brain {Organ urn:agi-ncimorgan:C1269537}
HNRNPA1 ---| cell differentiation	Regulation	The shuttling of hnRNP A1 is subject to regulation and is thought to play a role in cell proliferation, survival, and differentiation of normal and transformed cells (10)., The shuttling activity of hnRNP A1 has been proposed to play a role in cell proliferation, survival, and differentiation of normal and transformed cells . hnRNP A1 is also implicated in postsplicing activities, such as mRNA export and cap-dependent and internal ribosome entry site-mediated translation ., Mutational	info:pmid/15738418#body:49, info:pmid/22227431#body:16, info:pmid/23706315#body:90	2	3	

		inactivation of the hnRNP A1 binding sites alleviates inhibition of SA5639 ( ). hnRNP A1 is highly expressed in the lower to mid layers of the epithelium, but is undetectable in terminally differentiated keratinocytes that are permissive for papillomavirus late gene expression, lending support to the idea that hnRNP A1 inhibits HPV-16 late gene expression in a cell-differentiation dependent manner ( ).				
PRDX1 ---> cell differentiation	Regulation	In addition PRDX1 is involved in cell differentiation and proliferation, apoptosis and innate immunity., This indicates that OSF-3 plays an intrinsic role in the proliferation and/or differentiation of bone cells., Lastly, peroxiredoxin 1-stimulated endothelial cell proliferation, migration, and differentiation in a TLR4- and VEGF-dependent manner., Conversely, antioxidants, including glutathione peroxidase-1 and peroxiredoxin-1, suppress thioredoxin-1-expression and thereby osteoclastic differentiation., peroxiredoxin 1 induces endothelial cell proliferation, migration, and differentiation <more data available...>	info:pmid/17913528#abs:2, info:pmid/8089076#abs:5, info:pmid/21343392#abs:8, info:pmid/15358104#abs:8, info:pmid/22048194#cont:286, info:pmid/22561679#body:86, info:pmid/18996230#body:133, info:pmid/23277276#body:117, info:pmid/19766572#body:49, info:pmid/19446051#body:183 <more data available...>	2	13	Blood Vessels {Organ urn:agi-ncimorgan:C0005847}, Tongue {Organ urn:agi-ncimorgan:C1278913}, Spinal Cord {Organ urn:agi-ncimorgan:CL366046}, Aorta {Organ urn:agi-ncimorgan:C1278934}
HSPD1 --+> cell	Regulation	Finally, HSP60 effectively improves differentiation ability via reprogramming	info:pmid/21995449#cont:296,	2	5	Skin {Organ urn:agi-

differentiation		cells into a younger cell phenotype., HSP60 is involved in gene expression regulation, DNA replication, signal transduction, differentiation, apoptosis, and cellular senescence or immortalisation ., Collectively, these results suggest that HSP60 induces caspase-dependent apoptosis in osteoblast lineages without affecting osteoblastic differentiation., Alternatively, other genes, including hsp65, the gene coding for the 32-kDa protein, and the 16S-23S rRNA internal transcribed spacer, allow for the differentiation of all clinically important atypical mycobacteria. <more data available...>	info:pmid/22252117#body:158, info:pmid/19306954#body:92, info:pmid/23174494#body:42, info:pmid/10779593#body:164			ncimorgan:C0684084}, Testis {Organ urn:agi-ncimorgan:C1278981}
ENO1 ---> cell differentiation	Regulation	We suggest that alpha-enolase may play a significant role in the differentiation of lymphoma in man., Enolase 1 and calreticulin regulate the differentiation and function of mouse mast cells., In addition to this role in the glycolytic pathway, ENO1 is also reactive to hypoxic stress (38) and acts as a Myc-binding protein to regulate cell growth and differentiation (39)., Moreover, some investigators have found that enolase 1 can regulate the differentiation and function of mouse mast cells with calreticulin (spot 6), which was also found in our study [62]. <more data available...>	info:pmid/7787969#abs:9, info:pmid/21803152#title:1, info:pmid/21795435#cont:304, info:pmid/24116183#cont:894, info:pmid/17289554#body:30	2	5	Intestines {Organ urn:agi-ncimorgan:C0021853}, Eye {Organ urn:agi-ncimorgan:C1550636}
CNN3 ---> cell differentiation	Regulation	Thus, our study does not exclude the possibility that acidic calponin also plays a role in ß-cell differentiation, and the functional significance of acidic calponin	info:pmid/12606518#body:239, info:pmid/23276748#body:206	2	2	Skeletal system {Organ urn:agi-ncimorgan:C1283922}

		remains to be determined., These observations indicate that Rho-associated kinase-dependent CNN3 phosphorylation is required not only for membrane fusion but also for progression of the differentiation program.				
PRDX2 ---> cell differentiation	Regulation	Previous studies have suggested that Prdx2 can regulate many cellular functions including cell proliferation and differentiation ., Prx II participates in regulation of cellular functions such as cell proliferation and differentiation, and protects a number of specific proteins from oxidative damage ., peroxiredoxin-2, a novel group of peroxidases containing high anti-oxidant efficiency and which can also have a role in cell differentiation and apoptosis, was over-expressed in response to TPA, 12-dimethylbenz[a]anthracene and glyphosate (,) . <more data available...>	info:pmid/24125860#body:139, info:pmid/22749995#body:5, info:pmid/20045496#body:156, info:pmid/21248284#cont:20	2	4	Colorectal Region {Organ urn:agi-ncimorgan:C1711309}
ATP5O ---> cell differentiation	Regulation	Based upon our results we suggest that ATPo acts as a generalized signal for growth and differentiation in lineages of thymocytes destined to become mature T cells.	info:pmid/9281578#body:291	2	1	
YWHAZ ---| cell differentiation	Regulation	Figure 5. miR-451 repression of 14-3-3z regulates erythroid differentiation.	info:pmid/20679397#cont:160	2	1	
ATP5A1 ---> cell differentiation	Regulation	The identified copy number amplification genes, such as ZNF521, RNF138, RAB12, ATP5A1, PTPN2 and CTAGE1, are involved in multiple cellular processes, including transcription, proliferation, differentiation, migration and immunity.	info:pmid/23678296#cont:270	2	1	Fetus {Organ urn:agi-ncimorgan:C1305737}

<table>
<tr><td>PKM ---| cell differentiation</td><td>Regulation</td><td>The interference of pkm2 gene could significantly enhance the cell differentiation in the drug resistant NB4R2 cell line., Silencing of PKM2 enhances both apoptosis and differentiation of rat and human glioma spheroids., According to previous reports, pyruvate kinase M2 plays an important role in both cell proliferation and differentiation.</td><td>info:pmid/20137124#abs:8, info:pmid/24481450#abs:3, info:pmid/21526124#cont:206</td><td>2</td><td>3</td><td></td></tr>
<tr><td>OAT ---> cell differentiation</td><td>Regulation</td><td>This could mean that carbonyl reductase 1, ornithine aminotransferase, HSP27, GIPC1 and PSAT1 up-regulate adipogenic differentiation and down-regulate osteogenic differentiation in BM-mesenchymal stem cells.</td><td>info:pmid/22615730#cont:300</td><td>2</td><td>1</td><td></td></tr>
<tr><td>CKB --+> cell differentiation</td><td>Regulation</td><td>The amount of brain-type creatine kinase increased only twofold during P19 differentiation.</td><td>info:pmid/9648858#abs:5</td><td>2</td><td>1</td><td></td></tr>
<tr><td>PGK1 --+> cell differentiation</td><td>Regulation</td><td>We show that Prostate cancer-derived PGK1 induces osteoblastic differentiation of bone marrow stromal cells.</td><td>info:pmid/19825988#abs:5</td><td>2</td><td>1</td><td>Bone Marrow {Organ urn:agi-ncimorgan:C0005953}</td></tr>
<tr><td>ATP5A1 ---> cell proliferation</td><td>Regulation</td><td>An antibody against the ATP synthase a-subunit inhibited proliferation, migration and invasion in these breast cancer cells but not that of a non-tumor derived breast cell line., The identified copy number amplification genes, such as ZNF521, RNF138, RAB12, ATP5A1, PTPN2 and CTAGE1, are involved in multiple cellular processes, including transcription, proliferation, differentiation, migration and immunity., Mitochondrial ATP synthase inhibitors antagonize 5-Fluorouracil-</td><td>info:pmid/22152132#abs:10, info:pmid/23678296#cont:270, info:pmid/15833846#body:183, info:pmid/15753359#body:256</td><td>2</td><td>4</td><td>Fetus {Organ urn:agi-ncimorgan:C1305737}</td></tr>
</table>

		induced suppression of cell proliferation. <more data available...>				
CKB ---> cell proliferation	Regulation	It was found that Creatine kinase B knockdown inhibited Skov3 cell proliferation and induced apoptosis under hypoxia or hypoglycemia conditions., Furthermore, overexpression of CFP-tagged wild-type Creatine kinase brain in Caco-2 colon cancer cells dramatically increased the number of cells in G2/M but had little effect on cell proliferation.	info:pmid/23416112#abs:5, info:pmid/21308735#abs:7	2	2	

Table 3c: Cell Differentiation Cluster

Reference Table:

Relation	Type	Sentence	TextRef	Organ
CFL1 ---> cell differentiation	Regulation	These were classified in to 3 main groups of proteins: cytosolic factors, chaperones and the actin-binding protein cofilin, all of which are involved in cell differentiation, survival or metabolism.	info:pmid/19647006#abs:5	
CFL1 ---> cell differentiation	Regulation	Knockdown of cofilin with small-interfering RNA inhibited collagen gel contraction and reduced myofibroblast differentiation as assessed by the SMA incorporation into stress fibers.	info:pmid/18263709#abs:11	
CFL1 ---> cell differentiation	Regulation	Thirteen proteins from several pathways (nucleoside diphosphate kinase A, stathmin, valosin-containing protein, annexin A1, dihydropyrimidinase-related protein-3, DJ-1 protein, glutathione S-transferase P, lamin A/C, fascin, cofilin, vimentin, vinculin, and moesin) were differentially expressed and most have been shown to play a role in differentiation, migration, invasion, proliferation, apoptosis, drug resistance, or oncogenesis.	info:pmid/19156760#abs:5	
CFL1 ---> cell differentiation	Regulation	CO6a2, CO3a1, cofilin, nonmuscle isoform and HSPß1 had a tendency to increase with the differentiation.	info:pmid/20060088#body:119	
CFL1 ---> cell differentiation	Regulation	These results suggest that cofilin is involved in p130Cas-mediated differentiation of C2C12 myoblasts.	info:pmid/22587391#cont:204	
CFL1 ---> cell differentiation	Regulation	Inactivation of cofilin by LIMK is essential in differentiation and migration (Dawe et al., 2003[Go]; Nishita et al., 2005[Go]).	info:pmid/18216281#body:339	
CFL1 ---> cell differentiation	Regulation	We found that CFL1 increased the level of actin polymerization and inhibited brown preadipocyte differentiation.	info:pmid/24129212#body:21	
CFL1 ---> cell differentiation	Regulation	Cofilin phosphorylation increases during the differentiation of cultured human keratinocytes and has been implicated in the compaction of the granular layer of human epidermis in organotypic cultures ( ).	info:pmid/19647225#body:89	Epidermis {Organ urn:agi-ncimorgan:C0014520}
CFL1 ---> cell differentiation	Regulation	While Dstn2/2 brains have a normal gross morphology, conditional deletion of Cfl1 in neuronal cells causes excessive differentiation, changes in cell proliferation, and migration defects, resulting in a	info:pmid/21060807#cont:28	Brain {Organ urn:agi-ncimorgan:C1269537}

		lissencephaly phenotype [5].		
CFL1 ---> cell differentiation	Regulation	Moreover, performed proteomic analysis of differentiating neuroblastoma cells treated with diazinon and identified a number of up-regulated proteins classified as cytosolic factors, chaperones and the actin-binding protein cofilin, all of which are involved in cell differentiation, survival or metabolism.	info:pmid/20826231#body:19	Liver {Organ urn:agi-ncimorgan:CL384198}
YWHAB ---> cell differentiation	Regulation	These results provide additional evidence that RNH-1/14-3-3 beta protein participates in cellular differentiation, proliferation and transformation through the signal transduction pathways of various growth factors.	info:pmid/8749325#abs:6	
YWHAB ---> cell differentiation	Regulation	Thus, 14-3-3ß may contribute to control cell proliferation and differentiation by regulating Raf-1 and its downstream signal pathway factors.	info:pmid/21967815#body:153	
YWHAB ---> cell differentiation	Regulation	The Ca2+-dependent interactions of Scarf with annexins, calreticulin, and ERp72 may play crucial roles in Ca2+ homeostasis, molecular trafficking, and chaperone activity, and interactions with 14-3-3[?], 14-3-3ß, and nucleolin are potentially involved in growth inhibition and differentiation of keratinocytes.	info:pmid/17470426#body:241	
PEBP1 --+> cell differentiation	Regulation	Hippocampal cholinergic neurostimulating peptide (HCNP) is known to promote differentiation of septohippocampal cholinergic neurons.	info:pmid/22469272#abs:1	
PEBP1 --+> cell differentiation	Regulation	As a modulator of key signaling pathways, RKIP affects various cellular processes including cell differentiation, the cell cycle, apoptosis and cell migration.	info:pmid/18781826#abs:4	
PEBP1 --+> cell differentiation	Regulation	These results indicate that PEBP not only inhibits cell proliferation but also induces differentiation of human keratinocytes.	info:pmid/15155742#abs:13	
PEBP1 --+> cell differentiation	Regulation	These findings suggest that NGF and free-HCNP play a cooperative role during the biochemical differentiation of cholinergic neurons in medial septal nuclei.	info:pmid/8070052#abs:9	medial septal nucleus {Organ urn:agi-ncimorgan:C0175233}
PEBP1 --+> cell differentiation	Regulation	As mlc2a is involved in heart morphogenesis, and PEBP controls the proliferation and differentiation of different cell types, these genes are candidates for involvement in Down syndrome-Congenital heart disease.	info:pmid/15063784#abs:5	Heart {Organ urn:agi-ncimorgan:C1281570}
PEBP1 --+> cell	Regulation	Raf kinase inhibitor protein (RKIP) regulates growth and	info:pmid/21732351#	

differentiation		differentiation signaling of mitogen-activated protein kinases , GRK2 and NF-kappaB pathways each of which regulates cytotrophoblast differentiation and normal placental development.	abs:1	
PEBP1 --+> cell differentiation	Regulation	The phosphatidylethanolamine-binding protein family regulates signaling pathways to control growth and differentiation.	info:pmid/15894619#body:65	
PEBP1 --+> cell differentiation	Regulation	In addition, RKIP expression probably contributes to Hepatocellular carcinoma cell differentiation.	info:pmid/17030190#body:176	Endoderm {Organ urn:agi-ncimorgan:C0014144}
PEBP1 --+> cell differentiation	Regulation	Our previous study also revealed that the abnormal expression of RKIP plays an important role in the growth and differentiation process of gastric cancer.	info:pmid/24209905#cont:1007	
PEBP1 --+> cell differentiation	Regulation	In addition, it was postulated that RKIP may be involved in growth, transformation, and differentiation , which are often deregulated in many forms of cancer.	info:pmid/15313400#body:19	
PEBP1 --+> cell differentiation	Regulation	NMDA receptor activation enhances the release of a cholinergic differentiation peptide (HCNP) from hippocampal neurons in vitro.	info:pmid/9555001#title:1	Hippocampus {Organ urn:agi-ncimorgan:C0019564}
PEBP1 --+> cell differentiation	Regulation	Collectively, phosphatidylethanolamine-binding protein-1 affects various cellular processes including cell differentiation, cell cycle, apoptosis, and cell migration [32].	info:pmid/22227918#cont:639	tibialis anterior muscle {Organ urn:agi-organ:tibialis%20anterior%20muscle}
PEBP1 --+> cell differentiation	Regulation	Thus, RKIP mediates important cellular mechanisms, including cell differentiation, cell cycle, apoptosis and cell migration, and is deregulated in several human disorders (13).	info:pmid/23232914#cont:16	Intestines {Organ urn:agi-ncimorgan:C0021853}
PEBP1 --+> cell differentiation	Regulation	Moreover, it was also postulated that PEBP is involved in growth, transformation, and differentiation, which are often deregulated in many forms of cancer (Keller et al., 2004[Go]).	info:pmid/16608915#body:57	
PEBP1 --+> cell differentiation	Regulation	Interestingly, RKIP promotes differentiation of human keratino-cytes (Yamazaki et al, 2004), and both RKIP and B-Raf are downregulated in human SCC (Zaravinos et al, 2009), whereas Raf-1 is overexpressed (Riva et al, 1995).	info:pmid/21081934#cont:151	
PEBP1 --+> cell differentiation	Regulation	The NMDA-receptor, but not the AMPA receptor, mediates the release of the novel undecapeptide, HCNP, which then stimulates the differentiation of presynaptic septal cholinergic neurons.	info:pmid/10622376#body:379	

PEBP1 --+> cell differentiation	Regulation	Hence, evidence indicates that RKIP regulates the activity and mediates the crosstalk between several important cellular signaling pathways including cell differentiation, cell cycle, apoptosis and cell migration.	info:pmid/23601922#body:15	Prostate {Organ urn:agi-ncimorgan:C1278980}
PEBP1 --+> cell differentiation	Regulation	In the hippocampus, RKIP is thought to be a precursor of hippocampal cholinergic neuro-stimulating peptide (HCNP), which promotes the differentiation of hippocampal cholinergic neurons (Ojika et al., 2000).	info:pmid/23055494#cont:356	Hippocampus {Organ urn:agi-ncimorgan:C0019564}
PEBP1 --+> cell differentiation	Regulation	In addition, other bioactive peptides have been identified and found to represent fragments of cytosolic proteins; examples include diazepam-binding inhibitor, a fragment of acyl-CoA-binding protein that binds to GABAA receptors; hippocampal cholinergic neurostimulating peptide (HCNP), a fragment of phosphatidylethanolamine-binding protein that enhances the differentiation of hippocampal neurons; microcryptide-1, a fragment of cytochrome c oxidase subunit 8 that activates neutrophils; and ...	info:pmid/19380512#body:262	Hippocampus {Organ urn:agi-ncimorgan:C0019564}
PEBP1 --+> cell differentiation	Regulation	To clarify whether upregulation of RKIP during monocytic differentiation is a secondary effect or whether RKIP expression induces or supports the differentiation process, we transiently transfected THP-1 cells with an RKIP expression plasmid and analyzed the mRNA levels and cell surface expression of the pan-leukocyte marker CD45 and the macrophage specific maturation markers CD11c (leukocyte integrin) and CD36 (scavenger receptor).	info:pmid/16513087#body:90	
HNRNPK ---| cell differentiation	Regulation	As components of different mRNA-protein complexes, hnRNP K and hnRNP E1/E2 function in the control of mRNA translation and mRNA stability in haematopoietic cell differentiation.	info:pmid/15384226#abs:4	
HNRNPK ---| cell differentiation	Regulation	As components of different messenger ribonucleoprotein complexes, hnRNP K and heterogeneous nuclear ribonucleoproteins E1/E2 function as crucial modulators of mRNA stability and translation in hematopoietic cell differentiation.	info:pmid/22708489#abs:4	
HNRNPK ---| cell differentiation	Regulation	Regulation of neuroendocrine differentiation by AKT/hnRNPK/AR/ß-catenin signaling in prostate cancer cells.	info:pmid/22015967#title:1	

HNRNPK ---| cell differentiation	Regulation	For example HuB binding to p21 mRNA is directly antagonized by hnRNP K, which inhibits both p21 translation and cell differentiation ( ).	info:pmid/18054780#body:90	
HNRNPK ---| cell differentiation	Regulation	Note that the association between A6B1-integrin and hnRNP-K is greater on laminin substrates and increases further on differentiation.	info:pmid/21357748#cont:132	
HNRNPK ---| cell differentiation	Regulation	These reports and our results suggest that hnRNP K may promote cell proliferation and have a negative effect against the promotion of differentiation in vivo.	info:pmid/15671036#body:345	Brain {Organ urn:agi-ncimorgan:C1269537}
HNRNPK ---| cell differentiation	Regulation	A few of these specific examples, however, provide some insights into their possible functions in neurons. hnRNP K, for example, directly antagonizes the binding of HuB to the cyclin-dependent kinase inhibitor p21 mRNA 3'UTR to regulate neuroblastomal differentiation .	info:pmid/17428473#body:163	
HNRNPK ---| cell differentiation	Regulation	However, it is known that hnRNP K plays an important role in erythroid cell differentiation mediating 15-lipoxygenase mRNA silencing translation by binding to the differentiation control element sequence in the 3' UTR of the 15-lipoxygenase mRNA ( ).	info:pmid/16004877#body:105	
HNRNPK ---| cell differentiation	Regulation	We found that hnRNP K not only functions as a translational regulator of human reticulocyte 15-lipoxygenase mRNA (Fig. 2) but also represses expression of c-Src (Figs. 5 and 6), an important regulator of cell cycle control, proliferation, and differentiation (44, 45).	info:pmid/18441016#body:288	
HNRNPA1 ---| cell differentiation	Regulation	The shuttling of hnRNP A1 is subject to regulation and is thought to play a role in cell proliferation, survival, and differentiation of normal and transformed cells (10).	info:pmid/15738418#body:49	
HNRNPA1 ---| cell differentiation	Regulation	The shuttling activity of hnRNP A1 has been proposed to play a role in cell proliferation, survival, and differentiation of normal and transformed cells . hnRNP A1 is also implicated in postsplicing activities, such as mRNA export and cap-dependent and internal ribosome entry site-mediated translation .	info:pmid/22227431#body:16	
HNRNPA1 ---| cell differentiation	Regulation	Mutational inactivation of the hnRNP A1 binding sites alleviates inhibition of SA5639 ( ). hnRNP A1 is highly expressed in the lower to mid layers of the epithelium, but is undetectable in terminally	info:pmid/23706315#body:90	

		differentiated keratinocytes that are permissive for papillomavirus late gene expression, lending support to the idea that hnRNP A1 inhibits HPV-16 late gene expression in a cell-differentiation dependent manner ( ).		
PRDX1 ---> cell differentiation	Regulation	In addition PRDX1 is involved in cell differentiation and proliferation, apoptosis and innate immunity.	info:pmid/17913528#abs:2	
PRDX1 ---> cell differentiation	Regulation	This indicates that OSF-3 plays an intrinsic role in the proliferation and/or differentiation of bone cells.	info:pmid/8089076#abs:5	
PRDX1 ---> cell differentiation	Regulation	Lastly, peroxiredoxin 1-stimulated endothelial cell proliferation, migration, and differentiation in a TLR4- and VEGF-dependent manner.	info:pmid/21343392#abs:8	Blood Vessels {Organ urn:agi-ncimorgan:C0005847}
PRDX1 ---> cell differentiation	Regulation	Conversely, antioxidants, including glutathione peroxidase-1 and peroxiredoxin-1, suppress thioredoxin-1-expression and thereby osteoclastic differentiation.	info:pmid/15358104#abs:8	
PRDX1 ---> cell differentiation	Regulation	peroxiredoxin 1 induces endothelial cell proliferation, migration, and differentiation	info:pmid/22048194#cont:286	Blood Vessels {Organ urn:agi-ncimorgan:C0005847}
PRDX1 ---> cell differentiation	Regulation	peroxiredoxin 1 in turn can inhibit medial edge epithelial apoptosis, proliferation, and differentiation.	info:pmid/22561679#body:86	
PRDX1 ---> cell differentiation	Regulation	Additionally, peroxiredoxin-1 is one of the antioxidant enzymes and is involved in cellular proliferation and differentiation .	info:pmid/18996230#body:133	
PRDX1 ---> cell differentiation	Regulation	Altogether these data suggest that Prdx1 knockdown might promote the differentiation of HepG2 cells into a more mature form of hepatocyte.	info:pmid/23277276#body:117	
PRDX1 ---> cell differentiation	Regulation	TUNEL analysis showed no difference in cell death between Prdx1 -/- and wild-type embryos, suggesting that Prdx1 regulates the differentiation of motor neurons rather than their survival ( ).	info:pmid/19766572#body:49	
PRDX1 ---> cell differentiation	Regulation	Peroxiredoxin-1, also known as PAG (proliferation-associated gene), is a scavenger of reactive oxygen species, and is known to be involved in the redox regulation of cellular signalling and differentiation .	info:pmid/19446051#body:183	Tongue {Organ urn:agi-ncimorgan:C1278913}
PRDX1 ---> cell differentiation	Regulation	Prx I gene activation by the phorbol ester O-tetradecanoylphorbol-13-acetate, which primarily affects cellular proliferation and differentiation, is inhibited by the bacterial product	info:pmid/18070609#body:182	

		lipopolysaccharide, which is a prototypical proinflammatory mediator.		
PRDX1 ---> cell differentiation	Regulation	This signaling mechanism may more generally regulate neural development; in addition to motor neurons, the misexpression of GDE2 and Prdx1 can promote the differentiation of other subtypes of neurons throughout the spinal cord.	info:pmid/19766560#body:23	Spinal Cord {Organ urn:agi-ncimorgan:CL366046}
PRDX1 ---> cell differentiation	Regulation	... shear versus static) had many of the same genes significantly regulated as other published vascular microarray studies, including upregulation of antioxidants Cytochrome P450 peptide 1A1, PRDX1, and SOD2 and downregulation of a proinflammatory mediator Bone morphogenic protein 4.17-19 In addition, shear stress also protects both endothelial cell types from chondro/osteogenic differentiation, which is in accordance with the ventricular endothelial protection from this differentiation ...	info:pmid/16293796#body:164	Aorta {Organ urn:agi-ncimorgan:C1278934}
HSPD1 --+> cell differentiation	Regulation	Finally, HSP60 effectively improves differentiation ability via reprogramming cells into a younger cell phenotype.	info:pmid/21995449#cont:296	
HSPD1 --+> cell differentiation	Regulation	HSP60 is involved in gene expression regulation, DNA replication, signal transduction, differentiation, apoptosis, and cellular senescence or immortalisation .	info:pmid/22252117#body:158	
HSPD1 --+> cell differentiation	Regulation	Collectively, these results suggest that HSP60 induces caspase-dependent apoptosis in osteoblast lineages without affecting osteoblastic differentiation.	info:pmid/19306954#body:92	
HSPD1 --+> cell differentiation	Regulation	Alternatively, other genes, including hsp65, the gene coding for the 32-kDa protein, and the 16S-23S rRNA internal transcribed spacer, allow for the differentiation of all clinically important atypical mycobacteria.	info:pmid/23174494#body:42	Skin {Organ urn:agi-ncimorgan:C0684084}
HSPD1 --+> cell differentiation	Regulation	It may be that CPN60 in the cytosol orchestrates protein folding during terminal differentiation of activated sperm, as it does during structural modifications of germ cell mitochondria during spermatogenesis in the human testes .	info:pmid/10779593#body:164	Testis {Organ urn:agi-ncimorgan:C1278981}
ENO1 ---> cell differentiation	Regulation	We suggest that alpha-enolase may play a significant role in the differentiation of lymphoma in man.	info:pmid/7787969#abs:9	
ENO1 ---> cell	Regulation	Enolase 1 and calreticulin regulate the differentiation and function	info:pmid/21803152#t	

differentiation		of mouse mast cells.	itle:1	
ENO1 ---> cell differentiation	Regulation	In addition to this role in the glycolytic pathway, ENO1 is also reactive to hypoxic stress (38) and acts as a Myc-binding protein to regulate cell growth and differentiation (39).	info:pmid/21795435#cont:304	Intestines {Organ urn:agi-ncimorgan:C0021853}
ENO1 ---> cell differentiation	Regulation	Moreover, some investigators have found that enolase 1 can regulate the differentiation and function of mouse mast cells with calreticulin (spot 6), which was also found in our study [62].	info:pmid/24116183#cont:894	
ENO1 ---> cell differentiation	Regulation	When located in the nucleus, alpha-enolase is a Myc-binding protein (MBP-1), playing a crucial role in the regulation of cell growth and differentiation, as a negative regulator of the c-myc protooncogene leading to tumor suppression, and in the binding to cytoskeletal and chromatin structures.	info:pmid/17289554#body:30	Eye {Organ urn:agi-ncimorgan:C1550636}
CNN3 ---> cell differentiation	Regulation	Thus, our study does not exclude the possibility that acidic calponin also plays a role in ß-cell differentiation, and the functional significance of acidic calponin remains to be determined.	info:pmid/12606518#body:239	
CNN3 ---> cell differentiation	Regulation	These observations indicate that Rho-associated kinase-dependent CNN3 phosphorylation is required not only for membrane fusion but also for progression of the differentiation program.	info:pmid/23276748#body:206	Skeletal system {Organ urn:agi-ncimorgan:C1283922}
PRDX2 ---> cell differentiation	Regulation	Previous studies have suggested that Prdx2 can regulate many cellular functions including cell proliferation and differentiation .	info:pmid/24125860#body:139	Colorectal Region {Organ urn:agi-ncimorgan:C1711309}
PRDX2 ---> cell differentiation	Regulation	Prx II participates in regulation of cellular functions such as cell proliferation and differentiation, and protects a number of specific proteins from oxidative damage .	info:pmid/22749995#body:5	
PRDX2 ---> cell differentiation	Regulation	peroxiredoxin-2, a novel group of peroxidases containing high anti-oxidant efficiency and which can also have a role in cell differentiation and apoptosis, was over-expressed in response to TPA, 12-dimethylbenz[a]anthracene and glyphosate (,) .	info:pmid/20045496#body:156	
PRDX2 ---> cell differentiation	Regulation	PRDX2 orchestrates multiple cellular functions, including protecting protein and lipid against oxidative injury, cell proliferation, and differentiation, and mediates intracellular signaling pathways involved in apoptosis through the elimination of H2O2 [10].	info:pmid/21248284#cont:20	

ATP5O ---> cell differentiation	Regulation	Based upon our results we suggest that ATPo acts as a generalized signal for growth and differentiation in lineages of thymocytes destined to become mature T cells.	info:pmid/9281578#body:291	
YWHAZ ---| cell differentiation	Regulation	Figure 5. miR-451 repression of 14-3-3z regulates erythroid differentiation.	info:pmid/20679397#cont:160	
ATP5A1 ---> cell differentiation	Regulation	The identified copy number amplification genes, such as ZNF521, RNF138, RAB12, ATP5A1, PTPN2 and CTAGE1, are involved in multiple cellular processes, including transcription, proliferation, differentiation, migration and immunity.	info:pmid/23678296#cont:270	Fetus {Organ urn:agi-ncimorgan:C1305737}
PKM ---| cell differentiation	Regulation	The interference of pkm2 gene could significantly enhance the cell differentiation in the drug resistant NB4R2 cell line.	info:pmid/20137124#abs:8	
PKM ---| cell differentiation	Regulation	Silencing of PKM2 enhances both apoptosis and differentiation of rat and human glioma spheroids.	info:pmid/24481450#abs:3	
PKM ---| cell differentiation	Regulation	According to previous reports, pyruvate kinase M2 plays an important role in both cell proliferation and differentiation.	info:pmid/21526124#cont:206	
OAT ---> cell differentiation	Regulation	This could mean that carbonyl reductase 1, ornithine aminotransferase, HSP27, GIPC1 and PSAT1 up-regulate adipogenic differentiation and down-regulate osteogenic differentiation in BM-mesenchymal stem cells.	info:pmid/22615730#cont:300	
CKB --+> cell differentiation	Regulation	The amount of brain-type creatine kinase increased only twofold during P19 differentiation.	info:pmid/9648858#abs:5	
PGK1 --+> cell differentiation	Regulation	We show that Prostate cancer-derived PGK1 induces osteoblastic differentiation of bone marrow stromal cells.	info:pmid/19825988#abs:5	Bone Marrow {Organ urn:agi-ncimorgan:C0005953}
PKM --+> cell proliferation	Regulation	Knockdown of PKM2 attenuated PRL-stimulated cell proliferation.	info:pmid/20962042#abs:5	
PKM --+> cell proliferation	Regulation	Knockdown of PKM2 repressed proliferation and migration of the cells.	info:pmid/22807066#abs:6	
PKM --+> cell proliferation	Regulation	Acetylation-mimetic PKM2(K433) mutant promotes cell proliferation and tumorigenesis.	info:pmid/24120661#abs:5	
PKM --+> cell proliferation	Regulation	Additionally, PKM2-SAICAR was necessary to induce sustained Erk1/2 activation and mitogen-induced cell proliferation.	info:pmid/24606918#abs:7	
PKM --+> cell	Regulation	Expression of a PKM2 mutant that exists as a dimer promotes cell	info:pmid/22306293#	

proliferation		proliferation, indicating that protein kinase activity of PKM2 plays a role in promoting cell proliferation.	abs:8	
PKM --+> cell proliferation	Regulation	Cancer cells universally express the M2 isoform of the glycolytic enzyme pyruvate kinase (PKM2), and previous work has demonstrated that PKM2 expression is necessary for aerobic glycolysis and cell proliferation in vivo.	info:pmid/20005212#abs:3	
PKM --+> cell proliferation	Regulation	The prevalence of PKM2 in cancer cells relative to the prevalence of PKM1 in many normal cells, suggests a therapeutic strategy whereby activation of PKM2 may counter the abnormal cellular metabolism in cancer cells, and consequently decreased cellular proliferation.	info:pmid/22963766#abs:5	
PKM --+> cell proliferation	Regulation	Furthermore, the growth suppression effect of Cyclosporin A was impaired in MCF-7 cells when they were transfected with the PKM2 overexpression plasmid, suggesting that Cyclosporin A was an effective inhibitor of PKM2-dependent proliferation of breast cancer cells.	info:pmid/22580449#abs:7	
PKM --+> cell proliferation	Regulation	The nuclear functions of PKM2 contribute to cell proliferation.	info:pmid/23791887#body:130	
PKM --+> cell proliferation	Regulation	Overall, it appears that PKM2 nuclear functions contribute to cell proliferation.	info:pmid/22626471#body:114	
PKM --+> cell proliferation	Regulation	Thus, both cytosolic and nuclear PKM2 contribute to altered metabolism and proliferation in cancer.	info:pmid/24344305#cont:32	
PKM --+> cell proliferation	Regulation	One possibility is that PKM2 loss changes the balance of proliferation and apoptosis.	info:pmid/24120138#body:59	
PKM --+> cell proliferation	Regulation	Pyruvate kinase M2 promotes de novo serine synthesis to sustain mTORC1 activity and cell proliferation.	info:pmid/22509023#title:1	
PKM --+> cell proliferation	Regulation	Pyruvate kinase M2 promotes de novo serine synthesis to sustain mTORC1 activity and cell proliferation.	info:pmid/24305570#cont:482	
PKM --+> cell proliferation	Regulation	These results confirmed that PKM2 is critical for cancer metabolism and cellular proliferation.	info:pmid/22574221#cont:126	
PKM --+> cell proliferation	Regulation	All of these studies suggest that pyruvate kinase M2 plays an important role in cell proliferation.	info:pmid/21526124#cont:218	
PKM --+> cell proliferation	Regulation	These results indicate that EGFR-increased PKM2 expression is required for EGFR-promoted cell proliferation.	info:pmid/23123196#body:142	

PKM --+> cell proliferation	Regulation	Inhibition of mTOR, glycolysis, and PKM2 suppresses cell proliferation and tumorigenesis.	info:pmid/21325052#cont:185	
PKM --+> cell proliferation	Regulation	Once in the nucleus, PKM2 activates ß-catenin, leading to cell proliferation and tumorigenesis .	info:pmid/22617155#body:151	
PKM --+> cell proliferation	Regulation	Recently, Christofk et al. found that PKM2 is necessary for aerobic glycolysis and cell proliferation in vivo .	info:pmid/21334407#body:132	Fetus {Organ urn:agi-ncimorgan:C1305737}
PKM --+> cell proliferation	Regulation	This overexpression of PKM2 is involved in promoting proliferation and migration of some types of cancer cells (2, 5, 8).	info:pmid/23846818#cont:39	
PKM --+> cell proliferation	Regulation	Additionally, PKM2 promotes de novo serine synthesis to stimulate mTORC1 activity and sustain cell proliferation .	info:pmid/23523716#body:38	
PKM --+> cell proliferation	Regulation	Also, PKM2 inhibition by prolactin increases lactate content and stimulates proliferation in human cell lines (Varghese et al. 2010).	info:pmid/21985671#cont:814	
PKM --+> cell proliferation	Regulation	These observations are consistent with previous data showing that replacement of PKM2 with PKM1 impairs cell proliferation under low oxygen conditions8.	info:pmid/22922757#cont:427	
PKM --+> cell proliferation	Regulation	Pyruvate kinase isoform M2 (PKM2) is thought to critically regulate aerobic glycolysis for supplying metabolic intermediates for tumor cell proliferation .	info:pmid/21130743#body:91	
PKM --+> cell proliferation	Regulation	Thr-454 phosphorylation of PKM2 increases cancer cell proliferation. a, A549 cells were transfected with HA-tagged PKM2-WT or T454A.	info:pmid/24142698#cont:266	
PKM --+> cell proliferation	Regulation	They show that activating PKM2 suppresses cell proliferation — but severely inhibiting this enzyme is known to have similar effects, and can induce tumour regression in mice8,9.	info:pmid/23018962#cont:57	
PKM --+> cell proliferation	Regulation	Decreased PKM2 activity as a result of oncogenic signaling pathway activation is thought to contribute to tumor cell proliferation by enabling use of upstream glycolytic intermediates for biosynthesis .	info:pmid/22406683#body:89	
PKM --+> cell proliferation	Regulation	These novel activators of PKM2 provide the necessary tool compounds to explore the hypothesis that PKM2 activation will ameliorate the Warburg effect, and thereby decrease cancer cell proliferation.	info:pmid/20451379#body:122	
PKM --+> cell proliferation	Regulation	Furthermore, the most recent study showed that aside from its key role in tumor metabolism, PKM2 has the same function of protein kinase and can directly regulate cancer cell proliferation .	info:pmid/23880164#body:16	

PKM --+> cell proliferation	Regulation	Re-expression of PKM2 in PKM2 knockdown cells was reported to promote cell proliferation under hypoxic conditions , suggesting a role of PKM2 in the adaptive hypoxia response.	info:pmid/24508027#body:85	
PKM --+> cell proliferation	Regulation	In addition to its well-known role in glycolysis, PKM2 regulates proliferation and apoptosis of nontransformed cells in a cell-type-specific manner by largely unknown mechanisms (; ;).	info:pmid/22901803#body:10	Fetus {Organ urn:agi-ncimorgan:C1305737}
PKM --+> cell proliferation	Regulation	Since we found that METH preferentially acts on proliferating neural progenitor cells, we next sought to confirm nitrotyrosination of pyruvate kinase M2 , a protein that mediates cell proliferation.	info:pmid/21708025#cont:168	
PKM --+> cell proliferation	Regulation	insight as to whether the predominant expression of PKM2 observed in tumors is necessary for cancer cell proliferation, we investigated the role of PKM2 in tumor cell growth and maintenance.	info:pmid/23267074#cont:38	
PKM --+> cell proliferation	Regulation	Nuclear PKM2 phosphorylated the transcription factor STAT3 using phosphoenolpyruvate as a phosphate donor, and activation of STAT3 by PKM2 stimulated transcription of the MEK5 gene to increase cell proliferation ( ) .	info:pmid/22824010#body:108	
PKM --+> cell proliferation	Regulation	Recent studies showed that shikonin represses tumor pyruvate kinase M2 (PKM2) activity, which inhibits the metabolic rates of glucose and lactate in cancer cells and, thereby, suppresses cancer cell proliferation ( ).	info:pmid/23562787#body:151	
PKM --+> cell proliferation	Regulation	Knockdown of PKM2 using RNA interference significantly impairs cell growth in tissue culture, inhibition of PKM2 with peptide aptamers inhibits cell proliferation, and PKM2 expression is necessary for both aerobic glycolysis and tumor growth in vivo (31,32).	info:pmid/22581080#cont:526	Fetus {Organ urn:agi-ncimorgan:C1305737}
PKM --+> cell proliferation	Regulation	PKM2 depletion largely reduced both basal and EGF-induced tumour cell proliferation (Fig. 1b) and blocked EGF-enhanced expression of cyclin D1 and c-Myc (Fig. 1c), which are known to be important regulators of cell proliferation and downstream genes of b-catenin transactivation8.	info:pmid/22056988#cont:18	
PKM --+> cell proliferation	Regulation	The results strongly support our hypothesis that protein kinase activity of PKM2 promotes tumor/cell proliferation.	info:doi/10.1016/j.molcel.2012.01.001#body:	

			179	
PKM --+> cell proliferation	Regulation	Decreased PKM2 activity as a result of oncogenic signaling pathway activation is thought to contribute to tumor cell proliferation by enabling use of upstream glycolytic intermediates for biosynthesis .	info:doi/10.1016/j.semcdb.2012.02.003#body:89	
PKM --+> cell proliferation	Regulation	This suggests that pharmacological activation of PKM2 to levels associated with PKM1 may inhibit cell proliferation as well as be a potential therapeutic strategy for cancer.	info:doi/10.1016/j.bmcl.2011.08.114#body:13	
PKM --+> cell proliferation	Regulation	This finding, for the first time, highlights the essential non-metabolic functions of PKM2 by a dual role that is essential in EGFR-promoted ß-catenin transactivation, tumor cell proliferation, and tumorigenesis.	info:doi/10.5732/cjc.013.10228#cont:74	
PRDX1 ---> cell proliferation	Regulation	In addition PRDX1 is involved in cell differentiation and proliferation, apoptosis and innate immunity.	info:pmid/17913528#abs:2	
PRDX1 ---> cell proliferation	Regulation	This indicates that OSF-3 plays an intrinsic role in the proliferation and/or differentiation of bone cells.	info:pmid/8089076#abs:5	
PRDX1 ---> cell proliferation	Regulation	Deficiency of Prx I impaired cell proliferation and anchorage-independent growth.	info:pmid/23186333#cont:171	
PRDX1 ---> cell proliferation	Regulation	This indicates that Prx-I plays a role in cell proliferation, which is coincident with our previous result.	info:pmid/17976536#body:113	
PRDX1 ---> cell proliferation	Regulation	PrxI possesses not only antioxidant activity, but also regulatory activity in associated cell proliferation .	info:pmid/11065135#body:66	Brain {Organ urn:agi-ncimorgan:C1269537}
PRDX1 ---> cell proliferation	Regulation	This might explain, at least in part, why Prx I inhibition significantly increased p53 expressions and impaired cell proliferation.	info:pmid/20732753#body:102	
PRDX1 ---> cell proliferation	Regulation	peroxiredoxin 1 in turn can inhibit medial edge epithelial apoptosis, proliferation, and differentiation.	info:pmid/22561679#body:86	
PRDX1 ---> cell proliferation	Regulation	It has been suggested that Prx I regulates cell proliferation and apoptosis by its interaction with oncogene products such as c-Abl.	info:pmid/19566940#body:73	
PRDX1 ---> cell proliferation	Regulation	Additionally, peroxiredoxin-1 is one of the antioxidant enzymes and is involved in cellular proliferation and differentiation .	info:pmid/18996230#body:133	
PRDX1 ---> cell proliferation	Regulation	It seems that Prx I, PDGF-A, and PDGFR- a participate in transformation and proliferation, but they are not associated with invasion.	info:pmid/18992915#body:87	
PRDX1 ---> cell proliferation	Regulation	On the basis of the analysis of the cell cycle, Prx I expression very likely affects cell proliferation by regulating the cell cycle.	info:pmid/16414373#body:136	

PRDX1 ---> cell proliferation	Regulation	Prx I has also been suggested to be a tumor suppressor by regulating cell proliferation and transformation via direct interaction with the oncogenes c-Abl and c-Myc.	info:pmid/12960165#body:422	
PRDX1 ---> cell proliferation	Regulation	In the ventral and dorsal iris in all time points, peroxiredoxin 1, a protein that has been shown to play a role in proliferation and to be expressed in melanosomes, was upregulated (Table 3) [23,24].	info:pmid/23378727#cont:149	Dorsum {Organ urn:agi-ncimorgan:C0460009}
PRDX1 ---> cell proliferation	Regulation	At this point, there is no evidence that the secreted form of Peroxiredoxin-1 plays any role in proliferation and we plan to address this by supplementing the media of cells with a Peroxiredoxin-1 neutralizing antibody or purified Peroxiredoxin-1 protein.	info:pmid/21343469#cont:707	
PRDX1 ---> cell proliferation	Regulation	Prx I gene activation by the phorbol ester O-tetradecanoylphorbol-13-acetate, which primarily affects cellular proliferation and differentiation, is inhibited by the bacterial product lipopolysaccharide, which is a prototypical proinflammatory mediator.	info:pmid/18070609#body:182	
PRDX1 ---> cell proliferation	Regulation	Because proliferation-associated gene/NKEF-A was abundantly expressed in transformed and rapidly growing cells but was suppressed in quiescent cells, it was suggested that proliferation-associated gene/NKEF-A was important for cell proliferation.	info:pmid/8981042#body:9	
PRDX1 ---> cell proliferation	Regulation	Ectopic expression of HA-PrxI and -PrxII and oxidant-induced arrest Up-regulation of PrxI is thought to counteract the effects of enhanced oxidant production in tumor cells and thereby promote cell survival and proliferation (Chang et al., 2005; Park et al., 2006).	info:pmid/17145963#body:199	
PRDX1 ---> cell proliferation	Regulation	Among the 27 candidate immunoglobulin G-binding proteins obtained, guanine nucleotide binding protein (G protein), beta polypeptide 2-like 1 (GNB2L1; RACK1) and RAN were involved in cellular growth, and PRDX1, an antioxidase, participated in cellular antioxidate and proliferation.	info:pmid/24309932#cont:25	
PRDX1 ---> cell proliferation	Regulation	For example, overexpression of Prx I facilitates cell growth and proliferation by protecting them from oxidant-induced cell death, and Prx III is required for Myc-mediated rat fibroblast transformation and proliferation of breast cancer MCF7 cells (20).	info:pmid/21487000#cont:231	
HNRNPK --+>	Regulation	We found that SET and/or hnRNPK protein accumulation increased	info:pmid/24508256#	

cell proliferation		cellular proliferation.	abs:5	
HNRNPK --+> cell proliferation	Regulation	Finally, overexpression of hnRNP K in breast cancer cells significantly increased target c-myc promoter activity and c-Myc protein, hnRNP K protein levels, and enhanced breast cancer cell proliferation and growth in an anchorage-independent manner.	info:pmid/11121407#abs:7	
HNRNPK --+> cell proliferation	Regulation	These data suggested that hnRNP K plays an important role in cell growth and proliferation.	info:pmid/20499280#cont:173	
HNRNPK --+> cell proliferation	Regulation	HnRNP K is involved in switching from proliferation to (neuronal) differentiation .	info:pmid/19410666#body:130	Spleen {Organ urn:agi-ncimorgan:C1278932}
HNRNPK --+> cell proliferation	Regulation	These results correlate well with a previous study demonstrating that downregulation of hnRNPK decreases cellular proliferation .	info:pmid/23170974#cont:254	
HNRNPK --+> cell proliferation	Regulation	In contrast, overexpression of heterogeneous nuclear ribonucleoprotein K enhances breast cancer cell proliferation [32].	info:pmid/21799787#cont:335	
HNRNPK --+> cell proliferation	Regulation	Furthermore, downregulation of heterogeneous nuclear ribonucleoprotein K significantly inhibits growth factor-independent proliferation and colony formation .	info:pmid/19520192#body:169	
HNRNPK --+> cell proliferation	Regulation	Interestingly, the hnRNPK is involved in the activation of the human c-Myc promoter and enhances cell proliferation and growth of breast cancer cells in an anchorage independent manner .	info:pmid/18424265#body:176	Breast {Organ urn:agi-ncimorgan:C0006141}
HNRNPK --+> cell proliferation	Regulation	These reports and our results suggest that hnRNP K may promote cell proliferation and have a negative effect against the promotion of differentiation in vivo.	info:pmid/15671036#body:345	Brain {Organ urn:agi-ncimorgan:C1269537}
HNRNPK --+> cell proliferation	Regulation	Likewise, expression of hnRNP K is upregulated in grade III breast cancer samples and appears to correlate with anchorage-independence and enhanced proliferation of breast cancer cells (Mandal et al., 2001).	info:pmid/12476304#body:145	
HNRNPK --+> cell proliferation	Regulation	Interestingly, the hnRNPk, which is up-regulated in the current study, is thought to be involved in the activation of the human c-Myc promoter and enhances cell proliferation and growth of BC	info:pmid/17996735#body:139	

		cells in an anchorage independent manner .		
HNRNPK --+> cell proliferation	Regulation	In breast cancer cells, overexpression of hnRNP K enhances cell proliferation and anchorage-independent growth (Mandal et al, 2001), and in several states of enhanced cell proliferation, increased expression of this protein has also been found (Ostrowski and Bomsztyk, 2003).	info:pmid/19401687#body:48	
HNRNPK --+> cell proliferation	Regulation	Additionally, there is evidence to show that hnRNP K upregulation is a cause rather than an effect of proliferation; overexpression of hnRNP K in breast cancer cells significantly increases cell proliferation and growth in an anchorage-independent manner (Mandal et al, 2001).	info:pmid/16953238#body:223	
HNRNPK --+> cell proliferation	Regulation	More importantly, hnRNP K up-regulates multiple downstream genes, including eIF4E and c-Myc, through transcriptional and post-transcriptional regulation, increasing cell proliferation, anti-apoptosis, and metastasis, and thereby conferring a tumorigenic phenotype on cancer cells.	info:pmid/22321252#body:8	
HNRNPK --+> cell proliferation	Regulation	We found that hnRNP K not only functions as a translational regulator of human reticulocyte 15-lipoxygenase mRNA (Fig. 2) but also represses expression of c-Src (Figs. 5 and 6), an important regulator of cell cycle control, proliferation, and differentiation (44, 45).	info:pmid/18441016#body:288	
HNRNPK --+> cell proliferation	Regulation	We also explored the functional significance of hnRNPK and found that hnRNPK knockdown inhibited proliferation, whereas hnRNPK overexpression rescued the effects of T-cell leukaemia 1 knockdown in hepatocellular carcinoma and HCT116 cells (see online supplementary figure S7).	info:pmid/24352616#cont:302	
HNRNPK --+> cell proliferation	Regulation	More importantly, hnRNP K up-regulates multiple downstream genes, including eIF4E and c-Myc, through transcriptional and post-transcriptional regulation, increasing cell proliferation, anti-apoptosis, and metastasis, and thereby conferring a tumorigenic phenotype on cancer cells.	info:doi/10.1016/j.oraloncology.2012.01.005#body:8	
HNRNPA1 ---> cell proliferation	Regulation	Down regulation of hnRNP A1 expression by RNA interference inhibits the proliferation and migration of cancerous HepG2 cells, while overexpression of hnRNP A1 in normal HL-7702 cells	info:pmid/23062008#abs:5	

		increased the proliferation and migration of the cells.		
HNRNPA1 ---> cell proliferation	Regulation	The over-expression of hnRNP A1 could contribute to the maintenance of telomere repeats in cancer cells and allow enhanced cell proliferation.	info:pmid/16513142#body:128	
HNRNPA1 ---> cell proliferation	Regulation	The shuttling of hnRNP A1 is subject to regulation and is thought to play a role in cell proliferation, survival, and differentiation of normal and transformed cells (10).	info:pmid/15738418#body:49	
HNRNPA1 ---> cell proliferation	Regulation	Moreover, hnRNPA1 is associated with apoptosis resistance (Patry et al., 2003[Go]) and increased proliferation (He et al., 2005[Go]) in cancer cells, two important features of cancer cells.	info:pmid/17652158#body:269	
HNRNPA1 ---> cell proliferation	Regulation	Evidence of a direct involvement of the Heterogeneous nuclear ribonucleoproteins in proliferation was provided by RNAi, reduction of hnRNP A1 and A2 significantly reduced the proliferation rate of Colo 16 cells .	info:pmid/16378690#body:218	
HNRNPA1 ---> cell proliferation	Regulation	In addition, hnRNP A1 mRNA export activity is required for proliferation, survival, and tumorigenesis of acute phase Chronic myelogenous leukemia blasts and BCR/ABL + myeloid precursor cell lines ( ).	info:pmid/16286244#body:14	
HNRNPA1 ---> cell proliferation	Regulation	As for the genes in the cell growth and proliferation category, the upregulation of SEL1L, HNRNPA1, GAS5, DDIT4/REDD1, MTSS1, ARID4A, ANK3, ARMET, GNE and PTPRK, either reduce or inhibit cell proliferation, and these genes are expressed when growth is retarded ( ).	info:pmid/22101062#body:101	
HNRNPA1 ---> cell proliferation	Regulation	The shuttling activity of hnRNP A1 has been proposed to play a role in cell proliferation, survival, and differentiation of normal and transformed cells . hnRNP A1 is also implicated in postsplicing activities, such as mRNA export and cap-dependent and internal ribosome entry site-mediated translation .	info:pmid/22227431#body:16	
YWHAG --+> cell proliferation	Regulation	14-3-3 gamma is stimulated by IL-3 and promotes cell proliferation.	info:pmid/19124748#title:1	
YWHAG --+> cell proliferation	Regulation	Recent studies indicated that expression of 14-3-3 ? promotes cell proliferation .	info:pmid/20870266#body:12	

YWHAG --+> cell proliferation	Regulation	Human 14-3-3 gamma protein results in abnormal cell proliferation in the developing eye of Drosophila melanogaster.	info:pmid/18194556#title:1	Eye {Organ urn:agi-ncimorgan:C1550636}
YWHAG --+> cell proliferation	Regulation	In addition, 14-3-3? induces resistance to apoptotic programmed cell death and promotes proliferation of IL-3-dependent Ba/F3 cells .	info:pmid/22658894#body:68	Lung {Organ urn:agi-ncimorgan:C1278908}
YWHAG --+> cell proliferation	Regulation	Recent studies demonstrated that up-regulation of 14-3-3? promotes cell survival and proliferation through activation of distinct signal pathways in hematopoietic progenitor cells .	info:pmid/23500129#body:192	
YWHAG --+> cell proliferation	Regulation	receptor 4, and 14-3-3 protein gamma, are involved in promotion of inflammation, ROS production, cell proliferation, cardiovascular remodeling, neurodegeneration and tumor growth in vitro and in vivo through various mechanisms as reported previously (Table S1).	info:pmid/24386293#cont:303	Aorta {Organ urn:agi-ncimorgan:C1278934}
YWHAG --+> cell proliferation	Regulation	In astrocyte cell cultures 14-3-3? expression changed with time in contrast to that in cerebellar neuronal cultures . 14-3-3? is potentially involved in structural dynamics and proliferation via binding to phosphorylated GFAP and actin .	info:pmid/21920445#body:39	Cerebellum {Organ urn:agi-ncimorgan:C1268981}
OAT ---> cell proliferation	Regulation	In mammals, Ornithine d-aminotransferase has been shown to modulate cell proliferation by regulating intracellular ornithine concentrations.	info:pmid/20673832#body:204	
CFL1 ---> cell proliferation	Regulation	Cofilin-1 plays roles in cell migration, proliferation and phagocytosis.	info:pmid/24023293#abs:6	
CFL1 ---> cell proliferation	Regulation	In addition, n-cofilin is required for neuronal precursor cell proliferation and scattering.	info:pmid/15649475#abs:7	
CFL1 ---> cell proliferation	Regulation	Conversely, up-regulation of CFL1 in NSCs increased proliferation, adhesion, invasion and expression of the markers but reduced apoptosis.	info:pmid/20713416#abs:10	Ovary {Organ urn:agi-ncimorgan:CL384202}
CFL1 ---> cell proliferation	Regulation	The activity of cofilin, an actin-remodeling protein, is required for T lymphocyte activation with regard to formation of the immunological synapse, cytokine production, and proliferation.	info:pmid/16424196#abs:1	
CFL1 ---> cell proliferation	Regulation	Thirteen proteins from several pathways (nucleoside diphosphate kinase A, stathmin, valosin-containing protein, annexin A1, dihydropyrimidinase-related protein-3, DJ-1 protein, glutathione S-	info:pmid/19156760#abs:5	

		transferase P, lamin A/C, fascin, cofilin, vimentin, vinculin, and moesin) were differentially expressed and most have been shown to play a role in differentiation, migration, invasion, proliferation, apoptosis, drug resistance, or oncogenesis.		
CFL1 ---> cell proliferation	Regulation	Cell proliferation and cytokinesis is impaired in n-cofilin null macrophages.	info:pmid/22558315#cont:167	
CFL1 ---> cell proliferation	Regulation	These results suggest that cofilin phosphorylation regulates both cell proliferation and axon growth.	info:pmid/15572110#body:69	
CFL1 ---> cell proliferation	Regulation	Strikingly, depletion of CapZ and Cofilin clearly prevented contact inhibition of proliferation in center cells.	info:pmid/23954413#body:90	
CFL1 ---> cell proliferation	Regulation	Cofilin-1 plays roles in cell proliferation, phagocytosis, chemotactic movement and macropinocytosis (18,19).	info:pmid/21894436#cont:119	Pancreas {Organ urn:agi-ncimorgan:C1278931}
CFL1 ---> cell proliferation	Regulation	The phosphorylation of cofilin may then promote actin polymerisation, leading to proliferation and cytoskeletal rearrangement.	info:pmid/24370186#body:116	Periodontal Ligament {Organ urn:agi-ncimorgan:C0031093}
CFL1 ---> cell proliferation	Regulation	Although the mechanisms by which Cofilin-1 may promote uncontrolled cell proliferation are still poorly understood it may possibly be due to an impairment of DNA repair capacity .	info:pmid/23770296#body:274	
CFL1 ---> cell proliferation	Regulation	Nox1 plays a role in vascular smooth muscle cell migration, proliferation, and extracellular matrix production, effects that are mediated by cofilin (219).	info:pmid/23600794#cont:94	
CFL1 ---> cell proliferation	Regulation	However, the genetic deletion of Cofilin in the nervous system reduces neuronal cell proliferation and migration but not neurite formation ( ).	info:pmid/23259946#body:23	Nervous system {Organ urn:agi-ncimorgan:C0027763}
CFL1 ---> cell proliferation	Regulation	Mutations in actin-depolymerizing factors/cofilins from different species have been associated with lethality (; ;), arrest in cell proliferation, and disorganized actin cytoskeletons ( ).	info:pmid/16360805#body:4	
CFL1 ---> cell proliferation	Regulation	Hence, ILK/ß-parvin/cofilin signaling contributed critically to the abundant filopodium-like protrusion display and rapid cell proliferation in the 3D "Matrigel on-top" cultures of multiple colonization-competent carcinoma cell types.	info:pmid/24035453#body:100	
CFL1 ---> cell proliferation	Regulation	While Dstn2/2 brains have a normal gross morphology, conditional deletion of Cfl1 in neuronal cells causes excessive differentiation,	info:pmid/21060807#cont:28	Brain {Organ urn:agi-ncimorgan:C1269537}

		changes in cell proliferation, and migration defects, resulting in a lissencephaly phenotype [5].		
CFL1 ---> cell proliferation	Regulation	The knockdown of cofilin with small interference RNA attenuated the EGF-induced migration and proliferation of T24 cells.	info:doi/10.1016/j.juro.2010.02.645#body:16	
YWHAZ --+> cell proliferation	Regulation	It is concluded that 14-3-3? plays an important role in proliferation of AML cells and associates with BCL-2 and MCL-1 expression.	info:pmid/23998576#abs:13	
YWHAZ --+> cell proliferation	Regulation	Knockdown of YWHAZ expression using several specific siRNAs inhibited the proliferation, migration, and invasion of YWHAZ-overexpressing gastric cancer cells.	info:pmid/23422756#abs:7	Veins {Organ urn:agi-ncimorgan:C0042449}
YWHAZ --+> cell proliferation	Regulation	Using Co-immunoprecipitation, we demonstrated that 14-3-3zeta protein binds to NFkappaB, beta-catenin and Bcl-2, suggesting its involvement in cellular signaling, leading to proliferation of oral cancer cells.	info:pmid/17764575#abs:9	
YWHAZ --+> cell proliferation	Regulation	Depletion of 14-3-3? markedly increased apoptosis, reduced proliferation and receptor tyrosine kinase (HER2 and EGFR) signaling, and, importantly, reversed endocrine resistance.	info:pmid/21707964#abs:8	Endocrine system {Organ urn:agi-ncimorgan:C1280975}
YWHAZ --+> cell proliferation	Regulation	Taken together, our findings show that overexpression of 14-3-3? has a causal role in mammary tumorigenesis and progression, acting through miR-221 in cooperation with known oncogenic events to drive neoplastic cell proliferation.	info:pmid/24197133#abs:8	
YWHAZ --+> cell proliferation	Regulation	14-3-3?, a novel androgen-responsive gene, is upregulated in prostate cancer and promotes prostate cancer cell proliferation and survival.	info:pmid/22904106#title:1	
YWHAZ --+> cell proliferation	Regulation	To test the hypothesis that silencing of 14-3-3? suppresses hepatocellular carcinoma cell proliferation, we assessed expression of PCNA in si14-3-3?-transfected HepG2 cells during the first 4 days following transfection.	info:pmid/21334806#body:76	
YWHAZ --+> cell proliferation	Regulation	Overexpression of miR-451 and downregulation of 14-3-3? expression in endocrine-resistant cells restored the effectiveness of tamoxifen to decrease cell proliferation, increase apoptosis and reduce activation of EGFR/HER2 signaling.	info:pmid/23344024#cont:290	Endocrine system {Organ urn:agi-ncimorgan:C1280975}
YWHAZ --+>	Regulation	14-3-3z promotes breast cancer cell proliferation, survival and	info:pmid/21666713#c	Endocrine system

cell proliferation		receptor tyrosine kinase (EGFR, HER2) activation, and protein kinase signaling while suppressing apoptosis, all of which support the progression to endocrine resistance.	ont:483	{Organ urn:agi-ncimorgan:C1280975}
YWHAZ --+> cell proliferation	Regulation	In this study, a total of 71.7% of Pancreatic adenocarcinoma showed an advanced stage of disease with high expression of 14-3-3 zeta. 14-3-3 zeta has been proposed to be directly involved in proliferation and in cellular transformation . 14-3-3 zeta inhibits specifically stress-induced p38 and JNK signaling and the triggering of the apoptotic response.	info:pmid/24629487#body:85	Pancreas {Organ urn:agi-ncimorgan:C1278931}
YWHAZ --+> cell proliferation	Regulation	Moreover, the up-regulation of prohibitin, a repressor of E2F-mediated DNA synthesis and cellular proliferation , may also contribute to the observed berberine-induced loss of viability in these cells, as may the observed down-regulation of 14-3-3 zeta, a positive regulator of cell proliferation, whose overexpression in mammary epithelial cells has been shown to disrupt apoptotic signaling by down-regulating p53 .	info:pmid/22522123#body:156	
YWHAZ --+> cell proliferation	Regulation	Moreover, the up-regulation of prohibitin, a repressor of E2F-mediated DNA synthesis and cellular proliferation , may also contribute to the observed berberine-induced loss of viability in these cells, as may the observed down-regulation of 14-3-3 zeta, a positive regulator of cell proliferation, whose overexpression in mammary epithelial cells has been shown to disrupt apoptotic signaling by down-regulating p53 .	info:doi/10.1016/j.jprot.2012.03.010#body:156	
PRDX2 ---> cell proliferation	Regulation	These findings indicate that peroxiredoxin2 is involved in the proliferation of androgen receptor-expressing prostate cancer cells by modulating androgen receptor activity.	info:pmid/21539911#abs:9	
PRDX2 ---> cell proliferation	Regulation	Prx II deficiency results in increased production of H2O2, enhanced activation of Platelet-derived growth factor receptor and phospholipase Cgamma1, and subsequently increased cell proliferation and migration in response to Platelet-derived growth factor.	info:pmid/15902258#abs:4	
PRDX2 ---> cell proliferation	Regulation	Prx II deletion enhances concanavalin A -induced splenocyte proliferation and mixed lymphocyte reaction activity of bone marrow-derived CD11c-positive dendritic cells to stimulate	info:pmid/16290204#abs:8	Bone Marrow {Organ urn:agi-ncimorgan:C0005953}

		recipient splenocytes.		
PRDX2 ---> cell proliferation	Regulation	In addition, the administration of PEP-1-SOD1 and/or PEP-1-peroxiredoxin-2 ameliorated D-galactose-induced reductions of cell proliferation and neuroblast differentiation in the dentate gyrus and significantly reduced D-galactose-induced lipid peroxidation in the hippocampus.	info:pmid/23892988#abs:7	
PRDX2 ---> cell proliferation	Regulation	Rapamycin, AZD8055, and Torin-1 inhibit proliferation of MTT cells in vitro.	info:pmid/23307788#cont:125	
PRDX2 ---> cell proliferation	Regulation	These data suggest that knocking down Prx II in C6 alters cell proliferation.	info:pmid/18718523#body:236	
PRDX2 ---> cell proliferation	Regulation	Compared with rapamycin, PP242 and Torin1 impaired the proliferation of primary cells to a far greater degree [136,137].	info:pmid/20812900#cont:277	
PRDX2 ---> cell proliferation	Regulation	Peroxiredoxin 2 plays an important role in regulating cell proliferation and has anti-apoptotic properties .	info:pmid/18503785#body:149	
PRDX2 ---> cell proliferation	Regulation	As discussed above, Prx II negatively regulates growth factor signaling and, thus, also negatively regulates cell proliferation.	info:pmid/16290020#body:99	
PRDX2 ---> cell proliferation	Regulation	Prx II deficiency results in increased production of H2O2, enhanced activation of the platelet-derived growth factor receptor and phospholipase C–g1, and ...	info:pmid/20919930#cont:333	
PRDX2 ---> cell proliferation	Regulation	Previous studies have suggested that Prdx2 can regulate many cellular functions including cell proliferation and differentiation .	info:pmid/24125860#body:139	Colorectal Region {Organ urn:agi-ncimorgan:C1711309}
PRDX2 ---> cell proliferation	Regulation	The Prdx2 suppression by small interfering RNA inhibited proliferation of the isolated GCs and increased the apoptosis ratio.	info:pmid/21248284#cont:269	Ovarian Follicle {Organ urn:agi-ncimorgan:C1283799}
PRDX2 ---> cell proliferation	Regulation	Prx II participates in regulation of cellular functions such as cell proliferation and differentiation, and protects a number of specific proteins from oxidative damage .	info:pmid/22749995#body:5	
PRDX2 ---> cell proliferation	Regulation	PRDX2 suppression by small interfering RNA in granulosa cells of mouse ovary inhibits cell proliferation by augmenting H2O2 production (46).	info:pmid/22989627#cont:315	Ovary {Organ urn:agi-ncimorgan:CL384202}
PRDX2 ---> cell proliferation	Regulation	The proliferation of MNNG/HOS cells was significantly reduced at 96h after transfection with si-PRDX2-3, while that of MG63 cells was significantly reduced at 72h.	info:pmid/23911960#body:145	

PRDX2 ---> cell proliferation	Regulation	The Prx II knockdown enhanced the proliferation and chemotactic migration of human aortic SMCs in response to platelet-derived growth factor stimulation, which were attenuated by the gliotoxin pretreatment in a dose-dependent manner (Figure 5A).	info:pmid/23820076#cont:629	Aorta {Organ urn:agi-ncimorgan:C1278934}
PRDX2 ---> cell proliferation	Regulation	Peroxiredoxin 2 deficiency can result in increased H2O2 production, enhanced activation of platelet-driven growth factor receptor, and subsequently increased cell proliferation and migration in the vascular remodelling process (28).	info:pmid/17982696#cont:283	Blood Vessels {Organ urn:agi-ncimorgan:C0005847}
PRDX2 ---> cell proliferation	Regulation	For example, Choi et al. (2005[Go]) have found that the deficiency of peroxiredoxin II, a cellular peroxidase, results in increased production of H2O2, enhanced activation of PDGF receptor, and subsequently increased cell proliferation and migration in response to PDGF (Choi et al., 2005[Go]).	info:pmid/16391241#body:321	
PRDX2 ---> cell proliferation	Regulation	The physiological significance of these events was recently supported by the demonstration that depletion of reactive oxygen species-scavenging peroxiredoxin II enhances platelet-derived growth factor-receptor-induced proliferation, and concomitantly increases platelet-derived growth factor ß-receptor phosphorylation and reduces protein tyrosine phosphatase activity.23 .	info:pmid/16990553#body:217	
ENO1 ---> cell proliferation	Regulation	This study suggested that exogenous expression of MBP-1 induces cell death in fibroblasts by blocking cell proliferation.	info:pmid/8519685#abs:7	
ENO1 ---> cell proliferation	Regulation	We found that cell proliferation was inhibited by MBP-1 overexpression in human stomach adenocarcinoma SC-M1 cells.	info:pmid/19846662#abs:5	
ENO1 ---> cell proliferation	Regulation	Overexpressed ENO1 not only restored cell proliferation and cell-cycle progression, but also antagonized the regulation of NESG1 to cell-cycle regulators p21 and CCNA1 expression as well as induced the expression of C-Myc, pRB, and E2F1 in NESG1-ovexpressed nasopharyngeal carcinoma cells.	info:pmid/22997098#abs:5	
ENO1 ---> cell proliferation	Regulation	ENO1 overexpression promoted cell proliferation, migration, invasion and tumourigenesis.	info:pmid/20435467#body:151	Pancreas {Organ urn:agi-ncimorgan:C1278931}
ENO1 ---> cell proliferation	Regulation	ENO1, PIN1, GLUL and BCL2L1 are involved in mitogenesis, proliferation and apoptosis.	info:pmid/16466929#body:165	Brain {Organ urn:agi-ncimorgan:C1269537}

ENO1 ---> cell proliferation	Regulation	Furthermore, exogenous a-enolase expression promoted cell proliferation, migration, invasion, and tumorogenesis [105].	info:pmid/23118496#cont:198	
ENO1 ---> cell proliferation	Regulation	Recently, we have shown that exogenous expression of MBP-1 in androgen-independent prostate cancer cells inhibits cell proliferation (23).	info:pmid/15805119#body:87	
ENO1 ---> cell proliferation	Regulation	In addition, the results indicated that enolase-1 promoted cell proliferation, although the effect was not evident under normoxic conditions.	info:pmid/23381546#cont:315	
ENO1 ---> cell proliferation	Regulation	Recently, we determined that both expression and function of MBP-1 are regulated by alterations in exogenous glucose concentrations and correspond to changes in cell proliferation and lactate production [26].	info:pmid/20412594#body:255	
ENO1 ---> cell proliferation	Regulation	Thus, one possibility may be that the mutant SEDLINs that are associated with SEDT and lead to a loss of interaction with MBP1 may disrupt the tight control between proliferation and apoptosis in endochondral ossification.	info:pmid/20498720#body:230	
ENO1 ---> cell proliferation	Regulation	Exogenous MBP-1 expression inhibits the growth of breast tumors in nude mice , induces cell death in neuroblastoma cells , suppresses proliferation in non-small-cell lung cancer cells , and induces G0–G1 growth arrest in chronic myeloid leukemia cells .	info:pmid/23421821#cont:19	
ENO1 ---> cell proliferation	Regulation	In this study, expression of TPI-1 and ENO1, which enhances cancer cell survival and proliferation, were higher in metastatic tumors than in primary tumors, and TAGLN2, which suppresses invasion of cancer cells, was lower in metastatic tumors than in primary tumors.	info:pmid/23504277#cont:380	Cardiovascular system {Organ urn:agi-ncimorgan:C1269562}
ENO1 ---> cell proliferation	Regulation	In conclusion, results to date suggest that the differential expression of 14-3-3 protein a/ß, Cullin homolog 3, a-enolase and ezrin in H. pylori infection may play an important role in gastric carcinogenesis, including cell proliferation and cell adhesion, and that they may be induced by reactive oxygen species-mediated cell signaling.	info:pmid/15147740#body:139	Gastric mucosa {Organ urn:agi-ncimorgan:C0017136}
PGK1 ---> cell proliferation	Regulation	PGK1 modulates U251 cell proliferation.	info:pmid/24284928#cont:116	
PGK1 ---> cell	Regulation	Besides PGK1 gene, concomitant up-regulation of FGF1, FGF2, IL6,	info:pmid/23684136#	

proliferation		MUC1, and platelet-derived growth factor alpha polypeptide genes in F28/KMUH Cancer-associated fibroblasts may also be the explanations for Hepatocellular carcinoma cells to promote proliferation of F28/KMUH Cancer-associated fibroblasts and thus promote cancer progression.	body:79	
PGK1 ---> cell proliferation	Regulation	Besides PGK1 gene, concomitant up-regulation of FGF1, FGF2, IL6, MUC1, and platelet-derived growth factor alpha polypeptide genes in F28/KMUH Cancer-associated fibroblasts may also be the explanations for Hepatocellular carcinoma cells to promote proliferation of F28/KMUH Cancer-associated fibroblasts and thus promote cancer progression.	info:doi/10.1016/j.kjms.2012.08.012#body:79	
YWHAE ---> cell proliferation	Regulation	14-3-3epsilon regulates a wide range of biological processes, including cell cycle control, proliferation, and apoptosis, and plays a significant role in neurogenesis and the formation of malignant tumours.	info:pmid/20565895#abs:1	
YWHAE ---> cell proliferation	Regulation	YWHAE is involved in neuronal migration, proliferation, and cognitive impairment ( ).	info:pmid/23892282#body:169	Prosencephalon {Organ urn:agi-ncimorgan:C0085140}
PSMB6 --+> cell proliferation	Regulation	Knockdown of PSMB6 using siRNA also prevented hypoxia-induced proliferation.	info:pmid/23844134#abs:10	Pulmonary artery {Organ urn:agi-ncimorgan:C0034052}
ATP5A1 ---> cell proliferation	Regulation	An antibody against the ATP synthase a-subunit inhibited proliferation, migration and invasion in these breast cancer cells but not that of a non-tumor derived breast cell line.	info:pmid/22152132#abs:10	
ATP5A1 ---> cell proliferation	Regulation	The identified copy number amplification genes, such as ZNF521, RNF138, RAB12, ATP5A1, PTPN2 and CTAGE1, are involved in multiple cellular processes, including transcription, proliferation, differentiation, migration and immunity.	info:pmid/23678296#cont:270	Fetus {Organ urn:agi-ncimorgan:C1305737}
ATP5A1 ---> cell proliferation	Regulation	Mitochondrial ATP synthase inhibitors antagonize 5-Fluorouracil-induced suppression of cell proliferation.	info:pmid/15833846#body:183	
ATP5A1 ---> cell proliferation	Regulation	These findings indicate that the modulation of mitochondrial ATP synthase activity via expression of mutant or wild-type MTATP6 from the nucleus can affect cell proliferation and override the	info:pmid/15753359#body:256	

		effect of the mitochondrial DNA genotype.		
CKB ---> cell proliferation	Regulation	It was found that Creatine kinase B knockdown inhibited Skov3 cell proliferation and induced apoptosis under hypoxia or hypoglycemia conditions.	info:pmid/23416112#abs:5	
CKB ---> cell proliferation	Regulation	Furthermore, overexpression of CFP-tagged wild-type Creatine kinase brain in Caco-2 colon cancer cells dramatically increased the number of cells in G2/M but had little effect on cell proliferation.	info:pmid/21308735#abs:7	
