# Supplementary material for: Deciphering the Role of Emx1 in Neurogenesis: A Neuroproteomics Approach
Source: Front Mol Neurosci. 2016 Oct 17;9:98. doi: 10.3389/fnmol.2016.00098 (PMC5065984; doi:10.3389/fnmol.2016.00098)
Supplement: Supplementary file 4 [file Table4.PDF]

Table 4a: Cell Migration Cluster

Entity Table:

Name	Type	Description	Connectivity	Local Connectivity	Indegree
ENO1	Protein	enolase 1, (alpha)	336	1	0
PGK1	Protein	phosphoglycerate kinase 1	155	1	0
PKM	Protein	pyruvate kinase, muscle	346	1	0
ATP5A1	Protein	ATP synthase, H+ transporting, mitochondrial F1 complex, alpha subunit 1, cardiac muscle	139	1	0
YWHAB	Protein	tyrosine 3-monooxygenase/tryptophan 5-monooxygenase activation protein, beta polypeptide	251	1	0
TUBA1A	Protein	tubulin, alpha 1a	54	1	0
HSPD1	Protein	heat shock 60kDa protein 1 (chaperonin)	988	1	0
CFL1	Protein	cofilin 1 (non-muscle)	920	1	0
HNRNPK	Protein	heterogeneous nuclear ribonucleoprotein K	412	1	0
PEBP1	Protein	phosphatidylethanolamine binding protein 1	384	1	0
PRDX1	Protein	peroxiredoxin 1	388	1	0
YWHAZ	Protein	tyrosine 3-monooxygenase/tryptophan 5-monooxygenase activation protein, zeta polypeptide	557	1	0
YWHAE	Protein	tyrosine 3-monooxygenase/tryptophan 5-monooxygenase activation protein, epsilon polypeptide	328	1	0
CKB	Protein	creatine kinase, brain	158	1	0
cell migration	Cell Process		3904	14	14

Table 4b: Cell Migration Cluster

Relationship Table:

Relation	Type	Sentence	TextRef	Connectivity	# of References	Organ
YWHAB ---> cell migration	Regulation	14-3-3-beta expression influences cell migration and invasion, Forced overexpression of 14-3-3ß induced Hepatocellular carcinoma cell migration and increased cell proliferation, as well as tumor growth.	info:pmid/22925547#cont:162, info:pmid/21967815#body:146	2	2	
HSPD1 ---> cell migration	Regulation	In all cell lines, knockdown of Hsp60 or GANAB and silencing of Gp96 or Grp78 considerably enhanced or reduced cell migration and invasion, respectively., Recently, H. pylori Heat shock protein 60 has been reported to promote cancer development by inducing chronic inflammation and promoting tumor cell migration., PD 98059 at 5 µM partially reversed the cell migration stimulated by hsp60., Human HSP60 has been found to downregulate T-cell migration by interacting with TLR2 and to inhibit the secretion of proinflammatory cytokines by activated T cells [21,22]. <more data available...>	info:pmid/21642380#abs:8, info:pmid/20580690#abs:2, info:pmid/15194479#body:134, info:pmid/23300563#cont:31, info:pmid/16767222#body:61	2	5	
CFL1 --+> cell migration	Regulation	Cofilin-1 plays roles in cell migration, proliferation and phagocytosis., It is well established that unphosphorylated (active) cofilin is a required to drive cell migration., Cofilin is an important regulator of actin polymerization, cell migration, and chemotaxis., So it remains unclear whether cofilin phosphorylation plays a promoting or inhibitory role during cell migration., Cofilin knockdown impaired Aurora-A-driven cell migration and protrusion of the cell membrane., The cfl1	info:pmid/24023293#abs:6, info:pmid/18424072#abs:8, info:pmid/21504724#abs:1, info:pmid/21205790#abs:3, info:pmid/21045147#abs:5, info:pmid/21203473#abs:7, info:pmid/16651380#abs:3, info:pmid/23112838#abs:12, info:pmid/24209839#abs:9, info:pmid/18928553#abs:6 <more data available...>	2	85	Colon {Organ urn:agi-ncimorgan:C1281569}, airway {Organ urn:agi-ncimorgan:C0458827}, Immune system

		morpholino oligos-induced cell migration defect was found to be cell-autonomous in cell transplantation assays. <more data available...>				{Organ urn:agi-ncimorgan:C0020962}, Ectoderm {Organ urn:agi-ncimorgan:C0013574}, Mesenchyme {Organ urn:agi-ncimorgan:C0162415}, Cerebral cortex {Organ urn:agi-ncimorgan:C0007776}
PEBP1 --+> cell migration	Regulation	Small interfering RNA-mediated silencing of RKIP expression also reduces cell migration rate., RKIP augmented Hepatic stellate cell migration and enhanced wound closure., As a modulator of key signaling pathways, RKIP affects various cellular processes including cell differentiation, the cell cycle, apoptosis and cell migration., Inhibition of RKIP by small hairpin RNA transfection significantly decreased the inhibitory effect of Gemifloxacin on the nuclear factor ?B/Snail pathway and also inhibited cell migration and invasion. <more data available...>	info:pmid/16183022#abs:5, info:pmid/19323783#abs:14, info:pmid/18781826#abs:4, info:pmid/24005829#abs:10, info:pmid/17030190#body:133, info:pmid/19551145#body:265, info:pmid/20855151#body:16, info:pmid/21917533#body:4, info:pmid/22227918#cont:639, info:pmid/23232914#cont:16 <more data available...>	2	13	Intestines {Organ urn:agi-ncimorgan:C0021853}, tibialis anterior muscle {Organ urn:agi-organ:tibialis%20anterior%20muscle}, Prostate {Organ urn:agi-ncimorgan:C1278980}

YWHAE ---> cell migration	Regulation	Knockdown with specific siRNA abolished 14-3-3e-induced cell migration and epithelial-mesenchymal transition., Moreover, overexpression of 14-3-3epsilon resulted in the inhibition of cell migration induced by MAPK-activated protein kinase 5 overexpression or TNFalpha treatment., The authors also demonstrated that 14-3-3e prevented MK5-mediated F-actin reorganization and cell migration., 14-3-3-epsilon inhibits cell migration by blocking HSP 27 phosphorylation, which is required for F-actin polymerization.33 <more data available...>	info:pmid/23483955#abs:5, info:pmid/17728103#abs:4, info:pmid/19166925#body:88, info:pmid/22925547#cont:234, info:pmid/20565895#body:259, info:pmid/15167810#body:271	2	6	Cerebral cortex {Organ urn:agi-ncimorgan:C 0007776}
PGK1 --+> cell migration	Regulation	These results suggest that MIG-10 and ABI-1 interact physically to promote cell migration and process outgrowth in vivo., Grb7 is an Src homology 2-containing and pleckstrin homology domain-containing molecule, which shares significant homology with the Caenorhabditis elegans gene for Mig-10 involved in cell migration during embryogenesis., PGK1 modulates U251 cell migration ability., Grb7, Grb10 and Grb14 form a protein family that is phylogenetically related to the Caenorhabditis elegans Mig10 protein, which is involved in the regulation of embryonic neural cell migration . <more data available...>	info:pmid/23022657#abs:11, info:pmid/10446223#abs:2, info:pmid/24284928#cont:145, info:pmid/23743201#body:2, info:pmid/22451697#cont:173, info:pmid/19473962#body:44	2	6	
YWHAZ --+> cell migration	Regulation	The majority of the TGF-beta-induced proteins (such as tropomyosins, filamin A, B, & C, integrin-beta1, heat shock protein27, transglutaminase2, cofilin, 14-3-3 zeta, ezrin-radixin-moesin) are involved in the regulation of cell migration, adhesion and invasion, suggesting the acquisition of a invasive phenotype., ErbB2 and 14-3-3zeta overexpression, respectively, increased cell migration and decreased cell adhesion, two prerequisites of tumor cell invasion. 14-3-3zeta overexpression reduced cell adhesion by	info:pmid/16674103#abs:5, info:pmid/19732720#abs:4, info:pmid/15037601#body:235, info:pmid/23763993#body:16, info:pmid/24238270#cont:955, info:pmid/22193627#cont:145, info:pmid/19401330#title:1, info:pmid/21868386#cont:115, info:pmid/24163432#cont:384, info:pmid/23889253#cont:252 <more data available...>	2	12	

		activating the TGF-beta/Smads pathway that led to ZFHX1B/SIP-1 upregulation, E-cadherin loss, and epithelial-mesenchymal transition. <more data available...>				
ENO1 --+> cell migration	Regulation	Therefore, development of novel therapeutic strategies, such as anti-Pancreatic cancer immunotherapy, is crucial. a-Enolase (ENO1) is an enzyme expressed on the surface of pancreatic cancer cells and is able to promote cell migration and cancer metastasis., Secreted ENO1 promotes prostate cancer cell migration via its plasminogen-binding domain, CK8 and a-enolase bind and activate plasminogen to promote cell migration and invasion (13, 14, 17)., Recent studies revealed that, in addition to its innate catalytic function, a-enolase plays an important role in other biological/pathological processes, such as myogenesis , tRNA transport , K+ channel regulation , and tumor cell migration .	info:pmid/23640603#abs:3, info:pmid/22734040#cont:226, info:pmid/20406904#body:108, info:pmid/24361255#body:7	2	4	Prostate {Organ urn:agi-ncimorgan:C1278980}, Neurosecretory Systems {Organ urn:agi-ncimorgan:C0229526}
HNRNPK --+> cell migration	Regulation	Involvement of MEK and ERK in hnRNP-K-induced cell migration., More recently, it was shown that hnRNP K (56), G3BP1 (41), and Sam68 regulate cell migration (3)., We found that the cytoplasmic localization of hnRNP-K may mediate its role in cell migration and metastasis., Heterogeneous ribonucleoprotein K has also been shown to be involved in cell migration; a process necessary for cancer metastasis., Recent work has shown that hnRNP K regulates antiapoptosis and cell migration, two biological functions that are critically related to cancer development.9,11 <more data available...>	info:pmid/23564449#cont:220, info:pmid/19762470#body:318, info:pmid/17483488#body:164, info:pmid/16953238#body:60, info:pmid/20224598#cont:296, info:pmid/22582387#cont:282	2	6	
TUBA1A ---> cell migration	Regulation	That is the case of genes such as GNB2L1, an anchor protein involved in adhesion and migration of human glioma cells [51], DPYSL2, a promoter of microtubule assembly and neuronal development [52], TUBA1A [53] or CFL, which controls cell migration and cell cycle progression	info:pmid/20735813#cont:411	2	1	

		[54,55].				
CKB --+> cell migration	Regulation	Our experimental verification of this obviously speculative model showed that CK-B with a membrane anchor could indeed facilitate lamellipodia formation and stimulated cell migration.	info:pmid/19333390#body:278	2	1	
ATP5A1 --+> cell migration	Regulation	The suppression of cell migration by the anti-ATP synthase a-subunit antibody was not limited to a specific ECM interaction.	info:pmid/22152132#cont:657	2	1	
PRDX1 ---> cell migration	Regulation	Knockdown of human peroxiredoxin 1 significantly inhibited TGF-ß1-induced epithelial-to-mesenchymal transition and cell migration, whereas human peroxiredoxin 1 overexpression enhanced TGF-ß1-induced epithelial-to-mesenchymal transition and cell migration., For example, peroxiredoxin I and II association with the platelet-derived growth factor receptor in vascular smooth muscle cells regulated platelet-derived growth factor– dependent signaling and cell migration.39	info:pmid/22475482#abs:5, info:pmid/21636804#cont:238	2	2	
PKM --+> cell migration	Regulation	While in undifferentiated gastric cancer cells that lack E-cadherin, PKM2 can enhance EGFR downstream signaling activation and promote cell migration and invasion.56, This result is the opposite of what was observed with the BGC823 and SGC7901 cells; in AGS cells, PKM2 came into play as a stimulus and promoted cell migration and invasion.	info:pmid/24131935#cont:109, info:pmid/23840737#cont:199	2	2	

Table 4c: Cell Migration Cluster

Reference Table:

Relation	Type	Sentence	TextRef	Organ
YWHAB ---> cell migration	Regulation	14-3-3-beta expression influences cell migration and invasion	info:pmid/22925547#cont:162	
YWHAB ---> cell migration	Regulation	Forced overexpression of 14-3-3ß induced Hepatocellular carcinoma cell migration and increased cell proliferation, as well as tumor growth.	info:pmid/21967815#body:146	
HSPD1 ---> cell migration	Regulation	In all cell lines, knockdown of Hsp60 or GANAB and silencing of Gp96 or Grp78 considerably enhanced or reduced cell migration and invasion, respectively.	info:pmid/21642380#abs:8	
HSPD1 ---> cell migration	Regulation	Recently, H. pylori Heat shock protein 60 has been reported to promote cancer development by inducing chronic inflammation and promoting tumor cell migration.	info:pmid/20580690#abs:2	
HSPD1 ---> cell migration	Regulation	PD 98059 at 5 µM partially reversed the cell migration stimulated by hsp60.	info:pmid/15194479#body:134	
HSPD1 ---> cell migration	Regulation	Human HSP60 has been found to downregulate T-cell migration by interacting with TLR2 and to inhibit the secretion of proinflammatory cytokines by activated T cells [21,22].	info:pmid/23300563#cont:31	
HSPD1 ---> cell migration	Regulation	Recently, we reported that the human 60-kDa heat shock (HSP60) molecule, via innate TLR2 signaling, can downregulate T cell migration (16) and inhibit the secretion of proinflammatory cytokines by activated T cells (17).	info:pmid/16767222#body:61	
CFL1 --+> cell migration	Regulation	Cofilin-1 plays roles in cell migration, proliferation and phagocytosis.	info:pmid/24023293#abs:6	
CFL1 --+> cell migration	Regulation	It is well established that unphosphorylated (active) cofilin is a required to drive cell migration.	info:pmid/18424072#abs:8	
CFL1 --+> cell migration	Regulation	Cofilin is an important regulator of actin polymerization, cell migration, and chemotaxis.	info:pmid/21504724#abs:1	
CFL1 --+> cell migration	Regulation	So it remains unclear whether cofilin phosphorylation plays a promoting or inhibitory role during cell migration.	info:pmid/21205790#abs:3	
CFL1 --+> cell migration	Regulation	Cofilin knockdown impaired Aurora-A-driven cell migration and protrusion of the cell membrane.	info:pmid/21045147#abs:5	
CFL1 --+> cell migration	Regulation	The cfl1 morpholino oligos-induced cell migration defect was found to be cell-autonomous in cell transplantation assays.	info:pmid/21203473#abs:7	
CFL1 --+> cell migration	Regulation	Cofilin, a LIMK1 substrate, is essential for the regulation of actin polymerization and depolymerization during cell migration.	info:pmid/16651380#abs:3	

CFL1 --+> cell migration	Regulation	Cofilin is an actin severing protein and regulates membrane ruffling, lamellipodia formation and cell migration.	info:pmid/23112838#abs: 12	
CFL1 --+> cell migration	Regulation	Our model supports a direct role for cofilin-mediated actin polymerization in stimulated cell migration, including chemotaxis and cancer invasion.	info:pmid/24209839#abs: 9	
CFL1 --+> cell migration	Regulation	HIV-1-mediated aberrant activation of cofilin may also lead to abnormalities in T cell migration and activation that could contribute to viral pathogenesis.	info:pmid/18928553#abs: 6	
CFL1 --+> cell migration	Regulation	Expression of the wild type or non-phosphorylatable cofilin (A3 mutant) increased melanoma cell migration on vitronectin and invasion through a reconstituted basement membrane.	info:pmid/16337627#abs: 6	
CFL1 --+> cell migration	Regulation	Our earlier studies have shown that deletion of the gene corresponding to Leishmania actin-depolymerizing protein (ADF/cofilin) adversely affects flagellum assembly, intracellular trafficking, and cell division.	info:pmid/22492507#abs: 2	
CFL1 --+> cell migration	Regulation	Expression of constitutively active- or inactive-forms of cofilin also inhibits human erythroleukaemia cell migration and phosphorylated cofilin is localized to the front protrusions of human erythroleukaemia cells.	info:pmid/15066125#abs: 5	
CFL1 --+> cell migration	Regulation	Moreover, ADF knockdown or cofilin knockdown increased the rate of cell migration and the time of lamellipodia protrusion but through different mechanisms: lamellipodia protrude more frequently in ADF knockdown cells and are more persistent in cofilin knockdown cells.	info:pmid/24093776#abs: 5	
CFL1 --+> cell migration	Regulation	Given the well-defined roles of CD44, phosphorylated AKT in apoptosis and cancer, these results indicate that CD44-induced cell migration is dependent on its complex formation with Lyn and its consequent regulation of AKT phosphorylation and cofilin expression.	info:pmid/17599831#abs: 12	Colon {Organ urn:agi-ncimorgan :C1281569 }
CFL1 --+> cell migration	Regulation	The majority of the TGF-beta-induced proteins (such as tropomyosins, filamin A, B, & C, integrin-beta1, heat shock protein27, transglutaminase2, cofilin, 14-3-3 zeta, ezrin-radixin-moesin) are involved in the regulation of cell migration, adhesion and invasion, suggesting the acquisition of a invasive phenotype.	info:pmid/16674103#abs: 5	
CFL1 --+> cell migration	Regulation	Costimulation via the T-cell receptor/CD3 complex (signal 1) together with accessory receptors (signal 2) or triggering through the chemokine SDF1a (stromal cell-derived factor	info:pmid/24117811#abs: 7	

		1a) induce Ras-dependent dephosphorylation of cofilin, which is important for immune synapse formation, T-cell activation, and T-cell migration.		
CFL1 --+> cell migration	Regulation	Cofilin-1 and actin-depolymerizing factor are required for cell migration.	info:pmid/15548599#body:250	
CFL1 --+> cell migration	Regulation	Next, we tested whether this translates to an inhibition of cofilin-mediated cell migration.	info:pmid/21832093#cont:109	
CFL1 --+> cell migration	Regulation	Thus, cofilin is likely to play a critical role in directional cell migration.5	info:pmid/21868701#cont:32	
CFL1 --+> cell migration	Regulation	A Ras-MEK-cofilin module exclusively regulates 3D T-cell migration.	info:pmid/20811337#body:34	
CFL1 --+> cell migration	Regulation	Spatial and temporal regulation of cofilin activity by LIM kinase and Slingshot is critical for directional cell migration.	info:pmid/21321325#body:498	
CFL1 --+> cell migration	Regulation	Spatial and temporal regulation of cofilin activity by LIM kinase and Slingshot is critical for directional cell migration.	info:pmid/16230460#title:1	
CFL1 --+> cell migration	Regulation	The actin-severing protein cofilin is essential for directed cell migration and chemotaxis.	info:pmid/21264073#cont:269	
CFL1 --+> cell migration	Regulation	(2005) Spatial and temporal regulation of cofilin activity by LIM kinase and Slingshot is critical for directional cell migration.	info:pmid/23646165#cont:338	
CFL1 --+> cell migration	Regulation	Thus, it seems likely that proper regulation of LIMK1 activity and cofilin phosphorylation is required for cell migration.	info:pmid/16456544#body:68	
CFL1 --+> cell migration	Regulation	The overexpression of cofilin was reported to increase the velocity of cell migration in human glioblastoma cells .	info:pmid/21237490#body:86	
CFL1 --+> cell migration	Regulation	Recently, active, nonphosphorylated cofilin was shown to set the direction and increase the rate of cell migration (Ghosh et al., 2004).	info:pmid/16061695#body:276	
CFL1 --+> cell migration	Regulation	Cofilin plays a critical role in directional cell migration and serves as a dynamic component of the cell (47).	info:pmid/23221043#cont:230	airway {Organ urn:agi-ncimorgan:C0458827}
CFL1 --+> cell migration	Regulation	Previous studies showed that cofilin stimulates lamellipod protrusion and cell migration (Chan et al., 2000; Dawe et al., 2003; Ghosh et al., 2004).	info:pmid/15684033#body:61	
CFL1 --+> cell migration	Regulation	This results in activation of SSH1L and dephosphorylation of cofilin, allowing increased directed cell migration (Fig. 9).	info:pmid/23148218#cont:465	
CFL1 --+> cell migration	Regulation	The overexpres-sion of cofilin can increase the velocity of cell migration in dictyostelium [45] and in human glioblastoma cells [46].	info:pmid/22087286#cont:449	
CFL1 --+> cell migration	Regulation	Moreover, MEK-cofilin signaling controls T-cell migration in 3 dimensions but not 2D environments (Klemke et al., 2010).	info:pmid/22946052#cont:156	
CFL1 --+> cell	Regulation	Cofilin induces actin filament severing in	info:pmid/22366462#cont:	

migration		lamellipodia and is important for cell migration and invasion (Oser and Condeelis, 2009).	277	
CFL1 --+> cell migration	Regulation	Clusterin-mediated inactivation of cofilin by phosphorylation might therefore affect actin dynamics, cell migration, and morphology.	info:pmid/24381170#cont:336	
CFL1 --+> cell migration	Regulation	Thus, the balanced phosphoregulation of cofilin/actin-depolymerizing factor activity is important for tumor cell migration and invasion.	info:pmid/18171679#body:286	
CFL1 --+> cell migration	Regulation	Thus, the balanced phosphoregulation of cofilin/actin-depolymerizing factor activity is important for tumor cell migration and invasion.	info:pmid/18171680#body:286	
CFL1 --+> cell migration	Regulation	We show that cofilin is a regulator of cell migration in inflammatory cells, as well as a key regulator of cancer-cell metastasis.	info:pmid/19158339#body:64	
CFL1 --+> cell migration	Regulation	A moderate increase in cofilin accelerates cell migration, clearly a factor in metastasis, but greater increases reverse this effect.	info:pmid/20133134#body:86	
CFL1 --+> cell migration	Regulation	The metastatic cell lines contained increased amounts of phosphorylated inactive cofilin, a protein that plays an important role in cell migration.	info:pmid/16505008#body:175	
CFL1 --+> cell migration	Regulation	Cofilin activity is important for directional cell migration by maintaining a polarized actin cytoskeleton (Dawe et al. 2003; Ghosh et al. 2004; Mouneimne et al.	info:pmid/22215812#cont:325	
CFL1 --+> cell migration	Regulation	The over expression of cofilin by 2-4 fold at the protein level increases the velocity of cell migration in Dictyostelium and in human glioblastoma cells .	info:pmid/16926057#body:99	
CFL1 --+> cell migration	Regulation	The actin-binding proteins of the actin-depolymerisation factor/cofilin family play a key role in cell migration and cancer cell invasion.	info:pmid/18499298#body:283	
CFL1 --+> cell migration	Regulation	Cofilin mediates lammellipodium extension and cell migration by affecting actin filament dynamics at the leading edge of migrating cells.	info:pmid/16725345#body:123	
CFL1 --+> cell migration	Regulation	It has been reported that the F-actin dynamics is regulated by the phosphorylating cofilin at Ser3, which is critical for cell migration and chemotaxis.	info:pmid/23063350#body:106	
CFL1 --+> cell migration	Regulation	ADF/ cofilin proteins were shown to control chemotaxis [22], as well as neuronal crest cell migration in the developing embryo [23,24].	info:pmid/22558315#cont:32	
CFL1 --+> cell migration	Regulation	Nishita, M.; Tomizawa, C.; Yamamoto, M.; Horita, Y.; Ohashi, K.; Mizuno, K. Spatial and temporal regulation of cofilin activity by LIM kinase and Slingshot is critical for directional cell migration.	info:pmid/22414009#cont:965	
CFL1 --+> cell migration	Regulation	Once phosphated, ADF/cofilin becomes inactive, losing the filament-severing and	info:pmid/22360962#body:93	

		monomer binding activities, thereby promoting actin polymerization and cell migration .		
CFL1 --+> cell migration	Regulation	Overexpression of cofilin in Dictyostelium alters cytoskeletal organization, promotes membrane ruffling, and enhances cell migration ( ).	info:pmid/17338919#body:390	
CFL1 --+> cell migration	Regulation	In a recent study, it was demonstrated that local photoactivation of a caged cofilin led to the formation of lamellipodia at the site of activation and cell migration in this direction .	info:pmid/15501439#body:54	
CFL1 --+> cell migration	Regulation	In the human immune system, cofilin regulates actin dynamics and is involved in T cell migration and activation ( ).	info:pmid/18775311#body:175	Immune system {Organ urn:agi-ncimorgan:C0020962}
CFL1 --+> cell migration	Regulation	In these studies, the authors determined that both cofilin and PLC activation were required for the initial but not late changes in the generation of free barbed ends, thus regulating cell migration.	info:pmid/16921170#body:423	
CFL1 --+> cell migration	Regulation	This indicates that cofilin plays a crucial role in cytokinesis and cell migration by promoting actin filament turnover through its actin filament-disassembling activity [17,18].	info:pmid/18494608#body:42	
CFL1 --+> cell migration	Regulation	Cofilin-1 appears crucial for cell migration events in specific cell lineages derived from the neural ectoderm and the paraxial mesoderm ( ).	info:pmid/20403747#body:160	Ectoderm {Organ urn:agi-ncimorgan:C0013574}
CFL1 --+> cell migration	Regulation	We suggest that the mechanism described contributes to the action of reactive oxygen species in modulating cofilin-mediated actin dynamics during cell migration.	info:pmid/19339277#body:294	
CFL1 --+> cell migration	Regulation	Our previous studies showed that cofilin is genetically required for cell migration in the Drosophila ovary and that it promotes lamellipodial protrusions (Chen et al., 2001; Zhang et al., 2011).	info:pmid/22899846#cont:30	
CFL1 --+> cell migration	Regulation	Cofilin, a LIM kinase 1 substrate, is directly responsible for severing actin filaments and regulating actin polymerization and depolymerization during cell migration.	info:pmid/21725598#cont:189	
CFL1 --+> cell migration	Regulation	Cofilin-1 generates available actin ends by cleavage of F-actin molecules and is therefore involved in the regulation of cell migration, adhesion and phagocytosis .	info:pmid/22705049#body:230	
CFL1 --+> cell migration	Regulation	It has been shown that manipulation of DSTN/CFL1 activity and its regulators, affects the formation of protrusions (e.g. lamellipodia) and cell migration since they are involved in	info:pmid/22898637#body:112	Mesenchyme {Organ urn:agi-ncimorgan

		invadopodia formation .		:C0162415 }
CFL1 --+> cell migration	Regulation	J Cell Biol 165: 465–471 Nishita M, Tomizawa C, Yamamoto M, Horita Y, Ohashi K, Mizuno K (2005) Spatial and temporal regulation of cofilin activity by LIM kinase and Slingshot is critical for directional cell migration.	info:pmid/21525957#cont:415	
CFL1 --+> cell migration	Regulation	A present research demonstrated that CD44-induced cell migration was dependent on its complex formation with Lyn and its consequent regulation of AKT phosphorylation and cofilin expression (Subramaniam et al. 2007).	info:pmid/20157733#cont:314	
CFL1 --+> cell migration	Regulation	In so doing, cofilin regulates cell migration behavior, cell directionality (Ghosh et al., 2004; Sidani et al., 2007), and, ultimately, cell invasion (Wang et al., 2007; Oser and Condeelis, 2009; van Rheenen et al., 2009).	info:pmid/22105349#cont:36	
CFL1 --+> cell migration	Regulation	More recent work revealed that in vivo, actin depolymerizing factor/cofilins appear to increase the level of F-actin in the cell, enhance membrane protrusion, stabilize invadopodia, and accelerate directional cell migration.	info:pmid/16530787#body:5	
CFL1 --+> cell migration	Regulation	Cofilin plays a key role in cytoskeleton dynamics and cell migration by stimulating the severing and depolymerization of actin filaments, and, under certain conditions, can nucleate actin filaments ( ).	info:pmid/21074724#body:11	
CFL1 --+> cell migration	Regulation	Tangential migration, cell shape, and actin remodeling in n-coffl/fl,nes neurons. (A) Tangential migration is partially impaired in the cortex of E16 n-cofilin mutants.	info:pmid/17875668#body:100	Cerebral cortex {Organ urn:agi-ncimorgan:C0007776 }
CFL1 --+> cell migration	Regulation	In the present report, we identified specific proteins in inflammatory exudates and cytoskeleton protein cofilin-1 that were reduced by volatile anesthetics and are known to be important in cell migration.	info:pmid/18382663#body:345	
CFL1 --+> cell migration	Regulation	In conclusion, our results show that in mouse, n-cofilin is required to regulate cell migration in specific cell lineages derived from the neural ectoderm as well as the paraxial mesoderm.	info:pmid/15649475#body:159	Ectoderm {Organ urn:agi-ncimorgan:C0013574 }
CFL1 --+> cell migration	Regulation	Cofilin (also known as ADF), which is encoded by twinstar in D. melanogaster, promotes cell migration by severing filaments, which increases the concentration of uncapped barbed ends and thus can stimulate actin polymerization.	info:pmid/23000794#cont:539	
CFL1 --+> cell	Regulation	Cofilin is one of the essential components for in	info:pmid/17085086#body	

migration		vitro reconstitution of Listeria monocytogenes motility that is driven by actin polymerization , and it has recently been reported that cofilin stimulates lamellipod protrusion and cell migration .	:127	
CFL1 --+> cell migration	Regulation	The hydrolysis of PIP2 initiates the release of cell membrane proteins, including gelsolin and cofilin, and their binding to actin, which polymerizes and contributes to tumor cell migration and metastasis .	info:pmid/22257695#body:82	
CFL1 --+> cell migration	Regulation	Cofilin is an actin-binding protein that modulates lamellipodia formation, where cofilin acts by severing actin filaments, thereby regulating actin de-polymerization and actin cytoskeletal remodeling to influence cell migration.	info:pmid/22960576#body:60	
CFL1 --+> cell migration	Regulation	It was reported that P21-activated kinase4-induced cell rounding was dependent on cofilin phosphorylation, suggesting that regulation of cofilin activity was required for cell migration ( ) and that an abundance or deficiency of cofilin had a detrimental effect on cell migratory behavior ( ).	info:pmid/19778628#body:130	
CFL1 --+> cell migration	Regulation	Ena promotes actin polymerization but often restricts cell motility (Bear et al., 2000; Krause et al., 2002; Trichet et al., 2008); cofilin severs actin filaments but can promote net actin polymerization and cell migration (Loisel et al., 1999; Blanchoin et al., 2000; Ng and Luo, 2004).	info:pmid/21447553#cont:383	
CFL1 --+> cell migration	Regulation	Whilst LIMK1 and LIMK2 double knockouts only cause a moderately more severe phenotype compared to the LIMK1 knockout ( ), knocking out the LIMK1 effector, n-cofilin/cofilin-1 results in abnormal neural cell migration, neuronal defects and embryonic lethality at E10.5 ( ).	info:pmid/21167960#body:12	
CFL1 --+> cell migration	Regulation	In addition, Rho associated kinase-mediated activation of LIMKs and subsequent cofilin phosphorylation could affect cell migration , as constitutively active LIMK2 or excess phosphorylated cofilin inhibit cell polarization by inducing the formation of numerous lamellipodia (Figure 7) .	info:pmid/24528629#cont:170	
CFL1 --+> cell migration	Regulation	Of these, 22 proteins found to be down-regulated were nutrient or drug metabolizing enzymes, while the group of 29 up-regulated proteins, which include tropomyosins, filamins A, B, and C, intergrin-ß1, heat shock protein beta-1, tansglutaminase2, cofilin, 14-3-3 zeta and ezrin-radixin-moesin, were primarily involved in cell migration, adhesion, and invasion.	info:pmid/19439204#body:55	

CFL1 --+> cell migration	Regulation	Taken together, these results suggest that excessive phosphorylation of cofilin (and thus inactivation) by LIMK1 results in the loss of cell polarity, thereby inhibiting cell migration, because cofilin is a potent regulator of actin filament dynamics and is a key player in maintaining and extending lamellipodial protrusions at the leading edge of migrating cells .	info:pmid/20347121#body:115	
CFL1 --+> cell migration	Regulation	PKR activated by double stranded RNA can induce cancer cell intracellular reorganization of actin cytoskeleton and impair the formation of lamellipodia, thus inhibiting cell migration and blocking cell membrane ruffling mediated by promoting Ser3 phosphorylative inhibition of cofilin which serves as an actin severing protein and regulates membrane ruffling, lamellipodia formation and cell migration [94].	info:pmid/24237222#cont:252	
CFL1 --+> cell migration	Regulation	These results suggest that K1735 melanoma cell migration and invasion are mediated through cofilin activity.	info:doi/10.1016/j.yexcr.2005.11.011#body:208	
CFL1 --+> cell migration	Regulation	Once phosphated, ADF/cofilin becomes inactive, losing the filament-severing and monomer binding activities, thereby promoting actin polymerization and cell migration .	info:doi/10.1016/j.burns.2011.12.017#body:93	
CFL1 --+> cell migration	Regulation	To investigate how cell migration and invasion is enhanced in high grade brain tumors by cofilin we have developed a physical model based on the molecular mechanisms for actin turnover already known to be enhanced by cofilin.	info:doi/10.1016/j.bpj.2013.11.2039#body:3	
CFL1 --+> cell migration	Regulation	We found that a net dephosphorylation of cofilin results from simultaneous activation of both enzymes, thus providing a mechanism for fine control of vascular smooth muscle cell migration. *.	info:pmid/18096821#body:71	
CFL1 --+> cell migration	Regulation	It has been reported that the F-actin dynamics is regulated by the phosphorylating cofilin at Ser3, which is critical for cell migration and chemotaxis.	info:doi/10.1016/j.ejca.2012.09.018#body:106	
CFL1 --+> cell migration	Regulation	Cofilin-1 generates available actin ends by cleavage of F-actin molecules and is therefore involved in the regulation of cell migration, adhesion and phagocytosis .	info:doi/10.1016/j.intimp.2012.05.022#body:230	
CFL1 --+> cell migration	Regulation	Cofilin is an actin-binding protein that modulates lamellipodia formation, where cofilin acts by severing actin filaments, thereby regulating actin de-polymerization and actin cytoskeletal remodeling to influence cell migration.	info:doi/10.1016/j.mito.2012.08.002#body:60	
PEBP1 --+> cell migration	Regulation	Small interfering RNA-mediated silencing of RKIP expression also reduces cell migration	info:pmid/16183022#abs:5	

		rate.		
PEBP1 --+> cell migration	Regulation	RKIP augmented Hepatic stellate cell migration and enhanced wound closure.	info:pmid/19323783#abs:14	
PEBP1 --+> cell migration	Regulation	As a modulator of key signaling pathways, RKIP affects various cellular processes including cell differentiation, the cell cycle, apoptosis and cell migration.	info:pmid/18781826#abs:4	
PEBP1 --+> cell migration	Regulation	Inhibition of RKIP by small hairpin RNA transfection significantly decreased the inhibitory effect of Gemifloxacin on the nuclear factor ?B/Snail pathway and also inhibited cell migration and invasion.	info:pmid/24005829#abs:10	
PEBP1 --+> cell migration	Regulation	Ectopic expression of RKIP significantly decreased FOCUS cell migration rates (28%) compared with control cells (56%) (C).	info:pmid/17030190#body:133	
PEBP1 --+> cell migration	Regulation	In other reports, RKIP promotes cell migration by downregulating E cadherin and upregulating ß1 integrin [13], [14].	info:pmid/19551145#body:265	
PEBP1 --+> cell migration	Regulation	It could involve a direct effect on cell movement itself, in which case one would expect that RKIP would negatively regulate cell migration.	info:pmid/20855151#body:16	Intestines {Organ urn:agi-ncimorgan:C0021853}
PEBP1 --+> cell migration	Regulation	In addition, PEBP has been shown to regulate cell migration , to be an inhibitor of non-apoptotic programmed cell death and to act as a tumor suppressor.	info:pmid/21917533#body:4	
PEBP1 --+> cell migration	Regulation	Collectively, phosphatidylethanolamine-binding protein-1 affects various cellular processes including cell differentiation, cell cycle, apoptosis, and cell migration [32].	info:pmid/22227918#cont:639	tibialis anterior muscle {Organ urn:agi-organ:tibialis%20anterior%20muscle}
PEBP1 --+> cell migration	Regulation	Thus, RKIP mediates important cellular mechanisms, including cell differentiation, cell cycle, apoptosis and cell migration, and is deregulated in several human disorders (13).	info:pmid/23232914#cont:16	Intestines {Organ urn:agi-ncimorgan:C0021853}
PEBP1 --+> cell migration	Regulation	Hence, evidence indicates that RKIP regulates the activity and mediates the crosstalk between several important cellular signaling pathways including cell differentiation, cell cycle, apoptosis and cell migration.	info:pmid/23601922#body:15	Prostate {Organ urn:agi-ncimorgan:C1278980}
PEBP1 --+> cell migration	Regulation	The other crucial function of PEBP is to inhibit the activity of serine proteases, which can degrade components of the extracellular matrix	info:pmid/21556466#cont:157	

		to allow outgrowth of neuronal processes or to allow cell migration.		
PEBP1 --+> cell migration	Regulation	By influencing the Raf kinase and NF-?B pathways, RKIP-1 may make some cell types particularly sensitive to apoptotic signals , and it is possible that the absence of RKIP-1 promotes breast cell migration and metastasis by inducing the expression of chemokine receptors .	info:pmid/17706925#body:68	
YWHAE ---> cell migration	Regulation	Knockdown with specific siRNA abolished 14-3-3e-induced cell migration and epithelial-mesenchymal transition.	info:pmid/23483955#abs:5	
YWHAE ---> cell migration	Regulation	Moreover, overexpression of 14-3-3epsilon resulted in the inhibition of cell migration induced by MAPK-activated protein kinase 5 overexpression or TNFalpha treatment.	info:pmid/17728103#abs:4	
YWHAE ---> cell migration	Regulation	The authors also demonstrated that 14-3-3e prevented MK5-mediated F-actin reorganization and cell migration.	info:pmid/19166925#body:88	
YWHAE ---> cell migration	Regulation	14-3-3-epsilon inhibits cell migration by blocking HSP 27 phosphorylation, which is required for F-actin polymerization.33	info:pmid/22925547#cont:234	
YWHAE ---> cell migration	Regulation	Tak et al. found that 14-3-3epsilon inhibits cell migration in HeLa cells by interacting with MAPK-activated protein kinase 5 [29].	info:pmid/20565895#body:259	
YWHAE ---> cell migration	Regulation	Recently, exciting work with knockout mice has revealed molecular links between 14-3-3e and Lis1 that may underlie the cell migration abnormality of these diseases [158].	info:pmid/15167810#body:271	Cerebral cortex {Organ urn:agi-ncimorgan:C0007776}
PGK1 --+> cell migration	Regulation	These results suggest that MIG-10 and ABI-1 interact physically to promote cell migration and process outgrowth in vivo.	info:pmid/23022657#abs:11	
PGK1 --+> cell migration	Regulation	Grb7 is an Src homology 2-containing and pleckstrin homology domain-containing molecule, which shares significant homology with the Caenorhabditis elegans gene for Mig-10 involved in cell migration during embryogenesis.	info:pmid/10446223#abs:2	
PGK1 --+> cell migration	Regulation	PGK1 modulates U251 cell migration ability.	info:pmid/24284928#cont:145	
PGK1 --+> cell migration	Regulation	Grb7, Grb10 and Grb14 form a protein family that is phylogenetically related to the Caenorhabditis elegans Mig10 protein, which is involved in the regulation of embryonic neural cell migration .	info:pmid/23743201#body:2	
PGK1 --+> cell migration	Regulation	Interestingly, MIG-10 has been shown to asymmetrically localize in response to Netrin to mediate directional axon outgrowth and cell migration (Manser and Wood, 1990; Manser et	info:pmid/22451697#cont:173	

		al., 1997; Adler et al., 2006; Chang et al., 2006; Quinn et al., 2008).		
PGK1 --+> cell migration	Regulation	Firstly, Grb7 family members share a significantly conserved molecular architecture with the Caenorhabditis elegans Mig-10 protein, which is involved in neuronal cell migration during embryonic development (4, 5, 26), suggesting that Grb7 may play a role in cell migration.	info:pmid/19473962#body:44	
YWHAZ --+> cell migration	Regulation	The majority of the TGF-beta-induced proteins (such as tropomyosins, filamin A, B, & C, integrin-beta1, heat shock protein27, transglutaminase2, cofilin, 14-3-3 zeta, ezrin-radixin-moesin) are involved in the regulation of cell migration, adhesion and invasion, suggesting the acquisition of a invasive phenotype.	info:pmid/16674103#abs:5	
YWHAZ --+> cell migration	Regulation	ErbB2 and 14-3-3zeta overexpression, respectively, increased cell migration and decreased cell adhesion, two prerequisites of tumor cell invasion. 14-3-3zeta overexpression reduced cell adhesion by activating the TGF-beta/Smads pathway that led to ZFHX1B/SIP-1 upregulation, E-cadherin loss, and epithelial-mesenchymal transition.	info:pmid/19732720#abs:4	
YWHAZ --+> cell migration	Regulation	Known targets of 14-3-3zeta important for regulating cell migration and polarity are depicted..	info:pmid/15037601#body:235	
YWHAZ --+> cell migration	Regulation	It was proposed that this interaction is stabilized by 14-3-3-? in a ternary complex that accelerates cell migration.	info:pmid/23763993#body:16	
YWHAZ --+> cell migration	Regulation	14-3-3 zeta protein also facilitate cell migration by forming a ternary complex with integrin alpha-4 and paxillin .	info:pmid/24238270#cont:955	
YWHAZ --+> cell migration	Regulation	ErbB2 and 14-3-3zeta over-expression, respectively, increases cell migration and decreases cell adhesion.	info:pmid/22193627#cont:145	
YWHAZ --+> cell migration	Regulation	An integrin-alpha4-14-3-3zeta-paxillin ternary complex mediates localised Cdc42 activity and accelerates cell migration.	info:pmid/19401330#title:1	
YWHAZ --+> cell migration	Regulation	Silencing of 14-3-3? expression consequently suppressed EGF-induced cell migration in A431 cells (Fig. 4E).	info:pmid/21868386#cont:115	
YWHAZ --+> cell migration	Regulation	An integrin-alpha4-14-3-3zeta-paxillin ternary complex mediates localised Cdc42 activity and accelerates cell migration.	info:pmid/24163432#cont:384	
YWHAZ --+> cell migration	Regulation	Phosphorylation of 14-3-3? may trigger cell migration and associated events (e.g. changes in Golgi polarity and cell adhesion) [36] and may prevent Golgi dispersion [29].	info:pmid/23889253#cont:252	
YWHAZ --+> cell migration	Regulation	For example, overexpression of 14-3-3? in breast cancer cells conferred a higher risk of	info:pmid/20647762#cont:141	

		progression to an invasive phenotype, as well as increased cell migration and decreased cell adhesion.32		
YWHAZ --+> cell migration	Regulation	Of these, 22 proteins found to be down-regulated were nutrient or drug metabolizing enzymes, while the group of 29 up-regulated proteins, which include tropomyosins, filamins A, B, and C, intergrin-ß1, heat shock protein beta-1, tansglutaminase2, cofilin, 14-3-3 zeta and ezrin-radixin-moesin, were primarily involved in cell migration, adhesion, and invasion.	info:pmid/19439204#body:55	
ENO1 --+> cell migration	Regulation	Therefore, development of novel therapeutic strategies, such as anti-Pancreatic cancer immunotherapy, is crucial. a-Enolase (ENO1) is an enzyme expressed on the surface of pancreatic cancer cells and is able to promote cell migration and cancer metastasis.	info:pmid/23640603#abs:3	
ENO1 --+> cell migration	Regulation	Secreted ENO1 promotes prostate cancer cell migration via its plasminogen-binding domain	info:pmid/22734040#cont:226	Prostate {Organ urn:agi-ncimorgan:C1278980}
ENO1 --+> cell migration	Regulation	CK8 and a-enolase bind and activate plasminogen to promote cell migration and invasion (13, 14, 17).	info:pmid/20406904#body:108	
ENO1 --+> cell migration	Regulation	Recent studies revealed that, in addition to its innate catalytic function, a-enolase plays an important role in other biological/pathological processes, such as myogenesis , tRNA transport , K+ channel regulation , and tumor cell migration .	info:pmid/24361255#body:7	Neurosecretory Systems {Organ urn:agi-ncimorgan:C0229526}
HNRNPK --+> cell migration	Regulation	Involvement of MEK and ERK in hnRNP-K-induced cell migration.	info:pmid/23564449#cont:220	
HNRNPK --+> cell migration	Regulation	More recently, it was shown that hnRNP K (56), G3BP1 (41), and Sam68 regulate cell migration (3).	info:pmid/19762470#body:318	
HNRNPK --+> cell migration	Regulation	We found that the cytoplasmic localization of hnRNP-K may mediate its role in cell migration and metastasis.	info:pmid/17483488#body:164	
HNRNPK --+> cell migration	Regulation	Heterogeneous ribonucleoprotein K has also been shown to be involved in cell migration; a process necessary for cancer metastasis.	info:pmid/16953238#body:60	
HNRNPK --+> cell migration	Regulation	Recent work has shown that hnRNP K regulates antiapoptosis and cell migration, two biological functions that are critically related to cancer development.9,11	info:pmid/20224598#cont:296	
HNRNPK --+> cell migration	Regulation	This further supports a role of hnRNP K in tumorigenesis that can be attributed to	info:pmid/22582387#cont:282	

		improved tumor cell survival (Figs. 5 and 6) and/or improved tumor cell migration and metastasis (37).		
TUBA1A ---> cell migration	Regulation	That is the case of genes such as GNB2L1, an anchor protein involved in adhesion and migration of human glioma cells [51], DPYSL2, a promoter of microtubule assembly and neuronal development [52], TUBA1A [53] or CFL, which controls cell migration and cell cycle progression [54,55].	info:pmid/20735813#cont:411	
CKB --+> cell migration	Regulation	Our experimental verification of this obviously speculative model showed that CK-B with a membrane anchor could indeed facilitate lamellipodia formation and stimulated cell migration.	info:pmid/19333390#body:278	
ATP5A1 --+> cell migration	Regulation	The suppression of cell migration by the anti-ATP synthase a-subunit antibody was not limited to a specific ECM interaction.	info:pmid/22152132#cont:657	
PRDX1 ---> cell migration	Regulation	Knockdown of human peroxiredoxin 1 significantly inhibited TGF-ß1-induced epithelial-to-mesenchymal transition and cell migration, whereas human peroxiredoxin 1 overexpression enhanced TGF-ß1-induced epithelial-to-mesenchymal transition and cell migration.	info:pmid/22475482#abs:5	
PRDX1 ---> cell migration	Regulation	For example, peroxiredoxin I and II association with the platelet-derived growth factor receptor in vascular smooth muscle cells regulated platelet-derived growth factor–dependent signaling and cell migration.39	info:pmid/21636804#cont:238	
PKM --+> cell migration	Regulation	While in undifferentiated gastric cancer cells that lack E-cadherin, PKM2 can enhance EGFR downstream signaling activation and promote cell migration and invasion.56	info:pmid/24131935#cont:109	
PKM --+> cell migration	Regulation	This result is the opposite of what was observed with the BGC823 and SGC7901 cells; in AGS cells, PKM2 came into play as a stimulus and promoted cell migration and invasion.	info:pmid/23840737#cont:199	
