# Supplementary material for: Deciphering the Role of Emx1 in Neurogenesis: A Neuroproteomics Approach
Source: Front Mol Neurosci. 2016 Oct 17;9:98. doi: 10.3389/fnmol.2016.00098 (PMC5065984; doi:10.3389/fnmol.2016.00098)
Supplement: Supplementary file 5 [file Table5.PDF]

Table 5a: Cell proliferation Cluster

Entity Table:

Name	Type	Description	Connectivity	Local Connectivity	Indegree
ENO1	Protein	enolase 1, (alpha)	336	1	0
PGK1	Protein	phosphoglycerate kinase 1	155	1	0
PKM	Protein	pyruvate kinase, muscle	346	1	0
OAT	Protein	ornithine aminotransferase	98	1	0
ATP5A1	Protein	ATP synthase, H+ transporting, mitochondrial F1 complex, alpha subunit 1, cardiac muscle	139	1	0
YWHAB	Protein	tyrosine 3-monooxygenase/tryptophan 5-monooxygenase activation protein, beta polypeptide	251	1	0
HSPD1	Protein	heat shock 60kDa protein 1 (chaperonin)	988	1	0
CFL1	Protein	cofilin 1 (non-muscle)	920	1	0
HNRNPK	Protein	heterogeneous nuclear ribonucleoprotein K	412	1	0
HNRNPA1	Protein	heterogeneous nuclear ribonucleoprotein A1	353	1	0
PEBP1	Protein	phosphatidylethanolamine binding protein 1	384	1	0
PRDX1	Protein	peroxiredoxin 1	388	1	0
PRDX2	Protein	peroxiredoxin 2	347	1	0
YWHAZ	Protein	tyrosine 3-monooxygenase/tryptophan 5-monooxygenase activation protein, zeta polypeptide	557	1	0
PSMB6	Protein	proteasome subunit beta type 6-like	10	1	0
ARL3	Protein	ADP-ribosylation factor-like 3	44	1	0
YWHAE	Protein	tyrosine 3-monooxygenase/tryptophan 5-monooxygenase activation protein, epsilon polypeptide	328	1	0
YWHAG	Protein	tyrosine 3-monooxygenase/tryptophan 5-monooxygenase activation protein, gamma polypeptide	200	1	0
CKB	Protein	creatine kinase, brain	158	1	0
cell proliferation	Cell Process		8159	19	19

Table 5b: Cell proliferation Cluster

Relationship Table:

Relation	Type	Sentence	TextRef	Connectivity	# of References	Organ
ARL3 ---> cell proliferation	Regulation	The presence of apparently normal cilia in the Arl3 (-/-) mice suggests that the primary defect is not structurally defective cilia, but that Arl3 function may be critical for proper regulation of the intracellular transport process and/or Ca ++ signaling and proliferation.	info:pmid/16565502#body:209	2	1	
YWHAB --+> cell proliferation	Regulation	These results suggest that 14-3-3beta is a downstream effector of xCT in KS to mediate the cell proliferation., Knockdown of ß-catenin by small interfering RNA blocked cell proliferation induced by overexpression of 14-3-3ß., These results provide additional evidence that RNH-1/14-3-3 beta protein participates in cellular differentiation, proliferation and transformation through the signal transduction pathways of various growth factors., An earlier study showed that overexpression of 14-3-3ß promotes cell proliferation and oncogenic transformation. <more data available...>	info:pmid/20100173#abs:6, info:pmid/24065186#abs:6, info:pmid/8749325#abs:6, info:pmid/21967815#body:154, info:pmid/21946067#body:61, info:pmid/23886124#cont:73, info:pmid/16725345#body:193, info:pmid/24268498#body:80, info:pmid/18249187#body:70, info:pmid/15147740#body:139 <more	2	11	Gastric mucosa {Organ urn:agi-ncimorgan:C0017136}

			data available...>			
HSPD1 --+> cell proliferation	Regulation	Finally, the DNA-HSP65 vaccine was able to induce proliferation of peripheral blood lymphocytes., We assessed the hypothesis that HSP60 is induced in vascular cells infected with C pneumoniae and stimulates cell proliferation., Adenoviral-mediated overexpression of HSP60 induced increases in cell proliferation compared with uninfected vascular smooth muscle cell., HSP60 could promote DCs expressed CD86 and stimulate T lymphocytes proliferation in vitro, while Puerarin had significantly inhibitory effect., Altered peptide ligands of p1-20 can inhibit p1-20/hsp65-induced proliferation of DR3-restricted T cells in an allele specific manner in vitro. <more data available...>	info:pmid/18208592#abs:11, info:pmid/14500333#abs:5, info:pmid/23210434#abs:6, info:pmid/17569333#abs:8, info:pmid/9559973#abs:3, info:pmid/11339820#abs:12, info:pmid/7878764#abs:6, info:pmid/20433702#abs:8, info:pmid/7813115#abs:6, info:pmid/16843693#abs:12 <more data available...>	2	48	Blood Vessels {Organ urn:agi-ncimorgan:C0005847}, Brain {Organ urn:agi-ncimorgan:C1269537}, Popliteal lymph node group {Organ urn:agi-ncimorgan:C0588057}, Ocular orbit {Organ urn:agi-ncimorgan:C0029180}, Umbilical Blood {Organ urn:agi-ncimorgan:C1550673}, Placenta {Organ urn:agi-ncimorgan:C1550656}, Breast {Organ urn:agi-ncimorgan:C0006141}, Large Intestine {Organ urn:agi-ncimorgan:C1268999}
PEBP1 ---| cell proliferation	Regulation	The RKIP gene may play a role in inhibiting cellular proliferation., Ectopic expression of RKIP altered hepatocellular carcinoma cell proliferation and migration., RKIP inhibition potentiated mitogen-induced proliferation in lung fibroblasts., RKIP inhibits proliferation and transformation of myeloid cells and decreases transformation induced by mutant RAS., RKIP is important in beta-cell proliferation, and its downregulation may play a role in islet neoplasia., These results indicate that PEBP	info:pmid/22373584#abs:11, info:pmid/17030190#abs:11, info:pmid/21385176#abs:13, info:pmid/22388727#abs:9, info:pmid/15349122#abs:13, info:pmid/15155742#abs:13,	2	23	Lung {Organ urn:agi-ncimorgan:C1278908}, Islets of Langerhans {Organ urn:agi-ncimorgan:C0022131}, Heart {Organ urn:agi-ncimorgan:C1281570}, Umbilical Blood {Organ urn:agi-ncimorgan:C1550673}, head {Organ urn:agi-ncimorgan:C0018670},

		not only inhibits cell proliferation but also induces differentiation of human keratinocytes. <more data available...>	info:pmid/18567796#abs:8, info:pmid/23259789#abs:9, info:pmid/23416466#abs:5, info:pmid/15063784#abs:5 <more data available...>			Liver {Organ urn:agi-ncimorgan:CL384198}, Mesenchyme {Organ urn:agi-ncimorgan:C0162415}, Brain {Organ urn:agi-ncimorgan:C1269537}, Hippocampus {Organ urn:agi-ncimorgan:C0019564}
PKM --+> cell proliferation	Regulation	Knockdown of PKM2 attenuated PRL-stimulated cell proliferation., Knockdown of PKM2 repressed proliferation and migration of the cells., Acetylation-mimetic PKM2(K433) mutant promotes cell proliferation and tumorigenesis., Additionally, PKM2-SAICAR was necessary to induce sustained Erk1/2 activation and mitogen-induced cell proliferation., Expression of a PKM2 mutant that exists as a dimer promotes cell proliferation, indicating that protein kinase activity of PKM2 plays a role in promoting cell proliferation. <more data available...>	info:pmid/20962042#abs:5, info:pmid/22807066#abs:6, info:pmid/24120661#abs:5, info:pmid/24606918#abs:7, info:pmid/22306293#abs:8, info:pmid/20005212#abs:3, info:pmid/22963766#abs:5, info:pmid/22580449#abs:7, info:pmid/23791887#body:130, info:pmid/22626471#body:114 <more data available...>	2	42	Fetus {Organ urn:agi-ncimorgan:C1305737}
PRDX1 ---> cell proliferation	Regulation	In addition PRDX1 is involved in cell differentiation and proliferation, apoptosis and innate immunity., This indicates that	info:pmid/17913528#abs:2, info:pmid/8089076	2	19	Brain {Organ urn:agi-ncimorgan:C1269537}, Dorsum {Organ urn:agi-

		OSF-3 plays an intrinsic role in the proliferation and/or differentiation of bone cells., Deficiency of Prx I impaired cell proliferation and anchorage-independent growth., This indicates that Prx-I plays a role in cell proliferation, which is coincident with our previous result., PrxI possesses not only antioxidant activity, but also regulatory activity in associated cell proliferation . <more data available...>	#abs:5, info:pmid/23186333#cont:171, info:pmid/17976536#body:113, info:pmid/11065135#body:66, info:pmid/20732753#body:102, info:pmid/22561679#body:86, info:pmid/19566940#body:73, info:pmid/18996230#body:133, info:pmid/18992915#body:87 <more data available...>			ncimorgan:C0460009}
HNRNPK --+> cell proliferation	Regulation	We found that SET and/or hnRNPK protein accumulation increased cellular proliferation., Finally, overexpression of hnRNP K in breast cancer cells significantly increased target c-myc promoter activity and c-Myc protein, hnRNP K protein levels, and enhanced breast cancer cell proliferation and growth in an anchorage-independent manner., These data suggested that hnRNP K plays an important role in cell growth and proliferation., HnRNP K is involved in switching from proliferation to (neuronal) differentiation . <more data available...>	info:pmid/24508256#abs:5, info:pmid/11121407#abs:7, info:pmid/20499280#cont:173, info:pmid/19410666#body:130, info:pmid/23170974#cont:254, info:pmid/21799787#cont:335, info:pmid/19520192#body:169, info:pmid/18424265#body:176,	2	17	Spleen {Organ urn:agi-ncimorgan:C1278932}, Breast {Organ urn:agi-ncimorgan:C0006141}, Brain {Organ urn:agi-ncimorgan:C1269537}

			info:pmid/15671036#body:345, info:pmid/12476304#body:145 <more data available...>			
HNRNPA1 ---> cell proliferation	Regulation	Down regulation of hnRNP A1 expression by RNA interference inhibits the proliferation and migration of cancerous HepG2 cells, while overexpression of hnRNP A1 in normal HL-7702 cells increased the proliferation and migration of the cells., The over-expression of hnRNP A1 could contribute to the maintenance of telomere repeats in cancer cells and allow enhanced cell proliferation., The shuttling of hnRNP A1 is subject to regulation and is thought to play a role in cell proliferation, survival, and differentiation of normal and transformed cells (10). <more data available...>	info:pmid/23062008#abs:5, info:pmid/16513142#body:128, info:pmid/15738418#body:49, info:pmid/17652158#body:269, info:pmid/16378690#body:218, info:pmid/16286244#body:14, info:pmid/22101062#body:101, info:pmid/22227431#body:16	2	8	
YWHAG --+> cell proliferation	Regulation	14-3-3 gamma is stimulated by IL-3 and promotes cell proliferation., Recent studies indicated that expression of 14-3-3 ? promotes cell proliferation ., Human 14-3-3 gamma protein results in abnormal cell proliferation in the developing eye of Drosophila melanogaster., In addition, 14-3-3? induces resistance to apoptotic programmed cell death and promotes proliferation of IL-3-dependent Ba/F3 cells ., Recent studies demonstrated that up-regulation of 14-3-3? promotes cell survival and proliferation through activation of	info:pmid/19124748#title:1, info:pmid/20870266#body:12, info:pmid/18194556#title:1, info:pmid/22658894#body:68, info:pmid/23500129#body:192, info:pmid/24386293#cont:303, info:pmid/2192044	2	7	Eye {Organ urn:agi-ncimorgan:C1550636}, Lung {Organ urn:agi-ncimorgan:C1278908}, Aorta {Organ urn:agi-ncimorgan:C1278934}, Cerebellum {Organ urn:agi-ncimorgan:C1268981}

		distinct signal pathways in hematopoietic progenitor cells . <more data available...>	5#body:39			
OAT ---> cell proliferation	Regulation	In mammals, Ornithine d-aminotransferase has been shown to modulate cell proliferation by regulating intracellular ornithine concentrations.	info:pmid/20673832#body:204	2	1	
CFL1 ---> cell proliferation	Regulation	Cofilin-1 plays roles in cell migration, proliferation and phagocytosis., In addition, n-cofilin is required for neuronal precursor cell proliferation and scattering., Conversely, up-regulation of CFL1 in NSCs increased proliferation, adhesion, invasion and expression of the markers but reduced apoptosis., The activity of cofilin, an actin-remodeling protein, is required for T lymphocyte activation with regard to formation of the immunological synapse, cytokine production, and proliferation., Thirteen proteins from several pathways (nucleoside diphosphate kinase A, stathmin, valosin-containing protein, annexin A1, dihydropyrimidinase-related protein-3, DJ-1 protein, glutathione S-transferase P, lamin A/C, fascin, cofilin, vimentin, vinculin, and moesin) were differentially expressed and most have been shown to play a role in differentiation, migration, invasion, proliferation, apoptosis, drug resistance, or oncogenesis. <more data available...>	info:pmid/24023293#abs:6, info:pmid/15649475#abs:7, info:pmid/20713416#abs:10, info:pmid/16424196#abs:1, info:pmid/19156760#abs:5, info:pmid/22558315#cont:167, info:pmid/15572110#body:69, info:pmid/23954413#body:90, info:pmid/21894436#cont:119, info:pmid/24370186#body:116 <more data available...>	2	17	Ovary {Organ urn:agi-ncimorgan:CL384202}, Pancreas {Organ urn:agi-ncimorgan:C1278931}, Periodontal Ligament {Organ urn:agi-ncimorgan:C0031093}, Nervous system {Organ urn:agi-ncimorgan:C0027763}, Brain {Organ urn:agi-ncimorgan:C1269537}
YWHAZ --+> cell proliferation	Regulation	It is concluded that 14-3-3? plays an important role in proliferation of AML cells and associates with BCL-2 and MCL-1 expression., Knockdown of YWHAZ	info:pmid/23998576#abs:13, info:pmid/23422756#abs:7,	2	12	Veins {Organ urn:agi-ncimorgan:C0042449}, Endocrine system {Organ urn:agi-

		expression using several specific siRNAs inhibited the proliferation, migration, and invasion of YWHAZ-overexpressing gastric cancer cells., Using Co-immunoprecipitation, we demonstrated that 14-3-3zeta protein binds to NFkappaB, beta-catenin and Bcl-2, suggesting its involvement in cellular signaling, leading to proliferation of oral cancer cells., Depletion of 14-3-3? markedly increased apoptosis, reduced proliferation and receptor tyrosine kinase (HER2 and EGFR) signaling, and, importantly, reversed endocrine resistance. <more data available...>	info:pmid/17764575#abs:9, info:pmid/21707964#abs:8, info:pmid/24197133#abs:8, info:pmid/22904106#title:1, info:pmid/21334806#body:76, info:pmid/23344024#cont:290, info:pmid/21666713#cont:483, info:pmid/24629487#body:85 <more data available...>			ncimorgan:C1280975}, Pancreas {Organ urn:agi-ncimorgan:C1278931}
PRDX2 ---> cell proliferation	Regulation	These findings indicate that peroxiredoxin2 is involved in the proliferation of androgen receptor-expressing prostate cancer cells by modulating androgen receptor activity., Prx II deficiency results in increased production of H2O2, enhanced activation of Platelet-derived growth factor receptor and phospholipase Cgamma1, and subsequently increased cell proliferation and migration in response to Platelet-derived growth factor., Prx II deletion enhances concanavalin A -induced splenocyte proliferation and mixed lymphocyte reaction activity of bone marrow-derived CD11c-positive dendritic cells to stimulate recipient splenocytes. <more data available...>	info:pmid/21539911#abs:9, info:pmid/15902258#abs:4, info:pmid/16290204#abs:8, info:pmid/23892988#abs:7, info:pmid/23307788#cont:125, info:pmid/18718523#body:236, info:pmid/20812900#cont:277, info:pmid/18503785#body:149, info:pmid/1629002	2	19	Bone Marrow {Organ urn:agi-ncimorgan:C0005953}, Colorectal Region {Organ urn:agi-ncimorgan:C1711309}, Ovarian Follicle {Organ urn:agi-ncimorgan:C1283799}, Ovary {Organ urn:agi-ncimorgan:CL384202}, Aorta {Organ urn:agi-ncimorgan:C1278934}, Blood Vessels {Organ urn:agi-ncimorgan:C0005847}

			0#body:99, info:pmid/20919930#cont:333 <more data available...>			
ENO1 ---> cell proliferation	Regulation	This study suggested that exogenous expression of MBP-1 induces cell death in fibroblasts by blocking cell proliferation., We found that cell proliferation was inhibited by MBP-1 overexpression in human stomach adenocarcinoma SC-M1 cells., Overexpressed ENO1 not only restored cell proliferation and cell-cycle progression, but also antagonized the regulation of NESG1 to cell-cycle regulators p21 and CCNA1 expression as well as induced the expression of C-Myc, pRB, and E2F1 in NESG1-ovexpressed nasopharyngeal carcinoma cells. <more data available...>	info:pmid/8519685#abs:7, info:pmid/19846662#abs:5, info:pmid/22997098#abs:5, info:pmid/20435467#body:151, info:pmid/16466929#body:165, info:pmid/23118496#cont:198, info:pmid/15805119#body:87, info:pmid/23381546#cont:315, info:pmid/20412594#body:255, info:pmid/20498720#body:230 <more data available...>	2	13	Pancreas {Organ urn:agi-ncimorgan:C1278931}, Brain {Organ urn:agi-ncimorgan:C1269537}, Cardiovascular system {Organ urn:agi-ncimorgan:C1269562}, Gastric mucosa {Organ urn:agi-ncimorgan:C0017136}
PGK1 ---> cell proliferation	Regulation	PGK1 modulates U251 cell proliferation., Besides PGK1 gene, concomitant up-regulation of FGF1, FGF2, IL6, MUC1, and platelet-derived growth factor alpha polypeptide genes in F28/KMUH Cancer-associated fibroblasts may also be the explanations for Hepatocellular carcinoma cells to promote proliferation of F28/KMUH Cancer-associated fibroblasts	info:pmid/24284928#cont:116, info:pmid/23684136#body:79, info:doi/10.1016/j.kjms.2012.08.012#body:79	2	3	

		and thus promote cancer progression.				
YWHAE ---> cell proliferation	Regulation	14-3-3epsilon regulates a wide range of biological processes, including cell cycle control, proliferation, and apoptosis, and plays a significant role in neurogenesis and the formation of malignant tumours., YWHAE is involved in neuronal migration, proliferation, and cognitive impairment ( ).	info:pmid/20565895#abs:1, info:pmid/23892282#body:169	2	2	Prosencephalon {Organ urn:agi-ncimorgan:C0085140}
PSMB6 --+> cell proliferation	Regulation	Knockdown of PSMB6 using siRNA also prevented hypoxia-induced proliferation.	info:pmid/23844134#abs:10	2	1	Pulmonary artery {Organ urn:agi-ncimorgan:C0034052}
ATP5A1 ---> cell proliferation	Regulation	An antibody against the ATP synthase a-subunit inhibited proliferation, migration and invasion in these breast cancer cells but not that of a non-tumor derived breast cell line., The identified copy number amplification genes, such as ZNF521, RNF138, RAB12, ATP5A1, PTPN2 and CTAGE1, are involved in multiple cellular processes, including transcription, proliferation, differentiation, migration and immunity., Mitochondrial ATP synthase inhibitors antagonize 5-Fluorouracil-induced suppression of cell proliferation. <more data available...>	info:pmid/22152132#abs:10, info:pmid/23678296#cont:270, info:pmid/15833846#body:183, info:pmid/15753359#body:256	2	4	Fetus {Organ urn:agi-ncimorgan:C1305737}
CKB ---> cell proliferation	Regulation	It was found that Creatine kinase B knockdown inhibited Skov3 cell proliferation and induced apoptosis under hypoxia or hypoglycemia conditions., Furthermore, overexpression of CFP-tagged wild-type Creatine kinase brain in Caco-2 colon cancer cells dramatically increased the number of cells in G2/M but had little effect on cell proliferation.	info:pmid/23416112#abs:5, info:pmid/21308735#abs:7	2	2	

Table 5c: Cell proliferation Cluster

Reference Table:

Relation	Type	Sentence	TextRef	Organ
ARL3 ---> cell proliferation	Regulation	The presence of apparently normal cilia in the Arl3 (-/-) mice suggests that the primary defect is not structurally defective cilia, but that Arl3 function may be critical for proper regulation of the intracellular transport process and/or Ca ++ signaling and proliferation.	info:pmid/16565502 #body:209	
YWHAB --+> cell proliferation	Regulation	These results suggest that 14-3-3beta is a downstream effector of xCT in KS to mediate the cell proliferation.	info:pmid/20100173 #abs:6	
YWHAB --+> cell proliferation	Regulation	Knockdown of ß-catenin by small interfering RNA blocked cell proliferation induced by overexpression of 14-3-3ß.	info:pmid/24065186 #abs:6	
YWHAB --+> cell proliferation	Regulation	These results provide additional evidence that RNH-1/14-3-3 beta protein participates in cellular differentiation, proliferation and transformation through the signal transduction pathways of various growth factors.	info:pmid/8749325# abs:6	
YWHAB --+> cell proliferation	Regulation	An earlier study showed that overexpression of 14-3-3ß promotes cell proliferation and oncogenic transformation.	info:pmid/21967815 #body:154	
YWHAB --+> cell proliferation	Regulation	However, si-14-3-3ß decreased estrogen-induced proliferation of MCF7 cells by 4.5-fold (B).	info:pmid/21946067 #body:61	
YWHAB --+> cell proliferation	Regulation	The overexpression of 14-3-3ß protein can stimulate cell proliferation and migration, thus contribute to the growth of tumor.	info:pmid/23886124 #cont:73	
YWHAB --+> cell proliferation	Regulation	Sugiyama et al. using 14-3-3ß antisense demonstrated the role of 14-3-3ß in the rat hepatoma AFB1-K2 cancer cell progression showing that reduction in 14-3-3ß causes reduced cell proliferation and tumorigenicity in mice .	info:pmid/16725345 #body:193	
YWHAB --+> cell	Regulation	In search of intracellular regulatory	info:pmid/24268498	

proliferation		factors responsible for the induction of proliferation characterizing IPs, 14-3-3ß expression was investigated. 14-3-3ß is known to play a critical regulatory role in both survival and proliferation .	#body:80	
YWHAB --+> cell proliferation	Regulation	Earlier studies indicated that the expression of 14-3-3 proteins is increased in some human cancers, suggesting that these proteins may act as oncogenes. 14-3-3 ß has oncogenic effect in the context of cellular proliferation and tumorigenesis, and it is over-expressed in various murine tumor cell lines .	info:pmid/18249187 #body:70	
YWHAB --+> cell proliferation	Regulation	In conclusion, results to date suggest that the differential expression of 14-3-3 protein a/ß, Cullin homolog 3, a-enolase and ezrin in H. pylori infection may play an important role in gastric carcinogenesis, including cell proliferation and cell adhesion, and that they may be induced by reactive oxygen species-mediated cell signaling.	info:pmid/15147740 #body:139	Gastric mucosa {Organ urn:agi-ncimorgan:C0017136}
YWHAB --+> cell proliferation	Regulation	Considering the structural and functional similarities between 14-3-3 and a-synuclein , it is of interest that over-expression of the 14-3-3ß isoform in NIH3T3 cells induces extracellular signal-regulated kinase phosphorylation and increases cellular proliferation .	info:doi/10.1016/S0197-4580(02)00196-3#body:122	
HSPD1 --+> cell proliferation	Regulation	Finally, the DNA-HSP65 vaccine was able to induce proliferation of peripheral blood lymphocytes.	info:pmid/18208592 #abs:11	
HSPD1 --+> cell proliferation	Regulation	We assessed the hypothesis that HSP60 is induced in vascular cells infected with C pneumoniae and stimulates cell proliferation.	info:pmid/14500333 #abs:5	Blood Vessels {Organ urn:agi-ncimorgan:C0005847}
HSPD1 --+> cell proliferation	Regulation	Adenoviral-mediated overexpression of HSP60 induced increases in cell proliferation compared with uninfected vascular smooth muscle cell.	info:pmid/23210434 #abs:6	

HSPD1 --+> cell proliferation	Regulation	HSP60 could promote DCs expressed CD86 and stimulate T lymphocytes proliferation in vitro, while Puerarin had significantly inhibitory effect.	info:pmid/17569333#abs:8	
HSPD1 --+> cell proliferation	Regulation	Altered peptide ligands of p1-20 can inhibit p1-20/hsp65-induced proliferation of DR3-restricted T cells in an allele specific manner in vitro.	info:pmid/9559973#abs:3	
HSPD1 --+> cell proliferation	Regulation	The results show that exogenous bacterial hsp60 is able to activate ERK1/2 phosphorylation and thereby cause increased epithelial proliferation.	info:pmid/11339820#abs:12	
HSPD1 --+> cell proliferation	Regulation	This proliferation was markedly augmented by hsp65 (3-fold) and hsp70 (5-fold), whereas hsp10 and the protein control ovalbumin had no effect.	info:pmid/7878764#abs:6	
HSPD1 --+> cell proliferation	Regulation	Recombinant human HSP60 protein could increase the proliferation rate and the colony formation ability of PcDNA3.1(IGFBP7)-RKO cells.	info:pmid/20433702#abs:8	
HSPD1 --+> cell proliferation	Regulation	Although hsp65 and hsp18 induced similar proliferation in multibacillary patients, a high proportion of these patients did not generate cytotoxic effector cells in response to hsp65.	info:pmid/7813115#abs:6	
HSPD1 --+> cell proliferation	Regulation	The role of Toll-like receptor-mediated stimulation of cell proliferation by HSP60 was supported by the significant increase in proliferation of transfected human embryonic kidney cells.	info:pmid/16843693#abs:12	
HSPD1 --+> cell proliferation	Regulation	Stimulation of T cells with peptides of 12 and 13 amino acid residues of Yersinia-hsp60 caused highly efficient proliferation compared with longer peptides, full-length recombinant Yersinia-hsp60, or heat-killed Yersinia .	info:pmid/8706327#abs:6	
HSPD1 --+> cell proliferation	Regulation	To explore probes that can be used for studying signal transduction elicited by the chlamydial Hsp60, we have tested several natural products for their inhibitory actions on the Hsp60-induced proliferation of rat arterial smooth muscle cells.	info:pmid/15453708#abs:4	
HSPD1 --+> cell	Regulation	These signaling molecules, including	info:pmid/20880502	Brain {Organ

proliferation		ATP, glutamate, cytokines, prostaglandins, zinc, reactive oxygen species, and HSP60, may induce microglial proliferation and migration to the sites of injury.	#abs:3	urn:agi-ncimorgan:C1269537}
HSPD1 --+> cell proliferation	Regulation	These data demonstrate that the expressions of iNOS, HSP60, and HSP70 are involved in inflammatory processes and might play a role in the activation and proliferation of lining epithelium, leading to progression of periapical inflammatory lesions.	info:pmid/12220357#abs:12	
HSPD1 --+> cell proliferation	Regulation	Further analyses of the C pneumoniae-reactive T-cell lines showed that chlamydial 60-kDa heat-shock protein induced specific proliferation in 5 (71%) of 7 cases and revealed 2 haplotype (DRB1*1502 and DQB1*06) binding motifs in human 60-kDa heat-shock protein.	info:pmid/10764674#abs:9	
HSPD1 --+> cell proliferation	Regulation	Compounds currently known to directly or indirectly affect Hsp60 functions, such as protein folding, HIF-1a accumulation, or Hsp60-induced cell proliferation, are discussed along with strategies that might prove effective for developing Hsp60-targeting drugs for anticancer therapy.	info:pmid/23092316#abs:6	
HSPD1 --+> cell proliferation	Regulation	At 110 and 180 days post-conception, we identified proteins such as TCP1, FKBP4, or HSPD1 that may regulate adipocyte precursor proliferation by controlling cell-cycle progression and/or apoptosis or delaying PPAR?-induced differentiation.	info:pmid/21678425#abs:5	
HSPD1 --+> cell proliferation	Regulation	The improvement in adjuvant arthritis with hsp60-VV administration was associated with a specific immune response, as determined by the presence of antibodies to hsp60 in the sera and the proliferation induced by hsp60 of T cells from popliteal lymph nodes.	info:pmid/8406810#abs:9	Popliteal lymph node group {Organ urn:agi-ncimorgan:C0588057}
HSPD1 --+> cell proliferation	Regulation	The over-expressed proteins including guanine nucleotide-binding protein, isocitrate dehydrogenase , annexin A2, heat shock protein 60 (HSP 60),	info:pmid/23982774#abs:7	Ocular orbit {Organ urn:agi-ncimorgan:C

		calreticulin (CALR), protein disulfide-isomerase A3 (PDIA3), spectrin, superoxide dismutase , and transitional endoplasmic reticulum ATPase (TER ATPase) may contribute to increased thyroid-stimulating hormone receptor (TSHR) expression and cell proliferation.		0029180}
HSPD1 --+> cell proliferation	Regulation	We have recently shown that hsp60 increased cell proliferation by 25-75% .	info:pmid/15194479#body:117	
HSPD1 --+> cell proliferation	Regulation	Human HSP60 (HSP) induces cell proliferation in cord blood.	info:pmid/21931651#cont:72	Umbilical Blood {Organ urn:agi-ncimorgan:C1550673}
HSPD1 --+> cell proliferation	Regulation	These results indicate that MCF and Cpn60 significantly contribute to sulfolipid synthesis and cell proliferation.	info:pmid/21829746#cont:145	
HSPD1 --+> cell proliferation	Regulation	Results IL-4 stimulates Mononuclear cells proliferation induced by human but not bacterial hsp60.	info:pmid/9218524#body:101	
HSPD1 --+> cell proliferation	Regulation	Figure 5 HSP60 positively regulates proliferation and survival of established and primary neuroblastoma cell lines.	info:pmid/22012253#cont:167	
HSPD1 --+> cell proliferation	Regulation	For example, mycobacterial Hsp65 is known to stimulate cellular proliferation in mouse and human ?d + T cells .	info:pmid/9987177#body:158	
HSPD1 --+> cell proliferation	Regulation	... , it has been shown that the increased expression of HSP27, HSP60, and HSP70 and the overexpression of these factors induces cell survival, proliferation ...	info:pmid/21995449#cont:237	
HSPD1 --+> cell proliferation	Regulation	Direct C. pneumoniae infection and treatment with Chlamydial heat shock protein 60 can induce vascular smooth muscle cells proliferation.	info:pmid/22214836#body:18	
HSPD1 --+> cell proliferation	Regulation	Rhodobacter sphaeroides diphosphoryl lipid A partially attenuates C pneumoniae-induced proliferation and abolishes hsp60-induced proliferation.	info:pmid/11485974#body:158	
HSPD1 --+> cell proliferation	Regulation	We also found that HSP60 induced a dose-dependent decrease in cell	info:pmid/19306954#body:118	

		proliferation and promoted a corresponding increase in apoptosis in osteoblast lineages.		
HSPD1 --+> cell proliferation	Regulation	When MF59-YW002 was used as an adjuvant, HSP65-MUC1 strongly induced the proliferation of CD8+ T cells as indicated by the presence of CD69+/CD8+ double positive cells (A).	info:pmid/22595192 #body:117	
HSPD1 --+> cell proliferation	Regulation	Placental HSP60 did not appear to influence the proliferation of lymphocytes or any other placental cells under any conditions tested.	info:pmid/21227506 #body:262	Placenta {Organ urn:agi-ncimorgan:C 1550656}
HSPD1 --+> cell proliferation	Regulation	Treatment with bacterial heat shock protein 60, a bacterial toxin, increases HaCaT cell p38 activity, ERK1/2 activity, and cell proliferation (Zhang L et al, 2001).	info:pmid/12713588 #body:94	
HSPD1 --+> cell proliferation	Regulation	It has been shown that the chlamydial HSP 60 alone causes proliferation of human vascular smooth muscle cells in a p44/42 mitogen-activated protein kinase-dependent manner in vitro.	info:pmid/22660918 #cont:104	
HSPD1 --+> cell proliferation	Regulation	That means that the synergetic inhibition induced by triptolide and artesunate combination was more likely due to the proliferation related pathway triggered by HSP20 and HSP27 but HSP60.	info:pmid/24175808 #cont:177	Breast {Organ urn:agi-ncimorgan:C 0006141}
HSPD1 --+> cell proliferation	Regulation	They found that serum-soluble HSP60 induced specific proliferation in 71% of the cases and revealed 2 haplotype (DRB1*1502 and DQB1*06) binding motifs in human serum-soluble HSP60.	info:pmid/12377729 #body:219	
HSPD1 --+> cell proliferation	Regulation	Four peptides from HSP65 and their homologous peptides from HSP60 identified by T cell epitope mapping were able to stimulate proliferation of ?dT cells in Behçet's disease .	info:pmid/22197900 #body:105	
HSPD1 --+> cell proliferation	Regulation	Loss of hspd1 function in temperature-sensitive mutant zebrafish significantly reduces proliferation after acute light lesion in Müller glia that express the retinal progenitor gene pax6 (; Section).	info:pmid/24412518 #body:266	
HSPD1 --+> cell proliferation	Regulation	The upregulation of p53, Ki67, heat shock proteins (HSP60) and connexins	info:pmid/23532439 #cont:107	

		(26 and 30) also contributes to epidermal hyperproliferation, even though a clear molecular mechanism needs to be established.		
HSPD1 --+> cell proliferation	Regulation	However, Rhodobacter sphaeroides lipid A was also able to block the proliferation of smooth muscle cells stimulated by HSP60 from C. pneumoniae, indicating that Rhodobacter sphaeroides lipid A is not specific for endotoxin .	info:pmid/12758273 #body:95	
HSPD1 --+> cell proliferation	Regulation	As shown in A , oral immunization with LL.PCYT:HSP65 or LL.PHJ:HSP65 significantly reduced the HSP65-specific proliferation of bulk splenocytes in treated groups as compared to that of control group.	info:pmid/21497632 #body:103	
HSPD1 --+> cell proliferation	Regulation	These data indicate that the expression of iNOS and HSP60 is involved in inflammatory processes and may play a role in the activation and proliferation of lining epithelium, leading to the progression of periapical inflammatory lesions [77].	info:pmid/22920899 #cont:203	
HSPD1 --+> cell proliferation	Regulation	Thus far, we showed that H. pylori Heat shock protein 60 enhanced endothelial cell migration and cell proliferation; however, the downstream molecules involved in H. pylori Heat shock protein 60-induced angiogenesis are still unknown.	info:pmid/20580690 #body:52	
HSPD1 --+> cell proliferation	Regulation	We believe this issue is of some interest because the possibility of modulating therapeutically the expression of HSP60 and HSP10 could theoretically allow to restore the normal functions of cells in the early stages of carcinogenesis, allowing control of tumor cell proliferation and improving patient survival.	info:pmid/12913740 #body:10	Large Intestine {Organ urn:agi-ncimorgan:C1268999}
HSPD1 --+> cell proliferation	Regulation	Furthermore, the fraction of Adenosine triphosphate synthase that co-localized with hsp60 into lipid rafts (not shown) suggest that hsp60/Adenosine triphosphate synthase interactions participate also in signaling, and thereby influence	info:pmid/21326874 #cont:194	

		Adenosine triphosphate synthase-dependent activation and proliferation of endothelial cells [25,26].		
HSPD1 --+> cell proliferation	Regulation	These observations seem to contradict miR-206s role as a tumor suppressor, but it must be remembered that microRNAs target multiple genes, and other miR-206 targets include NOTCH3, PAX7, and HSP60, which play roles in cell proliferation and motility . miR-27a indirectly regulates ERa expression by reducing ZBTB10, an Sp repressor, that is reducing Sp1, Sp3, and Sp4 ( ).	info:pmid/22503553#body:60	
HSPD1 --+> cell proliferation	Regulation	ucts such as sesamol, vanillyl alcohol, trans-ferulic acid, zerumbone, humulene, and caryophylene, (Fig. 2), showed a moderate to significant inhibitory activity of Hsp60-induced cell proliferation.	info:doi/10.2174/1381612811319150011#cont:142	
HSPD1 --+> cell proliferation	Regulation	Chlamydial HSP60 stimulates proliferation of human vascular smooth muscle cells via TLR4 and p44/p42 mitogen-stimulated protein kinase and up-regulates human endothelial cells through TLR4 and MD-2 in a MyD88-dependent pathway.	info:doi/10.1016/S0955-470X(02)00009-5#body:111	
HSPD1 --+> cell proliferation	Regulation	These observations seem to contradict miR-206s role as a tumor suppressor, but it must be remembered that microRNAs target multiple genes, and other miR-206 targets include NOTCH3, PAX7, and HSP60, which play roles in cell proliferation and motility . miR-27a indirectly regulates ERa expression by reducing ZBTB10, an Sp repressor, that is reducing Sp1, Sp3, and Sp4 ( ).	info:doi/10.1016/j.tem.2012.03.002#body:60	
PEBP1 ---| cell proliferation	Regulation	The RKIP gene may play a role in inhibiting cellular proliferation.	info:pmid/22373584#abs:11	
PEBP1 ---| cell proliferation	Regulation	Ectopic expression of RKIP altered hepatocellular carcinoma cell proliferation and migration.	info:pmid/17030190#abs:11	
PEBP1 ---| cell proliferation	Regulation	RKIP inhibition potentiated mitogen-induced proliferation in lung	info:pmid/21385176#abs:13	Lung {Organ urn:agi-

		fibroblasts.		ncimorgan:C 1278908}
PEBP1 ---| cell proliferation	Regulation	RKIP inhibits proliferation and transformation of myeloid cells and decreases transformation induced by mutant RAS.	info:pmid/22388727 #abs:9	
PEBP1 ---| cell proliferation	Regulation	RKIP is important in beta-cell proliferation, and its downregulation may play a role in islet neoplasia.	info:pmid/15349122 #abs:13	Islets of Langerhans {Organ urn:agi-ncimorgan:C 0022131}
PEBP1 ---| cell proliferation	Regulation	These results indicate that PEBP not only inhibits cell proliferation but also induces differentiation of human keratinocytes.	info:pmid/15155742 #abs:13	
PEBP1 ---| cell proliferation	Regulation	Further investigation indicated that RKIP inhibited ovarian cancer cell proliferation by altering cell cycle progression rather than promoting apoptosis.	info:pmid/18567796 #abs:8	
PEBP1 ---| cell proliferation	Regulation	Taken together, our in vitro and in vivo data demonstrate that RKIP modulates the proliferation, apoptosis, migration, invasion and tumorigenicity of SGC7901 cells.	info:pmid/23259789 #abs:9	
PEBP1 ---| cell proliferation	Regulation	We show that RKIP depletion can rescue the compromised ERK activation and promote proliferation, and this rescue occurs through a Raf-1 dependent mechanism.	info:pmid/23416466 #abs:5	
PEBP1 ---| cell proliferation	Regulation	As mlc2a is involved in heart morphogenesis, and PEBP controls the proliferation and differentiation of different cell types, these genes are candidates for involvement in Down syndrome-Congenital heart disease.	info:pmid/15063784 #abs:5	Heart {Organ urn:agi-ncimorgan:C 1281570}
PEBP1 ---| cell proliferation	Regulation	Raf kinase inhibitory protein inhibits beta-cell proliferation.	info:pmid/18379591 #body:226	
PEBP1 ---| cell proliferation	Regulation	We examined how RKIP inhibited Chronic myelogenous leukemia cell proliferation.	info:pmid/20028985 #body:265	
PEBP1 ---| cell proliferation	Regulation	Loss of Raf kinase inhibitor protein promotes cell proliferation and migration of human hepatoma cells.	info:pmid/21617351 #cont:126	
PEBP1 ---| cell proliferation	Regulation	Among these, the prostatic binding protein (PBP/RKIP) has a predominant	info:pmid/20577840 #cont:43	Umbilical Blood {Organ

		role in proliferation and homing regulatory events [29–31].		urn:agi-ncimorgan:C1550673}
PEBP1 ---| cell proliferation	Regulation	Loss of Raf kinase inhibitor protein was demonstrated to stimulate Hepatocellular carcinoma proliferation and migration .	info:pmid/20371362#body:196	head {Organ urn:agi-ncimorgan:C0018670}
PEBP1 ---| cell proliferation	Regulation	Raf kinase inhibitor protein inhibits cell proliferation but promotes cell migration in rat hepatic stellate cells.	info:pmid/19323783#title:1	
PEBP1 ---| cell proliferation	Regulation	In ovarian cancer cells, RKIP increased cellular proliferation, anchorage-independent growth, cell adhesion, and invasion when downregulated [14].	info:pmid/22460832#cont:113	
PEBP1 ---| cell proliferation	Regulation	In support of our results, another study showed that the restoration of PEBP1 could decrease Hepatocellular carcinoma cell proliferation and migration in vitro .	info:pmid/20739083#body:112	Liver {Organ urn:agi-ncimorgan:CL384198}
PEBP1 ---| cell proliferation	Regulation	As we expected, overexpression of RKIP could suppress the cell proliferation (Figure W4, A and B), whereas RKIP knockdown could promote the cell proliferation (Figure W4, A and C).	info:pmid/23814485#cont:223	
PEBP1 ---| cell proliferation	Regulation	SNAI1 expression is inversely correlated with RKIP (Raf kinase inhibitor protein), a metastatic suppressor protein that inhibits cell survival, proliferation and invasiveness through targeting Raf-1/MEK/ERK and NF-?B signaling pathways .	info:pmid/24565133#cont:197	Mesenchyme {Organ urn:agi-ncimorgan:C0162415}
PEBP1 ---| cell proliferation	Regulation	Phosphatidylethanolamine-binding protein has been demonstrated to bind to Raf-1 and mitogenactivated protein kinase kinase (MAPKK/MEK), components of the extracellular signal-regulated protein kinase pathway, thereby inhibiting the pathway and resulting in the suppression of cell proliferation .	info:pmid/17482186#body:145	Brain {Organ urn:agi-ncimorgan:C1269537}
PEBP1 ---| cell proliferation	Regulation	Lee HC, Tian B, Sedivy JM, Wands JR, Kim M. Loss of Raf kinase inhibitor protein promotes cell proliferation and migration of human hepatoma cells.	info:embase/2013266176#cont:302	

PEBP1 ---| cell proliferation	Regulation	phosphatidylethanolamine binding protein 1, a precursor for the undecapeptide hippocampus cholinergic neurostimulatory peptide ( ), is involved in the secretion of acetylcholine and regulates cell proliferation.	info:doi/10.1016/j.neuroscience.2012.01.048#body:117	Hippocampus {Organ urn:agi-ncimorgan:C0019564}
PKM --+> cell proliferation	Regulation	Knockdown of PKM2 attenuated PRL-stimulated cell proliferation.	info:pmid/20962042#abs:5	
PKM --+> cell proliferation	Regulation	Knockdown of PKM2 repressed proliferation and migration of the cells.	info:pmid/22807066#abs:6	
PKM --+> cell proliferation	Regulation	Acetylation-mimetic PKM2(K433) mutant promotes cell proliferation and tumorigenesis.	info:pmid/24120661#abs:5	
PKM --+> cell proliferation	Regulation	Additionally, PKM2-SAICAR was necessary to induce sustained Erk1/2 activation and mitogen-induced cell proliferation.	info:pmid/24606918#abs:7	
PKM --+> cell proliferation	Regulation	Expression of a PKM2 mutant that exists as a dimer promotes cell proliferation, indicating that protein kinase activity of PKM2 plays a role in promoting cell proliferation.	info:pmid/22306293#abs:8	
PKM --+> cell proliferation	Regulation	Cancer cells universally express the M2 isoform of the glycolytic enzyme pyruvate kinase (PKM2), and previous work has demonstrated that PKM2 expression is necessary for aerobic glycolysis and cell proliferation in vivo.	info:pmid/20005212#abs:3	
PKM --+> cell proliferation	Regulation	The prevalence of PKM2 in cancer cells relative to the prevalence of PKM1 in many normal cells, suggests a therapeutic strategy whereby activation of PKM2 may counter the abnormal cellular metabolism in cancer cells, and consequently decreased cellular proliferation.	info:pmid/22963766#abs:5	
PKM --+> cell proliferation	Regulation	Furthermore, the growth suppression effect of Cyclosporin A was impaired in MCF-7 cells when they were transfected with the PKM2 overexpression plasmid, suggesting that Cyclosporin A was an effective inhibitor of PKM2-dependent proliferation of breast cancer cells.	info:pmid/22580449#abs:7	
PKM --+> cell	Regulation	The nuclear functions of PKM2	info:pmid/23791887	

proliferation		contribute to cell proliferation.	#body:130	
PKM --+> cell proliferation	Regulation	Overall, it appears that PKM2 nuclear functions contribute to cell proliferation.	info:pmid/22626471 #body:114	
PKM --+> cell proliferation	Regulation	Thus, both cytosolic and nuclear PKM2 contribute to altered metabolism and proliferation in cancer.	info:pmid/24344305 #cont:32	
PKM --+> cell proliferation	Regulation	One possibility is that PKM2 loss changes the balance of proliferation and apoptosis.	info:pmid/24120138 #body:59	
PKM --+> cell proliferation	Regulation	Pyruvate kinase M2 promotes de novo serine synthesis to sustain mTORC1 activity and cell proliferation.	info:pmid/22509023 #title:1	
PKM --+> cell proliferation	Regulation	Pyruvate kinase M2 promotes de novo serine synthesis to sustain mTORC1 activity and cell proliferation.	info:pmid/24305570 #cont:482	
PKM --+> cell proliferation	Regulation	These results confirmed that PKM2 is critical for cancer metabolism and cellular proliferation.	info:pmid/22574221 #cont:126	
PKM --+> cell proliferation	Regulation	All of these studies suggest that pyruvate kinase M2 plays an important role in cell proliferation.	info:pmid/21526124 #cont:218	
PKM --+> cell proliferation	Regulation	These results indicate that EGFR-increased PKM2 expression is required for EGFR-promoted cell proliferation.	info:pmid/23123196 #body:142	
PKM --+> cell proliferation	Regulation	Inhibition of mTOR, glycolysis, and PKM2 suppresses cell proliferation and tumorigenesis.	info:pmid/21325052 #cont:185	
PKM --+> cell proliferation	Regulation	Once in the nucleus, PKM2 activates ß-catenin, leading to cell proliferation and tumorigenesis .	info:pmid/22617155 #body:151	
PKM --+> cell proliferation	Regulation	Recently, Christofk et al. found that PKM2 is necessary for aerobic glycolysis and cell proliferation in vivo .	info:pmid/21334407 #body:132	Fetus {Organ urn:agi-ncimorgan:C1305737}
PKM --+> cell proliferation	Regulation	This overexpression of PKM2 is involved in promoting proliferation and migration of some types of cancer cells (2, 5, 8).	info:pmid/23846818 #cont:39	
PKM --+> cell proliferation	Regulation	Additionally, PKM2 promotes de novo serine synthesis to stimulate mTORC1 activity and sustain cell proliferation .	info:pmid/23523716 #body:38	
PKM --+> cell proliferation	Regulation	Also, PKM2 inhibition by prolactin increases lactate content and stimulates proliferation in human cell lines (Varghese et al. 2010).	info:pmid/21985671 #cont:814	
PKM --+> cell	Regulation	These observations are consistent	info:pmid/22922757	

proliferation		with previous data showing that replacement of PKM2 with PKM1 impairs cell proliferation under low oxygen conditions8.	#cont:427	
PKM --+> cell proliferation	Regulation	Pyruvate kinase isoform M2 (PKM2) is thought to critically regulate aerobic glycolysis for supplying metabolic intermediates for tumor cell proliferation .	info:pmid/21130743 #body:91	
PKM --+> cell proliferation	Regulation	Thr-454 phosphorylation of PKM2 increases cancer cell proliferation. a, A549 cells were transfected with HA-tagged PKM2-WT or T454A.	info:pmid/24142698 #cont:266	
PKM --+> cell proliferation	Regulation	They show that activating PKM2 suppresses cell proliferation — but severely inhibiting this enzyme is known to have similar effects, and can induce tumour regression in mice8,9.	info:pmid/23018962 #cont:57	
PKM --+> cell proliferation	Regulation	Decreased PKM2 activity as a result of oncogenic signaling pathway activation is thought to contribute to tumor cell proliferation by enabling use of upstream glycolytic intermediates for biosynthesis .	info:pmid/22406683 #body:89	
PKM --+> cell proliferation	Regulation	These novel activators of PKM2 provide the necessary tool compounds to explore the hypothesis that PKM2 activation will ameliorate the Warburg effect, and thereby decrease cancer cell proliferation.	info:pmid/20451379 #body:122	
PKM --+> cell proliferation	Regulation	Furthermore, the most recent study showed that aside from its key role in tumor metabolism, PKM2 has the same function of protein kinase and can directly regulate cancer cell proliferation .	info:pmid/23880164 #body:16	
PKM --+> cell proliferation	Regulation	Re-expression of PKM2 in PKM2 knockdown cells was reported to promote cell proliferation under hypoxic conditions , suggesting a role of PKM2 in the adaptive hypoxia response.	info:pmid/24508027 #body:85	
PKM --+> cell proliferation	Regulation	In addition to its well-known role in glycolysis, PKM2 regulates proliferation and apoptosis of nontransformed cells in a cell-type-specific manner by largely unknown	info:pmid/22901803 #body:10	Fetus {Organ urn:agi-ncimorgan:C 1305737}

		mechanisms (; ;).		
PKM --+> cell proliferation	Regulation	Since we found that METH preferentially acts on proliferating neural progenitor cells, we next sought to confirm nitrotyrosination of pyruvate kinase M2 , a protein that mediates cell proliferation.	info:pmid/21708025#cont:168	
PKM --+> cell proliferation	Regulation	insight as to whether the predominant expression of PKM2 observed in tumors is necessary for cancer cell proliferation, we investigated the role of PKM2 in tumor cell growth and maintenance.	info:pmid/23267074#cont:38	
PKM --+> cell proliferation	Regulation	Nuclear PKM2 phosphorylated the transcription factor STAT3 using phosphoenolpyruvate as a phosphate donor, and activation of STAT3 by PKM2 stimulated transcription of the MEK5 gene to increase cell proliferation ( ) .	info:pmid/22824010#body:108	
PKM --+> cell proliferation	Regulation	Recent studies showed that shikonin represses tumor pyruvate kinase M2 (PKM2) activity, which inhibits the metabolic rates of glucose and lactate in cancer cells and, thereby, suppresses cancer cell proliferation ( ).	info:pmid/23562787#body:151	
PKM --+> cell proliferation	Regulation	Knockdown of PKM2 using RNA interference significantly impairs cell growth in tissue culture, inhibition of PKM2 with peptide aptamers inhibits cell proliferation, and PKM2 expression is necessary for both aerobic glycolysis and tumor growth in vivo (31,32).	info:pmid/22581080#cont:526	Fetus {Organ urn:agi-ncimorgan:C1305737}
PKM --+> cell proliferation	Regulation	PKM2 depletion largely reduced both basal and EGF-induced tumour cell proliferation (Fig. 1b) and blocked EGF-enhanced expression of cyclin D1 and c-Myc (Fig. 1c), which are known to be important regulators of cell proliferation and downstream genes of b-catenin transactivation8.	info:pmid/22056988#cont:18	
PKM --+> cell proliferation	Regulation	The results strongly support our hypothesis that protein kinase activity of PKM2 promotes tumor/cell proliferation.	info:doi/10.1016/j.molcel.2012.01.001#body:179	
PKM --+> cell	Regulation	Decreased PKM2 activity as a result of	info:doi/10.1016/j.se	

proliferation		oncogenic signaling pathway activation is thought to contribute to tumor cell proliferation by enabling use of upstream glycolytic intermediates for biosynthesis .	mcdb.2012.02.003#body:89	
PKM --+> cell proliferation	Regulation	This suggests that pharmacological activation of PKM2 to levels associated with PKM1 may inhibit cell proliferation as well as be a potential therapeutic strategy for cancer.	info:doi/10.1016/j.bmcl.2011.08.114#body:13	
PKM --+> cell proliferation	Regulation	This finding, for the first time, highlights the essential non-metabolic functions of PKM2 by a dual role that is essential in EGFR-promoted ß-catenin transactivation, tumor cell proliferation, and tumorigenesis.	info:doi/10.5732/cjc.013.10228#cont:74	
PRDX1 ---> cell proliferation	Regulation	In addition PRDX1 is involved in cell differentiation and proliferation, apoptosis and innate immunity.	info:pmid/17913528#abs:2	
PRDX1 ---> cell proliferation	Regulation	This indicates that OSF-3 plays an intrinsic role in the proliferation and/or differentiation of bone cells.	info:pmid/8089076#abs:5	
PRDX1 ---> cell proliferation	Regulation	Deficiency of Prx I impaired cell proliferation and anchorage-independent growth.	info:pmid/23186333#cont:171	
PRDX1 ---> cell proliferation	Regulation	This indicates that Prx-I plays a role in cell proliferation, which is coincident with our previous result.	info:pmid/17976536#body:113	
PRDX1 ---> cell proliferation	Regulation	PrxI possesses not only antioxidant activity, but also regulatory activity in associated cell proliferation .	info:pmid/11065135#body:66	Brain {Organ urn:agi-ncimorgan:C1269537}
PRDX1 ---> cell proliferation	Regulation	This might explain, at least in part, why Prx I inhibition significantly increased p53 expressions and impaired cell proliferation.	info:pmid/20732753#body:102	
PRDX1 ---> cell proliferation	Regulation	peroxiredoxin 1 in turn can inhibit medial edge epithelial apoptosis, proliferation, and differentiation.	info:pmid/22561679#body:86	
PRDX1 ---> cell proliferation	Regulation	It has been suggested that Prx I regulates cell proliferation and apoptosis by its interaction with oncogene products such as c-Abl.	info:pmid/19566940#body:73	
PRDX1 ---> cell proliferation	Regulation	Additionally, peroxiredoxin-1 is one of the antioxidant enzymes and is involved in cellular proliferation and differentiation .	info:pmid/18996230#body:133	

PRDX1 ---> cell proliferation	Regulation	It seems that Prx I, PDGF-A, and PDGFR- a participate in transformation and proliferation, but they are not associated with invasion.	info:pmid/18992915#body:87	
PRDX1 ---> cell proliferation	Regulation	On the basis of the analysis of the cell cycle, Prx I expression very likely affects cell proliferation by regulating the cell cycle.	info:pmid/16414373#body:136	
PRDX1 ---> cell proliferation	Regulation	Prx I has also been suggested to be a tumor suppressor by regulating cell proliferation and transformation via direct interaction with the oncogenes c-Abl and c-Myc.	info:pmid/12960165#body:422	
PRDX1 ---> cell proliferation	Regulation	In the ventral and dorsal iris in all time points, peroxiredoxin 1, a protein that has been shown to play a role in proliferation and to be expressed in melanosomes, was upregulated (Table 3) [23,24].	info:pmid/23378727#cont:149	Dorsum {Organ urn:agi-ncimorgan:C0460009}
PRDX1 ---> cell proliferation	Regulation	At this point, there is no evidence that the secreted form of Peroxiredoxin-1 plays any role in proliferation and we plan to address this by supplementing the media of cells with a Peroxiredoxin-1 neutralizing antibody or purified Peroxiredoxin-1 protein.	info:pmid/21343469#cont:707	
PRDX1 ---> cell proliferation	Regulation	Prx I gene activation by the phorbol ester O-tetradecanoylphorbol- 13-acetate, which primarily affects cellular proliferation and differentiation, is inhibited by the bacterial product lipopolysaccharide, which is a prototypical proinflammatory mediator.	info:pmid/18070609#body:182	
PRDX1 ---> cell proliferation	Regulation	Because proliferation-associated gene/NKEF-A was abundantly expressed in transformed and rapidly growing cells but was suppressed in quiescent cells, it was suggested that proliferation-associated gene/NKEF-A was important for cell proliferation.	info:pmid/8981042#body:9	
PRDX1 ---> cell proliferation	Regulation	Ectopic expression of HA-PrxI and -PrxII and oxidant-induced arrest Up-regulation of PrxI is thought to counteract the effects of enhanced oxidant production in tumor cells and thereby promote cell survival and	info:pmid/17145963#body:199	

		proliferation (Chang et al., 2005; Park et al., 2006).		
PRDX1 ---> cell proliferation	Regulation	Among the 27 candidate immunoglobulin G-binding proteins obtained, guanine nucleotide binding protein (G protein), beta polypeptide 2-like 1 (GNB2L1; RACK1) and RAN were involved in cellular growth, and PRDX1, an antioxidase, participated in cellular antioxidate and proliferation.	info:pmid/24309932 #cont:25	
PRDX1 ---> cell proliferation	Regulation	For example, overexpression of Prx I facilitates cell growth and proliferation by protecting them from oxidant-induced cell death, and Prx III is required for Myc-mediated rat fibroblast transformation and proliferation of breast cancer MCF7 cells (20).	info:pmid/21487000 #cont:231	
HNRNPK --+> cell proliferation	Regulation	We found that SET and/or hnRNPK protein accumulation increased cellular proliferation.	info:pmid/24508256 #abs:5	
HNRNPK --+> cell proliferation	Regulation	Finally, overexpression of hnRNP K in breast cancer cells significantly increased target c-myc promoter activity and c-Myc protein, hnRNP K protein levels, and enhanced breast cancer cell proliferation and growth in an anchorage-independent manner.	info:pmid/11121407 #abs:7	
HNRNPK --+> cell proliferation	Regulation	These data suggested that hnRNP K plays an important role in cell growth and proliferation.	info:pmid/20499280 #cont:173	
HNRNPK --+> cell proliferation	Regulation	HnRNP K is involved in switching from proliferation to (neuronal) differentiation .	info:pmid/19410666 #body:130	Spleen {Organ urn:agi-ncimorgan:C1278932}
HNRNPK --+> cell proliferation	Regulation	These results correlate well with a previous study demonstrating that downregulation of hnRNPK decreases cellular proliferation .	info:pmid/23170974 #cont:254	
HNRNPK --+> cell proliferation	Regulation	In contrast, overexpression of heterogeneous nuclear ribonucleoprotein K enhances breast cancer cell proliferation [32].	info:pmid/21799787 #cont:335	
HNRNPK --+> cell proliferation	Regulation	Furthermore, downregulation of heterogeneous nuclear ribonucleoprotein K significantly	info:pmid/19520192 #body:169	

		inhibits growth factor-independent proliferation and colony formation .		
HNRNPK --+> cell proliferation	Regulation	Interestingly, the hnRNPK is involved in the activation of the human c-Myc promoter and enhances cell proliferation and growth of breast cancer cells in an anchorage independent manner .	info:pmid/18424265 #body:176	Breast {Organ urn:agi-ncimorgan:C0006141}
HNRNPK --+> cell proliferation	Regulation	These reports and our results suggest that hnRNP K may promote cell proliferation and have a negative effect against the promotion of differentiation in vivo.	info:pmid/15671036 #body:345	Brain {Organ urn:agi-ncimorgan:C1269537}
HNRNPK --+> cell proliferation	Regulation	Likewise, expression of hnRNP K is upregulated in grade III breast cancer samples and appears to correlate with anchorage-independence and enhanced proliferation of breast cancer cells (Mandal et al., 2001).	info:pmid/12476304 #body:145	
HNRNPK --+> cell proliferation	Regulation	Interestingly, the hnRNPk, which is up-regulated in the current study, is thought to be involved in the activation of the human c-Myc promoter and enhances cell proliferation and growth of BC cells in an anchorage independent manner .	info:pmid/17996735 #body:139	
HNRNPK --+> cell proliferation	Regulation	In breast cancer cells, overexpression of hnRNP K enhances cell proliferation and anchorage-independent growth (Mandal et al, 2001), and in several states of enhanced cell proliferation, increased expression of this protein has also been found (Ostrowski and Bomsztyk, 2003).	info:pmid/19401687 #body:48	
HNRNPK --+> cell proliferation	Regulation	Additionally, there is evidence to show that hnRNP K upregulation is a cause rather than an effect of proliferation; overexpression of hnRNP K in breast cancer cells significantly increases cell proliferation and growth in an anchorage-independent manner (Mandal et al, 2001).	info:pmid/16953238 #body:223	
HNRNPK --+> cell proliferation	Regulation	More importantly, hnRNP K up-regulates multiple downstream genes, including eIF4E and c-Myc, through transcriptional and post-transcriptional regulation, increasing	info:pmid/22321252 #body:8	

		cell proliferation, anti-apoptosis, and metastasis, and thereby conferring a tumorigenic phenotype on cancer cells.		
HNRNPK --+> cell proliferation	Regulation	We found that hnRNP K not only functions as a translational regulator of human reticulocyte 15-lipoxygenase mRNA (Fig. 2) but also represses expression of c-Src (Figs. 5 and 6), an important regulator of cell cycle control, proliferation, and differentiation (44, 45).	info:pmid/18441016 #body:288	
HNRNPK --+> cell proliferation	Regulation	We also explored the functional significance of hnRNPK and found that hnRNPK knockdown inhibited proliferation, whereas hnRNPK overexpression rescued the effects of T-cell leukaemia 1 knockdown in hepatocellular carcinoma and HCT116 cells (see online supplementary figure S7).	info:pmid/24352616 #cont:302	
HNRNPK --+> cell proliferation	Regulation	More importantly, hnRNP K up-regulates multiple downstream genes, including eIF4E and c-Myc, through transcriptional and post-transcriptional regulation, increasing cell proliferation, anti-apoptosis, and metastasis, and thereby conferring a tumorigenic phenotype on cancer cells.	info:doi/10.1016/j.oraloncology.2012.01.005#body:8	
HNRNPA1 ---> cell proliferation	Regulation	Down regulation of hnRNP A1 expression by RNA interference inhibits the proliferation and migration of cancerous HepG2 cells, while overexpression of hnRNP A1 in normal HL-7702 cells increased the proliferation and migration of the cells.	info:pmid/23062008 #abs:5	
HNRNPA1 ---> cell proliferation	Regulation	The over-expression of hnRNP A1 could contribute to the maintenance of telomere repeats in cancer cells and allow enhanced cell proliferation.	info:pmid/16513142 #body:128	
HNRNPA1 ---> cell proliferation	Regulation	The shuttling of hnRNP A1 is subject to regulation and is thought to play a role in cell proliferation, survival, and differentiation of normal and transformed cells (10).	info:pmid/15738418 #body:49	

HNRNPA1 ---> cell proliferation	Regulation	Moreover, hnRNPA1 is associated with apoptosis resistance (Patry et al., 2003[Go]) and increased proliferation (He et al., 2005[Go]) in cancer cells, two important features of cancer cells.	info:pmid/17652158 #body:269	
HNRNPA1 ---> cell proliferation	Regulation	Evidence of a direct involvement of the Heterogeneous nuclear ribonucleoproteins in proliferation was provided by RNAi, reduction of hnRNP A1 and A2 significantly reduced the proliferation rate of Colo 16 cells .	info:pmid/16378690 #body:218	
HNRNPA1 ---> cell proliferation	Regulation	In addition, hnRNP A1 mRNA export activity is required for proliferation, survival, and tumorigenesis of acute phase Chronic myelogenous leukemia blasts and BCR/ABL + myeloid precursor cell lines ( ).	info:pmid/16286244 #body:14	
HNRNPA1 ---> cell proliferation	Regulation	As for the genes in the cell growth and proliferation category, the upregulation of SEL1L, HNRNPA1, GAS5, DDIT4/REDD1, MTSS1, ARID4A, ANK3, ARMET, GNE and PTPRK, either reduce or inhibit cell proliferation, and these genes are expressed when growth is retarded ( ).	info:pmid/22101062 #body:101	
HNRNPA1 ---> cell proliferation	Regulation	The shuttling activity of hnRNP A1 has been proposed to play a role in cell proliferation, survival, and differentiation of normal and transformed cells . hnRNP A1 is also implicated in postsplicing activities, such as mRNA export and cap-dependent and internal ribosome entry site-mediated translation .	info:pmid/22227431 #body:16	
YWHAG --+> cell proliferation	Regulation	14-3-3 gamma is stimulated by IL-3 and promotes cell proliferation.	info:pmid/19124748 #title:1	
YWHAG --+> cell proliferation	Regulation	Recent studies indicated that expression of 14-3-3 ? promotes cell proliferation .	info:pmid/20870266 #body:12	
YWHAG --+> cell proliferation	Regulation	Human 14-3-3 gamma protein results in abnormal cell proliferation in the developing eye of Drosophila melanogaster.	info:pmid/18194556 #title:1	Eye {Organ urn:agi-ncimorgan:C 1550636}
YWHAG --+> cell proliferation	Regulation	In addition, 14-3-3? induces resistance to apoptotic programmed cell death and promotes proliferation of IL-3-	info:pmid/22658894 #body:68	Lung {Organ urn:agi-ncimorgan:C

		dependent Ba/F3 cells .		1278908}
YWHAG --+> cell proliferation	Regulation	Recent studies demonstrated that up-regulation of 14-3-3? promotes cell survival and proliferation through activation of distinct signal pathways in hematopoietic progenitor cells .	info:pmid/23500129 #body:192	
YWHAG --+> cell proliferation	Regulation	receptor 4, and 14-3-3 protein gamma, are involved in promotion of inflammation, ROS production, cell proliferation, cardiovascular remodeling, neurodegeneration and tumor growth in vitro and in vivo through various mechanisms as reported previously (Table S1).	info:pmid/24386293 #cont:303	Aorta {Organ urn:agi-ncimorgan:C 1278934}
YWHAG --+> cell proliferation	Regulation	In astrocyte cell cultures 14-3-3? expression changed with time in contrast to that in cerebellar neuronal cultures . 14-3-3? is potentially involved in structural dynamics and proliferation via binding to phosphorylated GFAP and actin .	info:pmid/21920445 #body:39	Cerebellum {Organ urn:agi-ncimorgan:C 1268981}
OAT ---> cell proliferation	Regulation	In mammals, Ornithine d-aminotransferase has been shown to modulate cell proliferation by regulating intracellular ornithine concentrations.	info:pmid/20673832 #body:204	
CFL1 ---> cell proliferation	Regulation	Cofilin-1 plays roles in cell migration, proliferation and phagocytosis.	info:pmid/24023293 #abs:6	
CFL1 ---> cell proliferation	Regulation	In addition, n-cofilin is required for neuronal precursor cell proliferation and scattering.	info:pmid/15649475 #abs:7	
CFL1 ---> cell proliferation	Regulation	Conversely, up-regulation of CFL1 in NSCs increased proliferation, adhesion, invasion and expression of the markers but reduced apoptosis.	info:pmid/20713416 #abs:10	Ovary {Organ urn:agi-ncimorgan:C L384202}
CFL1 ---> cell proliferation	Regulation	The activity of cofilin, an actin-remodeling protein, is required for T lymphocyte activation with regard to formation of the immunological synapse, cytokine production, and proliferation.	info:pmid/16424196 #abs:1	
CFL1 ---> cell proliferation	Regulation	Thirteen proteins from several pathways (nucleoside diphosphate kinase A, stathmin, valosin-containing protein, annexin A1, dihydropyrimidinase-related protein-3, DJ-1 protein, glutathione S-	info:pmid/19156760 #abs:5	

		transferase P, lamin A/C, fascin, cofilin, vimentin, vinculin, and moesin) were differentially expressed and most have been shown to play a role in differentiation, migration, invasion, proliferation, apoptosis, drug resistance, or oncogenesis.		
CFL1 ---> cell proliferation	Regulation	Cell proliferation and cytokinesis is impaired in n-cofilin null macrophages.	info:pmid/22558315 #cont:167	
CFL1 ---> cell proliferation	Regulation	These results suggest that cofilin phosphorylation regulates both cell proliferation and axon growth.	info:pmid/15572110 #body:69	
CFL1 ---> cell proliferation	Regulation	Strikingly, depletion of CapZ and Cofilin clearly prevented contact inhibition of proliferation in center cells.	info:pmid/23954413 #body:90	
CFL1 ---> cell proliferation	Regulation	Cofilin-1 plays roles in cell proliferation, phagocytosis, chemotactic movement and macropinocytosis (18,19).	info:pmid/21894436 #cont:119	Pancreas {Organ urn:agi-ncimorgan:C 1278931}
CFL1 ---> cell proliferation	Regulation	The phosphorylation of cofilin may then promote actin polymerisation, leading to proliferation and cytoskeletal rearrangement.	info:pmid/24370186 #body:116	Periodontal Ligament {Organ urn:agi-ncimorgan:C 0031093}
CFL1 ---> cell proliferation	Regulation	Although the mechanisms by which Cofilin-1 may promote uncontrolled cell proliferation are still poorly understood it may possibly be due to an impairment of DNA repair capacity .	info:pmid/23770296 #body:274	
CFL1 ---> cell proliferation	Regulation	Nox1 plays a role in vascular smooth muscle cell migration, proliferation, and extracellular matrix production, effects that are mediated by cofilin (219).	info:pmid/23600794 #cont:94	
CFL1 ---> cell proliferation	Regulation	However, the genetic deletion of Cofilin in the nervous system reduces neuronal cell proliferation and migration but not neurite formation ( ).	info:pmid/23259946 #body:23	Nervous system {Organ urn:agi-ncimorgan:C 0027763}
CFL1 ---> cell proliferation	Regulation	Mutations in actin-depolymerizing factors/cofilins from different species	info:pmid/16360805 #body:4	

		have been associated with lethality (; ;), arrest in cell proliferation, and disorganized actin cytoskeletons ( ).		
CFL1 ---> cell proliferation	Regulation	Hence, ILK/ß-parvin/cofilin signaling contributed critically to the abundant filopodium-like protrusion display and rapid cell proliferation in the 3D "Matrigel on-top" cultures of multiple colonization-competent carcinoma cell types.	info:pmid/24035453#body:100	
CFL1 ---> cell proliferation	Regulation	While Dstn2/2 brains have a normal gross morphology, conditional deletion of Cfl1 in neuronal cells causes excessive differentiation, changes in cell proliferation, and migration defects, resulting in a lissencephaly phenotype [5].	info:pmid/21060807#cont:28	Brain {Organ urn:agi-ncimorgan:C1269537}
CFL1 ---> cell proliferation	Regulation	The knockdown of cofilin with small interference RNA attenuated the EGF-induced migration and proliferation of T24 cells.	info:doi/10.1016/j.juro.2010.02.645#body:16	
YWHAZ --+> cell proliferation	Regulation	It is concluded that 14-3-3? plays an important role in proliferation of AML cells and associates with BCL-2 and MCL-1 expression.	info:pmid/23998576#abs:13	
YWHAZ --+> cell proliferation	Regulation	Knockdown of YWHAZ expression using several specific siRNAs inhibited the proliferation, migration, and invasion of YWHAZ-overexpressing gastric cancer cells.	info:pmid/23422756#abs:7	Veins {Organ urn:agi-ncimorgan:C0042449}
YWHAZ --+> cell proliferation	Regulation	Using Co-immunoprecipitation, we demonstrated that 14-3-3zeta protein binds to NFkappaB, beta-catenin and Bcl-2, suggesting its involvement in cellular signaling, leading to proliferation of oral cancer cells.	info:pmid/17764575#abs:9	
YWHAZ --+> cell proliferation	Regulation	Depletion of 14-3-3? markedly increased apoptosis, reduced proliferation and receptor tyrosine kinase (HER2 and EGFR) signaling, and, importantly, reversed endocrine resistance.	info:pmid/21707964#abs:8	Endocrine system {Organ urn:agi-ncimorgan:C1280975}
YWHAZ --+> cell proliferation	Regulation	Taken together, our findings show that overexpression of 14-3-3? has a causal role in mammary tumorigenesis and progression, acting through miR-221 in cooperation with known	info:pmid/24197133#abs:8	

		oncogenic events to drive neoplastic cell proliferation.		
YWHAZ --+> cell proliferation	Regulation	14-3-3?, a novel androgen-responsive gene, is upregulated in prostate cancer and promotes prostate cancer cell proliferation and survival.	info:pmid/22904106 #title:1	
YWHAZ --+> cell proliferation	Regulation	To test the hypothesis that silencing of 14-3-3? suppresses hepatocellular carcinoma cell proliferation, we assessed expression of PCNA in si14-3-3?-transfected HepG2 cells during the first 4 days following transfection.	info:pmid/21334806 #body:76	
YWHAZ --+> cell proliferation	Regulation	Overexpression of miR-451 and downregulation of 14-3-3? expression in endocrine-resistant cells restored the effectiveness of tamoxifen to decrease cell proliferation, increase apoptosis and reduce activation of EGFR/HER2 signaling.	info:pmid/23344024 #cont:290	Endocrine system {Organ urn:agi-ncimorgan:C1280975}
YWHAZ --+> cell proliferation	Regulation	14-3-3z promotes breast cancer cell proliferation, survival and receptor tyrosine kinase (EGFR, HER2) activation, and protein kinase signaling while suppressing apoptosis, all of which support the progression to endocrine resistance.	info:pmid/21666713 #cont:483	Endocrine system {Organ urn:agi-ncimorgan:C1280975}
YWHAZ --+> cell proliferation	Regulation	In this study, a total of 71.7% of Pancreatic adenocarcinoma showed an advanced stage of disease with high expression of 14-3-3 zeta. 14-3-3 zeta has been proposed to be directly involved in proliferation and in cellular transformation . 14-3-3 zeta inhibits specifically stress-induced p38 and JNK signaling and the triggering of the apoptotic response.	info:pmid/24629487 #body:85	Pancreas {Organ urn:agi-ncimorgan:C1278931}
YWHAZ --+> cell proliferation	Regulation	Moreover, the up-regulation of prohibitin, a repressor of E2F-mediated DNA synthesis and cellular proliferation , may also contribute to the observed berberine-induced loss of viability in these cells, as may the observed down-regulation of 14-3-3 zeta, a positive regulator of cell proliferation, whose overexpression in mammary epithelial cells has been shown to disrupt apoptotic signaling	info:pmid/22522123 #body:156	

		by down-regulating p53 .		
YWHAZ --+> cell proliferation	Regulation	Moreover, the up-regulation of prohibitin, a repressor of E2F-mediated DNA synthesis and cellular proliferation , may also contribute to the observed berberine-induced loss of viability in these cells, as may the observed down-regulation of 14-3-3 zeta, a positive regulator of cell proliferation, whose overexpression in mammary epithelial cells has been shown to disrupt apoptotic signaling by down-regulating p53 .	info:doi/10.1016/j.jprot.2012.03.010#body:156	
PRDX2 ---> cell proliferation	Regulation	These findings indicate that peroxiredoxin2 is involved in the proliferation of androgen receptor-expressing prostate cancer cells by modulating androgen receptor activity.	info:pmid/21539911#abs:9	
PRDX2 ---> cell proliferation	Regulation	Prx II deficiency results in increased production of H2O2, enhanced activation of Platelet-derived growth factor receptor and phospholipase Cgamma1, and subsequently increased cell proliferation and migration in response to Platelet-derived growth factor.	info:pmid/15902258#abs:4	
PRDX2 ---> cell proliferation	Regulation	Prx II deletion enhances concanavalin A -induced splenocyte proliferation and mixed lymphocyte reaction activity of bone marrow-derived CD11c-positive dendritic cells to stimulate recipient splenocytes.	info:pmid/16290204#abs:8	Bone Marrow {Organ urn:agi-ncimorgan:C0005953}
PRDX2 ---> cell proliferation	Regulation	In addition, the administration of PEP-1-SOD1 and/or PEP-1-peroxiredoxin-2 ameliorated D-galactose-induced reductions of cell proliferation and neuroblast differentiation in the dentate gyrus and significantly reduced D-galactose-induced lipid peroxidation in the hippocampus.	info:pmid/23892988#abs:7	
PRDX2 ---> cell proliferation	Regulation	Rapamycin, AZD8055, and Torin-1 inhibit proliferation of MTT cells in vitro.	info:pmid/23307788#cont:125	
PRDX2 ---> cell proliferation	Regulation	These data suggest that knocking down Prx II in C6 alters cell proliferation.	info:pmid/18718523#body:236	

PRDX2 ---> cell proliferation	Regulation	Compared with rapamycin, PP242 and Torin1 impaired the proliferation of primary cells to a far greater degree [136,137].	info:pmid/20812900 #cont:277	
PRDX2 ---> cell proliferation	Regulation	Peroxiredoxin 2 plays an important role in regulating cell proliferation and has anti-apoptotic properties .	info:pmid/18503785 #body:149	
PRDX2 ---> cell proliferation	Regulation	As discussed above, Prx II negatively regulates growth factor signaling and, thus, also negatively regulates cell proliferation.	info:pmid/16290020 #body:99	
PRDX2 ---> cell proliferation	Regulation	Prx II deficiency results in increased production of H2O2, enhanced activation of the platelet-derived growth factor receptor and phospholipase C–g1, and ...	info:pmid/20919930 #cont:333	
PRDX2 ---> cell proliferation	Regulation	Previous studies have suggested that Prdx2 can regulate many cellular functions including cell proliferation and differentiation .	info:pmid/24125860 #body:139	Colorectal Region {Organ urn:agi-ncimorgan:C 1711309}
PRDX2 ---> cell proliferation	Regulation	The Prdx2 suppression by small interfering RNA inhibited proliferation of the isolated GCs and increased the apoptosis ratio.	info:pmid/21248284 #cont:269	Ovarian Follicle {Organ urn:agi-ncimorgan:C 1283799}
PRDX2 ---> cell proliferation	Regulation	Prx II participates in regulation of cellular functions such as cell proliferation and differentiation, and protects a number of specific proteins from oxidative damage .	info:pmid/22749995 #body:5	
PRDX2 ---> cell proliferation	Regulation	PRDX2 suppression by small interfering RNA in granulosa cells of mouse ovary inhibits cell proliferation by augmenting H2O2 production (46).	info:pmid/22989627 #cont:315	Ovary {Organ urn:agi-ncimorgan:C L384202}
PRDX2 ---> cell proliferation	Regulation	The proliferation of MNNG/HOS cells was significantly reduced at 96h after transfection with si-PRDX2-3, while that of MG63 cells was significantly reduced at 72h.	info:pmid/23911960 #body:145	
PRDX2 ---> cell proliferation	Regulation	The Prx II knockdown enhanced the proliferation and chemotactic migration of human aortic SMCs in response to platelet-derived growth factor stimulation, which were	info:pmid/23820076 #cont:629	Aorta {Organ urn:agi-ncimorgan:C 1278934}

		attenuated by the gliotoxin pretreatment in a dose-dependent manner (Figure 5A).		
PRDX2 ---> cell proliferation	Regulation	Peroxiredoxin 2 deficiency can result in increased H2O2 production, enhanced activation of platelet-driven growth factor receptor, and subsequently increased cell proliferation and migration in the vascular remodelling process (28).	info:pmid/17982696#cont:283	Blood Vessels {Organ urn:agi-ncimorgan:C0005847}
PRDX2 ---> cell proliferation	Regulation	For example, Choi et al. (2005[Go]) have found that the deficiency of peroxiredoxin II, a cellular peroxidase, results in increased production of H2O2, enhanced activation of PDGF receptor, and subsequently increased cell proliferation and migration in response to PDGF (Choi et al., 2005[Go]).	info:pmid/16391241#body:321	
PRDX2 ---> cell proliferation	Regulation	The physiological significance of these events was recently supported by the demonstration that depletion of reactive oxygen species-scavenging peroxiredoxin II enhances platelet-derived growth factor-receptor-induced proliferation, and concomitantly increases platelet-derived growth factor ß-receptor phosphorylation and reduces protein tyrosine phosphatase activity.23 .	info:pmid/16990553#body:217	
ENO1 ---> cell proliferation	Regulation	This study suggested that exogenous expression of MBP-1 induces cell death in fibroblasts by blocking cell proliferation.	info:pmid/8519685#abs:7	
ENO1 ---> cell proliferation	Regulation	We found that cell proliferation was inhibited by MBP-1 overexpression in human stomach adenocarcinoma SC-M1 cells.	info:pmid/19846662#abs:5	
ENO1 ---> cell proliferation	Regulation	Overexpressed ENO1 not only restored cell proliferation and cell-cycle progression, but also antagonized the regulation of NESG1 to cell-cycle regulators p21 and CCNA1 expression as well as induced the expression of C-Myc, pRB, and E2F1 in NESG1-ovexpressed nasopharyngeal carcinoma cells.	info:pmid/22997098#abs:5	

ENO1 ---> cell proliferation	Regulation	ENO1 overexpression promoted cell proliferation, migration, invasion and tumourigenesis.	info:pmid/20435467#body:151	Pancreas {Organ urn:agi-ncimorgan:C1278931}
ENO1 ---> cell proliferation	Regulation	ENO1, PIN1, GLUL and BCL2L1 are involved in mitogenesis, proliferation and apoptosis.	info:pmid/16466929#body:165	Brain {Organ urn:agi-ncimorgan:C1269537}
ENO1 ---> cell proliferation	Regulation	Furthermore, exogenous a-enolase expression promoted cell proliferation, migration, invasion, and tumorogenesis [105].	info:pmid/23118496#cont:198	
ENO1 ---> cell proliferation	Regulation	Recently, we have shown that exogenous expression of MBP-1 in androgen-independent prostate cancer cells inhibits cell proliferation (23).	info:pmid/15805119#body:87	
ENO1 ---> cell proliferation	Regulation	In addition, the results indicated that enolase-1 promoted cell proliferation, although the effect was not evident under normoxic conditions.	info:pmid/23381546#cont:315	
ENO1 ---> cell proliferation	Regulation	Recently, we determined that both expression and function of MBP-1 are regulated by alterations in exogenous glucose concentrations and correspond to changes in cell proliferation and lactate production [26].	info:pmid/20412594#body:255	
ENO1 ---> cell proliferation	Regulation	Thus, one possibility may be that the mutant SEDLINs that are associated with SEDT and lead to a loss of interaction with MBP1 may disrupt the tight control between proliferation and apoptosis in endochondral ossification.	info:pmid/20498720#body:230	
ENO1 ---> cell proliferation	Regulation	Exogenous MBP-1 expression inhibits the growth of breast tumors in nude mice , induces cell death in neuroblastoma cells , suppresses proliferation in non-small-cell lung cancer cells , and induces G0–G1 growth arrest in chronic myeloid leukemia cells .	info:pmid/23421821#cont:19	
ENO1 ---> cell proliferation	Regulation	In this study, expression of TPI-1 and ENO1, which enhances cancer cell survival and proliferation, were higher	info:pmid/23504277#cont:380	Cardiovascular system {Organ

		in metastatic tumors than in primary tumors, and TAGLN2, which suppresses invasion of cancer cells, was lower in metastatic tumors than in primary tumors.		urn:agi-ncimorgan:C1269562}
ENO1 ---> cell proliferation	Regulation	In conclusion, results to date suggest that the differential expression of 14-3-3 protein a/ß, Cullin homolog 3, a-enolase and ezrin in H. pylori infection may play an important role in gastric carcinogenesis, including cell proliferation and cell adhesion, and that they may be induced by reactive oxygen species-mediated cell signaling.	info:pmid/15147740#body:139	Gastric mucosa {Organ urn:agi-ncimorgan:C0017136}
PGK1 ---> cell proliferation	Regulation	PGK1 modulates U251 cell proliferation.	info:pmid/24284928#cont:116	
PGK1 ---> cell proliferation	Regulation	Besides PGK1 gene, concomitant up-regulation of FGF1, FGF2, IL6, MUC1, and platelet-derived growth factor alpha polypeptide genes in F28/KMUH Cancer-associated fibroblasts may also be the explanations for Hepatocellular carcinoma cells to promote proliferation of F28/KMUH Cancer-associated fibroblasts and thus promote cancer progression.	info:pmid/23684136#body:79	
PGK1 ---> cell proliferation	Regulation	Besides PGK1 gene, concomitant up-regulation of FGF1, FGF2, IL6, MUC1, and platelet-derived growth factor alpha polypeptide genes in F28/KMUH Cancer-associated fibroblasts may also be the explanations for Hepatocellular carcinoma cells to promote proliferation of F28/KMUH Cancer-associated fibroblasts and thus promote cancer progression.	info:doi/10.1016/j.kjms.2012.08.012#body:79	
YWHAE ---> cell proliferation	Regulation	14-3-3epsilon regulates a wide range of biological processes, including cell cycle control, proliferation, and apoptosis, and plays a significant role in neurogenesis and the formation of malignant tumours.	info:pmid/20565895#abs:1	
YWHAE ---> cell proliferation	Regulation	YWHAE is involved in neuronal migration, proliferation, and cognitive impairment ( ).	info:pmid/23892282#body:169	Prosencephalon {Organ urn:agi-ncimorgan:C

				0085140}
PSMB6 --+> cell proliferation	Regulation	Knockdown of PSMB6 using siRNA also prevented hypoxia-induced proliferation.	info:pmid/23844134 #abs:10	Pulmonary artery {Organ urn:agi-ncimorgan:C 0034052}
ATP5A1 ---> cell proliferation	Regulation	An antibody against the ATP synthase a-subunit inhibited proliferation, migration and invasion in these breast cancer cells but not that of a non-tumor derived breast cell line.	info:pmid/22152132 #abs:10	
ATP5A1 ---> cell proliferation	Regulation	The identified copy number amplification genes, such as ZNF521, RNF138, RAB12, ATP5A1, PTPN2 and CTAGE1, are involved in multiple cellular processes, including transcription, proliferation, differentiation, migration and immunity.	info:pmid/23678296 #cont:270	Fetus {Organ urn:agi-ncimorgan:C 1305737}
ATP5A1 ---> cell proliferation	Regulation	Mitochondrial ATP synthase inhibitors antagonize 5-Fluorouracil-induced suppression of cell proliferation.	info:pmid/15833846 #body:183	
ATP5A1 ---> cell proliferation	Regulation	These findings indicate that the modulation of mitochondrial ATP synthase activity via expression of mutant or wild-type MTATP6 from the nucleus can affect cell proliferation and override the effect of the mitochondrial DNA genotype.	info:pmid/15753359 #body:256	
CKB ---> cell proliferation	Regulation	It was found that Creatine kinase B knockdown inhibited Skov3 cell proliferation and induced apoptosis under hypoxia or hypoglycemia conditions.	info:pmid/23416112 #abs:5	
CKB ---> cell proliferation	Regulation	Furthermore, overexpression of CFP-tagged wild-type Creatine kinase brain in Caco-2 colon cancer cells dramatically increased the number of cells in G2/M but had little effect on cell proliferation.	info:pmid/21308735 #abs:7	
