# Supplementary material for: Deciphering the Role of Emx1 in Neurogenesis: A Neuroproteomics Approach
Source: Front Mol Neurosci. 2016 Oct 17;9:98. doi: 10.3389/fnmol.2016.00098 (PMC5065984; doi:10.3389/fnmol.2016.00098)
Supplement: Supplementary file 6 [file Table6.pdf]

Table 6a: cytoskeleton organization Cluster

Entity Table:

Name	Type	Description	Connectivity	Local Connectivity	Indegree
PGK1	Protein	phosphoglycerate kinase 1	155	1	0
YWHAB	Protein	tyrosine 3-monooxygenase/tryptophan 5-monooxygenase activation protein, beta polypeptide	251	1	0
CNN3	Protein	calponin 3, acidic	55	1	0
CFL1	Protein	cofilin 1 (non-muscle)	920	1	0
RAP2A	Protein	RAP2A, member of RAS oncogene family	63	1	0
PEBP1	Protein	phosphatidylethanolamine binding protein 1	384	1	0
YWHAZ	Protein	tyrosine 3-monooxygenase/tryptophan 5-monooxygenase activation protein, zeta polypeptide	557	1	0
cytoskeleton organization and biogenesis	Cell Process		1618	7	7

Table 6b: cytoskeleton organization Cluster

Relationship Table:

Relation	Type	Sentence	TextRef	Connectivity	# of References	Organ
YWHAB ---> cytoskeleton organization and biogenesis	Regulation	Moreover, 14-3-3ß stimulated Ras-related C3 botulinum toxin substrate 1 -p21-activated kinase signaling to regulate Akt-mediated cytoskeletal organization, lamellipodia formation, and fibronectin matrix assembly.	info:pmid/21967815 #body:165	2	1	
CFL1 ---> cytoskeleton organization and biogenesis	Regulation	Cytoskeletal reorganization is partially mediated through cofilin, an actin assembly regulatory protein., Actin depolymerizing factor /cofilin family proteins are key regulators of actin filament turnover and cytoskeleton reorganization., tropomyosin1-induced cytoskeletal reorganization appears to be mediated through preventing cofilin interaction with microfilaments., These results suggest that cofilin plays critical roles in phagocytic functions through changes in cytoskeletal organization., Immunofluorescence and flow cytometry suggest that ouabain-induced active form of cofilin may be involved in cytoskeletal reorganization and cell volume regulation. <more data available...>	info:pmid/16337627 #abs:1, info:pmid/17583572 #abs:1, info:pmid/14722123 #abs:10, info:pmid/10430174 #abs:10, info:pmid/16713181 #abs:6, info:pmid/21693774 #abs:2, info:pmid/22450169 #abs:8, info:pmid/9224633#abs:9, info:pmid/16757346 #body:125, info:pmid/22446847 #cont:194 <more data available...>	2	42	Uterus {Organ urn:agi-ncimorgan:C1269032} , Vertebral column {Organ urn:agi-ncimorgan:C1267072} , Periodontal Ligament {Organ urn:agi-ncimorgan:C0031093} , Retina {Organ urn:agi-ncimorgan:C1962966} , Eye {Organ urn:agi-ncimorgan:C1550636} , Epidermis {Organ urn:agi-ncimorgan:C0014520} , Intestines {Organ urn:agi-ncimorgan:C0021853} , Veins {Organ urn:agi-ncimorgan:C0042449}
PGK1 --->	Regulation	We propose that the interaction between	info:pmid/18499456	2	4	

cytoskeleton organization and biogenesis		activated CED-10/Rac1 and MIG-10/lamellipodin triggers local cytoskeletal assembly and polarizes outgrowth activity in response to UNC-6/netrin., Therefore, MIG-10 would act downstream of Rac during axon guidance, and the data suggest that MIG-10/CED-10 interaction triggers local cytoskeletal assembly and polarizes Unc-6-dependent outgrowth-promoting activity., A later event in UNC-40 signaling in HSN is the ventral localization of MIG-10 (Lamellopodin), a ras-association/pleckstrin homology domain protein that modulates actin dynamics and promotes UNC-40-dependent cytoskeletal remodeling ( ). <more data available...>	#abs:9, info:pmid/22555291 #body:160, info:pmid/20627077 #body:120, info:doi/10.1016/j.cub.2008.04.050#body:79			
CNN3 ---> cytoskeleton organization and biogenesis	Regulation	In the present study, CNN3 was shown to participate in the cytoskeletal reorganization necessary, Obviously, CNN3 is involved in the cytoskeletal rearrangement phase and endows the plasma membrane with flexibility and mixing properties in both myoblasts and trophoblasts.	info:pmid/20861310 #cont:244, info:pmid/23276748 #body:219	2	2	
RAP2A ---> cytoskeleton organization and biogenesis	Regulation	However, in this context it is interesting to note that RAP2A, which is known to regulate cytoskeletal rearrangements, is a downstream target of NEDD4 and that knockdown of NEDD4 in neural cells leads to changes in the actin cytoskeleton .	info:pmid/22974840 #body:135	2	1	
PEBP1 ---> cytoskeleton organization and biogenesis	Regulation	Alternatively, it is also possible that RKIP plays a discrete role in cytoskeletal organization and migration that counteracts the effects of locostatin., In summary, either individually or, more likely, collectively, the accumulation of isoaspartyl residues in MAP-2, PEBP, UCHL1, stathmin, tubulin, and the microtubule-associated protein tau, would be expected to promote the disorganized cytoskeletal assembly that has been observed in PIMT knock-out mouse brains (14, 17).	info:pmid/19551145 #body:262, info:pmid/16923807 #body:294	2	2	Brain {Organ urn:agi-ncimorgan:C1269537}
YWHAZ ---> cytoskeleton	Regulation	Cytohesin-1 and 14-3-3? are also involved in cytoskeletal remodelling induced by the ß2	info:pmid/22458844 #cont:617,	2	2	

organization and biogenesis		integrins., On the other hand, the data from another group indicated that binding of 14-3-3? to GPIba inhibited platelet spreading on VWF surface, while disruption of 14-3-3? interaction with GPIba increased integrin-induced cytoskeletal reorganization [16].	info:pmid/22754302 #cont:30			

Table 6c: cytoskeleton organization Cluster

Reference Table:

Relation	Type	Sentence	TextRef	Organ
YWHAB ---> cytoskeleton organization and biogenesis	Regulation	Moreover, 14-3-3ß stimulated Ras-related C3 botulinum toxin substrate 1 -p21-activated kinase signaling to regulate Akt-mediated cytoskeletal organization, lamellipodia formation, and fibronectin matrix assembly.	info:pmid/21967815#body:165	
CFL1 ---> cytoskeleton organization and biogenesis	Regulation	Cytoskeletal reorganization is partially mediated through cofilin, an actin assembly regulatory protein.	info:pmid/16337627#abs:1	
CFL1 ---> cytoskeleton organization and biogenesis	Regulation	Actin depolymerizing factor /cofilin family proteins are key regulators of actin filament turnover and cytoskeleton reorganization.	info:pmid/17583572#abs:1	
CFL1 ---> cytoskeleton organization and biogenesis	Regulation	tropomyosin1-induced cytoskeletal reorganization appears to be mediated through preventing cofilin interaction with microfilaments.	info:pmid/14722123#abs:10	
CFL1 ---> cytoskeleton organization and biogenesis	Regulation	These results suggest that cofilin plays critical roles in phagocytic functions through changes in cytoskeletal organization.	info:pmid/10430174#abs:10	
CFL1 ---> cytoskeleton organization and biogenesis	Regulation	Immunofluorescence and flow cytometry suggest that ouabain-induced active form of cofilin may be involved in cytoskeletal reorganization and cell volume regulation.	info:pmid/16713181#abs:6	
CFL1 ---> cytoskeleton organization and biogenesis	Regulation	The actin-binding protein cofilin is involved in regulation of actin dynamics by promoting actin branching and cytoskeleton reorganization.	info:pmid/21693774#abs:2	Uterus {Organ urn:agi-ncimorgan:C1269032}
CFL1 ---> cytoskeleton organization and biogenesis	Regulation	It is hypothesized that phosphorylated HspB6 might affect interaction of 14-3-3 with protein phosphatases (and/or protein kinases) involved in dephosphorylation (or phosphorylation) of cofilin and by this means regulate cofilin-dependent reorganization of cytoskeleton.	info:pmid/22450169#abs:8	
CFL1 ---> cytoskeleton organization and biogenesis	Regulation	These data suggest that both cofilin (an actin-regulatory protein) and vimentin (an intermediate filament) may be key components of the cytoskeletal reorganization that mediates muscle cell development and adult skeletal-muscle repair.	info:pmid/9224633#abs:9	
CFL1 ---> cytoskeleton organization and biogenesis	Regulation	Cofilin regulates cytoskeletal rearrangement by depolymerizing actin ( ).	info:pmid/16757346#body:125	
CFL1 ---> cytoskeleton organization and biogenesis	Regulation	As discussed above, oxidation of cofilin can cause an abnormality in cytoskeleton reorganization and mitochondria.	info:pmid/22446847#cont:194	
CFL1 --->	Regulation	CFL1 and destrin are both actin-depolymerizing	info:pmid/23064469#cont:414	

cytoskeleton organization and biogenesis		proteins and are involved in the organization of the cytoskeleton.		
CFL1 ---> cytoskeleton organization and biogenesis	Regulation	This mechanism may be important in cells, where cofilin is essential for actin filament dynamics and cytoskeleton reorganization.	info:pmid/17134718#body:91	
CFL1 ---> cytoskeleton organization and biogenesis	Regulation	We further demonstrate activation of LIMK and cofilin, which are essential for regulating cytoskeletal organization.	info:pmid/15908432#body:115	
CFL1 ---> cytoskeleton organization and biogenesis	Regulation	Thus, cofilin is an integral component of cytoskeletal remodeling in spines and synaptic regulation.	info:pmid/22282498#cont:53	Vertebral column {Organ urn:agi-ncimorgan:C1267072}
CFL1 ---> cytoskeleton organization and biogenesis	Regulation	Thus, actin depolymerizing factor/cofilin has been shown to regulate migration and chemotaxis in various cell types by cytoskeleton remodeling .	info:pmid/22858003#body:166	
CFL1 ---> cytoskeleton organization and biogenesis	Regulation	The phosphorylation of cofilin may then promote actin polymerisation, leading to proliferation and cytoskeletal rearrangement.	info:pmid/24370186#body:116	Periodontal Ligament {Organ urn:agi-ncimorgan:C0031093}
CFL1 ---> cytoskeleton organization and biogenesis	Regulation	Phosphorylated cofilin is one of the major regulators of F-actin dynamics in spines, promotes cytoskeleton assembly and regulates spine morphology ( ).	info:pmid/21376239#body:20	Vertebral column {Organ urn:agi-ncimorgan:C1267072}
CFL1 ---> cytoskeleton organization and biogenesis	Regulation	Phosphorylated cofilin is one of the major regulators of F-actin dynamics in spines, promotes cytoskeleton assembly and regulates spine morphology ( ).	info:pmid/24632004#body:112	Vertebral column {Organ urn:agi-ncimorgan:C1267072}
CFL1 ---> cytoskeleton organization and biogenesis	Regulation	Overexpression of cofilin in Dictyostelium alters cytoskeletal organization, promotes membrane ruffling, and enhances cell migration ( ).	info:pmid/17338919#body:390	
CFL1 ---> cytoskeleton organization and biogenesis	Regulation	In summary, our studies demonstrate that [K +] e deprivation dephosphorylates cofilin and that dephosphorylated cofilin regulates cytoskeleton reorganization and cell volume.	info:pmid/16729967#body:59	
CFL1 ---> cytoskeleton organization and biogenesis	Regulation	Cofilin, an actin-binding protein, can polymerize actin and change the structure of cytoskeleton which is important in migration of cancer cells.	info:pmid/19649725#cont:81	
CFL1 ---> cytoskeleton organization and biogenesis	Regulation	Regulation of Actin depolymerizing factor/cofilin by phosphorylation/dephosphorylation or phosphoinositides is likely to be involved in cytoskeletal reorganization by intracellular signaling.	info:pmid/11901171#body:47	
CFL1 ---> cytoskeleton organization and biogenesis	Regulation	The actin-severing protein cofilin was shown to be essential for F-actin severing and cytoskeletal rearrangement that underlie B cell spreading [12].	info:pmid/23362305#cont:24	
CFL1 ---> cytoskeleton organization and biogenesis	Regulation	The possibility of local cofilin translation contributing to cytoskeletal remodelling is supported by the presence of cofilin transcripts in retinal axons.	info:pmid/16423696#body:148	Retina {Organ urn:agi-ncimorgan:C1962966}
CFL1 --->	Regulation	Slingshot cofilin phosphatase localization is	info:pmid/16169194#title:1	Eye {Organ

cytoskeleton organization and biogenesis		regulated by receptor tyrosine kinases and regulates cytoskeletal structure in the developing Drosophila eye.		urn:agi-ncimorgan:C1550636}
CFL1 ---> cytoskeleton organization and biogenesis	Regulation	Phosphorylation of cofilin at Ser3 through the RhoA/Rho kinase/LIM kinase (LIMK) pathway inhibits the severing of F-actin and induces cytoskeletal rearrangement ( ).	info:pmid/23337538#body:23	
CFL1 ---> cytoskeleton organization and biogenesis	Regulation	Examples of downstream effectors of Ca 2+ are Rho family GTPases ( ) and actin-depolymerization factor/cofilin ( ) that alter cytoskeletal organization for growth cone turning.	info:pmid/20471350#body:4	
CFL1 ---> cytoskeleton organization and biogenesis	Regulation	That is, a number of phosphatidylinositol 4,5-bisphosphate (PIP2)-binding proteins including gelsolin, cofilin, profilin, and a-actinin are known to bind to actin and regulate cytoskeletal assembly (1-4).	info:pmid/10364215#body:29	
CFL1 ---> cytoskeleton organization and biogenesis	Regulation	That is, a number of phosphatidylinositol 4,5-bisphosphate (PIP2)-binding proteins including gelsolin, cofilin, profilin, and a-actinin are known to bind to actin and regulate cytoskeletal assembly (1-4).	info:pmid/10364218#body:29	
CFL1 ---> cytoskeleton organization and biogenesis	Regulation	Several other candidate target proteins, namely actin, tropomyosin, and UNC-60/cofilin, are involved in cytoskeleton structure formation, suggesting that cytoskeleton function could be regulated by O-GlcNAcylation.	info:pmid/19940149#body:323	
CFL1 ---> cytoskeleton organization and biogenesis	Regulation	Panel B: Western blotting demonstrates that quercetin does not disrupt signaling through TGF-ß/SMAD or c-Abl pathways in NIH-3T3 cells, but instead affects cytoskeletal organization mediated by cofilin.	info:pmid/23819596#cont:222	
CFL1 ---> cytoskeleton organization and biogenesis	Regulation	First, cofilin may play an important role in the membrane movement of the activated cells, resulting in the formation of ruffled membranes or phagocytic vesicles through depolymerization of actin and remodeling of the cytoskeleton.	info:pmid/7642640#body:195	
CFL1 ---> cytoskeleton organization and biogenesis	Regulation	Moreover, it is postulated that phosphorylated HspB6 can displace cofilin from its complex with 14-3-3, thus leading to cofilin dephosphorylation causing rearrangement of cytoskeleton and regulation of smooth muscle contraction .	info:pmid/21081103#body:139	
CFL1 ---> cytoskeleton organization and biogenesis	Regulation	For the first time, the present study reported that expression level of BMP-2 in COS-7 cells genetically modified by plasmid transfection modulated the phosphorylation of LIMK1 and cofilin, which are thought to be involved in cytoskeletal remodeling.	info:pmid/22556173#cont:261	
CFL1 ---> cytoskeleton organization and biogenesis	Regulation	The signaling cascade initiated by FAK phosphorylation results in cytoskeletal rearrangements involving cofilin that interacts directly with actin; this interaction is apparently guided by proteins such as MAPK (mitogen-activated protein kinase)-p38 and HSP (heat shock protein)-27 .	info:pmid/20083150#body:24	
CFL1 ---> cytoskeleton organization	Regulation	The ubiquitous actin-binding protein, cofilin, is required for cytoskeleton formation, and it is indispensable for cell cycle control [ ], whereas	info:pmid/19740640#body:41	

and biogenesis		knockdown of p27(kip1) releases the G(1) phase arrest induced by overexpression of cofilin [ ].		
CFL1 ---> cytoskeleton organization and biogenesis	Regulation	During telophase, LIMK1 accumulates at the cleavage furrow, which is an actin/myosin-based structure , suggesting that LIMK1 regulates cytokinesis through cofilin phosphorylation involved in the actin cytoskeletal reorganization.	info:pmid/16455074#body:106	
CFL1 ---> cytoskeleton organization and biogenesis	Regulation	Cofilin is known to be primarily involved in cytoskeletal reorganization by depolymerizing actin, which influences cell migration and growth.29 Phosphorylation of cofilin at Ser3 inhibits its activity.15 The observed increase in cofilin phosphorylation suggests that persistent inactivation of cofilin may explain the impaired migration in NADPH oxidase1y/- cells.	info:pmid/19150879#body:211	
CFL1 ---> cytoskeleton organization and biogenesis	Regulation	In the presence of extracellular gradient of stimuli, CXCL12 or epidermal growth factors (EGF), human breast cancer cells respond with a polarized activation of PDK1/Akt2/PKC?, followed by cytoskeleton rearrangement mediated by LIMK/cofilin and an increase in cell adhesion mediated by integrin, resulting in directional migration .	info:pmid/21600875#body:4	Epidermis {Organ urn:agi-ncimorgan:C0014520}
CFL1 ---> cytoskeleton organization and biogenesis	Regulation	However, it was recently demonstrated that post-entry events, such as HIV-1 nuclear localization and integration, are promoted by a cofilin-mediated cytoskeletal reorganization dependent on the exposure of T cells to several chemokines (CCL19, CXCL9, CXCL10 and CCL20) that can synergize with the signaling pathways activated by the interactions between gp120, CD4 and CXCR4 .	info:pmid/21872676#body:61	
CFL1 ---> cytoskeleton organization and biogenesis	Regulation	Importantly, cofilin phosphorylation, which can contribute to cytoskeleton remodelling, was also Rho kinase dependent in our studies.	info:doi/10.1016/j.biocel.2011.10.014#body:158	Intestines {Organ urn:agi-ncimorgan:C0021853}
CFL1 ---> cytoskeleton organization and biogenesis	Regulation	These results indicate a role for phosphorylation of cofilin at the leading edge that is required for rearrangement of the cytoskeleton in myelinating OLs in response to estrogen stimulation.	info:doi/10.1016/j.neures.2009.09.347#body:5	Veins {Organ urn:agi-ncimorgan:C0042449}
PGK1 ---> cytoskeleton organization and biogenesis	Regulation	We propose that the interaction between activated CED-10/Rac1 and MIG-10/lamellipodin triggers local cytoskeletal assembly and polarizes outgrowth activity in response to UNC-6/netrin.	info:pmid/18499456#abs:9	
PGK1 ---> cytoskeleton organization and biogenesis	Regulation	Therefore, MIG-10 would act downstream of Rac during axon guidance, and the data suggest that MIG-10/CED-10 interaction triggers local cytoskeletal assembly and polarizes Unc-6-dependent outgrowth-promoting activity.	info:pmid/22555291#body:160	
PGK1 ---> cytoskeleton organization and biogenesis	Regulation	A later event in UNC-40 signaling in HSN is the ventral localization of MIG-10 (Lamellopodin), a ras-association/pleckstrin homology domain protein that modulates actin dynamics and promotes UNC-40-dependent cytoskeletal remodeling ( ).	info:pmid/20627077#body:120	
PGK1 ---> cytoskeleton	Regulation	MIG-10/lamellipodin can regulate actin polymerization, suggesting that MIG-10 might	info:doi/10.1016/j.cub.2008.04.050#body:79	

organization and biogenesis		mediate asymmetric cytoskeletal assembly in the growth cone.		
CNN3 ---> cytoskeleton organization and biogenesis	Regulation	In the present study, CNN3 was shown to participate in the cytoskeletal reorganization necessary	info:pmid/20861310#cont:244	
CNN3 ---> cytoskeleton organization and biogenesis	Regulation	Obviously, CNN3 is involved in the cytoskeletal rearrangement phase and endows the plasma membrane with flexibility and mixing properties in both myoblasts and trophoblasts.	info:pmid/23276748#body:219	
RAP2A ---> cytoskeleton organization and biogenesis	Regulation	However, in this context it is interesting to note that RAP2A, which is known to regulate cytoskeletal rearrangements, is a downstream target of NEDD4 and that knockdown of NEDD4 in neural cells leads to changes in the actin cytoskeleton .	info:pmid/22974840#body:135	
PEBP1 ---> cytoskeleton organization and biogenesis	Regulation	Alternatively, it is also possible that RKIP plays a discrete role in cytoskeletal organization and migration that counteracts the effects of locostatin.	info:pmid/19551145#body:262	
PEBP1 ---> cytoskeleton organization and biogenesis	Regulation	In summary, either individually or, more likely, collectively, the accumulation of isoaspartyl residues in MAP-2, PEBP, UCHL1, stathmin, tubulin, and the microtubule-associated protein tau, would be expected to promote the disorganized cytoskeletal assembly that has been observed in PIMT knock-out mouse brains (14, 17).	info:pmid/16923807#body:294	Brain {Organ urn:agi-ncimorgan:C1269537}
YWHAZ ---> cytoskeleton organization and biogenesis	Regulation	Cytohesin-1 and 14-3-3? are also involved in cytoskeletal remodelling induced by the ß2 integrins.	info:pmid/22458844#cont:617	
YWHAZ ---> cytoskeleton organization and biogenesis	Regulation	On the other hand, the data from another group indicated that binding of 14-3-3? to GPIba inhibited platelet spreading on VWF surface, while disruption of 14-3-3? interaction with GPIba increased integrin-induced cytoskeletal reorganization [16].	info:pmid/22754302#cont:30	
