# Supplementary material for: Deciphering the Role of Emx1 in Neurogenesis: A Neuroproteomics Approach
Source: Front Mol Neurosci. 2016 Oct 17;9:98. doi: 10.3389/fnmol.2016.00098 (PMC5065984; doi:10.3389/fnmol.2016.00098)
Supplement: Supplementary file 7 [file Table7.PDF]

Table 7a: Global Analysis Cluster

Entity Table:

Name	Type	Description	Connectivity	Local Connectivity	Indegree
ENO1	Protein	enolase 1, (alpha)	336	2	1
PGK1	Protein	phosphoglycerate kinase 1	155	3	0
PKM	Protein	pyruvate kinase, muscle	346	3	0
ATP5A1	Protein	ATP synthase, H+ transporting, mitochondrial F1 complex, alpha subunit 1, cardiac muscle	139	1	0
EMX1	Protein	empty spiracles homeobox 1	62	3	0
YWHAB	Protein	tyrosine 3-monooxygenase/tryptophan 5-monooxygenase activation protein, beta polypeptide	251	2	0
TUBA1A	Protein	tubulin, alpha 1a	54	1	0
HSPD1	Protein	heat shock 60kDa protein 1 (chaperonin)	988	2	0
CFL1	Protein	cofilin 1 (non-muscle)	920	4	1
HNRNPK	Protein	heterogeneous nuclear ribonucleoprotein K	412	1	0
VEGFA	Protein	vascular endothelial growth factor A	6086	17	7
MDK	Protein	midkine (neurite growth-promoting factor 2)	500	5	1
PEBP1	Protein	phosphatidylethanolamine binding protein 1	384	4	0
PRDX1	Protein	peroxiredoxin 1	388	3	0
PRDX2	Protein	peroxiredoxin 2	347	1	0
YWHAZ	Protein	tyrosine 3-monooxygenase/tryptophan 5-monooxygenase activation protein, zeta polypeptide	557	2	1
YWHAE	Protein	tyrosine 3-monooxygenase/tryptophan 5-monooxygenase activation protein, epsilon polypeptide	328	4	0
vascularization	Cell Process		3046	11	11
brain	Cell Process		1097	7	7

development				
neurogenesis	Cell Process	1405	7	7
synaptic transmission	Cell Process	1133	5	5
axon guidance	Cell Process	465	4	4
hippocampus morphology	ClinicalParameter	98	2	2

Table 7b: Global Analysis

Relationship Table:

Relation	Type	Sentence	TextRef	Connectivity	# of References	Organ
VEGFA --+> vascularization	Regulation	VEGF165 induces marked vascular growth., VEGF is a major inducer of angiogenesis., VEGF-A promotes angiogenesis in many tissues., VEGF is an important factor for angiogenesis., VEGF plays a central role in tumor angiogenesis., CRHR-2 inhibits VEGF-induced neovascularization., VEGF plays a principal role in tumor angiogenesis., VEGF was an important factor in tumor angiogenesis., VEGF plays a central role in angiogenesis in cancer., It inhibits VEGF-induced angiogenesis in preclinical models. <more data available...>	info:pmid/1384392#abs:4, info:pmid/19800880#abs:1, info:pmid/16453023#abs:1, info:pmid/14717227#abs:1, info:pmid/17615535#abs:4, info:pmid/18484196#abs:3, info:pmid/15064856#abs:3, info:pmid/12500403#abs:11, info:pmid/22992725#abs:1, info:pmid/22264850#abs:2 <more data available...>	2	122	Blood Vessels {Organ urn:agi-ncimorgan:C0005847}, Cornea, Eye
VEGFA --+> neurogenesis	Regulation	Vascular endothelial growth factor (VEGF) regulates neurogenesis., In addition, recent studies indicate that VEGF enhances neurogenesis after ischemia., Vascular endothelial growth factor (VEGF) enhances neurogenesis in ischemic brains.,	info:pmid/16269210#abs:1, info:pmid/21469168#abs:7, info:pmid/19834383#abs:1,	2	100	Brain {Organ urn:agi-ncimorgan:C1269537}, Hippocampus {Organ urn:agi-ncimorgan:C0019564}, Vascular system

		Furthermore, inhibition of VEGF expression by RNA interference completely blocked the environmental induction of neurogenesis., Recently, VEGF has also been proposed to play a role in neural development, neuroprotection, and adult neurogenesis., Importantly, this was done in settings that allowed the uncoupling of VEGF-promoted angiogenesis, neurogenesis, and memory. <more data available...>	info:pmid/15258583#abs:7, info:pmid/16472200#abs:8, info:pmid/14983237#abs:2, info:pmid/21385942#abs:4, info:pmid/20885857#abs:2, info:pmid/17618600#abs:2, info:pmid/20040492#abs:1 <more data available...>			{Organ urn:agi-ncimorgan:C0489903}, Nerve {Organ urn:agi-ncimorgan:C1280541}, Cerebrum {Organ urn:agi-ncimorgan:C1280654}, subventricular zone {Organ urn:agi-ncimorgan:C0521406}, Endocrine system {Organ urn:agi-ncimorgan:C1280975}, Prosencephalon {Organ urn:agi-ncimorgan:C0085140}, Blood capillaries {Organ urn:agi-ncimorgan:C0006901}, dentate gyrus {Organ urn:agi-ncimorgan:C0152314} <more data available...>
MDK --+> vascularization	Regulation	Both thymidine phosphorylase and midkine are important for angiogenesis in laryngeal squamous cell carcinoma., Moreover, MDK downregulates VEGF-A-induced neovascularization and vascular permeability in vivo., These results suggest that midkine may play important roles in malignant transformation and tumor angiogenesis in salivary gland tumors.,	info:pmid/18476626#abs:7, info:pmid/18392135#abs:5, info:pmid/20637680#abs:6, info:pmid/16832814#abs:9, info:pmid/2270756	2	39	Microvessels {Organ urn:agi-ncimorgan:C2350570}, Lung {Organ urn:agi-ncimorgan:C1278908}, Brain {Organ urn:agi-ncimorgan:C1269537

		Midkine small interfering RNA suppressed mainly cell proliferation and slightly angiogenesis, whereas paclitaxel enhanced apoptosis and slightly suppressed angiogenesis. <more data available...>	3#abs:6, info:pmid/18222265#abs:8, info:pmid/19152444#abs:5, info:pmid/9685995#abs:12, info:pmid/19698107#body:255, info:pmid/22051879#cont:200 <more data available...>			}, Mesenchyme {Organ urn:agi-ncimorgan:C0162415 }, Blood Vessels {Organ urn:agi-ncimorgan:C0005847 }, Heart {Organ urn:agi-ncimorgan:C1281570 }, Aorta {Organ urn:agi-ncimorgan:C1278934 }, Head of pancreas {Organ urn:agi-ncimorgan:C0227579 }
HSPD1 ---> vascularization	Regulation	Furthermore, Hsp60 from C. pneumoniae has been shown to promote the growth of vascular smooth muscle cells ., Furthermore, HSP65 also attenuated tumor-induced angiogenesis in the intradermal model and pulmonary metastasis in the tail intravenously injected model of mice., Secondly, HSP65-X10-ßhCGCTP37 may hamper angiogenesis via down-regulation of MMPs., Helicobacter pylori-derived Heat shock protein 60 enhances angiogenesis via a CXCR2-mediated signaling pathway., (2010) Helicobacter pylori-derived Heat shock protein 60 enhances angiogenesis via a CXCR2-mediated signaling pathway. <more data available...>	info:pmid/15453708#abs:3, info:pmid/23135134#abs:6, info:pmid/19913113#body:209, info:pmid/20580690#title:1, info:pmid/23555707#cont:596, info:pmid/22315307#cont:254, info:pmid/21330606#cont:212, info:pmid/21565524#body:6	2	8	Blood Vessels {Organ urn:agi-ncimorgan:C0005847 }
VEGFA --->	Regulation	These data suggest that VEGF and VEGFR-2	info:pmid/2008402	2	6	Prosencephalon

brain development		are likely involved in several aspects of human brain development., We show that VEGF produced by the embryonic neuroectoderm is required for the vascularization and the development of the brain., Most functions of VEGF that are essential for proper brain development are, in fact, dispensable in the adult brain as was clearly demonstrated using a conditional brain-specific VEGF loss-of-function approach., Both VEGF and reelin play important roles in neuronal migration during brain development . <more data available...>	1#abs:8, info:pmid/14983237#abs:4, info:pmid/23475068#abs:4, info:pmid/23916658#body:72, info:pmid/15571667#body:132, info:pmid/19118187#body:222			{Organ urn:agi-ncimorgan:C0085140}, Neuroectoderm {Organ urn:agi-ncimorgan:CL321642}, Brain {Organ urn:agi-ncimorgan:C1269537}
PEBP1 ---> neurogenesis	Regulation	Based on these previous data, HCNP/HCNP-pp might be involved in neurogenesis and/or gliogenesis in adult rat progenitor cells via novel mechanisms other than its function in inhibition of the Erk pathway.	info:pmid/20206149#body:87	2	1	Hippocampus {Organ urn:agi-ncimorgan:C0019564}
EMX1 ---> brain development	Regulation	Emx family homeobox genes, Emx1 and Emx2, play an essential role in rostral brain development in mammalian embryos., Transcription factors Emx1, 2, and Otx1, 2 can play roles in the rostral brain development., Like the fruit-fly counterpart, Emx1 and Emx2 are involved in brain development and are prevalently expressed in the cerebral cortex during embryogenesis between days E8.5 and E16.	info:pmid/12617801#abs:1, info:pmid/9113125#body:172, info:pmid/16197942#body:139	2	3	hindbrain {Organ urn:agi-ncimorgan:C0035507}, Cerebral cortex {Organ urn:agi-ncimorgan:C0007776}
MDK ---> neurogenesis	Regulation	Midkine ameliorates ischemic injury in the heart and brain, enhances oocyte maturation, and is involved in	info:pmid/24460672#abs:2, info:pmid/8537332	2	13	Brain {Organ urn:agi-ncimorgan:C1269537}, Neuroectoderm

		neurogenesis., Comparing the mode of Midkine expression between Xenopus and the mouse, we propose that Midkine plays evolutionally conserved roles in neurogenesis and development of the craniofacial architecture of ectomesenchymal origin., Midkine gene transfer protects against focal brain ischemia and augments neurogenesis., Midkine participates in cell growth, survival, migration, neurogenesis, and carcinogenesis. <more data available...>	#abs:7, info:pmid/19535098#title:1, info:pmid/18329695#body:3, info:pmid/19909807#body:146, info:pmid/16914133#body:11, info:pmid/10683378#body:55, info:pmid/15450683#body:1, info:pmid/23418741#cont:14, info:pmid/24372230#cont:25 <more data available...>			{Organ urn:agi-ncimorgan:CL321642}, Kidney {Organ urn:agi-ncimorgan:C1278978}, Peripheral Nerves {Organ urn:agi-ncimorgan:C0031119}, Central Nervous System {Organ urn:agi-ncimorgan:C0927232}
EMX1 ---> neurogenesis	Regulation	Our data suggest that deletion of the Emx1 gene reduces hippocampal neurogenesis and affects higher motor function that requires extensive learning., C, Examples of enhanced green fluorescent protein+ cells in granule cell layer labeled with bromodeoxyuridine, indicating that Emx1 lineage participates in adult neurogenesis.	info:pmid/17490651#abs:9, info:pmid/17596436#body:229	2	2	Upper Extremity {Organ urn:agi-ncimorgan:C1140618}
PRDX1 ---> neurogenesis	Regulation	Their gain-of-function analysis shows that, although misexpression of Prdx1 alone has little effect on neuronal development, Prdx1 synergistically promotes motor neuron differentiation when combined with doses of GDE2 too low to trigger neurogenesis.	info:pmid/19766560#body:17	2	1	
PEBP1 ---|	Regulation	Loss of RKIP enhanced both angiogenesis	info:pmid/1526959	2	7	Brain {Organ urn:agi-

vascularization		and vascular invasion, and protected against apoptosis., To evaluate the effect of RKIP-mediated tumor growth and angiogenesis in vivo, we performed a CAM assay., Reduced RKIP expression has been shown to affect cell growth, angiogenesis, apoptosis and gene integrity (8)., C) Hematoxylin-eosin staining of the paraffin embedded tumors showing the higher vascularization induced by RKIP inhibition., Loss of RKIP was associated with metastasis development, and increased angiogenesis and vascular invasion were suggested as possible mechanisms (18). <more data available...>	7#abs:10, info:pmid/22292035#cont:223, info:pmid/23674108#cont:27, info:pmid/23527098#cont:340, info:pmid/18230656#body:199, info:pmid/20855151#body:11, info:pmid/15686621#body:112			ncimorgan:C1269537 }, Prostate {Organ urn:agi-ncimorgan:C1278980 }, Breast {Organ urn:agi-ncimorgan:C0006141 }
VEGFA ---> synaptic transmission	Regulation	VEGF is a potent mitogen and survival factor for endothelial cells and neurons, and modulator of synaptic transmission., Our findings suggest that VEGF released from neuronal cells plays a local role in Ca2+ influx and synaptic transmission that may influence the generation of long-term changes in synaptic efficacy., VEGF also decreased synaptic transmission in slices from normal rats., VEGF also modulates synaptic transmission (12), suggesting that the effects of this factor are multifaceted., Also, increased VEGF gene expression facilitates excitatory synaptic transmission and promotes neuronal plasticity . <more data available...>	info:pmid/18061540#abs:4, info:pmid/18221855#abs:9, info:pmid/18065154#body:167, info:pmid/17360578#body:34, info:pmid/22985858#body:177, info:pmid/23076132#cont:122, info:pmid/20427666#body:429, info:pmid/22808185#cont:155, info:pmid/16192378#body:65, info:pmid/1959603	2	15	Hippocampus {Organ urn:agi-ncimorgan:C0019564 }, hypothalamus {Organ urn:agi-ncimorgan:C0020663 }, Vertebral column {Organ urn:agi-ncimorgan:C1267072 }, Brain {Organ urn:agi-ncimorgan:C1269537 }, Blood Vessels {Organ urn:agi-ncimorgan:C0005847 }

			8#body:129 <more data available...>			
CFL1 ---> synaptic transmission	Regulation	Because the phosphorylation levels and activities of LIMK and cofilin contribute to hippocampal excitatory synaptic transmission and plasticity ( ), fasudil hydrochloride might protect synaptic structure and function by inhibiting LIMK2 and cofilin phosphorylation.	info:doi/10.1016/j.neuroscience.2011.10.030#body:167	2	1	Hippocampus {Organ urn:agi-ncimorgan:C0019564 }
TUBA1A ---> brain development	Regulation	Interestingly, mutation analysis of the other tubulin genes that are also highly expressed in the developing nervous system, TUBA1B, TUBA1C and TUBB3, did not yield any deleterious mutations , suggesting that TUBA1A has a unique role in brain development, perhaps through interactions with its distinct protein-binding partners.	info:pmid/17997185#body:62	2	1	Nervous system {Organ urn:agi-ncimorgan:C0027763 }
YWHAE ---> synaptic transmission	Regulation	Taken together, it can be speculated that 14-3-3 epsilon is involved in synaptogenesis and/or synaptic transmissions and Cadmium possibly affects them through phosphorylation of 14-3-3 epsilon.	info:pmid/18835323#body:124	2	1	
PGK1 ---> vascularization	Regulation	These findings indicate that overexpression of PGK-1 in LLC-1 reduces the COX-2 expression, and, in turn, affect PGE2, cell invasion, angiogenesis, and the immune functions, and finally inhibit the tumor progression., Overexpression of phosphoglycerate kinase 1 reduced the secretion of vascular endothelial growth factor and decreased tumor angiogenesis., Phosphoglycerate kinase 1, which has not	info:pmid/18814280#abs:12, info:pmid/19299076#body:144, info:pmid/21647379#cont:164, info:pmid/15850924#body:16	2	4	

		been associated with the mitotic spindle previously, is essential for glycolysis and also prevents angiogenesis in tumors [61]. <more data available...>				
YWHAE ---| axon guidance	Regulation	We next compared 14-3-3e-dependent axon guidance defects to those resulting from manipulating Sema-1a/PlexA signaling.	info:pmid/22500634#body:33	2	1	Nerve {Organ urn:agi-ncimorgan:C1280541 }
PKM --+> vascularization	Regulation	An interesting hypothesis of the authors is that Tumor M2-PK released from tumors might stimulate angiogenesis by binding to TEM8., One possible mechanism to explain the tumor-promoting properties of PKM1 and PKM2 fibroblasts could be an increase in tumor angiogenesis., In addition to its effects on transcription of metabolic genes, PKM2 stimulates HIF-1- and HIF-2-mediated expression of the VEGFA gene (which encodes vascular endothelial growth factor), thereby promoting angiogenesis ( ) .	info:pmid/20156581#body:216, info:pmid/22236875#cont:182, info:pmid/22824010#body:81	2	3	
ATP5A1 ---| vascularization	Regulation	ATPM-Raf, acts in a dominant negative fashion to suppress angiogenesis.	info:doi/10.1016/j.acra.2007.04.003#body:19	2	1	
YWHAE ---> brain development	Regulation	Thus, 14-3-3 e has pivotal roles in neuronal migration and development of the brain., Recently, it was shown that 14-3-3e protein encoded by YWHAE gene is important in brain development and neuronal migration .	info:pmid/23078967#body:81, info:pmid/15196593#body:146	2	2	
CFL1 ---> brain development	Regulation	N-cofilin and ADF have distinct roles in brain development., By contrast, neuronal complexity, brain development, and synaptic function are severely impaired in	info:pmid/17875668#body:48, info:pmid/22046357#cont:321,	2	3	Heart Ventricle {Organ urn:agi-ncimorgan:C0018827 }

		n-cofilin mutants [6,17]., N-cofilin also controls cell cycle progression in neuronal progenitors in the ventricular zone during brain cortex development [ ].	info:pmid/19740640#body:42			
YWHAZ ---> synaptic transmission	Regulation	In Drosophila, 14-3-3? proteins are enriched at nerve terminals and involved in regulating synaptic transmissions at the neuromuscular junction ( )., Increased expression of 14-3-3z in Alzheimer's disease brain may, therefore, cause synaptic pathology by inhibiting neurite outgrowth, synapse formation, and synaptic transmission.	info:pmid/16982421#body:22, info:pmid/24367683#cont:451	2	2	Brain {Organ urn:agi-ncimorgan:C1269537 }
PGK1 --+> axon guidance	Regulation	Dosage sensitive genetic interactions indicate that MIG-10 functions with ABI-1 and WVE-1 to mediate axon guidance., Recent work has demonstrated that MIG-10 functions as an effector for Rac during axon guidance., Also, the C. elegans MRL protein Mig-10 uses its affinity to Ena/VASP to promote axon guidance and outgrowth [ ]., C. elegans MIG-10 plays roles in both Slit-dependent and netrin-dependent axon guidance pathways., MIG-10/lamellipodin and AGE-1/PI3K promote axon guidance and outgrowth in response to slit and netrin. <more data available...>	info:pmid/23209429#abs:8, info:pmid/18951796#body:69, info:pmid/19615876#body:61, info:pmid/16618541#body:91, info:pmid/24553288#cont:427, info:pmid/20417104#body:133, info:pmid/23628914#cont:347, info:pmid/22699910#cont:331, info:doi/10.1016/j.cub.2008.04.050#body:92	2	9	Nerve {Organ urn:agi-ncimorgan:C1280541 }, Colon {Organ urn:agi-ncimorgan:C1281569 }, Dendrites {Organ urn:agi-ncimorgan:C0011305 }
VEGFA --+> axon guidance	Regulation	Similar to Shh and Netrin-1, VEGF-mediated commissural axon guidance	info:pmid/21658588#abs:6,	2	11	Commissure {Organ urn:agi-

		requires the activity of Src family kinases., Vascular endothelial growth factor (VEGF) and fibroblast growth factor play important roles in the formation of the blood vascular system and in axon guidance, nervous system development and function., However, evidence has emerged that VEGF-A also promotes a wide range of neuronal functions, both in vitro and in vivo, including neurogenesis, neuronal migration, neuronal survival and axon guidance. <more data available...>	info:pmid/24623082#abs:1, info:pmid/22434866#abs:2, info:pmid/21658587#body:160, info:pmid/21835339#body:133, info:pmid/24267647#body:66, info:pmid/23076132#cont:31, info:pmid/12517344#body:155, info:doi/10.1016/j.neuron.2011.05.020#body:66, info:doi/10.1016/j.ceb.2012.02.002#body:80 <more data available...>				ncimorgan:C1185742 }, Vascular system {Organ urn:agi-ncimorgan:C0489903 }, Blood Vessels {Organ urn:agi-ncimorgan:C0005847 }, Cerebellum {Organ urn:agi-ncimorgan:C1268981 }
YWHAB --+> vascularization	Regulation	The 14-3-3ß protein is overexpressed in K2 cells compared with untransformed rat liver tissue. 14-3-3ß downregulation through transfection with an antisense 14-3-3ß expression vector inhibits cell growth, tumorigenicity, and angiogenesis of K2 cells.	info:pmid/24468084#body:8	2	1		liver parenchyma {Organ urn:agi-ncimorgan:C0736268 }
VEGFA ---> CFL1	Regulation	VEGF-A induced LIMK1 activation and cofilin phosphorylation, and this was inhibited by the p38 mitogen-activated protein kinase inhibitor SB203580., Inhibition of JNK1/2, c-Src, and phosphatidylinositol 3-kinase/Akt	info:pmid/16456544#abs:4, info:pmid/20463056#abs:4, info:pmid/20026108#body:202	2	3		Arteries {Organ urn:agi-ncimorgan:C0003842 }

		suppressed VEGF-induced stress fiber formation and cofilin-1 phosphorylation. c-Src inhibition suppressed VEGF-induced phosphorylation of focal adhesion kinase, paxillin, and focal adhesion., We also found that recombinant human ChM-I inhibits VEGF-A-induced Ser-3 phosphorylation of cofilin and possibly enhances the actin depolymerization in the Human umbilical vein endothelial cells and the MSS31 cells ( ).				
PGK1 ---| VEGFA	MolTransport	Overexpression of PGK1 reduced the secretion of vascular endothelial growth factor and interleukin-8 and increased the generation of angiostatin., Overexpression of phosphoglycerate kinase 1 reduced the secretion of vascular endothelial growth factor and decreased tumor angiogenesis.	info:pmid/17210694#abs:4, info:pmid/19299076#body:144	2	2	
VEGFA ---| MDK	Regulation	Among the 96 genes on the array, 25 genes were differentially expressed, of which 24 were upregulated and one (midkine) was downregulated in the VEGF/MT tumors.	info:pmid/15765121#body:180	2	1	Blood Vessels {Organ urn:agi-ncimorgan:C0005847 }
MDK ---> VEGFA	Regulation	We propose a model in which MDK is a new modulator of the VEGF-A-VEGFR-2 axis., Further studies on the transcriptional regulation of the VEGF gene mediated by midkine are needed; currently, in vitro studies are being conducted to investigate midkine-VEGF cross-talking during tumour angiogenesis.	info:pmid/18392135#abs:6, info:pmid/17110085#body:89	2	2	Microvessels {Organ urn:agi-ncimorgan:C2350570 }
HSPD1 ---| VEGFA	Expression	The DNA-hsp65 treatment blocked the expression of VEGF in mice tuberculous meningitis., vascular endothelial growth factor expression was downregulated by	info:pmid/23491717#abs:10, info:pmid/23135134#cont:185,	2	4	Brain {Organ urn:agi-ncimorgan:C1269537 }, Heart {Organ urn:agi-

		HSP65 vaccination., Knockdown of TLR2 significantly attenuated the HSP60-stimulated expressions of VEGF (P<0.01), IL-10 (P<0.01), and the phosphorylation of Stat3 (P<0.01) (Fig. S5B-D)., The mechanism of tumor vessel attenuation in this model may be as follows: Firstly, HSP65-X10-ßhCGCTP37 may attenuate angiogenesis through vascular endothelial growth factor.	info:pmid/19654875#body:187, info:pmid/19913113#body:207			ncimorgan:C1281570 }
YWHAB ---> VEGFA	Expression	The deregulated expression of 14-3-3ß largely participated in tumorigenic angiogenesis through the constitutive stimulation of VEGF expression., Reduced 14-3-3ß levels have been shown to inhibit VEGF production and angiogenesis and to increase apoptosis and suppress tumor size, while downregulation of 14-3-3ß by antisense 14-3-3ß RNA suppresses hepatoma cell growth both in vitro and in vivo .	info:pmid/20388496#body:44, info:pmid/24268498#body:87	2	2	
PKM --+> VEGFA	Expression	PKM2 binding enhances the expression of hypoxia-inducible factor 1 target genes ( ) including LDHA, PDK1, and VEGFA (encoding the vascular endothelial growth factor) ( )., In addition to its effects on transcription of metabolic genes, PKM2 stimulates HIF-1- and HIF-2-mediated expression of the VEGFA gene (which encodes vascular endothelial growth factor), thereby promoting angiogenesis ( ) ., Moreover, T454A mutant PKM2 was significantly less potent to activate endogenous HIF-1a target genes, such as	info:pmid/24508027#body:93, info:pmid/22824010#body:81, info:pmid/24142698#cont:237, info:pmid/21785006#cont:138, info:pmid/23880164#body:99, info:pmid/21620138#body:177	2	6	

		LDHA, PDK1, ENO1, VEGF, and GLUT1, in HEK293T, HepG2, or HeLa cells (Fig. 7, b–d). <more data available...>				
HNRNPK --+> VEGFA	Expression	This study shows that hnRNP K augments efficiency of VEGF mRNA translation stimulated by ANG II., PKCdelta-mediated phosphorylation of hnRNP K is required for Ang II stimulation of VEGF mRNA translation., angiotensin II stimulation of vascular endothelial growth factor mRNA translation partly depended on increased binding of hnRNPK to the 3' end of vascular endothelial growth factor mRNA (111)., Recently, it was proposed that hnRNP K activates the VEGF-A promoter by binding to unwound superhelical single stranded C-rich sequences upstream of the transcription start site and to support association of transcription initiation factors [27]. hnRNP K is composed of modular regions that confer binding both to RNA or DNA as well as protein-protein interaction domains [28]. <more data available...>	info:pmid/17581920#abs:10, info:pmid/18295448#abs:10, info:pmid/16959824#body:323, info:pmid/22879910#cont:39, info:doi/10.1016/j.cellsig.2008.01.016#body:106	2	5	cortex of kidney {Organ urn:agi-ncimorgan:C0022655 }
PEBP1 ---> synaptic transmission	Regulation	Likewise, isoaspartate-rich and potentially dysfunctional a-synuclein, ß-synuclein, UCHL1, PEBP, clathrin light chains a and b, calreticulin, calmodulin, and synapsin 1 (a protein whose functions include tethering synaptic vesicles to the cytoskeleton (15, 21, 63)), could drive the aberrant synaptic transmission recorded in these mice (13, 14, 16, 17).	info:pmid/16923807#body:295	2	1	Brain {Organ urn:agi-ncimorgan:C1269537 }
VEGFA --->	Regulation	In addition to guidance processes, VEGF	info:doi/10.1016/j.	2	1	

hippocampus morphology		directly controls survival of subsets of neuronal populations [ ] and modulates neuronal plasticity and memory functions [ ], demonstrating that VEGF affects cognition processes by hippocampal circuits.	ceb.2012.02.002#body:83			
YWHAE ---> neurogenesis	Regulation	14-3-3epsilon regulates a wide range of biological processes, including cell cycle control, proliferation, and apoptosis, and plays a significant role in neurogenesis and the formation of malignant tumours.	info:pmid/20565895#abs:1	2	1	
ENO1 ---> vascularization	Regulation	Recently, it has been shown that MBP-1 overexpression results in the modulation of MMP-2 expression, the inhibition of in vitro angiogenesis and the regression of primary and metastatic breast tumor growth in an immunocompetent mouse model, [29]., The critical proteins in the enolase-1-mediated active proliferation of endothelial cells and the active transformation of angiogenesis of breast cancer cells responding to hypoxia were identified using cellular and molecular biological and proteomics techniques., Although pharmacological induction of VEGF and ENO1 gene expression may provide therapeutic benefits under ischemic conditions, by stimulating angiogenesis and glycolysis, respectively, agents that also induce erythropoietin expression and erythropoiesis would not be useful because of the risk of vascular accidents associated with polycythemia (Sokol et al., 1995).	info:pmid/20886042#body:332, info:pmid/23381546#cont:49, info:pmid/9804609#body:183	2	3	Blood Vessels {Organ urn:agi-ncimorgan:C0005847 }

MDK ---> brain development	Regulation	From in vitro cell biology studies and the embryonic expression pattern of midkine in the central nervous system it was concluded that midkine directs neurite interconnections during an early phase of brain development and may later on have a maintenance function in some restricted areas.	info:pmid/12122009#body:49	2	1	Central Nervous System {Organ urn:agi-ncimorgan:C0927232 }
VEGFA --+> YWHAZ	Regulation	E, VEGF decreases binding of exogenous HDAC7 to ß-catenin but increases its binding to 14-3-3 ? proteins., We found that FLNA, vimentin, CRYAB and YWHAZ are constantly over-expressed in myeloma ECs and enhanced by VEGF, FGF2, HGF and myeloma plasma cell conditioned medium.	info:pmid/20224040#body:200, info:pmid/21963844#cont:133	2	2	
VEGFA --+> ENO1	Regulation	In response to hypoxia, mammalian cells express multiple gene products [including erythropoietin and vascular endothelial growth factor (VEGF)] that serve to increase O2 delivery, as well as glucose transporters and glycolytic enzymes (such as enolase 1) that allow metabolic adaptation to decreased O2 availability.	info:pmid/9804609#abs:1	2	1	
CFL1 ---> axon guidance	Regulation	Furthermore, COFILIN is involved in the regulation of the actin cytoskeleton and in axon guidance., The effect of Limk1/cofilin on axon growth has been clearly demonstrated in vitro, but it remains unclear whether Limk1/ cofilin acts as an intracellular effector of axon guidance in vivo.	info:pmid/21563072#cont:330, info:pmid/21084599#cont:42	2	2	Commissure {Organ urn:agi-ncimorgan:C1185742 }
PKM ---> neurogenesis	Regulation	Thus, impairment of pyruvate kinase M2 and other neural progenitor cell proteins via nitration may contribute to impairment	info:pmid/21708025#cont:233	2	1	Hippocampus {Organ urn:agi-ncimorgan:C0019564

		of hippocampal neurogenesis in the setting of METH abuse.				}
PRDX1 ---> vascularization	Regulation	These results suggest a mechanism by which peroxiredoxin 1 regulates angiogenesis in CaP and suggests a scenario of continued stimulation of angiogenesis and inflammation in CaP.	info:pmid/23185615#cont:334	2	1	
PRDX1 --+> VEGFA	Expression	The mechanism by which Peroxiredoxin 1 regulates VEGF expression in normoxic conditions was investigated in the current study.	info:pmid/23185615#abs:5	2	1	
EMX1 ---| hippocampus morphology	Regulation	Because the hippocampal morphology was severely disrupted in Emx1-Dicer cko brains by embryonic day 18.5, mostly due to reduced	info:pmid/21991391#cont:128	2	1	Brain {Organ urn:agi-ncimorgan:C1269537 }
PRDX2 --+> vascularization	Regulation	Furthermore, PrxII deficiency suppresses tumor angiogenesis in vivo.	info:pmid/22099303#abs:7	2	1	
PEBP1 ---> brain development	Regulation	The HCNP precursor protein , composed of 186 amino acids, is an inhibitory factor of the c-Raf/MEK cascade and may be involved in fetal rat brain development via the inhibition of phosphorylation of Erk.	info:pmid/20682295#abs:2	2	1	medial septal nucleus {Organ urn:agi-ncimorgan:C0175233 }

Table 7c: Global Analysis

Reference Table:

Relation	Type	Sentence	TextRef	Organ
VEGFA --+> vascularization	Regulation	VEGF165 induces marked vascular growth.	info:pmid/1384392#abs:4	
VEGFA --+> vascularization	Regulation	VEGF is a major inducer of angiogenesis.	info:pmid/19800880#abs:1	
VEGFA --+> vascularization	Regulation	VEGF-A promotes angiogenesis in many tissues.	info:pmid/16453023#abs:1	
VEGFA --+> vascularization	Regulation	VEGF is an important factor for angiogenesis.	info:pmid/14717227#abs:1	
VEGFA --+> vascularization	Regulation	VEGF plays a central role in tumor angiogenesis.	info:pmid/17615535#abs:4	
VEGFA --+> vascularization	Regulation	CRHR-2 inhibits VEGF-induced neovascularization.	info:pmid/18484196#abs:3	
VEGFA --+> vascularization	Regulation	VEGF plays a principal role in tumor angiogenesis.	info:pmid/15064856#abs:3	
VEGFA --+> vascularization	Regulation	VEGF was an important factor in tumor angiogenesis.	info:pmid/12500403#abs:11	
VEGFA --+> vascularization	Regulation	VEGF plays a central role in angiogenesis in cancer.	info:pmid/22992725#abs:1	
VEGFA --+> vascularization	Regulation	It inhibits VEGF-induced angiogenesis in preclinical models.	info:pmid/22264850#abs:2	
VEGFA --+> vascularization	Regulation	VEGF is crucial for metaphyseal bone vascularization.	info:pmid/14722611#abs:1	
VEGFA --+> vascularization	Regulation	VEGF and HIF-1 are potent inducers of angiogenesis.	info:pmid/18288940#abs:7	
VEGFA --+> vascularization	Regulation	VEGF is an important regulator of tumor angiogenesis.	info:pmid/12687277#abs:2	
VEGFA --+>	Regulation	VEGF-A is the essential factor in tumor angiogenesis.	info:pmid/18282597#abs:2	

vascularization				
VEGFA --+> vascularization	Regulation	VEGF is the primary stimulator of angiogenesis.	info:pmid/17193594#abs:10	
VEGFA --+> vascularization	Regulation	In these responses, VEGF regulates angiogenesis.	info:pmid/16832062#abs:2	Blood Vessels {Organ urn:agi-ncimorgan:C0005847}
VEGFA --+> vascularization	Regulation	Vascular endothelial growth factor is a mediator of angiogenesis.	info:pmid/12232762#abs:2	
VEGFA --+> vascularization	Regulation	In this study, we test their effects on VEGF-induced angiogenesis.	info:pmid/12135741#abs:5	
VEGFA --+> vascularization	Regulation	HIF-1alpha and VEGF are well known inducers of angiogenesis.	info:pmid/15947208#abs:10	
VEGFA --+> vascularization	Regulation	VEGF induces in vivo angiogenesis and vascular permeability.	info:pmid/9053843#abs:2	
VEGFA --+> vascularization	Regulation	VEGF is a crucial inducer of angiogenesis both in vivo and in vitro.	info:pmid/15474452#abs:2	
VEGFA --+> vascularization	Regulation	VEGF is probably important in the angiogenesis of melanomas.	info:pmid/9626350#abs:12	
VEGFA --+> vascularization	Regulation	Vascular endothelial growth factor (VEGF) is involved in angiogenesis.	info:pmid/15736481#abs:1	
VEGFA --+> vascularization	Regulation	VEGF plays an important role in tumor angiogenesis and growth.	info:pmid/15746177#abs:4	
VEGFA --+> vascularization	Regulation	Vascular endothelial growth factor (VEGF) is crucial for angiogenesis.	info:pmid/19774949#abs:2	
VEGFA --+> vascularization	Regulation	VEGF is the best characterized mediator of tumor angiogenesis.	info:pmid/18000042#abs:1	
VEGFA --+> vascularization	Regulation	Angiogenesis of PC12 xenografts is mediated by VEGF.	info:pmid/12490855#abs:12	
VEGFA --+> vascularization	Regulation	Inhibition of VEGF impairs angiogenesis and disrupts metastatic spread.	info:pmid/20939812#abs:10	
VEGFA --+> vascularization	Regulation	Vascular endothelial growth factor is the main mediator of angiogenesis.	info:pmid/16967703#abs:1	
VEGFA --+> vascularization	Regulation	In vivo, VEGF-induced angiogenesis was impaired by myristoylated peptide.	info:pmid/12067896#abs:7	

VEGFA --+> vascularization	Regulation	Vascular endothelial growth factor is a potent stimulator of angiogenesis.	info:pmid/22371751#abs:1	
VEGFA --+> vascularization	Regulation	VEGF is fundamental in the development and maintenance of the vasculature.	info:pmid/16361360#abs:1	
VEGFA --+> vascularization	Regulation	Vascular endothelial growth factor (VEGF) stimulates angiogenesis in vivo.	info:pmid/12752628#abs:2	
VEGFA --+> vascularization	Regulation	VEGF plays a pivotal role in tumor angiogenesis and tumorigenesis.	info:pmid/24337450#abs:3	
VEGFA --+> vascularization	Regulation	VEGF is a key mediator of both physiologic and tumor angiogenesis.	info:pmid/15289855#abs:2	
VEGFA --+> vascularization	Regulation	VEGF and BMP play important roles in angiogenesis and osteogenesis.	info:pmid/20817238#abs:1	
VEGFA --+> vascularization	Regulation	Vascular endothelial growth factor plays an important role in angiogenesis.	info:pmid/24023685#abs:2	
VEGFA --+> vascularization	Regulation	VEGF-A is the most potent angiogenic factor in tumour angiogenesis.	info:pmid/24416596#abs:1	
VEGFA --+> vascularization	Regulation	KSHV vGPCR is constitutively active and induces VEGF-mediated angiogenesis.	info:pmid/11504542#abs:2	
VEGFA --+> vascularization	Regulation	Vascular endothelial growth factor (VEGF) is a key mediator of angiogenesis.	info:pmid/22877947#abs:1	
VEGFA --+> vascularization	Regulation	Vascular endothelial growth factor (VEGF) is a potent angiogenesis mediator.	info:pmid/18158186#abs:1	
VEGFA --+> vascularization	Regulation	Angiogenesis is largely driven by vascular endothelial growth factor (VEGF).	info:pmid/19219548#abs:1	
VEGFA --+> vascularization	Regulation	FGF-2 and VEGF are potent angiogenesis inducers in vivo and in vitro.	info:pmid/9647657#abs:1	
VEGFA --+> vascularization	Regulation	Vascular-endothelial-growth-factor (VEGF) is a key mediator of angiogenesis.	info:pmid/22786517#abs:1	
VEGFA --+> vascularization	Regulation	Vascular endothelial growth factor (VEGF) is a key mediator in angiogenesis.	info:pmid/16292207#abs:4	
VEGFA --+> vascularization	Regulation	Vascular endothelial growth factor (VEGF) is a major inducer of angiogenesis.	info:pmid/18092518#abs:1	
VEGFA --+> vascularization	Regulation	Vascular endothelial growth factor (VEGF) is a major inducer of angiogenesis.	info:pmid/11074523#abs:2	

VEGFA --+> vascularization	Regulation	Vascular endothelial growth factor (VEGF) is a key regulator of angiogenesis.	info:pmid/18392139#abs:2	
VEGFA --+> vascularization	Regulation	Vascular endothelial growth factor (VEGF) is a key regulator of angiogenesis.	info:pmid/15515117#abs:1	
VEGFA --+> vascularization	Regulation	Vascular endothelial growth factor (VEGF) is a key regulator of angiogenesis.	info:pmid/23788940#abs:2	
VEGFA --+> vascularization	Regulation	Vascular endothelial growth factor (VEGF) is a key regulator in angiogenesis.	info:pmid/12738717#abs:1	
VEGFA --+> vascularization	Regulation	Vascular endothelial growth factor (VEGF) is a key regulator of angiogenesis.	info:pmid/23132736#abs:2	
VEGFA --+> vascularization	Regulation	Vascular endothelial growth factor (VEGF) is a key regulator of angiogenesis.	info:pmid/15885310#abs:1	
VEGFA --+> vascularization	Regulation	VEGF has an essential role in angiogenesis and vascular permeability.	info:pmid/21424068#abs:3	
VEGFA --+> vascularization	Regulation	Vascular endothelial growth factor (VEGF) is a key regulator of angiogenesis.	info:pmid/12825195#abs:1	
VEGFA --+> vascularization	Regulation	Vascular endothelial cell growth factor (VEGF) is essential for angiogenesis.	info:pmid/11250939#abs:1	
VEGFA --+> vascularization	Regulation	Vascular endothelial growth factor (VEGF) is a key regulator of angiogenesis.	info:pmid/21996035#abs:2	
VEGFA --+> vascularization	Regulation	Vascular endothelial growth factor (VEGF) is a key modulator of angiogenesis.	info:pmid/21659383#abs:1	
VEGFA --+> vascularization	Regulation	Vascular endothelial growth factor (VEGF) is a key regulator of angiogenesis.	info:pmid/16954424#abs:3	
VEGFA --+> vascularization	Regulation	Vascular endothelial growth factor (VEGF) is a key regulator of angiogenesis.	info:pmid/10668221#abs:1	
VEGFA --+> vascularization	Regulation	Vascular endothelial growth factor (VEGF) is a key modulator of angiogenesis.	info:pmid/19774210#abs:2	
VEGFA --+> vascularization	Regulation	VEGF165 specifically induces angiogenesis in doses of 0.5 microgram and more.	info:pmid/7694800#abs:7	
VEGFA --+> vascularization	Regulation	Vascular endothelial growth factor (VEGF) is a potent inducer of angiogenesis.	info:pmid/20034820#abs:2	
VEGFA --+> vascularization	Regulation	During hypoxia, VEGF promotes angiogenesis in the testis.	info:pmid/17374694#abs:2	

VEGFA --+> vascularization	Regulation	VEGF plays an important role in angiogenesis and hepatic regeneration.	info:pmid/17431590#abs:3	
VEGFA --+> vascularization	Regulation	Vascular Endothelial Growth Factor (VEGF) is a major regulator of angiogenesis.	info:pmid/21980393#abs:1	
VEGFA --+> vascularization	Regulation	Vascular endothelial growth factor (VEGF) is a potent mediator of angiogenesis.	info:pmid/19729876#abs:2	
VEGFA --+> vascularization	Regulation	Vascular endothelial growth factor (VEGF) is a potent mediator of angiogenesis.	info:pmid/18330755#abs:1	
VEGFA --+> vascularization	Regulation	Vascular endothelial growth factor (VEGF) plays a crucial role in angiogenesis.	info:pmid/12653851#abs:2	
VEGFA --+> vascularization	Regulation	Vascular endothelial growth factor (VEGF) plays a central role in angiogenesis.	info:pmid/22238513#abs:1	
VEGFA --+> vascularization	Regulation	Vascular endothelial growth factor (VEGF) is a major regulator of angiogenesis.	info:pmid/9918864#abs:1	
VEGFA --+> vascularization	Regulation	Vascular endothelial growth factor (VEGF) is a major modulator of angiogenesis.	info:pmid/15352169#abs:1	
VEGFA --+> vascularization	Regulation	Vascular endothelial growth factor (VEGF) plays a pivotal role in angiogenesis.	info:pmid/18759208#abs:1	
VEGFA --+> vascularization	Regulation	Vascular endothelial growth factor (VEGF) plays a crucial role in angiogenesis.	info:pmid/20837456#abs:2	
VEGFA --+> vascularization	Regulation	Vascular endothelial growth factor (VEGF) plays a pivotal role in angiogenesis.	info:pmid/20621071#abs:1	
VEGFA --+> vascularization	Regulation	Vascular endothelial growth factor (VEGF) is a potent mediator of angiogenesis.	info:pmid/17092923#abs:1	
VEGFA --+> vascularization	Regulation	VEGF is a key regulator of tumor angiogenesis.	info:pmid/14972022#abs:9	
VEGFA --+> vascularization	Regulation	Here, we report that TRPC6 is important for VEGF-mediated angiogenesis.	info:pmid/19394138#abs:3	
VEGFA --+> vascularization	Regulation	Ingested holo-hLF did not affect VEGF-A-mediated angiogenesis.	info:pmid/15192265#abs:4	
VEGFA --+> vascularization	Regulation	Vascular endothelial growth factor (VEGF) plays a critical role in angiogenesis.	info:pmid/20707740#abs:1	
VEGFA --+> vascularization	Regulation	The vascular endothelial growth factor (VEGF) plays a major role in angiogenesis.	info:pmid/23586368#abs:1	

VEGFA --+> vascularization	Regulation	The vascular endothelial growth factor (VEGF) has a pivotal role in angiogenesis.	info:pmid/22699782#abs:1	
VEGFA --+> vascularization	Regulation	Vascular endothelial growth factor (VEGF) is a crucial regulator of angiogenesis.	info:pmid/24183611#abs:1	
VEGFA --+> vascularization	Regulation	VEGF stimulates angiogenesis and acts as an autocrine growth factor.	info:pmid/16508632#abs:2	
VEGFA --+> vascularization	Regulation	Vascular endothelial growth factor A (VEGF-A) is a key regulator of angiogenesis.	info:pmid/20477757#abs:1	
VEGFA --+> vascularization	Regulation	Vascular endothelial growth factor (VEGF) is a potent stimulator of angiogenesis.	info:pmid/17875714#abs:1	
VEGFA --+> vascularization	Regulation	Vascular endothelial growth factor (VEGF) and its receptors promote angiogenesis.	info:pmid/17823371#abs:2	
VEGFA --+> vascularization	Regulation	Vascular endothelial growth factor (VEGF) is a critical regulator of angiogenesis.	info:pmid/17196035#abs:2	
VEGFA --+> vascularization	Regulation	Vascular endothelial growth factor (VEGF) is a critical regulator of angiogenesis.	info:pmid/22965162#abs:1	
VEGFA --+> vascularization	Regulation	Whereas VEGF(165) stimulates angiogenesis, VEGF(165)b is anti-angiogenic.	info:pmid/16423853#abs:3	
VEGFA --+> vascularization	Regulation	Vascular endothelial growth factor (VEGF) plays an important role in angiogenesis.	info:pmid/11908718#abs:1	
VEGFA --+> vascularization	Regulation	Vascular endothelial growth factor-A (VEGFA) is the main mediator of angiogenesis.	info:pmid/23383089#abs:1	
VEGFA --+> vascularization	Regulation	Vascular endothelial growth factor A (VEGF-A) is a potent inducer of angiogenesis.	info:pmid/17363377#abs:1	
VEGFA --+> vascularization	Regulation	Vascular endothelial growth factor (VEGF) plays an important role in angiogenesis.	info:pmid/17625371#abs:1	
VEGFA --+> vascularization	Regulation	VEGF plays a pivotal role in the neovascularization of the choroid in AMD.	info:pmid/16935207#abs:1	
VEGFA --+> vascularization	Regulation	Vascular endothelial growth factor (VEGF) is a potent stimulator for angiogenesis.	info:pmid/17951537#abs:1	
VEGFA --+> vascularization	Regulation	Vascular endothelial growth factor (VEGF) plays an important role in angiogenesis.	info:pmid/12429176#abs:1	
VEGFA --+> vascularization	Regulation	Vascular endothelial growth factor (VEGF) is a fundamental factor for angiogenesis.	info:pmid/19603178#abs:1	

VEGFA --+> vascularization	Regulation	Vascular endothelial growth factor (VEGF) is an important regulator of angiogenesis.	info:pmid/18317954#abs:3	
VEGFA --+> vascularization	Regulation	Vascular endothelial growth factor (VEGF) is an important regulator of angiogenesis.	info:pmid/23390328#abs:3	
VEGFA --+> vascularization	Regulation	Much evidence indicates that VEGF is a key mediator of angiogenesis.	info:pmid/15153419#abs:3	
VEGFA --+> vascularization	Regulation	Vascular endothelial cell growth factor (VEGF) is a major regulator of angiogenesis.	info:pmid/10339488#abs:1	
VEGFA --+> vascularization	Regulation	Vascular endothelial growth factor (VEGF) is an important regulator of angiogenesis.	info:pmid/11146397#abs:1	
VEGFA --+> vascularization	Regulation	Vascular endothelial growth factor (VEGF) is a principal stimulator of angiogenesis.	info:pmid/15263820#abs:1	
VEGFA --+> vascularization	Regulation	Vascular endothelial growth factor (VEGF) is an important regulator of angiogenesis.	info:pmid/10213909#abs:1	
VEGFA --+> vascularization	Regulation	Potential prodrugs of inhibitors of VEGF-induced angiogenesis have been investigated.	info:pmid/17254788#abs:1	
VEGFA --+> vascularization	Regulation	VEGF(165)b inhibits angiogenesis and is downregulated in tumours.	info:pmid/18657413#abs:3	
VEGFA --+> vascularization	Regulation	Vascular endothelial growth factor (VEGF) is one of the major angiogenesis regulators.	info:pmid/9790910#abs:1	
VEGFA --+> vascularization	Regulation	Importantly, the inclusion of VEGF enhanced vascularization.	info:pmid/19914711#abs:3	
VEGFA --+> vascularization	Regulation	The vascular endothelial growth factor (VEGF) plays an important role in angiogenesis.	info:pmid/11396794#abs:1	
VEGFA --+> vascularization	Regulation	The VEGF-induced pattern of neovascularization was also investigated.	info:pmid/20495752#abs:4	Cornea
VEGFA --+> vascularization	Regulation	VEGF is critical also for reproductive and bone angiogenesis.	info:pmid/15294883#abs:5	
VEGFA --+> vascularization	Regulation	Inhibition of VEGF activities is able to reduce angiogenesis and tumour growth.	info:pmid/12838326#abs:2	
VEGFA --+> vascularization	Regulation	Vascular endothelial growth factor (VEGF) is an essential regulator of vascularization.	info:pmid/10982845#abs:1	
VEGFA --+> vascularization	Regulation	The vascular endothelial growth factor (VEGF) is an important regulator of angiogenesis.	info:pmid/20187681#abs:2	

VEGFA --+> vascularization	Regulation	The vascular endothelial growth factor (VEGF) is an important regulator of angiogenesis.	info:pmid/21117958#abs:2	
VEGFA --+> vascularization	Regulation	Vascular endothelial growth factor (VEGF) plays key roles in tumor angiogenesis.	info:pmid/16309186#abs:1	
VEGFA --+> vascularization	Regulation	This suggests that VEGF is involved in subretinal angiogenesis.	info:pmid/8703891#abs:11	Eye
VEGFA --+> vascularization	Regulation	Vascular endothelial growth factor (VEGF) is known to play a major role in angiogenesis.	info:pmid/19266483#abs:1	
VEGFA --+> vascularization	Regulation	The association with E-selectin in VEGF-induced angiogenesis was also evaluated.	info:pmid/11399949#abs:2	
VEGFA --+> vascularization	Regulation	Vascular endothelial growth factor (VEGF) plays a key role in tumor angiogenesis.	info:pmid/10370645#abs:1	
VEGFA --+> vascularization	Regulation	Aims: Neovascularisation is mainly mediated by vascular endothelial growth factor (VEGF).	info:pmid/19736088#abs:1	
VEGFA --+> neurogenesis	Regulation	Vascular endothelial growth factor (VEGF) regulates neurogenesis.	info:pmid/16269210#abs:1	
VEGFA --+> neurogenesis	Regulation	In addition, recent studies indicate that VEGF enhances neurogenesis after ischemia.	info:pmid/21469168#abs:7	
VEGFA --+> neurogenesis	Regulation	Vascular endothelial growth factor (VEGF) enhances neurogenesis in ischemic brains.	info:pmid/19834383#abs:1	Brain {Organ urn:agi-ncimorgan:C1269537}
VEGFA --+> neurogenesis	Regulation	Furthermore, inhibition of VEGF expression by RNA interference completely blocked the environmental induction of neurogenesis.	info:pmid/15258583#abs:7	Hippocampus {Organ urn:agi-ncimorgan:C0019564}
VEGFA --+> neurogenesis	Regulation	Furthermore, inhibition of VEGF expression by RNA interference completely blocked the environmental induction of neurogenesis.	info:pmid/16472200#abs:8	Hippocampus {Organ urn:agi-ncimorgan:C0019564}
VEGFA --+> neurogenesis	Regulation	Recently, VEGF has also been proposed to play a role in neural development, neuroprotection, and adult neurogenesis.	info:pmid/14983237#abs:2	Vascular system {Organ urn:agi-ncimorgan:C0489903}
VEGFA --+> neurogenesis	Regulation	Importantly, this was done in settings that allowed the uncoupling of VEGF-promoted angiogenesis, neurogenesis, and memory.	info:pmid/21385942#abs:4	Hippocampus {Organ urn:agi-ncimorgan:C0019564}
VEGFA --+> neurogenesis	Regulation	Several recent studies demonstrate that VEGF also promotes neurogenesis, neuronal patterning,	info:pmid/20885857#abs:2	

		neuroprotection and glial growth.		
VEGFA --+> neurogenesis	Regulation	Vascular endothelial growth factor (VEGF) promotes the proliferation of vascular endothelial cells and the neurogenesis of neural stem cells.	info:pmid/17618600#abs:2	
VEGFA --+> neurogenesis	Regulation	The angiogenic factor vascular endothelial growth factor A (VEGF) has been shown to have a role in neurogenesis, but how it affects adult neurogenesis is not fully understood.	info:pmid/20040492#abs:1	
VEGFA --+> neurogenesis	Regulation	Adult hippocampal neurogenesis is not only affected by external stimuli but also regulated by internal growth factors including BDNF, VEGF and IGF-1.	info:pmid/19470236#abs:4	
VEGFA --+> neurogenesis	Regulation	Collectively, these findings suggest that MSCs secrete bioactive factors, including HGF and VEGF, that stimulate neurogenesis and improve outcomes of TBI in a rat model.	info:pmid/22876972#abs:8	Brain {Organ urn:agi-ncimorgan:C1269537}
VEGFA --+> neurogenesis	Regulation	Conversely, prototypic angiogenic factors such as VEGF control neurogenesis and regulate axon and neuron guidance, independently of their angiogenic activity.	info:pmid/20120252#abs:7	Nerve {Organ urn:agi-ncimorgan:C1280541}
VEGFA --+> neurogenesis	Regulation	In conclusion, VEGF significantly augments neurogenesis and angiogenesis and reduces lesion volumes after traumatic brain injury.	info:pmid/20068579#abs:8	
VEGFA --+> neurogenesis	Regulation	Recent evidence indicates that VEGF facilitates memory and learning through stimulating angiogenesis and neurogenesis in the rat hippocampal dendate gyrus.	info:pmid/16569480#abs:2	
VEGFA --+> neurogenesis	Regulation	Vascular endothelial growth factors and their high-affinity tyrosine kinase Vascular endothelial growth factor receptors are key regulators of both angiogenesis and neurogenesis.	info:pmid/23475064#abs:1	
VEGFA --+> neurogenesis	Regulation	We investigated the molecular mechanisms through which VEGF stimulates neurogenesis in primary cultures of rat cerebral cortical neurons.	info:pmid/16329123#abs:3	Cerebrum {Organ urn:agi-ncimorgan:C1280654}
VEGFA --+>	Regulation	Our data suggest that Erythropoietin-increased VEGF	info:pmid/15178821#abs:1	subventricular zone

neurogenesis		and BDNF may be involved in angiogenesis and neurogenesis, which could contribute to functional recovery.	1	{Organ urn:agi-ncimorgan:C0521406}
VEGFA --+> neurogenesis	Regulation	Vascular endothelial growth factor is upregulated after a hypoxic insult and is involved in neuronal survival, angiogenesis, and neurogenesis during the recovery process.	info:pmid/20101028#abs:2	
VEGFA --+> neurogenesis	Regulation	These findings provide evidence that VEGF increases subventricular zone neurogenesis and neuromigration, consistent with a possible role in repair.	info:pmid/17243175#abs:8	Brain {Organ urn:agi-ncimorgan:C1269537}
VEGFA --+> neurogenesis	Regulation	Recent studies suggest that vascular endothelial growth factor (VEGF), a cytokine involved in angiogenesis and neurogenesis, may also be dysregulated during stress and depression.	info:pmid/19062198#abs:2	Endocrine system {Organ urn:agi-ncimorgan:C1280975}
VEGFA --+> neurogenesis	Regulation	Brain-derived neurotrophic factor (BDNF), a major neurotrophin and vascular endothelial growth factor (VEGF) have a documented role in neurogenesis, angiogenesis, and neuronal survival.	info:pmid/23929745#abs:1	
VEGFA --+> neurogenesis	Regulation	VEGF confers neuroprotection and promotes neurogenesis and cerebral angiogenesis, but the manner in which these effects may interact in the ischemic brain is poorly understood.	info:pmid/12813020#abs:2	Brain {Organ urn:agi-ncimorgan:C1269537}
VEGFA --+> neurogenesis	Regulation	The vascular endothelial growth factor (VEGF) signaling, which modulates angiogenesis and neurogenesis within the neurovascular unit, might play an important role in the neuro-endocrine-immune stress-adaptation system.	info:pmid/23871390#abs:1	Endocrine system {Organ urn:agi-ncimorgan:C1280975}
VEGFA --+> neurogenesis	Regulation	Vascular endothelial growth factor (VEGF) promotes neurogenesis in the adult hippocampus, but the way in which this process occurs in the Alzheimer's disease brain is still unknown.	info:pmid/22232015#abs:1	Hippocampus {Organ urn:agi-ncimorgan:C0019564}
VEGFA --+> neurogenesis	Regulation	To assess the importance of androgen-regulated, VEGF-induced MMP2 to adult angiogenesis and	info:pmid/18171938#abs:9	Prosencephalon {Organ urn:agi-

		neurogenesis, we treated testosterone-implanted females with the gelatinase inhibitor SB-3CT.		ncimorgan:C0085140}
VEGFA --+> neurogenesis	Regulation	However, evidence has emerged that VEGF-A also promotes a wide range of neuronal functions, both in vitro and in vivo, including neurogenesis, neuronal migration, neuronal survival and axon guidance.	info:pmid/22434866#abs:2	
VEGFA --+> neurogenesis	Regulation	Moreover, VEGF modulates neurogenesis in constitutive neurogenic and non-neurogenic regions of adult mammalian brains, which is a promising therapeutic strategy for neuronal replacement in traumatic brain injury and neurodegenerative diseases.	info:pmid/17882975#abs:3	Brain {Organ urn:agi-ncimorgan:C1269537}
VEGFA --+> neurogenesis	Regulation	Vascular endothelial growth factors have been shown to participate in atherosclerosis, arteriogenesis, cerebral edema, neuroprotection, neurogenesis, angiogenesis, postischemic brain and vessel repair, and the effects of transplanted stem cells in experimental stroke.	info:pmid/23475070#abs:1	
VEGFA --+> neurogenesis	Regulation	Angiogenesis factors, especially vascular endothelial growth factor, are now known to have roles in the birth of new neurons (neurogenesis), the prevention or mitigation of neuronal injury (neuroprotection), and the pathogenesis of stroke, Alzheimer's disease and motor neuron disease.	info:pmid/16355213#abs:3	
VEGFA --+> neurogenesis	Regulation	Functionally, members of the vascular endothelial growth factor (VEGF) family can stimulate neurogenesis as well as angiogenesis, but it has been unclear whether they act directly via VEGF receptors expressed by neural cells, or indirectly via the release of growth factors from angiogenic capillaries.	info:pmid/21498572#abs:2	Blood capillaries {Organ urn:agi-ncimorgan:C0006901}
VEGFA --+> neurogenesis	Regulation	VEGF is necessary for exercise-induced adult hippocampal neurogenesis.	info:pmid/14656329#title:1	
VEGFA --+> neurogenesis	Regulation	Therefore, VEGF may play an important role in adult neurogenesis.	info:pmid/16753262#body:112	dentate gyrus {Organ urn:agi-

				ncimorgan:C0152314}
VEGFA --+> neurogenesis	Regulation	Recent studies indicate that VEGF can stimulate neurogenesis.	info:pmid/19745179#body:166	Blood Vessels {Organ urn:agi-ncimorgan:C0005847}
VEGFA --+> neurogenesis	Regulation	Vascular endothelial growth factor (VEGF) stimulates neurogenesis in vitro and in vivo.	info:pmid/12181492#title:1	
VEGFA --+> neurogenesis	Regulation	Vascular endothelial growth factor (VEGF) stimulates neurogenesis in vitro and in vivo.	info:pmid/19553916#body:352	
VEGFA --+> neurogenesis	Regulation	In addition to its angiogenic role, VEGF also stimulates neurogenesis .	info:pmid/19150488#body:143	Cerebral cortex {Organ urn:agi-ncimorgan:C0007776}
VEGFA --+> neurogenesis	Regulation	Through its receptors, VEGF is also involved in neurogenesis ( ).	info:pmid/23603139#body:176	Microvessels {Organ urn:agi-ncimorgan:C2350570}
VEGFA --+> neurogenesis	Regulation	First, VEGF produced by transplanted cells may promote neurogenesis.	info:pmid/21226624#cont:239	subventricular zone {Organ urn:agi-ncimorgan:C0521406}
VEGFA --+> neurogenesis	Regulation	Furthermore, VEGF promotes post-ischemic neurogenesis and neuromigration ( ).	info:pmid/18822285#body:9	Brain {Organ urn:agi-ncimorgan:C1269537}
VEGFA --+> neurogenesis	Regulation	VEGF is necessary for exercise-induced adult hippocampal neurogenesis.	info:pmid/16034445#body:295	
VEGFA --+> neurogenesis	Regulation	VEGF is necessary for exercise-induced adult hippocampal neurogenesis.	info:pmid/17211450#body:216	
VEGFA --+> neurogenesis	Regulation	Vascular endothelial growth factor stimulates neurogenesis in vitro and in vivo.	info:pmid/23432711#cont:326	subventricular zone {Organ urn:agi-ncimorgan:C0521406}
VEGFA --+> neurogenesis	Regulation	What is the relationship between VEGFA- and VEGFB-induced neurogenesis?	info:pmid/16337622#body:73	Brain {Organ urn:agi-ncimorgan:C1269537}
VEGFA --+> neurogenesis	Regulation	Vascular endothelial growth factor (VEGF) stimulates neurogenesis in vitro and in vivo.	info:pmid/21470108#cont:642	
VEGFA --+> neurogenesis	Regulation	Vascular endothelial growth factor (VEGF) stimulates neurogenesis in vitro and in vivo.	info:pmid/19571795#body:380	Hippocampus {Organ urn:agi-ncimorgan:C0019564}
VEGFA --+>	Regulation	For example, the angiogenic factor VEGF is also	info:pmid/17357084#body:	Brain {Organ urn:agi-

neurogenesis		involved in the stimulation of neurogenesis.	110	ncimorgan:C1269537}
VEGFA --+> neurogenesis	Regulation	such as increased postnatal neurogenesis (16 –18), which is also increased by VEGF (19, 20).	info:pmid/21343256#cont:31	Endocrine system {Organ urn:agi-ncimorgan:C1280975}
VEGFA --+> neurogenesis	Regulation	VEGF also promotes neurogenesis, learning, memory and inhibition of apoptosis ( ).	info:pmid/16530019#body:2	
VEGFA --+> neurogenesis	Regulation	There is also evidence that vascular endothelial growth factor regulates neurogenesis.	info:pmid/21937982#cont:156	Hippocampus {Organ urn:agi-ncimorgan:C0019564}
VEGFA --+> neurogenesis	Regulation	Vascular endothelial growth factor (VEGF) stimulates neurogenesis in vitro and in vivo.	info:pmid/24046321#cont:414	Blood Vessels {Organ urn:agi-ncimorgan:C0005847}
VEGFA --+> neurogenesis	Regulation	Thus, VEGF appears to be pivotal for both exercise-induced neurogenesis and angiogenesis.	info:pmid/18474414#body:93	
VEGFA --+> neurogenesis	Regulation	Both IGF-1 and VEGF individually mediate part of the effect of running on neurogenesis ( ).	info:pmid/21056618#body:403	Hippocampus {Organ urn:agi-ncimorgan:C0019564}
VEGFA --+> neurogenesis	Regulation	Moreover, VEGF promotes exercise-induced neurogenesis in mice [111].	info:pmid/24251394#cont:261	
VEGFA --+> neurogenesis	Regulation	These studies suggested that vascular endothelial growth factor might be important in neurogenesis.	info:pmid/19232476#body:128	
VEGFA --+> neurogenesis	Regulation	This is the case of VEGF that has been shown to positively regulate neurogenesis [284].	info:pmid/22394166#cont:485	Nervous system {Organ urn:agi-ncimorgan:C0027763}
VEGFA --+> neurogenesis	Regulation	In addition, VEGF stimulates neurogenesis in vivo and in vitro .	info:pmid/17854994#body:77	Retina {Organ urn:agi-ncimorgan:C1962966}
VEGFA --+> neurogenesis	Regulation	Fabel et al. found that VEGF is necessary for physical exercise–induced hippocampal neurogenesis.76	info:pmid/22856427#cont:118	
VEGFA --+> neurogenesis	Regulation	Finally, VEGF in mammals affects both angiogenesis and neurogenesis in the hippocampus [71].	info:pmid/21891868#cont:175	Brain {Organ urn:agi-ncimorgan:C1269537}
VEGFA --+> neurogenesis	Regulation	BDNF and VEGF are crucially involved in the processes of neurogenesis and synaptic plasticity.	info:pmid/23950576#cont:247	
VEGFA --+> neurogenesis	Regulation	Our previous study shows that VEGF can also stimulate neurogenesis in adult brain (;).	info:pmid/23182805#body:51	Brain {Organ urn:agi-ncimorgan:C1269537}

VEGFA --+> neurogenesis	Regulation	Intracerebroventricular administration of VEGF also enhanced neurogenesis in a stroke model .	info:pmid/15177192#body:112	Blood Vessels {Organ urn:agi-ncimorgan:C0005847}
VEGFA --+> neurogenesis	Regulation	These findings also support the hypothesis that VEGF accelerates the process of neurogenesis early after grafting.	info:pmid/15992890#body:91	Nerve {Organ urn:agi-ncimorgan:C1280541}
VEGFA --+> neurogenesis	Regulation	VEGF also promotes neurogenesis ( ) and has neuroprotective effects following brain injury ( ).	info:pmid/24252224#body:106	Nervous system {Organ urn:agi-ncimorgan:C0027763}
VEGFA --+> neurogenesis	Regulation	Recently, we found that Vascular endothelial growth factor also stimulate neurogenesis in vitro and in vivo.	info:pmid/21275797#cont:194	subventricular zone {Organ urn:agi-ncimorgan:C0521406}
VEGFA --+> neurogenesis	Regulation	For example, vascular endothelial growth factor has been shown to increase adult hippocampal neurogenesis ( ).	info:pmid/21983279#body:141	Microvessels {Organ urn:agi-ncimorgan:C2350570}
VEGFA --+> neurogenesis	Regulation	This indicates that hippocampal plasticity and neurogenesis are mediated by Vascular endothelial growth factor.	info:pmid/17282988#body:388	Hippocampus {Organ urn:agi-ncimorgan:C0019564}
VEGFA --+> neurogenesis	Regulation	Importantly, both erythropoietin and VEGF stimulate neurogenesis (Studer et al., 2000; Sun et al., 2003).	info:pmid/23303921#cont:413	Brain {Organ urn:agi-ncimorgan:C1269537}
VEGFA --+> neurogenesis	Regulation	VEGF, together with BDNF, become focused as an important factor for the running-induced neurogenesis ( ).	info:pmid/17397954#body:86	
VEGFA --+> neurogenesis	Regulation	Vascular endothelial growth factor (VEGF) promotes adult neurogenesis ( ) and stimulates stem cells.	info:pmid/17459722#body:50	
VEGFA --+> neurogenesis	Regulation	Vascular endothelial growth factor also promotes neurogenesis after cerebral ischemia (Sun et al, 2003).	info:pmid/21772310#cont:207	Brain {Organ urn:agi-ncimorgan:C1269537}
VEGFA --+> neurogenesis	Regulation	Adult hippocampal neurogenesis is regulated by growth factors, including BDNF, VEGF, and IGF-1 .	info:pmid/20670674#body:74	Hippocampus {Organ urn:agi-ncimorgan:C0019564}
VEGFA --+> neurogenesis	Regulation	As an angiogenic protein, VEGF promotes neurogenesis after central nervous system injury.	info:pmid/20850508#body:87	Central Nervous System {Organ urn:agi-ncimorgan:C0927232}

VEGFA --+> neurogenesis	Regulation	Furthermore, it has been shown how VEGF administration after Traumatic brain injury promotes neurogenesis ( ).	info:pmid/22917609#body:20	
VEGFA --+> neurogenesis	Regulation	Recent reports demonstrated that VEGF stimulates also neurogenesis in vivo and in vitro and exerts neuroprotective effects.	info:pmid/15763178#body:12	neocortex {Organ urn:agi-organ:C0175173}
VEGFA --+> neurogenesis	Regulation	[Changing of the expression of VEGF genes encoded important regulators of angiogenesis and neurogenesis under hypoxic and ischemic conditions].	info:pmid/23350126#title:1	
VEGFA --+> neurogenesis	Regulation	Neuronal expression of VEGF has also been proposed to play an important role in the regulation of neurogenesis .	info:pmid/23470903#body:227	
VEGFA --+> neurogenesis	Regulation	Hence, we believe that the neuronal expression of VEGF most likely regulates neurogenesis in the subgranular zone.	info:pmid/19571140#body:421	
VEGFA --+> neurogenesis	Regulation	Hobson et al. showed that following sciatic nerve axotomy, Vascular endothelial growth factor induced neurogenesis .	info:pmid/16872810#body:124	sciatic nerve {Organ urn:agi-ncimorgan:C0036394}
VEGFA --+> neurogenesis	Regulation	We thus believe that a combination of VEGF with a diuretic may represent an attractive approach, not only to enhance neurogenesis, but also to reduce the ...	info:pmid/21091268#cont:189	
VEGFA --+> neurogenesis	Regulation	VEGF overexpression enhances striatal neurogenesis in brain of adult rat after a transient middle cerebral artery occlusion.	info:pmid/17061257#title:1	
VEGFA --+> neurogenesis	Regulation	VEGF secreted from endothelial cells promotes neural stem cell proliferation and neurogenesis.	info:pmid/23470250#body:214	
VEGFA --+> neurogenesis	Regulation	Growth factors, such as vascular endothelial growth factor and heparin-binding epidermal growth factor, can also increase adult neurogenesis .	info:pmid/18638527#body:81	
VEGFA --+> neurogenesis	Regulation	Intraventricular infusion of VEGF increases neurogenesis in the subventricular zone and dentate gyrus of adult mice.	info:pmid/19375666#body:107	

VEGFA --+> neurogenesis	Regulation	Or inversely, IGF or vascular endothelial growth factor may enhance neurogenesis which in turn may somehow induce cell death.	info:pmid/16793155#body:135	
VEGFA --+> neurogenesis	Regulation	VEGF, an angiogenic factor, not only induces angiogenesis, but also stimulates neurogenesis and axonal outgrowth ( ).	info:pmid/16730914#body:14	
VEGFA --+> neurogenesis	Regulation	In addition, VEGF was also believed to be involved in the regulation of neurogenesis after global and focal ischemia .	info:pmid/16098951#body:85	Central Nervous System {Organ urn:agi-ncimorgan:C0927232}
VEGFA --+> neurogenesis	Regulation	EGFR, VEGF, BDNF, HGF, PDGF, Shh, Wnt, and Sox are involved in neurogenesis and tumorigenesis .	info:pmid/17673180#body:82	
VEGFA --+> neurogenesis	Regulation	(2003) vascular endothelial growth factor-induced neuroprotection, neurogenesis, and angiogenesis after focal cerebral ischemia.	info:pmid/20668522#cont:431	
VEGFA --+> neurogenesis	Regulation	Sun concluded that VEGF reduced the infarct size in his rats and enhanced cerebral angiogenesis and neurogenesis [101].	info:pmid/22850316#cont:285	Cerebral cortex {Organ urn:agi-ncimorgan:C0007776}
VEGFA --+> neurogenesis	Regulation	VEGF mediates neurogenesis by augmenting the proliferation and differentiation of neural progenitor cells ( ).	info:pmid/23764464#body:141	
VEGFA --+> neurogenesis	Regulation	In contrast, delayed administration of VEGF (>24 h after ischemia) improves these measures (2– 4) and enhances neurogenesis (5).	info:pmid/23456363#cont:17	
VEGFA --+> neurogenesis	Regulation	VEGF may also be beneficial to neurons by promoting neurogenesis182 and by mediating mechanisms of hypoxic preconditioning.183 .	info:pmid/18806052#body:212	
VEGFA --+> neurogenesis	Regulation	In addition, VEGF stimulates neurogenesis in response to environmental enrichment thus contributing to hippocampal plasticity [14].	info:pmid/23202441#cont:31	
VEGFA --+> neurogenesis	Regulation	VEGF may modulate neurogenesis in constitutive neurogenic and non-neurogenic regions of adult mammalian brains ( ).	info:pmid/21807419#body:107	Brain {Organ urn:agi-ncimorgan:C1269537}
VEGFA --+> neurogenesis	Regulation	Vascular endothelial growth factor , in addition to regulating angiogenesis, is also a regulator of	info:pmid/18786562#body:129	Hippocampus {Organ urn:agi-

		hippocampal neurogenesis.		ncimorgan:C0019564}
VEGFA --+> neurogenesis	Regulation	VEGF stimulates neurogenesis in vivo and in vitro and this might be of interest in the context of lesion-induced neurogenesis .	info:pmid/15212950#body:130	Cerebral cortex {Organ urn:agi-ncimorgan:C0007776}
VEGFA --+> neurogenesis	Regulation	VEGF can promote dentate neurogenesis in both the intact and the injured adult brain following intracerebroventricular administration .	info:pmid/18374402#body:349	Brain {Organ urn:agi-ncimorgan:C1269537}
VEGFA --+> neurogenesis	Regulation	Stimulation of angiogenesis leads to an increase of neurogenesis mediated by angiogenic factors, such as angiopoietin-1 and VEGF .	info:pmid/23266366#body:129	Microvessels {Organ urn:agi-ncimorgan:C2350570}
VEGFA --+> neurogenesis	Regulation	A peripheral blockade of VEGF abolished exercise-induced neurogenesis, whereby the baseline levels of neurogenesis were not affected ( ).	info:pmid/20692101#body:170	Hippocampus {Organ urn:agi-ncimorgan:C0019564}
MDK --+> vascularization	Regulation	Both thymidine phosphorylase and midkine are important for angiogenesis in laryngeal squamous cell carcinoma.	info:pmid/18476626#abs:7	
MDK --+> vascularization	Regulation	Moreover, MDK downregulates VEGF-A-induced neovascularization and vascular permeability in vivo.	info:pmid/18392135#abs:5	Microvessels {Organ urn:agi-ncimorgan:C2350570}
MDK --+> vascularization	Regulation	These results suggest that midkine may play important roles in malignant transformation and tumor angiogenesis in salivary gland tumors.	info:pmid/20637680#abs:6	
MDK --+> vascularization	Regulation	Midkine small interfering RNA suppressed mainly cell proliferation and slightly angiogenesis, whereas paclitaxel enhanced apoptosis and slightly suppressed angiogenesis.	info:pmid/16832814#abs:9	
MDK --+> vascularization	Regulation	Exogenous midkine induced neovascularization in a chorioallantoic membrane assay compared with negative control as measured by counting the number of branching points per visual field (1,074 ± 54 vs. 211 ± 70; $P < 0.05$).	info:pmid/22707563#abs:6	
MDK --+> vascularization	Regulation	The midkine treatment also increased collagen accumulation and facilitated angiogenesis in the infarcted area, and the viable muscle area after	info:pmid/18222265#abs:8	

		myocardial infarction dose-dependently increased.		
MDK --+> vascularization	Regulation	There is mounting evidence that Midkine plays a significant role in carcinogenesis-related activities, such as proliferation, migration, anti-apoptosis, mitogenesis, transforming, and angiogenesis.	info:pmid/19152444#abs:5	
MDK --+> vascularization	Regulation	Heparin-related growth factors (FGFs, Midkine family), VEGF or endothelin could be more particularly implicated in metastatic progression by stimulating cell motility, angiogenesis and metastatic implantation by a two-way cooperation between the tumor and the stroma in which it is implanted.	info:pmid/9685995#abs:12	
MDK --+> vascularization	Regulation	midkine plays a significant role in angiogenesis.	info:pmid/19698107#body:255	Lung {Organ urn:agi-ncimorgan:C1278908}
MDK --+> vascularization	Regulation	Cytokines, such as Midkine, VEGF, BFGF and IL-8, play a role in tumor neovascularization.	info:pmid/22051879#cont:200	
MDK --+> vascularization	Regulation	Various studies have shown that MDK is involved in mitogenesis, transformation, survival, migration and angiogenesis .	info:pmid/19883768#body:4	
MDK --+> vascularization	Regulation	In addition, Midkine enhances plasminogen activator and plasmin activity in tissue repair and angiogenesis .	info:pmid/15823575#body:6	
MDK --+> vascularization	Regulation	Several studies revealed that overexpression of midkine promoted tumor growth, survival, invasion, and tumor angiogenesis [7–9].	info:pmid/22235180#cont:19	
MDK --+> vascularization	Regulation	On the other hand, midkine also regulates inflammatory responses and in vivo angiogenesis .	info:pmid/19535098#body:10	Brain {Organ urn:agi-ncimorgan:C1269537}
MDK --+> vascularization	Regulation	The overexpression of Midkine appears to contribute to the malignant phenotypes of glioblastoma such as enhanced angiogenesis.	info:pmid/21375488#cont:389	
MDK --+> vascularization	Regulation	Various studies have shown that MDK and PTN are involved in mitogenesis, transformation, survival, migration and angiogenesis .	info:pmid/22871361#body:7	Mesenchyme {Organ urn:agi-ncimorgan:C0162415}
MDK --+> vascularization	Regulation	In vitro studies demonstrated that Midkine promotes angiogenesis (3), cell growth (4), and cell	info:pmid/15197188#body:66	Blood Vessels {Organ urn:agi-

		migration (5).		ncimorgan:C0005847}
MDK --+> vascularization	Regulation	Finally, midkine inhibits the induction of apoptosis (23), promotes angiogenesis (24), and stimulates tyrosine phosphorylation of several cellular proteins, ...	info:pmid/17204554#body:76	
MDK --+> vascularization	Regulation	Midkine, a member of a highly conserved and developmentally regulated gene family, has a critical role in cell growth, survival, migration, angiogenesis, and carcinogenesis.7	info:pmid/21917682#cont:22	
MDK --+> vascularization	Regulation	However, it was reported that Midkine downregulated vascular endothelial growth factor-A-induced neovascularisation and vascular permeability in a recent study (van der Horst et al, 2008).	info:pmid/18682710#body:211	
MDK --+> vascularization	Regulation	Since MDK is known to induce angiogenesis [29], we sought whether inhibition of angiogenesis by iMDK might in part contribute to the reduction of lung tumors in vivo.	info:pmid/23976985#cont:194	
MDK --+> vascularization	Regulation	Previously, the antiapoptotic effect and angiogenesis induced by midkine were reported to be associated with the activation of ERK and phosphatidylinositol 3-kinase /AKT.	info:pmid/24291499#body:86	Heart {Organ urn:agi-ncimorgan:C1281570}
MDK --+> vascularization	Regulation	Under stress microenvironments many other secreted proteins such as osteopontin (OPN), pleiotrophin (PTN) and midkine also played pivotal roles in cell survival, proliferation, angiogenesis and metastasis .	info:pmid/19328625#body:92	
MDK --+> vascularization	Regulation	Midkine has definitely been proven to be involved in the pathogenesis and development of a variety of solid tumors by promoting proliferation and angiogenesis, as well as by inhibiting apoptosis .	info:pmid/19409372#body:98	
MDK --+> vascularization	Regulation	Midkine and the related PTN (pleiotrophin) are heparin-binding growth factors known to play multiple roles in anti-apoptosis, angiogenesis, tissue	info:pmid/23418741#cont:14	

		repair, tumorigenesis and neurogenesis in mammals [1].		
MDK --+> vascularization	Regulation	It has been shown that Midkine promotes cell proliferation, differentiation, survival and migration, and is involved in a variety of biological processes, including neuronal development, angiogenesis and oncogenesis	info:pmid/24516630#cont:25	
MDK --+> vascularization	Regulation	The induction of angiogenesis is mediated by several angiogenic factors, such as the fibroblast growth factor family members, vascular endothelial growth factor (VEGF), platelet-derived endothelial cell growth factor (PDECGF), angiogenin , and midkine .	info:pmid/9933044#body:2	
MDK --+> vascularization	Regulation	Furthermore, midkine is known to enhance plasminogen activator and plasmin activity in bovine aortic endothelial cells (21, 22), which suggests that midkine may also have a role in tissue repair and angiogenesis.	info:pmid/12122009#body:56	Aorta {Organ urn:agi-ncimorgan:C1278934}
MDK --+> vascularization	Regulation	Midkine is involved in the development of cancer through diverse biological activities, including enhancement of fibrinolytic activity , anti-apoptotic effect , induction of mitogenesis , transformation , angiogenesis and chemotaxis .	info:pmid/17267033#body:4	
MDK --+> vascularization	Regulation	midkine and PTN have been shown in vitro to promote neurite outgrowth (Kaneda et al., 1996), neuronal differentiation, neuronal survival (Kikuchi et al., 1993) and tumor angiogenesis (for review see Kurtz et al., 1995; Zhang and Deuel, 1999).	info:pmid/11244508#body:81	
MDK --+> vascularization	Regulation	Moreover, the angiogenic action of Midkine in tumors is strongly suggested by the observation that transfection of the breast carcinoma line MCF-7 with Midkine accelerates tumor growth and increases tumor vascularity after cell implantation in nude mice .	info:pmid/12648577#body:86	
MDK --+>	Regulation	Consistent with these results, transfection of the	info:pmid/17622248#body:	

vascularization		breast carcinoma line MCF-7 with Midkine accelerates tumour growth and increases tumour vascularity after implantation of Midkine-overexpressing MCF-7 cells into nude mice (Choudhuri et al, 1997).	216	
MDK --+> vascularization	Regulation	In accordance with its high expression in various malignant tumors, midkine exerts cancer-related activities in the process of carcinogenesis, including transformation, fibrinolysis, cell migration, enhancement of cell survival and angiogenesis, and anti-apoptotic effects .	info:pmid/22841548#body:96	Head of pancreas {Organ urn:agi-ncimorgan:C0227579}
MDK --+> vascularization	Regulation	Consistent with these results, the angiogenic action of midkine in tumours is strongly suggested by the observation that transfection of the breast carcinoma line MCF-7 with midkine accelerates tumour growth and increases tumour vascularity after cell implantation in nude mice .	info:pmid/17110085#body:82	
MDK --+> vascularization	Regulation	midkine promotes angiogenesis (Choudhuri et al., 1997[Go]), neurite outgrowth (Muramatsu et al., 1993[Go]), survival of neurons (Owada et al., 1999[Go]), cell growth (Muramatsu and Muramatsu, 1991[Go]), fibrinolysis (Kojima et al., 1995[Go]) and cell migration (Takada et al., 1997[Go]; Maeda et al., 1999[Go]; Horiba et al., 2000[Go]).	info:pmid/12077357#body:53	
MDK --+> vascularization	Regulation	midkine transforms NIH3T3 cells (Kadomatsu et al, 1997), enhances fibrinolysis (Kojima et al, 1995), and promotes cell growth (Muramatsu and Muramatsu, 1991; Muramatsu et al, 1993; Takei et al, 2001), cell survival (Qi et al, 2000), cell migration (Takada et al, 1997; Maeda et al, 1999; Horiba et al, 2000; Qi et al, 2001), and angiogenesis (Choudhuri et al, 1997).	info:pmid/12771916#body:44	
MDK --+> vascularization	Regulation	midkine was previously shown to associate with several cell surface proteins that could be serving as its signaling receptor supporting the notion that	info:pmid/18851943#body:48	

		midkine might form multiple protein complexes or enhance a multiprotein complex formation in order to perform its functions as a regulator of cell proliferation, cell differentiation, cell migration and cell invasiveness, therefore contributing to tumorigenesis, metastasis, and angiogenesis .		
MDK --+> vascularization	Regulation	Conclusions: Midkine may play important roles in malignant transformation and tumour angiogenesis in salivary gland tumours.	info:doi/10.1016/j.ijom.2009.03.497#body:8	
MDK --+> vascularization	Regulation	Midkine , a 13-kDa heparin-binding growth factor, has been proposed to mediate various developmental processes, including angiogenesis, cell migration, and proliferation in various tissues including the developing lung.	info:doi/10.1016/j.carpath.2004.03.041#body:1	Lung {Organ urn:agi-ncimorgan:C1278908}
HSPD1 ---> vascularization	Regulation	Furthermore, Hsp60 from C. pneumoniae has been shown to promote the growth of vascular smooth muscle cells .	info:pmid/15453708#abs:3	
HSPD1 ---> vascularization	Regulation	Furthermore, HSP65 also attenuated tumor-induced angiogenesis in the intradermal model and pulmonary metastasis in the tail intravenously injected model of mice.	info:pmid/23135134#abs:6	
HSPD1 ---> vascularization	Regulation	Secondly, HSP65-X10-ßhCGCTP37 may hamper angiogenesis via down-regulation of MMPs.	info:pmid/19913113#body:209	Blood Vessels {Organ urn:agi-ncimorgan:C0005847}
HSPD1 ---> vascularization	Regulation	Helicobacter pylori-derived Heat shock protein 60 enhances angiogenesis via a CXCR2-mediated signaling pathway.	info:pmid/20580690#title:1	
HSPD1 ---> vascularization	Regulation	(2010) Helicobacter pylori-derived Heat shock protein 60 enhances angiogenesis via a CXCR2-mediated signaling pathway.	info:pmid/23555707#cont:596	
HSPD1 ---> vascularization	Regulation	Furthermore, Hsp60 induces proliferation of vascular smooth muscle cells (38), which might also contribute to cardiovascular disease (39).	info:pmid/22315307#cont:254	
HSPD1 --->	Regulation	Previously, Hochleitner et al described the release of	info:pmid/21330606#cont:	

vascularization		HSP60 after shear stress,29 and others showed the capability of HSP60 to bind directly to TLR4, thereby initiating proliferation of vascular smooth muscle cells.30	212	
HSPD1 ---> vascularization	Regulation	The role of Helicobacter pylori heat shock protein 60 in gastric carcinogenesis has been proved to promote inflammation, angiogenesis, and migration abilities of monocytes and gastric tumor cells .	info:pmid/21565524#body:6	
VEGFA ---> brain development	Regulation	These data suggest that VEGF and VEGFR-2 are likely involved in several aspects of human brain development.	info:pmid/20084021#abs:8	Prosencephalon {Organ urn:agi-ncimorgan:C0085140}
VEGFA ---> brain development	Regulation	We show that VEGF produced by the embryonic neuroectoderm is required for the vascularization and the development of the brain.	info:pmid/14983237#abs:4	Neuroectoderm {Organ urn:agi-ncimorgan:CL321642}
VEGFA ---> brain development	Regulation	Most functions of VEGF that are essential for proper brain development are, in fact, dispensable in the adult brain as was clearly demonstrated using a conditional brain-specific VEGF loss-of-function approach.	info:pmid/23475068#abs:4	Brain {Organ urn:agi-ncimorgan:C1269537}
VEGFA ---> brain development	Regulation	Both VEGF and reelin play important roles in neuronal migration during brain development .	info:pmid/23916658#body:72	
VEGFA ---> brain development	Regulation	Thus, a critical physiological dose of vascular endothelial growth factor is essential in normal cerebral development.	info:pmid/15571667#body:132	Prosencephalon {Organ urn:agi-ncimorgan:C0085140}
VEGFA ---> brain development	Regulation	Based on the fact that the onset of VEGF120 expression is 2 weeks after birth (supplemental Fig. 1, available at www.jneurosci.org as supplemental material), these results suggest that VEGF enhances postnatal brain development, to some extent.	info:pmid/19118187#body:222	Brain {Organ urn:agi-ncimorgan:C1269537}
PEBP1 ---> neurogenesis	Regulation	Based on these previous data, HCNP/HCNP-pp might be involved in neurogenesis and/or gliogenesis in adult rat progenitor cells via novel mechanisms other than its function in inhibition of the Erk	info:pmid/20206149#body:87	Hippocampus {Organ urn:agi-ncimorgan:C0019564}

		pathway.		
EMX1 ---> brain development	Regulation	Emx family homeobox genes, Emx1 and Emx2, play an essential role in rostral brain development in mammalian embryos.	info:pmid/12617801#abs:1	
EMX1 ---> brain development	Regulation	Transcription factors Emx1, 2, and Otx1, 2 can play roles in the rostral brain development.	info:pmid/9113125#body:172	hindbrain {Organ urn:agi-ncimorgan:C0035507}
EMX1 ---> brain development	Regulation	Like the fruit-fly counterpart, Emx1 and Emx2 are involved in brain development and are prevalently expressed in the cerebral cortex during embryogenesis between days E8.5 and E16.	info:pmid/16197942#body:139	Cerebral cortex {Organ urn:agi-ncimorgan:C0007776}
MDK ---> neurogenesis	Regulation	Midkine ameliorates ischemic injury in the heart and brain, enhances oocyte maturation, and is involved in neurogenesis.	info:pmid/24460672#abs:2	Brain {Organ urn:agi-ncimorgan:C1269537}
MDK ---> neurogenesis	Regulation	Comparing the mode of Midkine expression between Xenopus and the mouse, we propose that Midkine plays evolutionally conserved roles in neurogenesis and development of the craniofacial architecture of ectomesenchymal origin.	info:pmid/8537332#abs:7	Neuroectoderm {Organ urn:agi-ncimorgan:CL321642}
MDK ---> neurogenesis	Regulation	Midkine gene transfer protects against focal brain ischemia and augments neurogenesis.	info:pmid/19535098#title:1	
MDK ---> neurogenesis	Regulation	Midkine participates in cell growth, survival, migration, neurogenesis, and carcinogenesis.	info:pmid/18329695#body:3	
MDK ---> neurogenesis	Regulation	midkine, a RA-inducible molecule is considered to play important roles in fetal nervous system development and neurogenesis, as is Xenopus midkine .	info:pmid/19909807#body:146	Kidney {Organ urn:agi-ncimorgan:C1278978}
MDK ---> neurogenesis	Regulation	Among their diverse biological roles, Ptn and Midkine have been involved in neurogenesis by promotion of neurite outgrowth and nerve cell migration ( ).	info:pmid/16914133#body:11	
MDK ---> neurogenesis	Regulation	Both midkine and pleiotrophin/heparin-binding growth-associated molecule have neurotrophic activities and are considered to be involved in	info:pmid/10683378#body:55	Kidney {Organ urn:agi-ncimorgan:C1278978}

		neurogenesis and tumor progression (14-17).		
MDK ---> neurogenesis	Regulation	Midkine , a heparin-binding growth factor, has putative functions in neurogenesis, neurodifferentiation and cell survival in developing brain and tissue culture .	info:pmid/15450683#body:1	
MDK ---> neurogenesis	Regulation	Midkine and the related PTN (pleiotrophin) are heparin-binding growth factors known to play multiple roles in anti-apoptosis, angiogenesis, tissue repair, tumorigenesis and neurogenesis in mammals [1].	info:pmid/23418741#cont:14	
MDK ---> neurogenesis	Regulation	MDK gene transfer protects against focal brain ischemia and augments neurogenesis [13], and it may also have a protective role against cardiac ischemia and reperfusion injury through reducing apoptotic reaction [14].	info:pmid/24372230#cont:25	Peripheral Nerves {Organ urn:agi-ncimorgan:C0031119}
MDK ---> neurogenesis	Regulation	In light of these data, we propose that in the vertebrate central nervous system Midkine is a component of the complex environment of extrinsic regulatory molecules (for example) that functions, perhaps in an autocrine manner, to regulate developmental neurogenesis.	info:pmid/23111152#cont:246	Central Nervous System {Organ urn:agi-ncimorgan:C0927232}
MDK ---> neurogenesis	Regulation	These findings show that Midkine-dependent Alk signaling is essential for in vitro proliferation of immature sympathetic neurons and raise the question of to what extent sympathetic neurogenesis is controlled by Midkine/Alk in the embryo.	info:pmid/21989914#cont:188	
MDK ---> neurogenesis	Regulation	In addition, activation of astrocytes increases the production of FGF-2, midkine, nerve growth factor (NGF), insulin-like growth factor-1 (IGF-1), brain-derived neurotrophic factor (BDNF), and other trophic factors that contribute to the survival and neurogenesis of neurons and neural stem cell.	info:pmid/18533253#body:9	
EMX1 --->	Regulation	Our data suggest that deletion of the Emx1 gene	info:pmid/17490651#abs:9	Upper Extremity {Organ

neurogenesis		reduces hippocampal neurogenesis and affects higher motor function that requires extensive learning.		urn:agi-ncimorgan:C1140618}
EMX1 ---> neurogenesis	Regulation	C, Examples of enhanced green fluorescent protein+ cells in granule cell layer labeled with bromodeoxyuridine, indicating that Emx1 lineage participates in adult neurogenesis.	info:pmid/17596436#body:229	
PRDX1 ---> neurogenesis	Regulation	Their gain-of-function analysis shows that, although misexpression of Prdx1 alone has little effect on neuronal development, Prdx1 synergistically promotes motor neuron differentiation when combined with doses of GDE2 too low to trigger neurogenesis.	info:pmid/19766560#body:17	
PEBP1 ---| vascularization	Regulation	Loss of RKIP enhanced both angiogenesis and vascular invasion, and protected against apoptosis.	info:pmid/15269597#abs:10	
PEBP1 ---| vascularization	Regulation	To evaluate the effect of RKIP-mediated tumor growth and angiogenesis in vivo, we performed a CAM assay.	info:pmid/22292035#cont:223	
PEBP1 ---| vascularization	Regulation	Reduced RKIP expression has been shown to affect cell growth, angiogenesis, apoptosis and gene integrity (8).	info:pmid/23674108#cont:27	Brain {Organ urn:agi-ncimorgan:C1269537}
PEBP1 ---| vascularization	Regulation	C) Hematoxylin-eosin staining of the paraffin embedded tumors showing the higher vascularization induced by RKIP inhibition.	info:pmid/23527098#cont:340	
PEBP1 ---| vascularization	Regulation	Loss of RKIP was associated with metastasis development, and increased angiogenesis and vascular invasion were suggested as possible mechanisms (18).	info:pmid/18230656#body:199	Prostate {Organ urn:agi-ncimorgan:C1278980}
PEBP1 ---| vascularization	Regulation	Forced overexpression of RKIP in human prostate cancer cells reduces tumor angiogenesis and metastasis in vivo in an orthotopic murine xenograft model .	info:pmid/20855151#body:11	Breast {Organ urn:agi-ncimorgan:C0006141}
PEBP1 ---| vascularization	Regulation	Additionally, it was noted that the vasculature in the primary tumors of RKIP overexpressing cells was less	info:pmid/15686621#body:112	

		developed, suggesting that RKIP could function as a metastasis suppressor by decreasing angiogenesis and vascular invasion.		
VEGFA ---> synaptic transmission	Regulation	VEGF is a potent mitogen and survival factor for endothelial cells and neurons, and modulator of synaptic transmission.	info:pmid/18061540#abs:4	
VEGFA ---> synaptic transmission	Regulation	Our findings suggest that VEGF released from neuronal cells plays a local role in Ca2+ influx and synaptic transmission that may influence the generation of long-term changes in synaptic efficacy.	info:pmid/18221855#abs:9	
VEGFA ---> synaptic transmission	Regulation	VEGF also decreased synaptic transmission in slices from normal rats.	info:pmid/18065154#body:167	Hippocampus {Organ urn:agi-ncimorgan:C0019564}
VEGFA ---> synaptic transmission	Regulation	VEGF also modulates synaptic transmission (12), suggesting that the effects of this factor are multifaceted.	info:pmid/17360578#body:34	Hippocampus {Organ urn:agi-ncimorgan:C0019564}
VEGFA ---> synaptic transmission	Regulation	Also, increased VEGF gene expression facilitates excitatory synaptic transmission and promotes neuronal plasticity .	info:pmid/22985858#body:177	hypothalamus {Organ urn:agi-ncimorgan:C0020663}
VEGFA ---> synaptic transmission	Regulation	Some evidence supports the possibility that VEGF-A modulates synaptic transmission directly by altering calcium influx (Fig. 1C).	info:pmid/23076132#cont:122	Vertebral column {Organ urn:agi-ncimorgan:C1267072}
VEGFA ---> synaptic transmission	Regulation	VEGF released from neurons may act, at least in part, via VEGF receptor 2 to enhance excitatory synaptic transmission.	info:pmid/20427666#body:429	
VEGFA ---> synaptic transmission	Regulation	It has been shown that VEGF can suppress glutamatergic synaptic transmission in all major synapses in the hippocampus [21,22].	info:pmid/22808185#cont:155	Hippocampus {Organ urn:agi-ncimorgan:C0019564}
VEGFA ---> synaptic transmission	Regulation	The results provide the first evidence that VEGF influences synaptic transmission in the brain and a potential explanation for the neuroprotective actions of VEGF.	info:pmid/16192378#body:65	Hippocampus {Organ urn:agi-ncimorgan:C0019564}
VEGFA ---> synaptic	Regulation	As recently reviewed by , VEGF is a potent mitogen and survival factor for endothelial cells and neurons,	info:pmid/19596038#body:129	

transmission		as well as a modulator of synaptic transmission.		
VEGFA ---> synaptic transmission	Regulation	VEGF influences synaptic plasticity in hippocampus-dependent processes, such as learning and memory ( ), and modulates synaptic transmission ( ).	info:pmid/19059450#body:3	Brain {Organ urn:agi-ncimorgan:C1269537}
VEGFA ---> synaptic transmission	Regulation	VEGF also influences synaptic plasticity in hippocampus-dependent processes, such as learning and memory ( ), and modulates synaptic transmission ( ).	info:pmid/21515361#body:28	Blood Vessels {Organ urn:agi-ncimorgan:C0005847}
VEGFA ---> synaptic transmission	Regulation	Previous studies have demonstrated that vascular endothelial growth factor is regulated by neuronal activity and that vascular endothelial growth factor increases hippocampal long-term potentiation and synaptic transmission (Cao et al, 2004).	info:pmid/17851537#body:454	
VEGFA ---> synaptic transmission	Regulation	Moreover, VEGF may influence the brain mechanism of synaptic transmission: electrophysiological studies have shown that this growth factor decreases the evoked response of hippocampal neurons to synaptic stimulation through the damping of the glutamatergic pathway ( ).	info:pmid/21570438#body:9	Brain {Organ urn:agi-ncimorgan:C1269537}
VEGFA ---> synaptic transmission	Regulation	It is recently found that VEGF released from cultured hippocampus neurons plays a local role in Ca2+ influx and synaptic transmission in a TRPC dependent manner .	info:doi/10.1016/j.neulet.2012.03.016#body:75	Hippocampus {Organ urn:agi-ncimorgan:C0019564}
CFL1 ---> synaptic transmission	Regulation	Because the phosphorylation levels and activities of LIMK and cofilin contribute to hippocampal excitatory synaptic transmission and plasticity ( ), fasudil hydrochloride might protect synaptic structure and function by inhibiting LIMK2 and cofilin phosphorylation.	info:doi/10.1016/j.neuroscience.2011.10.030#body:167	Hippocampus {Organ urn:agi-ncimorgan:C0019564}
TUBA1A ---> brain development	Regulation	Interestingly, mutation analysis of the other tubulin genes that are also highly expressed in the developing nervous system, TUBA1B, TUBA1C and TUBB3, did not yield any deleterious mutations , suggesting that TUBA1A has a unique role in brain	info:pmid/17997185#body:62	Nervous system {Organ urn:agi-ncimorgan:C0027763}

		development, perhaps through interactions with its distinct protein-binding partners.		
YWHAE ---> synaptic transmission	Regulation	Taken together, it can be speculated that 14-3-3 epsilon is involved in synaptogenesis and/or synaptic transmissions and Cadmium possibly affects them through phosphorylation of 14-3-3 epsilon.	info:pmid/18835323#body:124	
PGK1 ---> vascularization	Regulation	These findings indicate that overexpression of PGK-1 in LLC-1 reduces the COX-2 expression, and, in turn, affect PGE2, cell invasion, angiogenesis, and the immune functions, and finally inhibit the tumor progression.	info:pmid/18814280#abs:12	
PGK1 ---> vascularization	Regulation	Overexpression of phosphoglycerate kinase 1 reduced the secretion of vascular endothelial growth factor and decreased tumor angiogenesis.	info:pmid/19299076#body:144	
PGK1 ---> vascularization	Regulation	Phosphoglycerate kinase 1, which has not been associated with the mitotic spindle previously, is essential for glycolysis and also prevents angiogenesis in tumors [61].	info:pmid/21647379#cont:164	
PGK1 ---> vascularization	Regulation	These gene products include the glycolytic enzymes, phosphoglycerate kinase-1 (PGK-1), and lactate dehydrogenase-A (LDH-A) ( ) and those gene products that promote increased oxygen delivery via angiogenesis (vascular endothelial growth factor [VEGF]) ( ) or erthyropoiesis (erthyropoetin [Epo]) ( ).	info:pmid/15850924#body:16	
YWHAE ---| axon guidance	Regulation	We next compared 14-3-3e-dependent axon guidance defects to those resulting from manipulating Sema-1a/PlexA signaling.	info:pmid/22500634#body:33	Nerve {Organ urn:agi-ncimorgan:C1280541}
PKM --+> vascularization	Regulation	An interesting hypothesis of the authors is that Tumor M2-PK released from tumors might stimulate angiogenesis by binding to TEM8.	info:pmid/20156581#body:216	
PKM --+> vascularization	Regulation	One possible mechanism to explain the tumor-promoting properties of PKM1 and PKM2 fibroblasts could be an increase in tumor angiogenesis.	info:pmid/22236875#cont:182	
PKM --+>	Regulation	In addition to its effects on transcription of	info:pmid/22824010#body:	

vascularization		metabolic genes, PKM2 stimulates HIF-1- and HIF-2-mediated expression of the VEGFA gene (which encodes vascular endothelial growth factor), thereby promoting angiogenesis ( ) .	81	
ATP5A1 ---| vascularization	Regulation	ATPM-Raf, acts in a dominant negative fashion to suppress angiogenesis.	info:doi/10.1016/j.acra.2007.04.003#body:19	
YWHAE ---> brain development	Regulation	Thus, 14-3-3 e has pivotal roles in neuronal migration and development of the brain.	info:pmid/23078967#body:81	
YWHAE ---> brain development	Regulation	Recently, it was shown that 14-3-3e protein encoded by YWHAE gene is important in brain development and neuronal migration .	info:pmid/15196593#body:146	
CFL1 ---> brain development	Regulation	N-cofilin and ADF have distinct roles in brain development.	info:pmid/17875668#body:48	
CFL1 ---> brain development	Regulation	By contrast, neuronal complexity, brain development, and synaptic function are severely impaired in n-cofilin mutants [6,17].	info:pmid/22046357#cont:321	
CFL1 ---> brain development	Regulation	N-cofilin also controls cell cycle progression in neuronal progenitors in the ventricular zone during brain cortex development [ ].	info:pmid/19740640#body:42	Heart Ventricle {Organ urn:agi-ncimorgan:C0018827}
YWHAZ ---> synaptic transmission	Regulation	In Drosophila, 14-3-3? proteins are enriched at nerve terminals and involved in regulating synaptic transmissions at the neuromuscular junction ( ).	info:pmid/16982421#body:22	Brain {Organ urn:agi-ncimorgan:C1269537}
YWHAZ ---> synaptic transmission	Regulation	Increased expression of 14-3-3z in Alzheimer's disease brain may, therefore, cause synaptic pathology by inhibiting neurite outgrowth, synapse formation, and synaptic transmission.	info:pmid/24367683#cont:451	
PGK1 --+> axon guidance	Regulation	Dosage sensitive genetic interactions indicate that MIG-10 functions with ABI-1 and WVE-1 to mediate axon guidance.	info:pmid/23209429#abs:8	
PGK1 --+> axon guidance	Regulation	Recent work has demonstrated that MIG-10 functions as an effector for Rac during axon guidance.	info:pmid/18951796#body:69	
PGK1 --+>	Regulation	Also, the C. elegans MRL protein Mig-10 uses its	info:pmid/19615876#body:	

axon guidance		affinity to Ena/VASP to promote axon guidance and outgrowth [ ].	61	
PGK1 --+> axon guidance	Regulation	C. elegans MIG-10 plays roles in both Slit-dependent and netrin-dependent axon guidance pathways.	info:pmid/16618541#body:91	Nerve {Organ urn:agi-ncimorgan:C1280541}
PGK1 --+> axon guidance	Regulation	MIG-10/lamellipodin and AGE-1/PI3K promote axon guidance and outgrowth in response to slit and netrin.	info:pmid/24553288#cont:427	
PGK1 --+> axon guidance	Regulation	In C. elegans, mig-10 and unc-34/ena function together to mediate unc-6/netrin-dependent axon guidance decisions [ ].	info:pmid/20417104#body:133	
PGK1 --+> axon guidance	Regulation	MIG-10/lamellipodin and AGE-1/PI3K promote axon guidance and outgrowth in response to slit and netrin.	info:pmid/23628914#cont:347	Colon {Organ urn:agi-ncimorgan:C1281569}
PGK1 --+> axon guidance	Regulation	A recent report has shown that Mig-10, a C. elegans Lpd ortholog, interacts with active Rac, a Rho family protein, and Mig-10 regulates axon guidance downstream of Rac in response to netrin and slit (Quinn et al., 2008).	info:pmid/22699910#cont:331	Dendrites {Organ urn:agi-ncimorgan:C0011305}
PGK1 --+> axon guidance	Regulation	Our data indicate that MIG-10/lamellipodin and MAX-2, a member of the PAK family, act in parallel genetic pathways to mediate axon guidance.	info:doi/10.1016/j.cub.2008.04.050#body:92	
VEGFA --+> axon guidance	Regulation	Similar to Shh and Netrin-1, VEGF-mediated commissural axon guidance requires the activity of Src family kinases.	info:pmid/21658588#abs:6	Commissure {Organ urn:agi-ncimorgan:C1185742}
VEGFA --+> axon guidance	Regulation	Vascular endothelial growth factor (VEGF) and fibroblast growth factor play important roles in the formation of the blood vascular system and in axon guidance, nervous system development and function.	info:pmid/24623082#abs:1	Vascular system {Organ urn:agi-ncimorgan:C0489903}
VEGFA --+> axon guidance	Regulation	However, evidence has emerged that VEGF-A also promotes a wide range of neuronal functions, both in vitro and in vivo, including neurogenesis, neuronal migration, neuronal survival and axon guidance.	info:pmid/22434866#abs:2	
VEGFA --+>	Regulation	However, evidence is still lacking that VEGF-A	info:pmid/21658587#body:	Blood Vessels {Organ

axon guidance		controls axon guidance in vivo.	160	urn:agi-ncimorgan:C0005847}
VEGFA --+> axon guidance	Regulation	One of the best-known examples is VEGF, which regulates neuronal cell migration ( ), axon guidance ( ), turning of leading processes of migrating cerebellar neurons ( ), and dendritogenesis ( ).	info:pmid/21835339#body:133	Cerebellum {Organ urn:agi-ncimorgan:C1268981}
VEGFA --+> axon guidance	Regulation	In turn, classical angiogenic molecules, such as VEGF, participate in neurogenesis (neurovascular niche), neuronal cell migration, axon guidance, dendritogenesis, and oligodendrocyte precursor migration ( ).	info:pmid/24267647#body:66	
VEGFA --+> axon guidance	Regulation	For example, VEGF-A, the most widely studied vascular endothelial growth factor family member, is essential for blood vascular development, but also regulates neuronal generation, survival, migration and axon guidance.5	info:pmid/23076132#cont:31	Blood Vessels {Organ urn:agi-ncimorgan:C0005847}
VEGFA --+> axon guidance	Regulation	Future Npn-1, semaphorin, plexin, VEGF165, and VEGFR structures and costructures will foster an improved understanding of the molecular details involved in assembly of the tripartite functional signaling complexes (sema/Npn/plexin; VEGF 165/Npn/VEGFR) essential to axon guidance and angiogenesis and contribute to the design of molecules to favorably modulate these processes.	info:pmid/12517344#body:155	
VEGFA --+> axon guidance	Regulation	Taken together, these studies are the first to report that vascular endothelial growth factor is essential for proper axon guidance at the CNS midline in vivo.	info:doi/10.1016/j.neuron.2011.05.020#body:66	Commissure {Organ urn:agi-ncimorgan:C1185742}
VEGFA --+> axon guidance	Regulation	VEGF mediates commissural axon guidance, directs granule cell migration in the cerebellum via VEGFR2 expressed in these cells [ ] and modulates NMDA receptor activity before synapse formation [ ].	info:doi/10.1016/j.ceb.2012.02.002#body:80	Commissure {Organ urn:agi-ncimorgan:C1185742}
VEGFA --+> axon guidance	Regulation	One would not get surprised if NrCAM is involved in angiogenesis, in light of its interaction with neuropilins, plexins, and semaphorins, all of which	info:doi/10.1016/j.mcn.2011.12.002#body:275	Blood Vessels {Organ urn:agi-ncimorgan:C0005847}

		are involved in angiogenesis, together with the recent and rather surprising findings that VEGF, a vascular growth factor, is also involved in axon guidance, supporting an idea of common molecular mechanisms shared by axon guidance and angiogenesis ( ).		
YWHAB --+> vascularization	Regulation	The 14-3-3ß protein is overexpressed in K2 cells compared with untransformed rat liver tissue. 14-3-3ß downregulation through transfection with an antisense 14-3-3ß expression vector inhibits cell growth, tumorigenicity, and angiogenesis of K2 cells.	info:pmid/24468084#body:8	liver parenchyma {Organ urn:agi-ncimorgan:C0736268}
VEGFA ---> CFL1	Regulation	VEGF-A induced LIMK1 activation and cofilin phosphorylation, and this was inhibited by the p38 mitogen-activated protein kinase inhibitor SB203580.	info:pmid/16456544#abs:4	
VEGFA ---> CFL1	Regulation	Inhibition of JNK1/2, c-Src, and phosphatidylinositol 3-kinase/Akt suppressed VEGF-induced stress fiber formation and cofilin-1 phosphorylation. c-Src inhibition suppressed VEGF-induced phosphorylation of focal adhesion kinase, paxillin, and focal adhesion.	info:pmid/20463056#abs:4	Arteries {Organ urn:agi-ncimorgan:C0003842}
VEGFA ---> CFL1	Regulation	We also found that recombinant human ChM-I inhibits VEGF-A-induced Ser-3 phosphorylation of cofilin and possibly enhances the actin depolymerization in the Human umbilical vein endothelial cells and the MSS31 cells ( ).	info:pmid/20026108#body:202	
PGK1 ---| VEGFA	MolTransport	Overexpression of PGK1 reduced the secretion of vascular endothelial growth factor and interleukin-8 and increased the generation of angiostatin.	info:pmid/17210694#abs:4	
PGK1 ---| VEGFA	MolTransport	Overexpression of phosphoglycerate kinase 1 reduced the secretion of vascular endothelial growth factor and decreased tumor angiogenesis.	info:pmid/19299076#body:144	
VEGFA ---| MDK	Regulation	Among the 96 genes on the array, 25 genes were differentially expressed, of which 24 were upregulated and one (midkine) was downregulated	info:pmid/15765121#body:180	Blood Vessels {Organ urn:agi-ncimorgan:C0005847}

		in the VEGF/MT tumors.		
MDK ---> VEGFA	Regulation	We propose a model in which MDK is a new modulator of the VEGF-A-VEGFR-2 axis.	info:pmid/18392135#abs:6	Microvessels {Organ urn:agi-ncimorgan:C2350570}
MDK ---> VEGFA	Regulation	Further studies on the transcriptional regulation of the VEGF gene mediated by midkine are needed; currently, in vitro studies are being conducted to investigate midkine-VEGF cross-talking during tumour angiogenesis.	info:pmid/17110085#body:89	
HSPD1 ---| VEGFA	Expression	The DNA-hsp65 treatment blocked the expression of VEGF in mice tuberculous meningitis.	info:pmid/23491717#abs:10	Brain {Organ urn:agi-ncimorgan:C1269537}
HSPD1 ---| VEGFA	Expression	vascular endothelial growth factor expression was downregulated by HSP65 vaccination.	info:pmid/23135134#cont:185	Heart {Organ urn:agi-ncimorgan:C1281570}
HSPD1 ---| VEGFA	Expression	Knockdown of TLR2 significantly attenuated the HSP60-stimulated expressions of VEGF (P<0.01), IL-10 (P<0.01), and the phosphorylation of Stat3 (P<0.01) (Fig. S5B-D).	info:pmid/19654875#body:187	
HSPD1 ---| VEGFA	Expression	The mechanism of tumor vessel attenuation in this model may be as follows: Firstly, HSP65-X10-ßhCGCTP37 may attenuate angiogenesis through vascular endothelial growth factor.	info:pmid/19913113#body:207	
YWHAB ---> VEGFA	Expression	The deregulated expression of 14-3-3ß largely participated in tumorigenic angiogenesis through the constitutive stimulation of VEGF expression.	info:pmid/20388496#body:44	
YWHAB ---> VEGFA	Expression	Reduced 14-3-3ß levels have been shown to inhibit VEGF production and angiogenesis and to increase apoptosis and suppress tumor size, while downregulation of 14-3-3ß by antisense 14-3-3ß RNA suppresses hepatoma cell growth both in vitro and in vivo .	info:pmid/24268498#body:87	
PKM --+> VEGFA	Expression	PKM2 binding enhances the expression of hypoxia-inducible factor 1 target genes ( ) including LDHA, PDK1, and VEGFA (encoding the vascular endothelial growth factor) ( ).	info:pmid/24508027#body:93	

PKM --+> VEGFA	Expression	In addition to its effects on transcription of metabolic genes, PKM2 stimulates HIF-1- and HIF-2-mediated expression of the VEGFA gene (which encodes vascular endothelial growth factor), thereby promoting angiogenesis ( ) .	info:pmid/22824010#body:81	
PKM --+> VEGFA	Expression	Moreover, T454A mutant PKM2 was significantly less potent to activate endogenous HIF-1a target genes, such as LDHA, PDK1, ENO1, VEGF, and GLUT1, in HEK293T, HepG2, or HeLa cells (Fig. 7, b– d).	info:pmid/24142698#cont:237	
PKM --+> VEGFA	Expression	However, the effect of PKM2 coactivator function was not restricted to genes encoding metabolic enzymes, as recruitment of hypoxia-inducible factor 1a and hypoxia-inducible factor 1ß to the VEGF gene was also impaired by PKM2 knockdown.	info:pmid/21785006#cont:138	
PKM --+> VEGFA	Expression	Furthermore, nuclear PKM2 is a coactivator of hypoxia-inducible factor 1 and promotes transactivation of hypoxia-inducible factor 1 target genes, including lactate dehydrogenase A (LDHA), phosphoinositide-dependent kinase-1 (PDK1) and vascular endothelial growth factor A .	info:pmid/23880164#body:99	
PKM --+> VEGFA	Expression	PKM2 also stimulates hypoxia-inducible factor 1/HIF-2-dependent VEGF gene expression in hypoxic HeLa cells, suggesting that, through its function as a hypoxia-inducible factor 1 coactivator, PKM2 may play a far broader role in promoting cancer progression than has been appreciated heretofore.	info:pmid/21620138#body:177	
HNRNPK --+> VEGFA	Expression	This study shows that hnRNP K augments efficiency of VEGF mRNA translation stimulated by ANG II.	info:pmid/17581920#abs:10	
HNRNPK --+> VEGFA	Expression	PKCdelta-mediated phosphorylation of hnRNP K is required for Ang II stimulation of VEGF mRNA translation.	info:pmid/18295448#abs:10	cortex of kidney {Organ urn:agi-ncimorgan:C0022655}
HNRNPK --+> VEGFA	Expression	angiotensin II stimulation of vascular endothelial growth factor mRNA translation partly depended on increased binding of hnRNPK to the 3' end of	info:pmid/16959824#body:323	cortex of kidney {Organ urn:agi-ncimorgan:C0022655}

		vascular endothelial growth factor mRNA (111).		
HNRNPK --+> VEGFA	Expression	Recently, it was proposed that hnRNP K activates the VEGF-A promoter by binding to unwound superhelical single stranded C-rich sequences upstream of the transcription start site and to support association of transcription initiation factors [27]. hnRNP K is composed of modular regions that confer binding both to RNA or DNA as well as protein-protein interaction domains [28].	info:pmid/22879910#cont:39	
HNRNPK --+> VEGFA	Expression	Binding of hnRNP K to VEGF mRNA plays a regulatory role in the stimulation of VEGF mRNA translation by Ang II .	info:doi/10.1016/j.cellsig.2008.01.016#body:106	
PEBP1 ---> synaptic transmission	Regulation	Likewise, isoaspartate-rich and potentially dysfunctional a-synuclein, ß-synuclein, UCHL1, PEBP, clathrin light chains a and b, calreticulin, calmodulin, and synapsin 1 (a protein whose functions include tethering synaptic vesicles to the cytoskeleton (15, 21, 63)), could drive the aberrant synaptic transmission recorded in these mice (13, 14, 16, 17).	info:pmid/16923807#body:295	Brain {Organ urn:agi-ncimorgan:C1269537}
VEGFA ---> hippocampus morphology	Regulation	In addition to guidance processes, VEGF directly controls survival of subsets of neuronal populations [ ] and modulates neuronal plasticity and memory functions [ ], demonstrating that VEGF affects cognition processes by hippocampal circuits.	info:doi/10.1016/j.ceb.2012.02.002#body:83	
YWHAE ---> neurogenesis	Regulation	14-3-3epsilon regulates a wide range of biological processes, including cell cycle control, proliferation, and apoptosis, and plays a significant role in neurogenesis and the formation of malignant tumours.	info:pmid/20565895#abs:1	
ENO1 ---> vascularization	Regulation	Recently, it has been shown that MBP-1 overexpression results in the modulation of MMP-2 expression, the inhibition of in vitro angiogenesis and the regression of primary and metastatic breast tumor growth in an immunocompetent mouse	info:pmid/20886042#body:332	

		model, [29].		
ENO1 ---> vascularization	Regulation	The critical proteins in the enolase-1-mediated active proliferation of endothelial cells and the active transformation of angiogenesis of breast cancer cells responding to hypoxia were identified using cellular and molecular biological and proteomics techniques.	info:pmid/23381546#cont:49	
ENO1 ---> vascularization	Regulation	Although pharmacological induction of VEGF and ENO1 gene expression may provide therapeutic benefits under ischemic conditions, by stimulating angiogenesis and glycolysis, respectively, agents that also induce erythropoietin expression and erythropoiesis would not be useful because of the risk of vascular accidents associated with polycythemia (Sokol et al., 1995).	info:pmid/9804609#body:183	Blood Vessels {Organ urn:agi-ncimorgan:C0005847}
MDK ---> brain development	Regulation	From in vitro cell biology studies and the embryonic expression pattern of midkine in the central nervous system it was concluded that midkine directs neurite interconnections during an early phase of brain development and may later on have a maintenance function in some restricted areas.	info:pmid/12122009#body:49	Central Nervous System {Organ urn:agi-ncimorgan:C0927232}
VEGFA --+> YWHAZ	Regulation	E, VEGF decreases binding of exogenous HDAC7 to ß-catenin but increases its binding to 14-3-3 ? proteins.	info:pmid/20224040#body:200	
VEGFA --+> YWHAZ	Regulation	We found that FLNA, vimentin, CRYAB and YWHAZ are constantly over-expressed in myeloma ECs and enhanced by VEGF, FGF2, HGF and myeloma plasma cell conditioned medium.	info:pmid/21963844#cont:133	
VEGFA --+> ENO1	Regulation	In response to hypoxia, mammalian cells express multiple gene products [including erythropoietin and vascular endothelial growth factor (VEGF)] that serve to increase O2 delivery, as well as glucose transporters and glycolytic enzymes (such as enolase 1) that allow metabolic adaptation to decreased O2	info:pmid/9804609#abs:1	

		availability.		
CFL1 ---> axon guidance	Regulation	Furthermore, COFILIN is involved in the regulation of the actin cytoskeleton and in axon guidance.	info:pmid/21563072#cont:330	
CFL1 ---> axon guidance	Regulation	The effect of Limk1/cofilin on axon growth has been clearly demonstrated in vitro, but it remains unclear whether Limk1/ cofilin acts as an intracellular effector of axon guidance in vivo.	info:pmid/21084599#cont:42	Commissure {Organ urn:agi-ncimorgan:C1185742}
PKM ---> neurogenesis	Regulation	Thus, impairment of pyruvate kinase M2 and other neural progenitor cell proteins via nitration may contribute to impairment of hippocampal neurogenesis in the setting of METH abuse.	info:pmid/21708025#cont:233	Hippocampus {Organ urn:agi-ncimorgan:C0019564}
PRDX1 ---> vascularization	Regulation	These results suggest a mechanism by which peroxiredoxin 1 regulates angiogenesis in CaP and suggests a scenario of continued stimulation of angiogenesis and inflammation in CaP.	info:pmid/23185615#cont:334	
PRDX1 --+> VEGFA	Expression	The mechanism by which Peroxiredoxin 1 regulates VEGF expression in normoxic conditions was investigated in the current study.	info:pmid/23185615#abs:5	
EMX1 ---| hippocampus morphology	Regulation	Because the hippocampal morphology was severely disrupted in Emx1-Dicer cko brains by embryonic day 18.5, mostly due to reduced	info:pmid/21991391#cont:128	Brain {Organ urn:agi-ncimorgan:C1269537}
PRDX2 --+> vascularization	Regulation	Furthermore, PrxII deficiency suppresses tumor angiogenesis in vivo.	info:pmid/22099303#abs:7	
PEBP1 ---> brain development	Regulation	The HCNP precursor protein , composed of 186 amino acids, is an inhibitory factor of the c-Raf/MEK cascade and may be involved in fetal rat brain development via the inhibition of phosphorylation of Erk.	info:pmid/20682295#abs:2	medial septal nucleus {Organ urn:agi-ncimorgan:C0175233}
