# Supplementary material for: Deciphering the Role of Emx1 in Neurogenesis: A Neuroproteomics Approach
Source: Front Mol Neurosci. 2016 Oct 17;9:98. doi: 10.3389/fnmol.2016.00098 (PMC5065984; doi:10.3389/fnmol.2016.00098)
Supplement: Supplementary file 8 [file DataSheet1.docx]

**Supplementary Materials and Methods**

**Gel staining with silver or Coomassie Blue**

Gels were stained with silver staining according to the manufacturer’s instructions (Pierce Silver Stain Kit, ThermoFisher). In brief, gels were washed twice in ultrapure water, fixed for 15 minutes at RT 10 % (v/v) acetic acid solution (ACS Plus grade, Fisher) in 30 % (v/v) ethanol. Afterwards gels were washed 3 times for 20 min with 30 % (v/v) ethanol. Gels were incubated in sensitizer solution 1 minute and stained for 30 minutes. Gels were incubated with developer solution, washed with water and stopped with 5% acetic acid. Brown protein spots were detectable. Gels for mass spec analysis were stained with Bio-Rad Coomassie Blue R250 for 20 min and destained in 40% HPLC grade ethanol /10% acetic acid.

**In-gel digestion procedure**

For in-gel digestion procedures, manual in-gel digestion was performed according to an established protocol ([Pu, Amoscato, Bier, & Lazo, 2002](#_ENREF_59)). Briefly, spots selected for protein identification were excised from Coomassie stained gels. Each gel slice was diced into small pieces, put into 150 µl of 25 mM NH_4_HCO_3_ (Fluka) / 50 % (v/v) acetonitrile (ACN) (Burdick-Jackson, HPLC grade) and vortexed for 10 min. The supernatant was extracted and discarded. This process was repeated until the gel pieces were colorless. Afterwards gel pieces were covered with 100 % (v/v) ACN and let stand until the gel pieces were shrunk and turned white. After removing of ACN, gel pieces were put into a speed vac until dryness. 25 µl of trypsin solution (Promega) was added and gel pieces were incubated for 60 min at 4 °C. Any trypsin which was not absorbed into the gel was aspirated. About 25 µl of 25 mM NH_4_HCO_3_ (Fluka) was added to cover the gel pieces. After spinning down briefly, tubes were incubated at 37 °C for at least 12 hrs. After incubation samples were spinned down again and supernatants were collected into a 0.5 ml tube. 25 µl of 50 % (v/v) ACN/ 0.1 % (v/v) trifluoroacetic acid (Pierce) was added and samples were vortexed for 15 min. After centrifugation supernatants were collected again into the same tube and the whole process was repeated. A total of about 75 µl of combined peptide extract was put into the speed vac until approximately 10 µl were left over. Extracts were now ready to use for spectrometric analysis.

**Western blot analysis**

Cell lysates were prepared by incubating dissociated neurospheres in lysis buffer (50 mmol/L Tris-Cl, pH 7.4, 150 mmol/L NaCl, 1% Triton X-100, 2 mmol/L EDTA, 10 mmol/L NaF, 1 mmol/L Na_3_VO_4_, and protease inhibitors Complete, Roche, Mannheim, Germany) and subsequent use of a 23 Gauge syringe. Lysates were cleared from cellular debris by centrifugation, and protein concentrations were determined using the DC protein assay (Bio-Rad, Hercules, CA, USA). Proteins were denatured in Laemmli loading buffer at 95 °C for 5 min and 15-30 µg per lane were resolved by sodium dodecyl sulfate–polyacrylamide gel electrophoresis on 12% gels. Proteins were blotted onto polyvinylidene difluoride membranes (Millipore, Temecula, CA, USA). Membranes were blocked with QuickBlocker (EMD, San Diego, CA, USA) for 1 h at ambient room temperature, and probed overnight at 4°C with antibodies against Actin (Santa Cruz Biotechnology, Inc., Santa Cruz, CA, USA), specific to Cofilin1 and P-Cofilin1 (Abcam, Cambridge, CA, USA). Binding was detected using secondary anti-goat or anti-rabbit horseradish peroxidase conjugated antibodies (Sigma Aldrich, St. Louis, MO, USA). Immunoreactive bands were visualized using chemiluminescent ECL plus substrate (Amersham Biosciences, Piscataway, NJ, USA). Blot membranes were stained with Coomassie Brilliant Blue G-250 (Bio-Rad, Hercules, CA, USA) to assure that comparable protein amounts had been loaded and transfer was proper. For analysis of Cofilin1 phosphorylation, cells were incubated with or without 10 ng/mL recombinant human VEGF_165_ (R&D Systems, Minneapolis, USA) for 5 min, 1 h or 24h as indicated.

**Gene Ontology: Molecular Function, Biological Process and Cellular Localization of Emx1 altered Proteome**

Using PANTHER software, Emx altered proteome (38 proteins) was used to for gene ontology classification. Differential proteins assessed for molecular function were sorted into 8 categories. Similarly, 76 assignments were obtained for Biological Process and sorted into 12 biological process classifications. This is because some proteins may be assigned with more than 1 molecular function and biological process. A detailed list of protein assignments to each class is provided in the pie chart representing percentage of the functional categories of altered proteins shown in the **Supple fig 2 and 3**. Cellular localization of the proteins showed cytoplasmic, cytoskeletal, mitochondrial and neural distribution as shown in **Supple fig 4.**

**Supplemental Figure Legends**

**Supplemental Figure 1. VEGF increases NSC proliferation and survival**. VEGF-A increased NSC proliferation and blockade of VEGFR2 reduced self-renewal. **A**, NSCs were plated onto poly-L-ornithine coated plates at 10^5^/well (24-well plate). 24 hrs after plating, recombinant mouse VEGF-A (100 ng/ml, R&D system) was added to culture medium and BrdU was added into culture 24 hrs later at 10 mM for 4 or 17 hrs as indicated prior to fixation with 4% PFA. The number of BrdU positive cells was estimated only in live cells. **B**, 400 dissociated NSCs (from the second passage) were plated per well in a 6-well plate (no coating). SU1498 was added into culture at concentrations as indicated. The number of neurospheres per well was counted two weeks later and results compared by one-way ANOVA (*: p<0.05; **: p<0.01; #: p<0.001). N=6/group.

**Supplemental Figure 2. Molecular Function gene ontology (GO) analysis depicting Emx1 altered proteins are shown in pie chart.** Data indicate 8 subcategories distribution with a majority of catalytic, structural and binding functions among others.

**Supplemental Figure 3. Pie Chart of Biological processes GO analysis depicting Emx1 altered proteins.** Data indicate 12 subcategories distribution with a majority of developmental, cell communication and metabolic processes.

**Supplemental Figure 4. Cellular Localization GO analysis depicting Emx1 altered proteins distribution.** Proteins localization showed cytoplasmic, cytoskeletal, mitochondrial and neural distribution.

**Supplemental Tables**

**Supplemental Table 1. Altered differential upregulated proteins identified in the *Emx1* KO vs. WT with their mass spectrometry data.** A total of 33 proteins were differentially upregulated in the *Emx1* KO vs. WT proteome on the 2-D gel. Differential proteins are presented with their detailed full mass spectrometry data including, Mwt, peptide sequence, % coverage, etc.

**Supplemental Table 2. Altered differential downregulated proteins identified in the *Emx1* KO vs. WT with their mass spectrometry data.** A total of 5 differentially altered proteins were shown to be downregulated in the *Emx1* KO vs. WT proteome on the 2-D gel. Differential proteins are presented with their detailed full mass spectrometry data including, Mwt, peptide sequence, % coverage, etc.

**Supplemental Table 3: *In silico* validation** **of the cellular differentiation cluster analysis**. List of unique proteins identified per cluster is listed. Shown are the protein entity involved in this cellular differentiation biological process (a), along with the interaction type and directionality: inhibitory, regulatory, binding etc. (b) and finally the PubMed reference utilized to derive these interaction type (c).

**Supplemental Table 4: *In silico* validation of the cellular migration cluster analysis**. List of unique proteins identified per cluster is listed. Shown are the protein entity involved in this cellular migration biological process (a), along with the interaction type and directionality: inhibitory, regulatory, binding etc. (b) and finally the PubMed reference utilized to derive these interaction type (c).

**Supplemental Table 5: *In silico* validation of the cell proliferation cluster analysis**. List of unique proteins identified per cluster is listed. Shown are the protein entity involved in this cell proliferation biological process (a), along with the interaction type and directionality: inhibitory, regulatory, binding etc. (b) and finally the PubMed reference utilized to derive these interaction type (c).

**Supplemental Table 6: *In silico* validation of the cytoskeletal organization cluster analysis**. List of unique proteins identified per cluster is listed. Shown are the protein entity involved in this cytoskeletal organization biological process (a), along with the interaction type and directionality: inhibitory, regulatory, binding etc. (b) and finally the PubMed reference utilized to derive these interaction type (c).

**Supplemental Table 7: *In silico* validation of the Global interaction Map and Enriched biological process and cellular pathways involved**. A global non-targeted approach was applied to assess the altered pathways implicated with the Emx1 differentially expressed proteome. List of proteins and unique pathways is listed. Shown are the protein entity involved in this cytoskeletal organization biological process (a), along with the interaction type and directionality: inhibitory, regulatory, binding etc. (b) and finally the PubMed referenced utilized to derive these interaction type (c).
