# Supplementary figures and images for: Deciphering the Role of Emx1 in Neurogenesis: A Neuroproteomics Approach
Source: Front Mol Neurosci. 2016 Oct 17;9:98. doi: 10.3389/fnmol.2016.00098 (PMC5065984; doi:10.3389/fnmol.2016.00098)

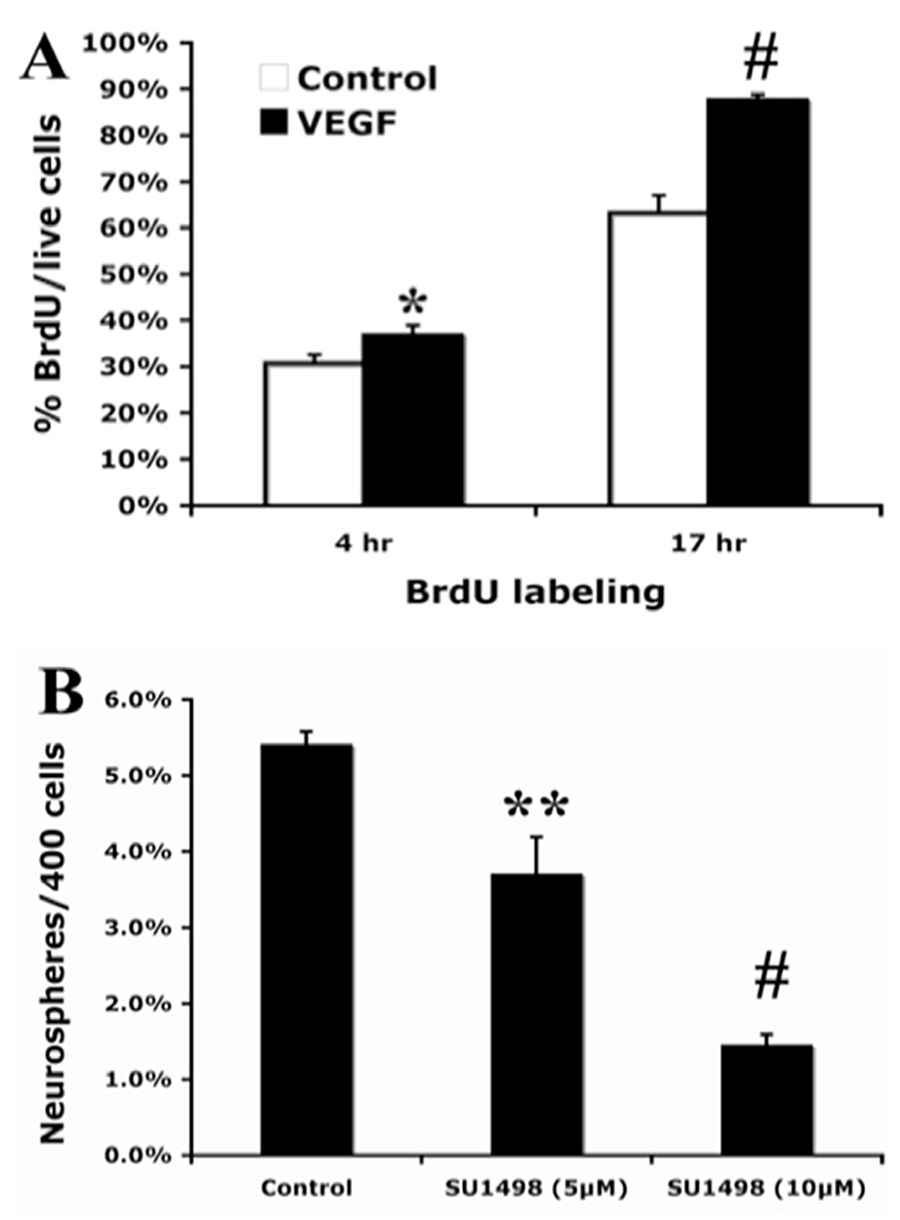

Supplement: Supplementary file 9 [file Image1.TIF]

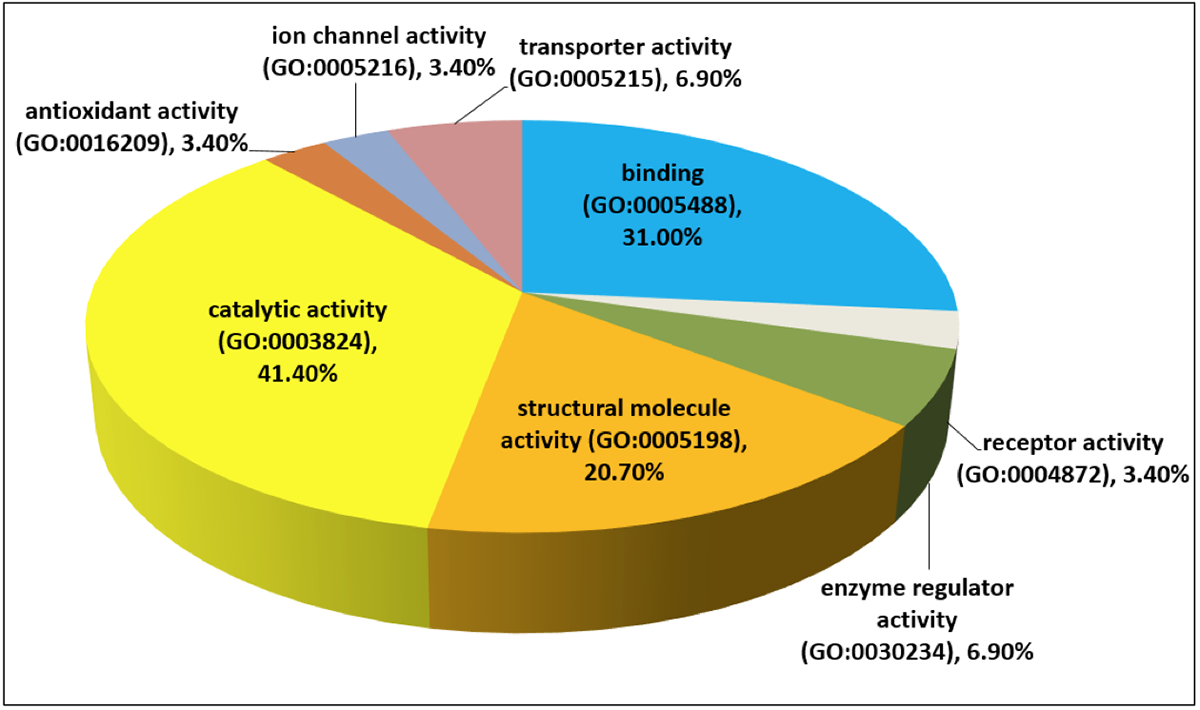

Supplement: Supplementary file 10 [file Image2.tif]

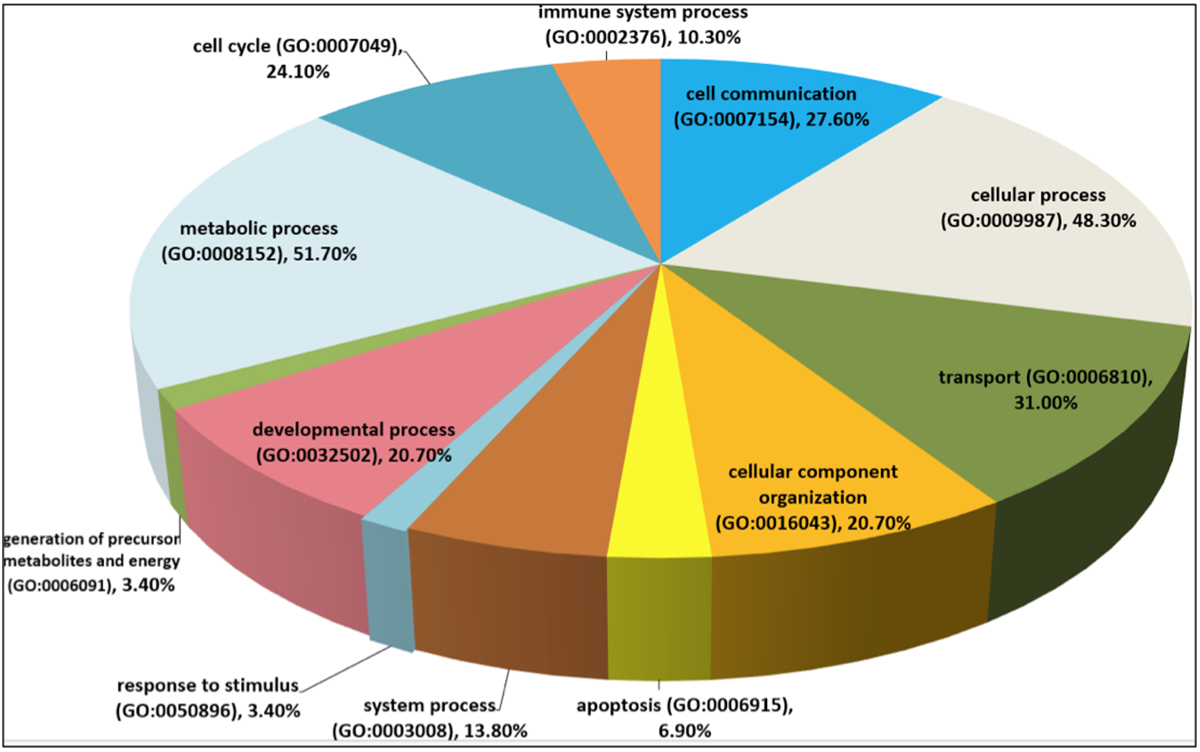

Supplement: Supplementary file 11 [file Image3.tif]

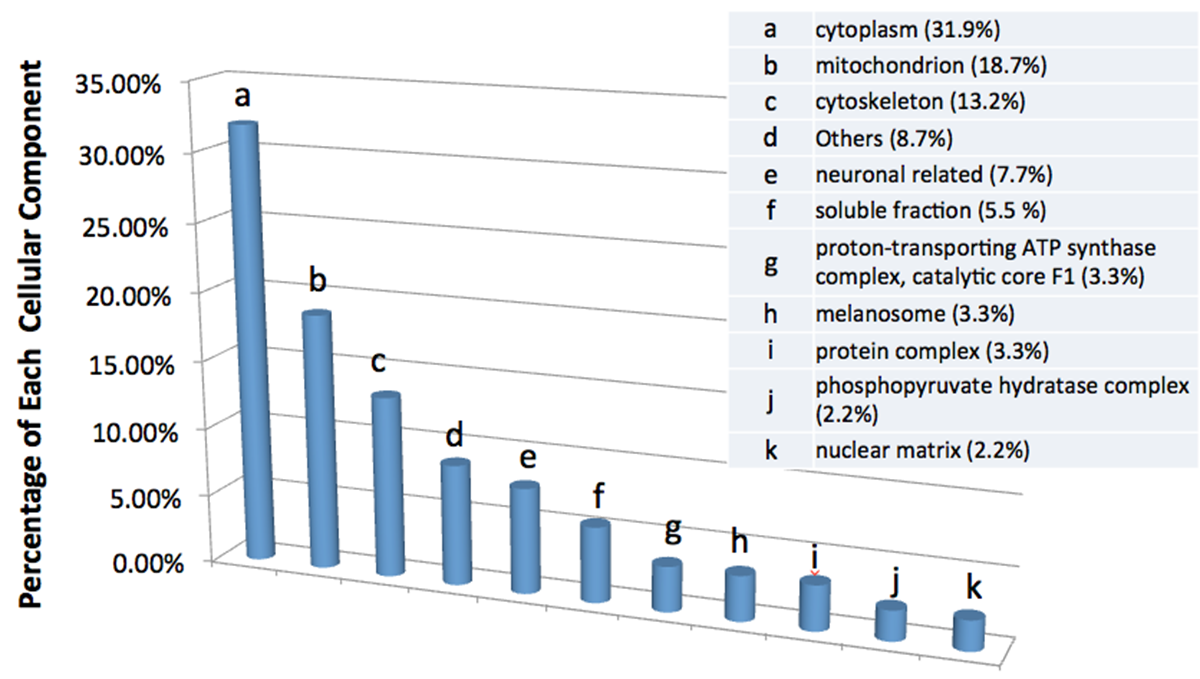

Supplement: Supplementary file 12 [file Image4.tif]
